# Author Correction: Identification and characterization of endo-α- exo-α-, and exo-β-D-arabinofuranosidases degrading lipoarabinomannan and arabinogalactan of mycobacteria

**DOI:** 10.1038/s41467-023-42065-0

**Published:** 2023-10-09

**Authors:** Michiko Shimokawa, Akihiro Ishiwata, Toma Kashima, Chiho Nakashima, Jiaman Li, Riku Fukushima, Naomi Sawai, Miku Nakamori, Yuuki Tanaka, Azusa Kudo, Sae Morikami, Nao Iwanaga, Genki Akai, Nobutaka Shimizu, Takatoshi Arakawa, Chihaya Yamada, Kanefumi Kitahara, Katsunori Tanaka, Yukishige Ito, Shinya Fushinobu, Kiyotaka Fujita

**Affiliations:** 1 Faculty of Agriculture, Kagoshima University, Kagoshima, 890-0065 Japan; 2 Cluster for Pioneering Research, RIKEN, Saitama, 351-0198 Japan; 3 Department of Biotechnology, The University of Tokyo, Bunkyo-ku, Tokyo 113-8657 Japan; 4 Photon Factory, Institute of Materials Structure Science, High Energy Accelerator Research Organization (KEK), Tsukuba, Ibaraki 305-0801 Japan; 5 Faculty of Pharmaceutical Sciences, Tokyo University of Science, Noda, Chiba 278-8510 Japan; 6 School of Agriculture, Meiji University, Kawasaki, Kanagawa 214-8571 Japan; 7 Department of Chemical Science and Engineering, Tokyo Institute of Technology, Meguro-ku, Tokyo 152-8552 Japan; 8 Graduate School of Science, Osaka University, Osaka, 560-0043 Japan; 9 CRIIM, The University of Tokyo, Bunkyo-ku, Tokyo 113-8657 Japan

**Keywords:** X-ray crystallography, Glycobiology, Enzyme mechanisms, Bacterial genes

Correction to: *Nature Communications* 10.1038/s41467-023-41431-2, published online 19 September 2023

The original version of the Supplementary Information associated with this Article contained an error on page 38, which incorrectly read ‘The details of the experimental conditions and analysis of these SEC-SAXS experiments are summarized in Supplementary Table 4.’ The correct version states ‘Dataset 1’ in place of ‘Supplementary Table 4’. The HTML has been updated to include a corrected version of the Supplementary Information.

The original version of this Article contained an error in the Data Availability statement, which incorrectly omitted the following: ‘The SAXS data has been submitted to SASBDB with the deposition ID of SASDQQ8 [https://www.sasbdb.org/data/SASDQQ8/]’. This has been corrected in both the PDF and HTML versions of the Article.

### Supplementary information

Updated Supplementary Information

