# Supplementary material for: Identification and characterization of endo-α-, exo-α-, and exo-β-d-arabinofuranosidases degrading lipoarabinomannan and arabinogalactan of mycobacteria
Source: Nat Commun. 2023 Sep 19;14:5803. doi: 10.1038/s41467-023-41431-2 (PMC10509167; doi:10.1038/s41467-023-41431-2)
Supplement: Supplementary file 1 — Supplementary Information File [file 41467_2023_41431_MOESM1_ESM.pdf]

# Supplemental Information for Shimokawa *et al.*

## Supplementary Figures


**Supplementary Fig. 1. Time course of MsLAM degradation by nEndoMA**

**Supplementary Fig. 2. Selection procedure for candidate genes for endo-D-arabinanase**

**Supplementary Fig. 3. Sugar induction of the D-arabinan degradation PUL genes**

**Supplementary Fig. 4. Domain structures of the D-arabinan degrading enzymes**

**Supplementary Fig. 5. Purification and characterization of recombinant EndoMA1 and EndoMA2**

**Supplementary Fig. 6. Degradation of synthetic oligo-D-arabinofuranosides by EndoMA1 and EndoMA2**

**Supplementary Fig. 7. ESI-TOF MS analysis of the hydrolysis of A22BβT by EndoMA1**

**Supplementary Fig. 8. NMR analysis of the transglycosylation product by EndoMA1**

**Supplementary Fig. 9. Analysis of the purified samples of ExoMA1 and ExoMA2**

**Supplementary Fig. 10. Substrate specificity and kinetic analysis of ExoMA1**

**Supplementary Fig. 11. Substrate specificity and kinetic analysis of ExoMA2**

**Supplementary Fig. 12. NMR analysis of the anomer-retaining transglycosylation and hydrolysis reactions of ExoMA2**

**Supplementary Fig. 13. Cooperative action of D-arabinan degrading enzymes**

**Supplementary Fig. 14. Electron density maps of the bound oligo-D-arabinofuranosides and quaternary structure of EndoMA1 in the crystal structure**

**Supplementary Fig. 15. Size exclusion chromatography and SDS-PAGE of EndoMA1**

**Supplementary Fig. 16. Results of SEC-MALS/RI and SEC-SAXS measurements for EndoMA1**

**Supplementary Fig. 17. Environment of Glu243 and EndoMA1 mutant activity**

**Supplementary Fig. 18. Comparison of EndoMA1 with structural homologs**

**Supplementary Fig. 19. Size exclusion chromatography, protomer interface, and electron microscopy of ExoMA1**

**Supplementary Fig. 20. Structural comparison of ExoMA1 with homolog enzymes**

**Supplementary Fig. 21. Electron density maps, quaternary structure, and size exclusion chromatography of EndoMA2**

**Supplementary Fig. 22. Phylogenetic tree of EndoMA1, EndoMA2, and DUF4185 (GH183) proteins**

**Supplementary Fig. 23. Phylogenetic tree of ExoMA1 and GH172 proteins**

**Supplementary Fig. 24. Phylogenetic tree of ExoMA2 and homologs**

Supplementary Fig. 25. Amino acid sequence alignment of ExoMA2 and GH116 proteins

## Supplementary Tables

Supplementary Table 1. General features of the draft genome of *Mi. arabinogalactanolyticum* JCM 9171

Supplementary Table 2. Previously reported NMR data of Me-α-A4B

Supplementary Table 3. Crystallographic data statistics of EndoMA1

Supplementary Table 4. Dali structural similarity search of the two domains of EndoMA1

Supplementary Table 5. Crystallographic data statistics of ExoMA1 and ExoMA2

## Supplementary Methods

Details of protein production for crystallography

Software and servers for protein crystallography

Details of SEC-MALS/RI and SEC-SAXS experiments and analysis

Synthesis of oligo-D-arabinofuranosides

## Supplementary References

## Supplementary Figures

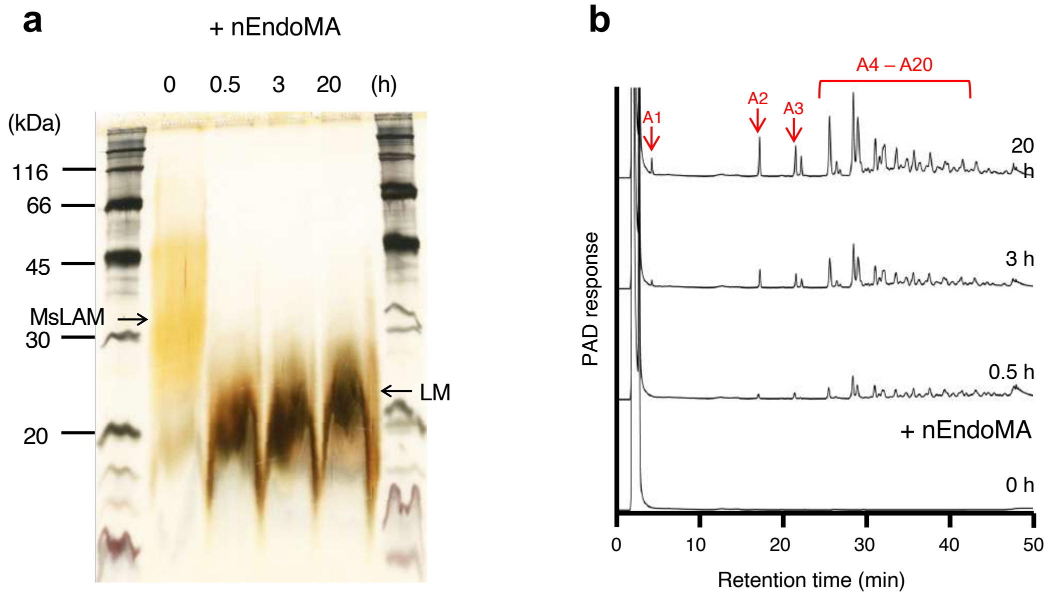


**Supplementary Fig. 1. Time course of MsLAM degradation by nEndoMA. a,** SDS-PAGE of MsLAM with silver-PAS staining. **b,** HPAEC-PAD analysis. MsLAM (0.25 mg/mL) was incubated with nEndoMA in 50 mM sodium phosphate buffer (pH 6.5) at 37°C. The reaction was stopped for 0, 0.5, 3, and 20 h by boiling the sample for 3 min.

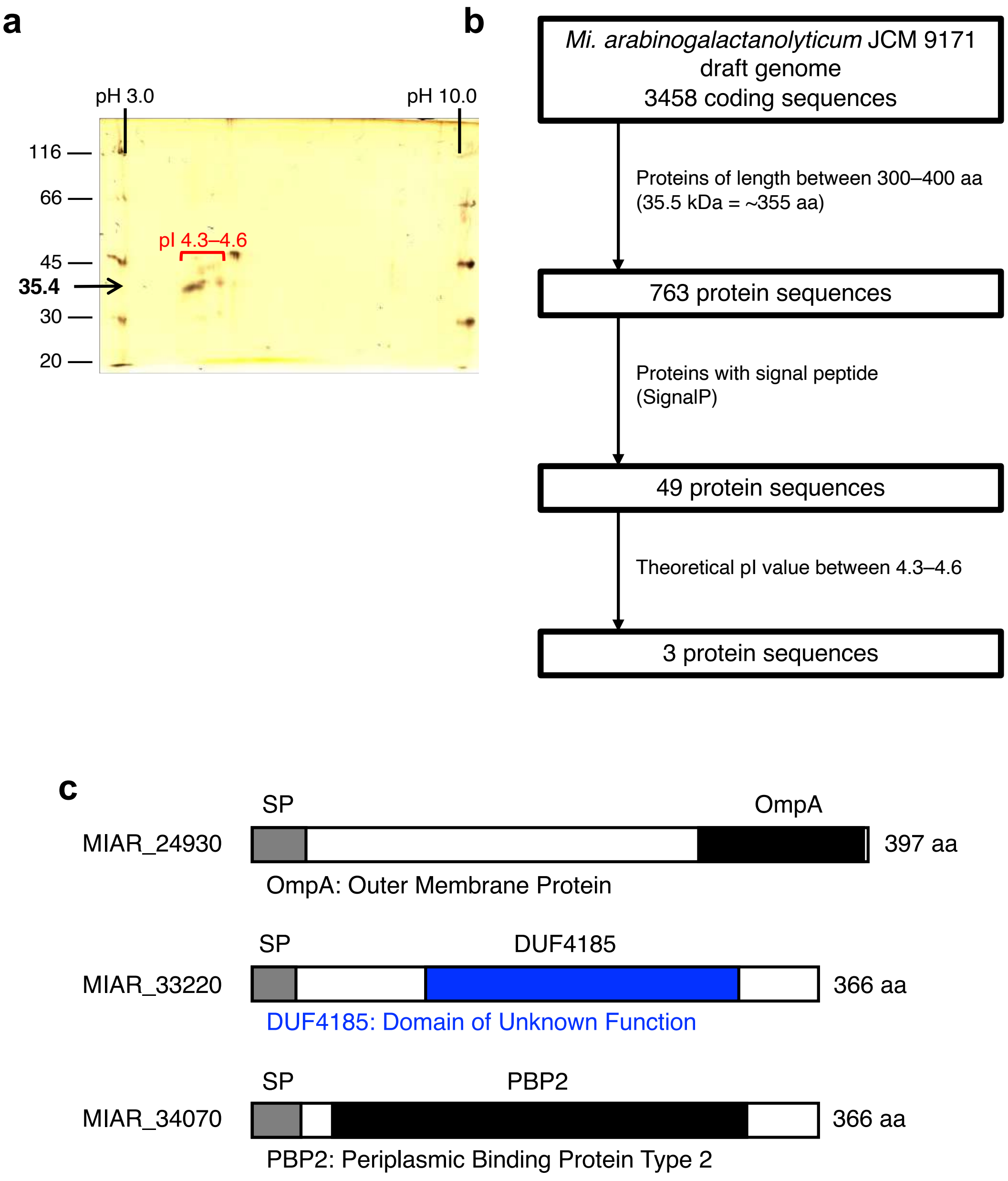


**Supplementary Fig. 2. Selection procedure for candidate genes for endo-D-arabinanase. a,** Two-dimensional electrophoresis of the purified protein (nEndoMA) with silver-staining. **b,** Flowchart of the candidate gene selection. **c,** Domain structures of the selected candidate gene products. SP: signal peptide.

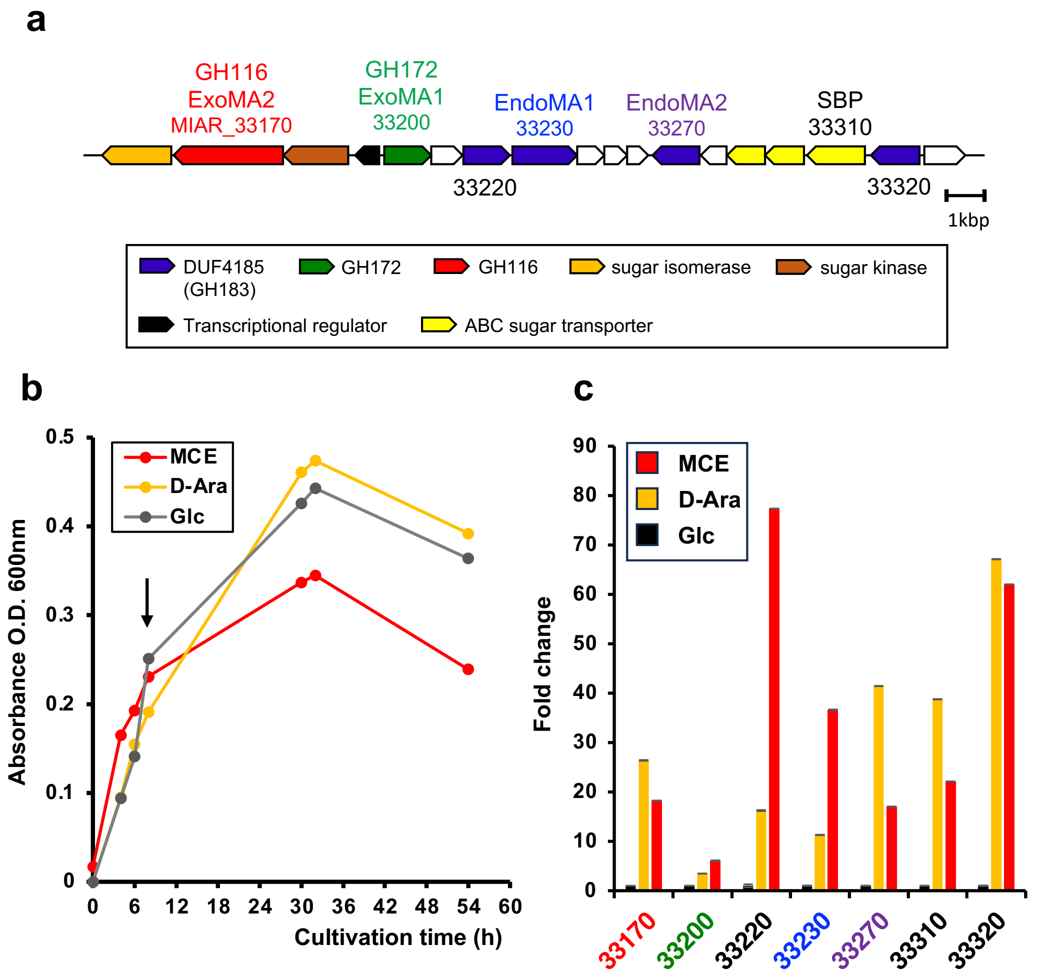


**Supplementary Fig. 3. Sugar induction of the D-arabinan degradation PUL genes. a,** The D-arabinan degradation PUL of *M. arabinogalactanolyticum*. **b,** Bacterial cells were induced by either glucose (Glc), D-arabinose (D-Ara), or mycobacterial cell wall extracts (MCE). Cells were harvested at middle exponential growth phase (shown as an arrow). **c,** Gene expression profiles of the D-arabinan degradation PUL. EndoMA1 (MIAR_33230), EndoMA2 (MIAR_33270), DUF4185_1 (MIAR_33220), DUF4185_2 (MIAR_33320), ExoMA1 (MIAR_33200), ExoMA2 (MIAR_33170), and SBP of ABC sugar transporter (MIAR_33310) genes were analyzed by qRT-PCR. The fold changes were expressed in transcription levels compared to those grown on glucose. Data are indicated as the mean ± SD (n = 3).

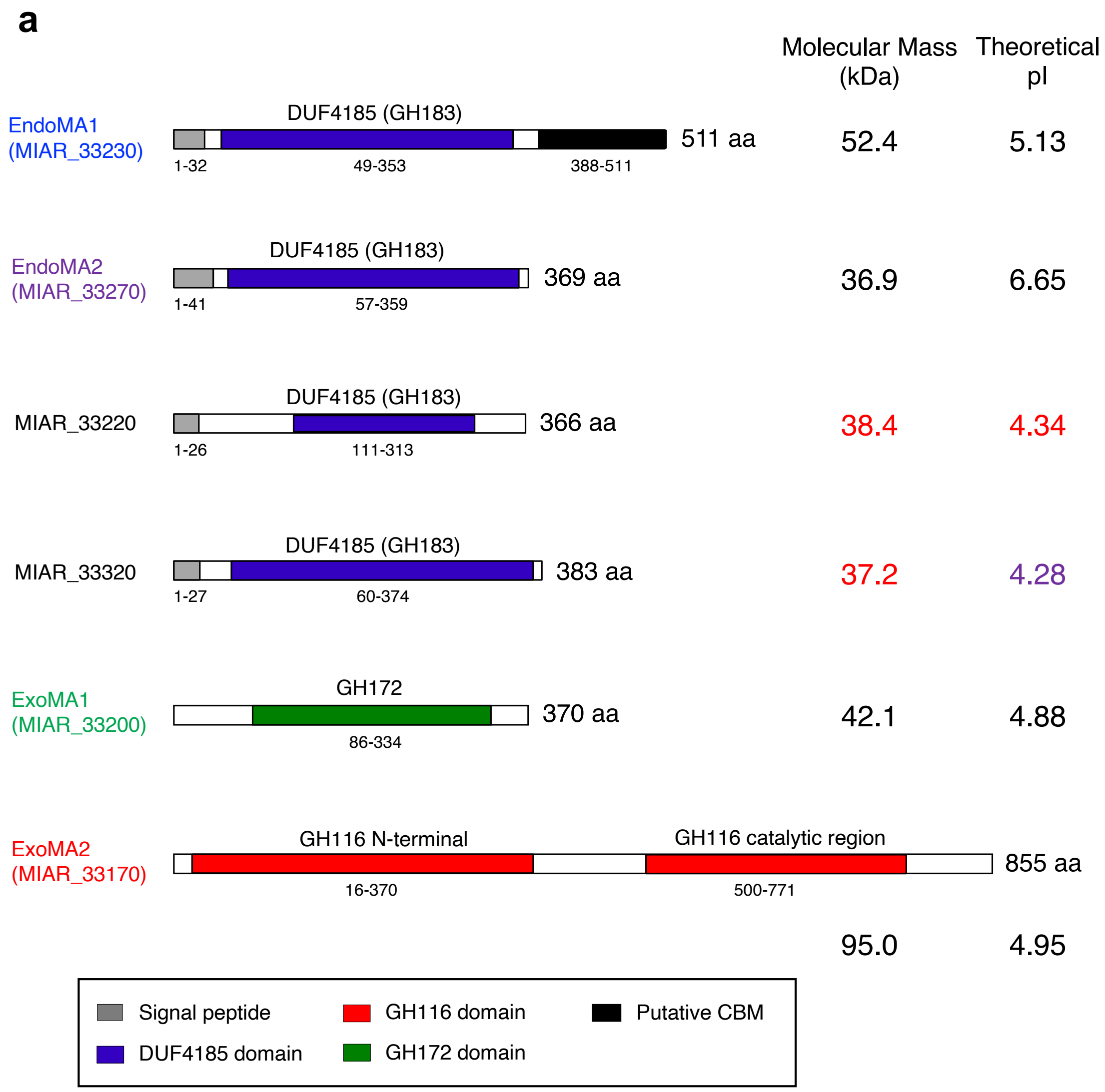


**b**

| | EndoMA2 MIAR_33270 | MIAR_33220 | MIAR_33320 |
|---|---|---|---|
| EndoMA1 MIAR_33230 | 27% | 22% | 35% |
| EndoMA2 MIAR_33270 | – | 24% | 29% |
| MIAR_33220 | | – | 22% |

**Supplementary Fig. 4. Domain structures of the D-arabinan degrading enzymes. a,** Domain structure of enzymes. Conserved domains were identified using the InterPro server (https://www.ebi.ac.uk/interpro/). The molecular mass and theoretical pI values were calculated using Genetyx software. **b,** Sequence identity of DUF4185 (GH183) proteins.

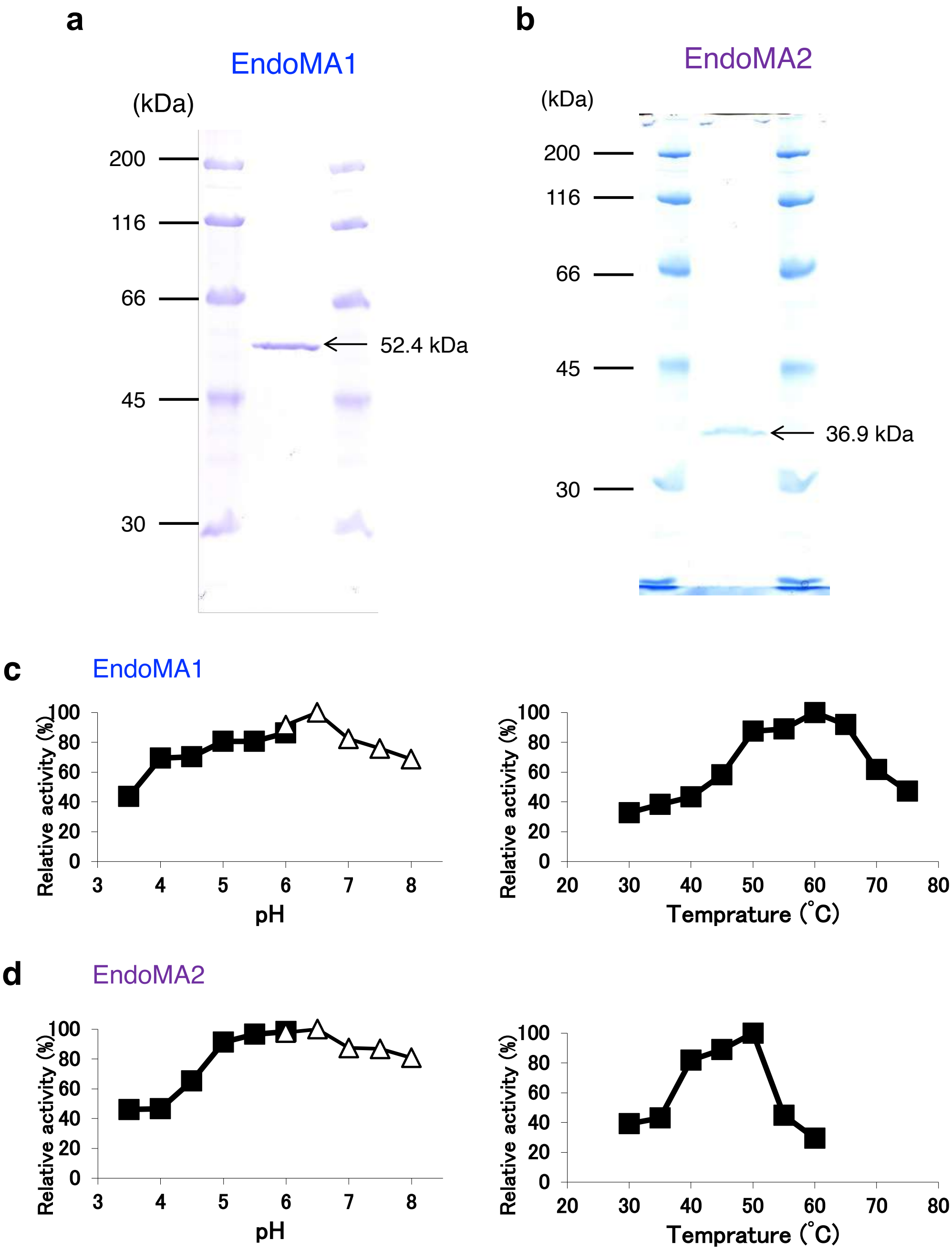


**Supplementary Fig. 5. Purification and characterization of recombinant EndoMA1 and EndoMA2. a and b,** SDS-PAGE of purified EndoMA1 (**a**) and EndoMA2 (**b**) proteins. **c and d,** pH (left) and temperature (right) profiles of the activity of EndoMA1 (**c**) and EndoMA2 (**d**). Data points measured in sodium acetate and sodium phosphate buffers are shown by closed squares and open triangles, respectively. The standard assay condition is in sodium phosphate buffer (pH 6.5) at 37°C. The reducing power of the reaction product was quantified using MtLAM as the substrate. Detailed assay conditions are described in Methods.

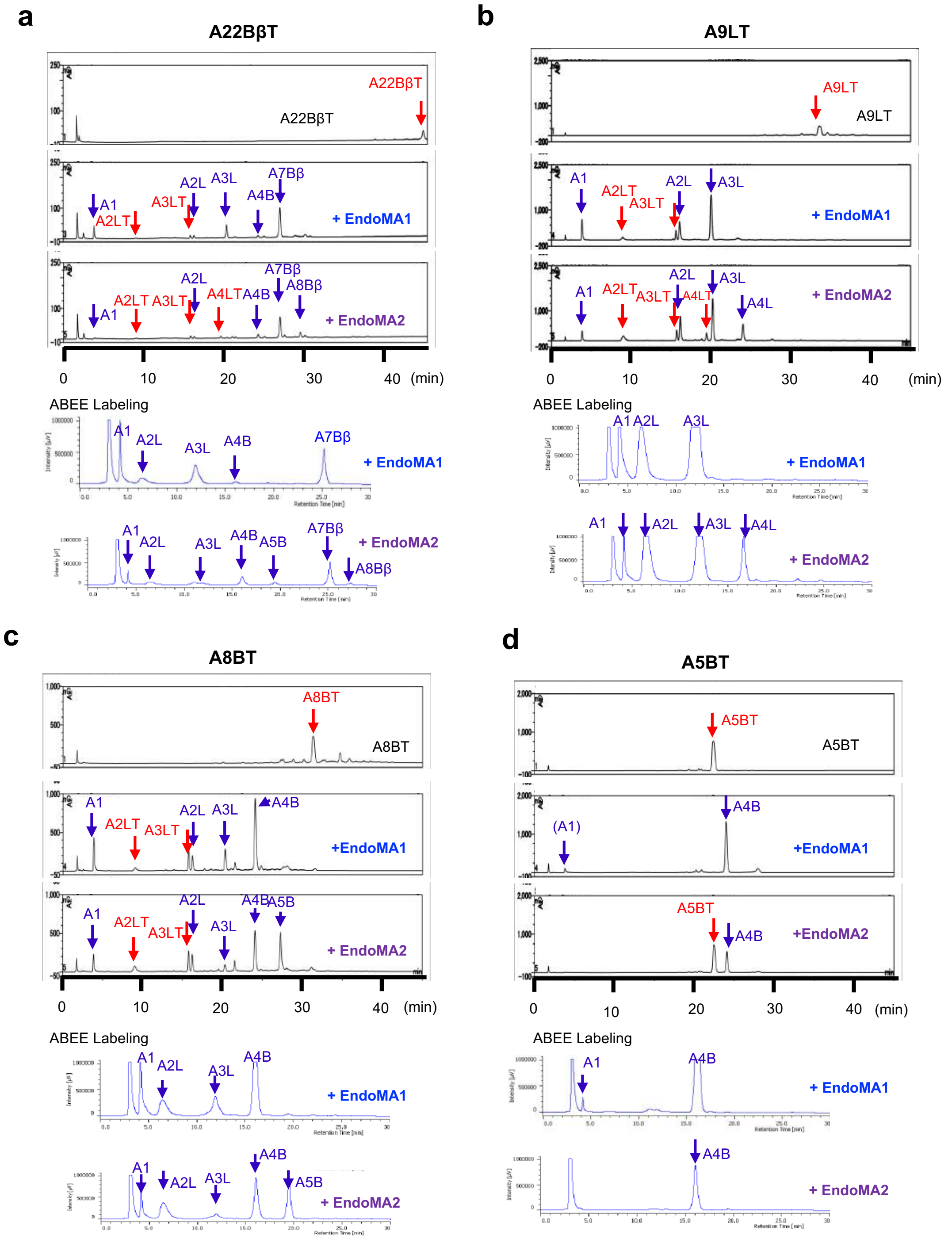


**Supplementary Fig. 6. Degradation of synthetic oligo-D-arabinofuranosides by EndoMA1 and EndoMA2. a**, A22BβT. **b,** A9LT. **c,** A8BT. **d,** A5BT. Each substrate (0.50 mg/mL) in 50 mM sodium acetate (pH 6.0) was incubated with EndoMA1 or EndoMA2 (1.0 µg/mL) at 37°C for 20 h. The top three and bottom two chromatograms in each panel are HPEAC-PAD and HPLC, monitored by fluorescence of *p*-aminobenzoic ethyl ester (ABEE), respectively. ABEE

labeling detects oligosaccharides having an enzyme-cleaved reducing end. None of the substrates had a reducing end because of the presence of the acetonide tag. The letters A, B, L, β, and T on the peaks represent arabinose, branched, linear, β-Ara*f* bond, and acetonide tag, respectively; the numbers denote the degree of polymerization. Names of the degradation products with and without an acetonide tag are indicated by red and purple colors, respectively.

**a**

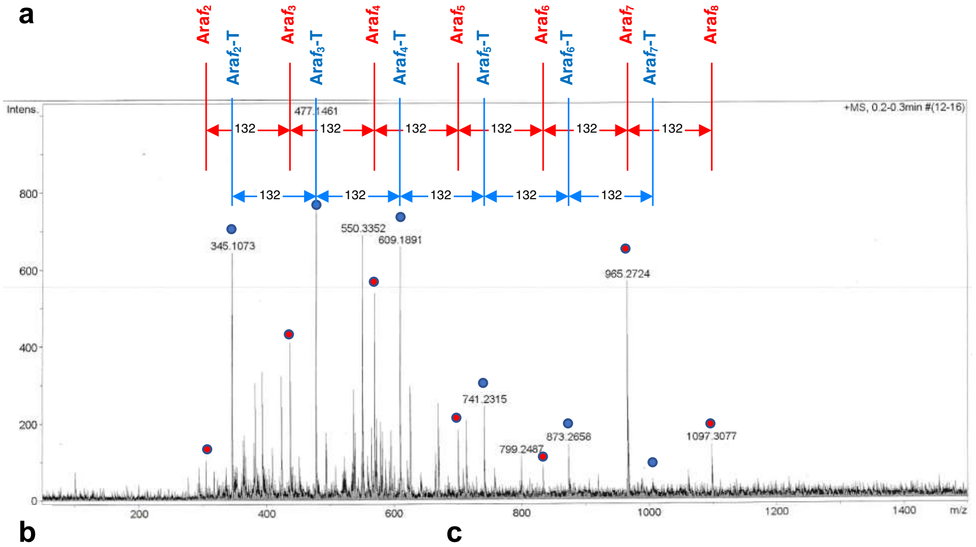


**b**

| reducing | composition | calcd | found |
|---|---|---|---|
| Ara$f_2$ | $C_{10}H_{18}Na_1O_9$ | 305.0849 | 305.0722 |
| Ara$f_3$ | $C_{15}H_{26}Na_1O_{13}$ | 437.1271 | 437.1161 |
| Ara$f_4$ | $C_{20}H_{34}Na_1O_{17}$ | 569.1694 | 569.1559 |
| Ara$f_5$ | $C_{25}H_{42}Na_1O_{21}$ | 701.2116 | 701.1965 |
| Ara$f_6$ | $C_{30}H_{50}Na_1O_{25}$ | 833.2539 | 833.2387 |
| Ara$f_7$ | $C_{35}H_{58}Na_1O_{29}$ | 965.2962 | 965.2724 |
| Ara$f_8$ | $C_{40}H_{66}Na_1O_{33}$ | 1097.3384 | 1097.3123 |

**c**

| acetonide (T) | composition | calcd | found |
|---|---|---|---|
| Ara$f_2$-T | $C_{13}H_{22}Na_1O_9$ | 345.1162 | 345.1073 |
| Ara$f_3$-T | $C_{18}H_{30}Na_1O_{13}$ | 477.1584 | 477.1461 |
| Ara$f_4$-T | $C_{23}H_{38}Na_1O_{17}$ | 609.2007 | 609.1891 |
| Ara$f_5$-T | $C_{28}H_{46}Na_1O_{21}$ | 741.2429 | 741.2315 |
| Ara$f_6$-T | $C_{33}H_{54}Na_1O_{25}$ | 873.2852 | 873.2658 |
| Ara$f_7$-T | $C_{38}H_{62}Na_1O_{29}$ | 1005.3275 | 1005.3075 |

**d**

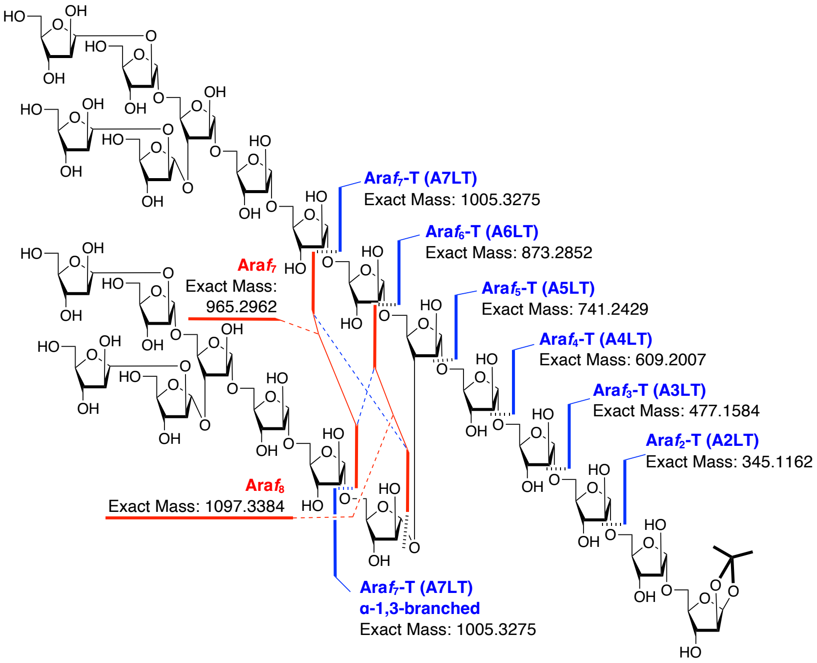


**Supplementary Fig. 7. ESI-TOF MS analysis of the hydrolysis of A22BβT by EndoMA1. a,** Mass spectrum of the sample obtained from the hydrolysis of A22BβT by EndoMA1. The red and blue circles indicate the peaks related to the reducing hemiacetals and acetonide derivatives, respectively. The assignment displayed on the spectrum is based on the analysis from (**b**) and (**c**).

The difference between peaks in both reducing (red) and acetonide-tagged (blue) oligo-D-arabinofuranosides was 132 Da which indicates the one D-arabinose residue. (**b**) Observed mass data of the reducing hemiacetals (**Ara*f*$_{2–8}$**). (**c**) Observed mass data of the acetonide derivatives (**Ara*f*$_{2–7}$-acetonide**). (**d**) The EndoMA1 cleavage site of A22BβT for major reducing hemiacetals and acetonide derivatives. The reducing fragments (**Ara*f*$_7$** and **Ara*f*$_8$**) with high intensity were obtained from the non-reducing side of A22BβT. Detection of **Ara*f*$_7$** concomitant with **Ara*f*$_8$** also in the MASS analysis indicates that the endo-cleavage of the fragments might occur by EndoMA1. Other smaller reducing fragments (**Ara*f*$_{2–6}$**) might be obtained by the action of multiple actions of EndoMA1 from initial products accepted as the substrate again.

**a**

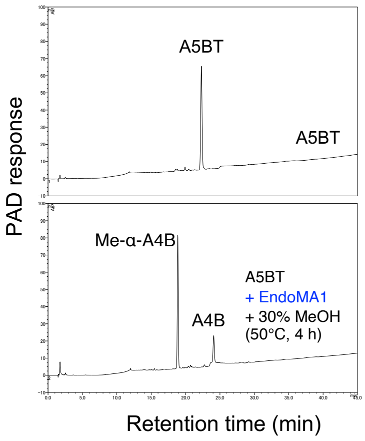


**b**

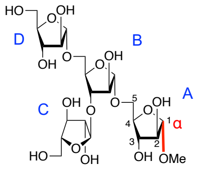


Methyl α-D-arabinofuranosyl[D]-(1→5)-[α-D-arabinofuranosyl[C]-(1→3)]-α-D-arabinofuranosyl[B]-(1→5)-α-D-arabinofuranoside[A]

| in $D_2O$, r.t. | | $^1H$/ δ ppm | mult., $^3J_{H-H}$ Hz |
|---|---|---|---|
| α-D-Araf[D](1→5) | 1 | 5.08 | s |
| | 2 | 4.12–4.14 | m |
| | 3 | 3.92–3.96 | m |
| | 4 | 4.10 | m |
| | 5 | 3.80–3.85 | m |
| | | 3.707 | dd, 12.4, 6.0 |
| α-D-Araf[C](1→3) | 1 | 5.15 | s |
| | 2 | 4.12–4.14 | m |
| | 3 | 3.92–3.96 | m |
| | 4 | 4.00–4.06 | m |
| | 5 | 3.80–3.85 | m |
| | | 3.706 | dd, 12.0, 5.2 |
| (→3,5)- α-D-Araf[B](1→ | 1 | 5.11 | s |
| | 2 | 4.29 | dd, 2.4, 0.8 |
| | 3 | 4.11 | dd, 6.0, 3.2 |
| | 4 | 4.31 | ddd, 6.0, 5.6, 3.2 |
| | 5 | 3.92–3.96 | m |
| | | 3.80–3.85 | m |
| →5)-α-D-Araf[A]- | 1 | 4.92 | s |
| | 2 | 4.05 | m |
| | 3 | 4.00–4.06 | m |
| | 4 | 4.16 | ddd, 6.0, 3.2, 1.6 |
| | 5 | 3.88 | dd, 12.0, 6.0 |
| | | 3.78 | dd, 12.0, 3.2 |
| OMe | | 3.42 | s |

For standard of $^1H$, HOD peak was used at δ 4.80 ppm.

**c**

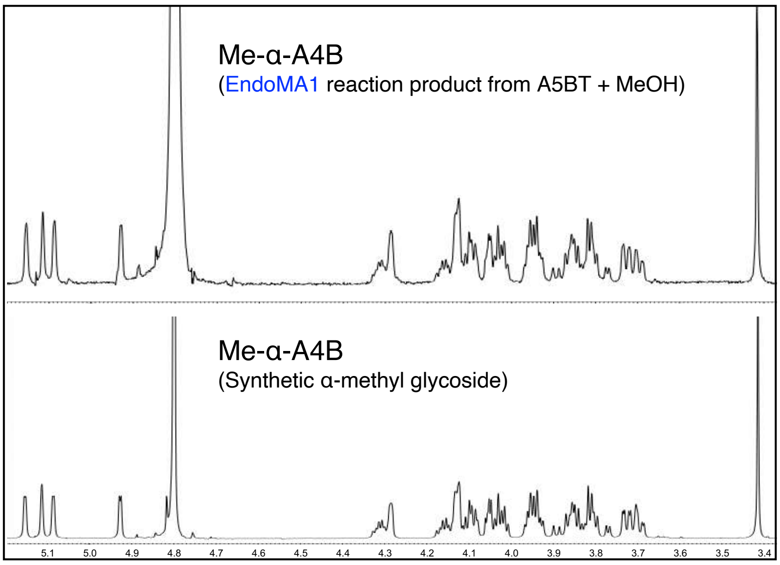


**Supplementary Fig. 8. NMR analysis of the transglycosylation product by EndoMA1.** The reaction product from A5BT in the presence of methanol was analyzed using HPAEC-PAD (**a**) and NMR (**b**). **c**, Comparison of the $^1H$ NMR spectra of the reaction product (upper panel) and the authentic sample (lower panel) of Me-α-A4B (Methyl α-D-arabinofuranosyl-(1→5)-[α-D-arabinofuranosyl-(1→3)]-α-D-arabinofuranosyl-(1→5)-α-D-arabinofuranoside). Details of the NMR analysis is described in Methods.

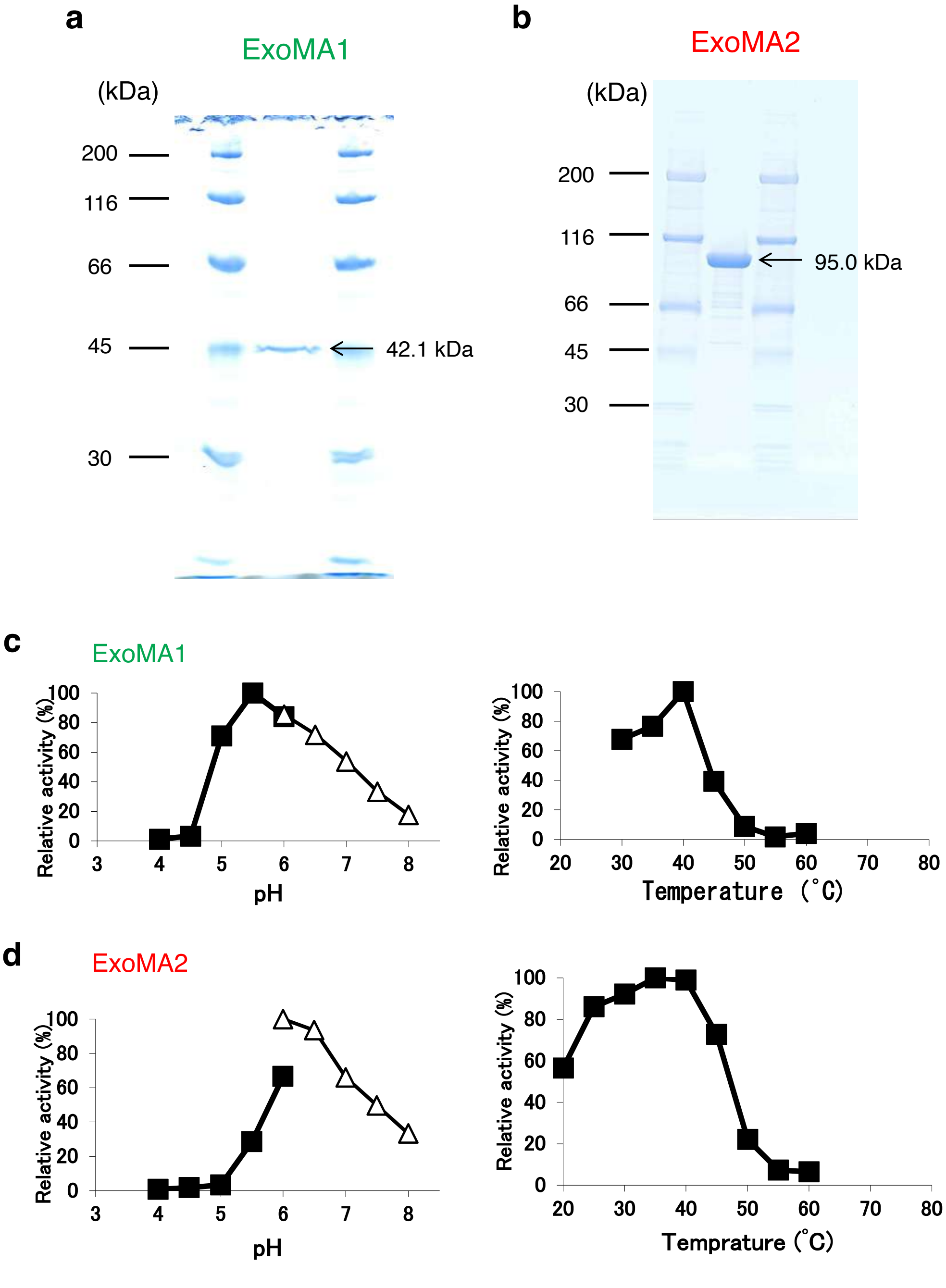


**Supplementary Fig. 9. Analysis of the purified samples of ExoMA1 and ExoMA2. a and b,** SDS-PAGE of ExoMA1 (**a**) and ExoMA2 (**b**). **c and d,** pH (left) and temperature (right) profiles of the activities of ExoMA1 (**c**) and ExoMA2 (**d**). Standard assay conditions for ExoMA1 and ExoMA2 were 1 mM *p*NP-α-D-Ara*f* in 50 mM sodium acetate (pH 5.5) and 4 mM *p*NP-β-D-Ara*f* in 50 mM sodium phosphate (pH 6.0), respectively. Data points measured in sodium acetate and sodium phosphate buffers are shown by closed squares and open triangles, respectively. Detailed assay conditions are described in Methods.

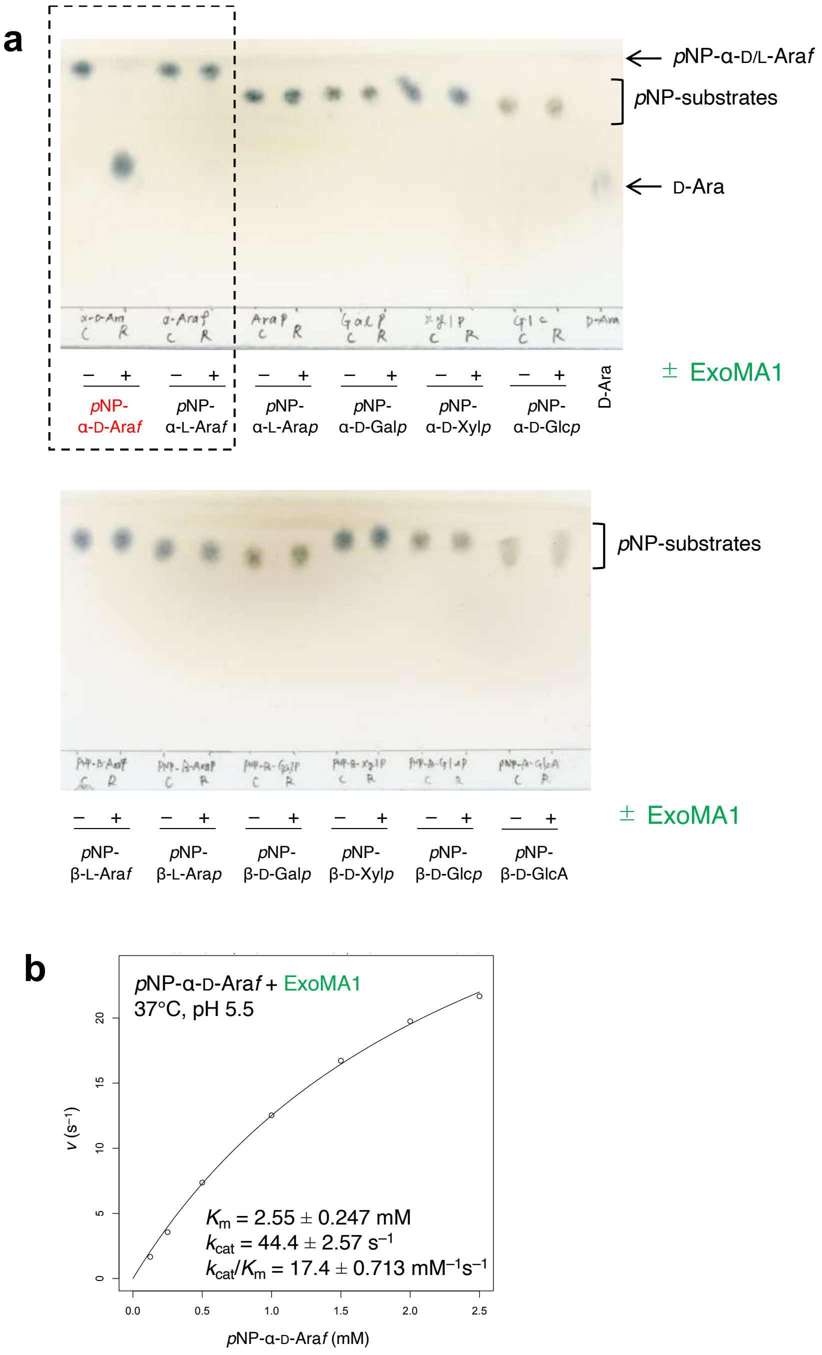


**Supplementary Fig. 10. Substrate specificity and kinetic analysis of ExoMA1. a,** TLC analysis using various *p*NP-substrates. Each *p*NP-substrate (1.25 mM) was incubated with or without ExoMA1 (1.0 μg/mL) in 50 mM sodium acetate (pH 5.5) at 37°C for 22 h. TLC plates were visualized by spraying the orcinol-sulfate reagent. **b,** *S*-*v* plot of the activity toward *p*NP-α-D-Ara*f*. The activity for 0.125-2.5 mM *p*NP-α-D-Ara*f* was measured in 50 mM sodium acetate (pH 5.5) at 37°C. The kinetic parameters calculated by curve fitting are shown.

**a**

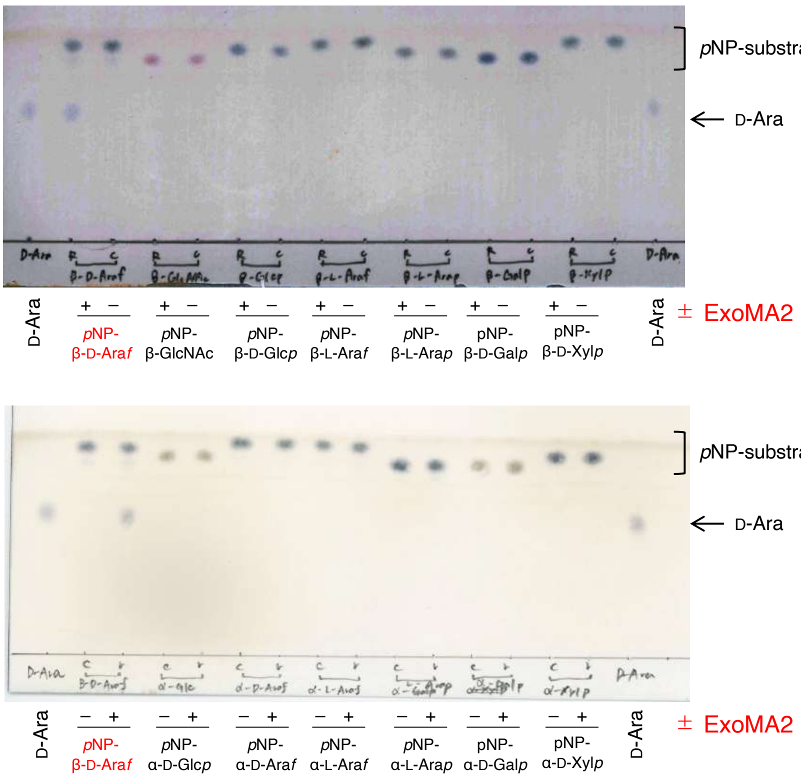


**b**

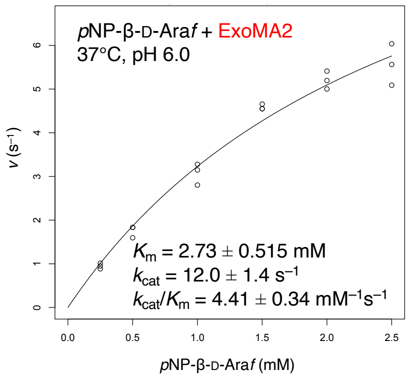


**Supplementary Fig. 11. Substrate specificity and kinetic analysis of ExoMA2. a,** TLC analysis using various *p*NP-substrates. Each *p*NP-substrate (1.25 mM) was incubated with or without ExoMA2 in 50 mM sodium phosphate (pH 6.0) at 37°C for 22 h. TLC plates were visualized by spraying diphenyl amine-aniline-phosphoric acid (upper panel) and orcinol-sulfate reagent (lower panel). **b,** *S*-*v* plot of the activity toward *p*NP-β-D-Ara*f*. The activity for 0.25-2.5 mM *p*NP-β-D-Ara*f* was measured in 50 mM sodium phosphate (pH 6.0) at 37°C. The kinetic parameters calculated by curve fitting are shown.

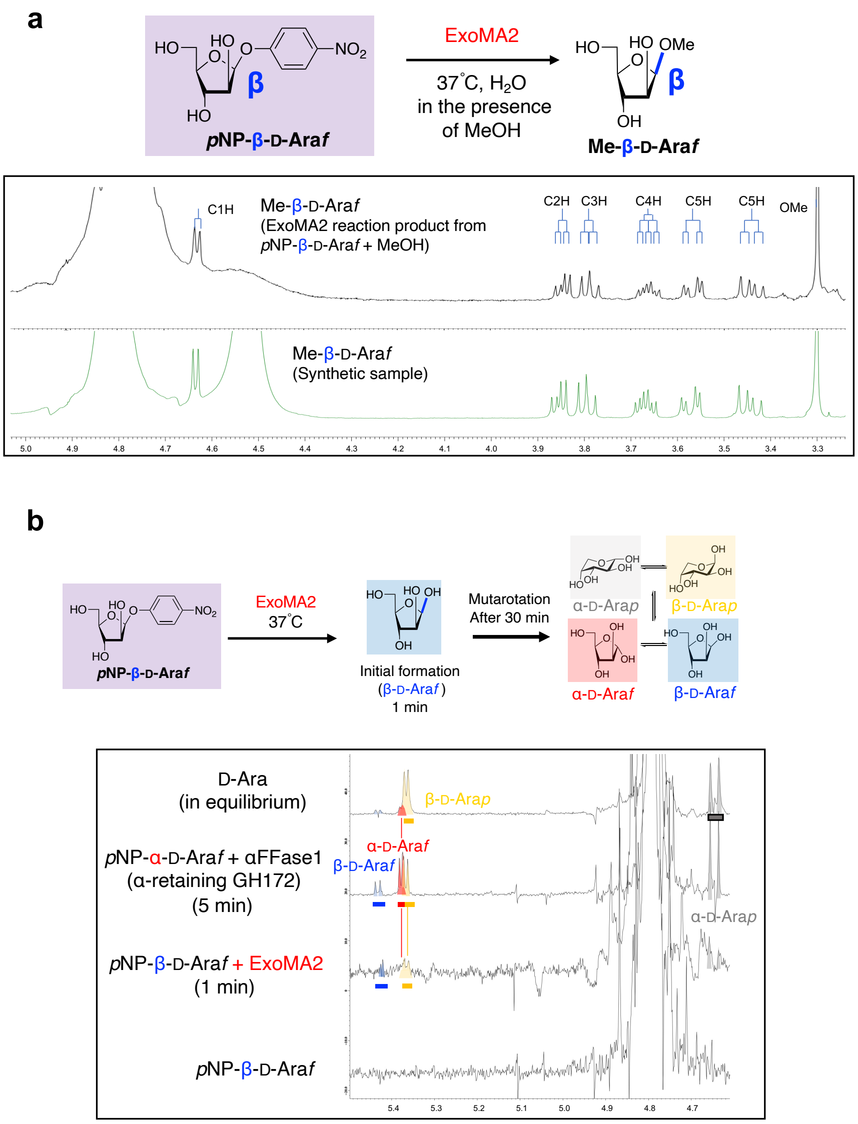


**Supplementary Fig. 12. NMR analysis of the anomer-retaining transglycosylation and hydrolysis reactions of ExoMA2. a,** Comparison of the $^{1}$H NMR spectra of the transglycosylation reaction product from *p*NP-β-D-Ara*f* in the presence of methanol and an authentic sample of Me-β-D-Ara*f*. **b,** $^{1}$H NMR analysis of the initial hydrolysis reaction product from *p*NP-β-D-Ara*f*. *p*NP-β-D-Ara*f* (20 mM) in 650 μL of phosphate buffer (pH 6.0) was treated with 8.2 μL of enzyme solution (531 μg/mL in $D_2O$) in an NMR tube, and the results were recorded on a 400 MHz NMR spectrometer. Spectra of the initial reaction product of GH172 αFFase 1 (anomer-retaining GH) from *p*NP-α-D-Ara*f*, equilibrated D-Ara, and *p*NP-β-D-Ara*f* are also shown. H-1 signal of arabinose: β-D-Ara*f* (doublet at 5.42 ppm), α-D-Ara*p* (doublet at 5.38 ppm, $^{3}J_{H1–H2}$ = 3.6 Hz), β-D-Ara*p* (doublet at 4.66 ppm, $^{3}J_{H1–H2}$ = 8.0 Hz), and α-D-Ara*f* (doublet at 5.39 ppm, $^{3}J_{H1–H2}$ = 2.8 Hz). The H-1 signal of α-D-Ara*f* has been reported previously[1].

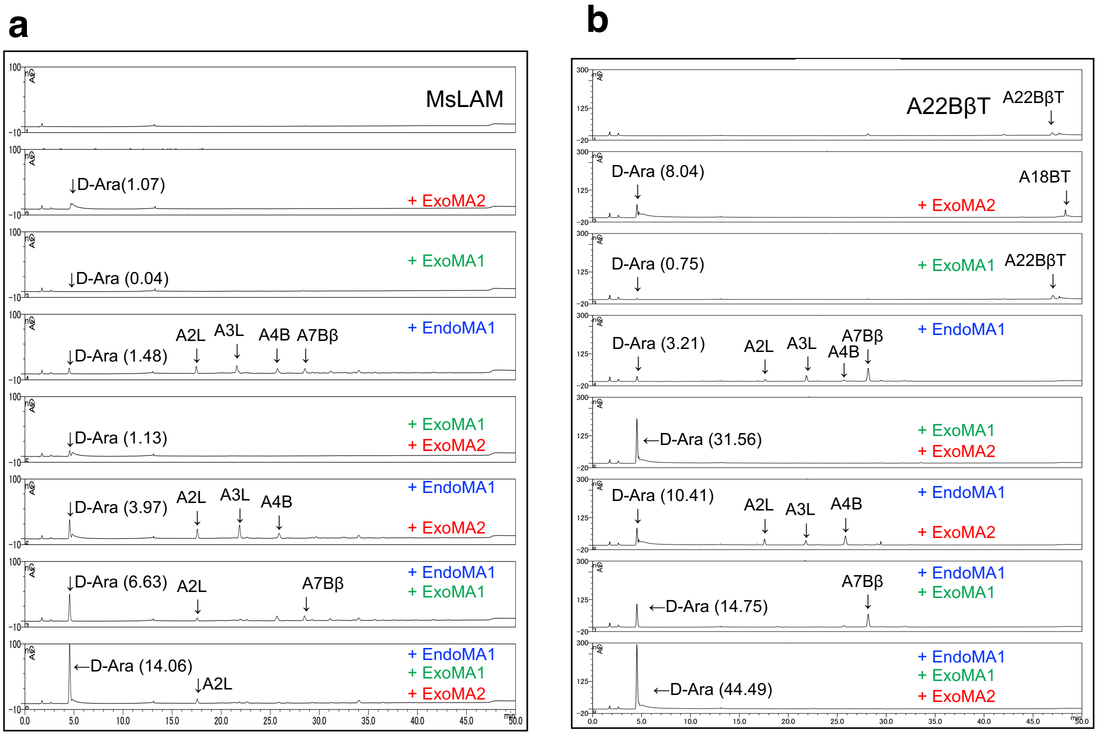


**Supplementary Fig. 13. Cooperative action of D-arabinan degrading enzymes. a and b,** HPAEC-PAD analysis of the combination reactions of EndoMA1, ExoMA1, and ExoMA2 with MsLAM (**a**) and A22BβT (**b**). MsLAM (0.25 mg/mL) or A22BβT (40 μg/mL) were incubated with enzymes (1.0 μg/mL each) in 50 mM sodium phosphate buffer (pH 6.0) at 37°C for 18 h. The reaction products were analyzed using HPAEC-PAD. Numbers in parentheses indicate the peak area of D-Ara.

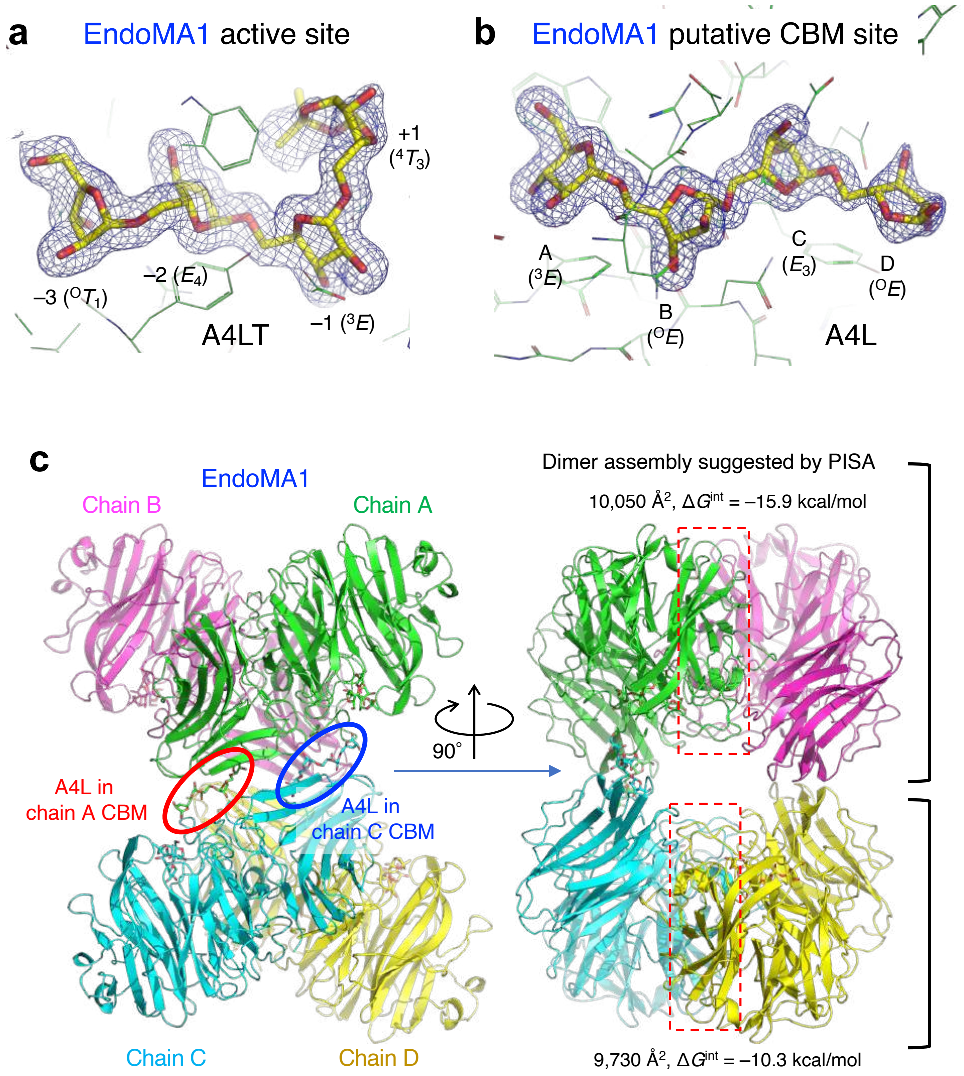


**Supplementary Fig. 14. Electron density maps of the bound oligo-D-arabinofuranosides and quaternary structure of EndoMA1 in the crystal structure. a and b,** Polder maps (3.0σ) of A4LT in the active site (**a**) and A4L in the D-arabinan binding site of the putative CBM domain (**b**). The furanose ring conformations of Ara*f* are shown in parentheses. **c,** Tetramer in the asymmetric unit viewed from two orientations. PISA analysis suggested that the assembly in the solution is a dimer. $\Delta G_{\mathrm{int}}$, solvation-free energy gain upon formation of the assembly at the interface indicated by the box with a red dotted line.

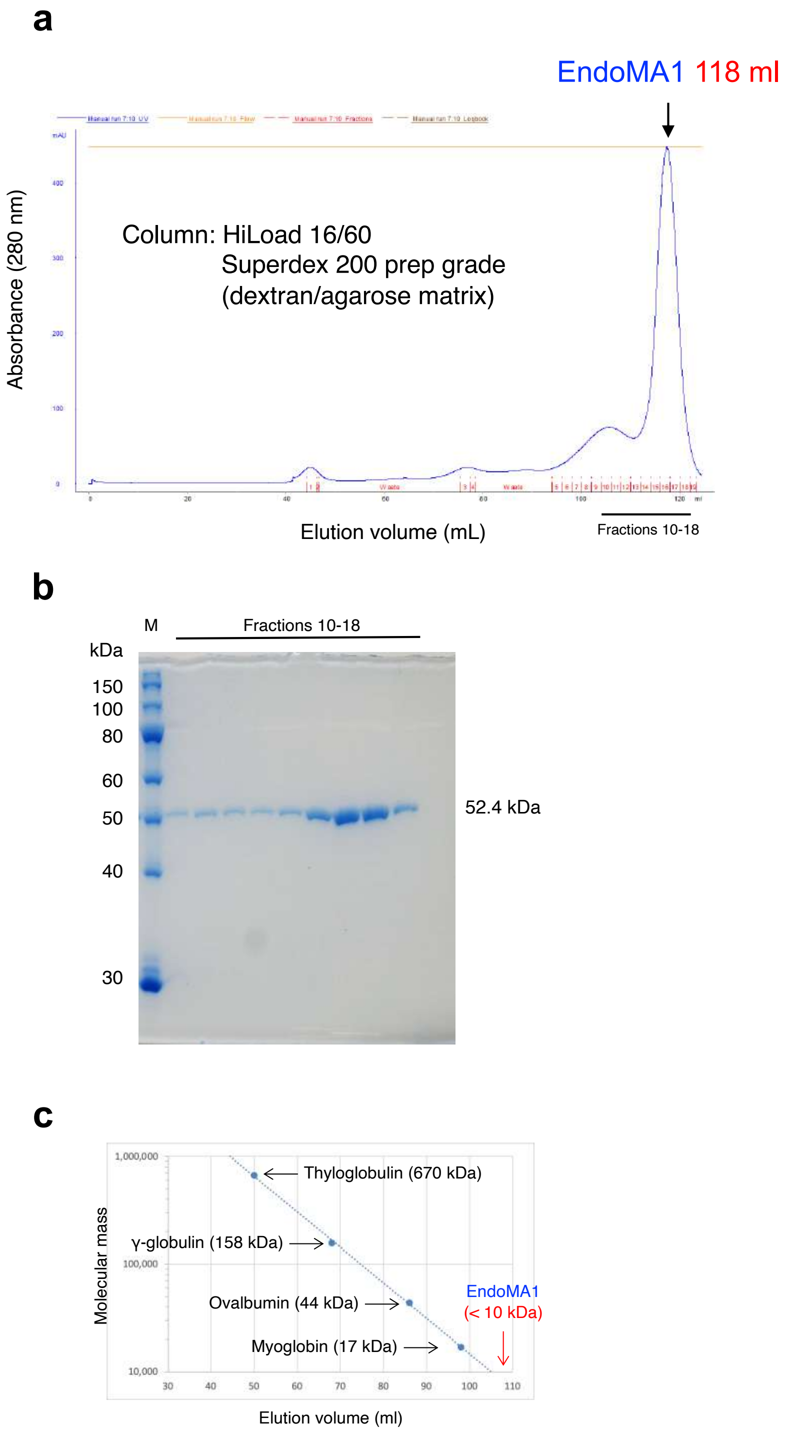


**Supplementary Fig. 15. Size exclusion chromatography and SDS-PAGE of EndoMA1. a,** Chromatogram of EndoMA1 using a Superdex size exclusion column. **b,** SDS-PAGE of the fractions. **c,** Molecular mass estimation using molecular marker proteins. EndoMA1 eluted at a position behind the expected molecular mass, probably due to interaction with the carrier.

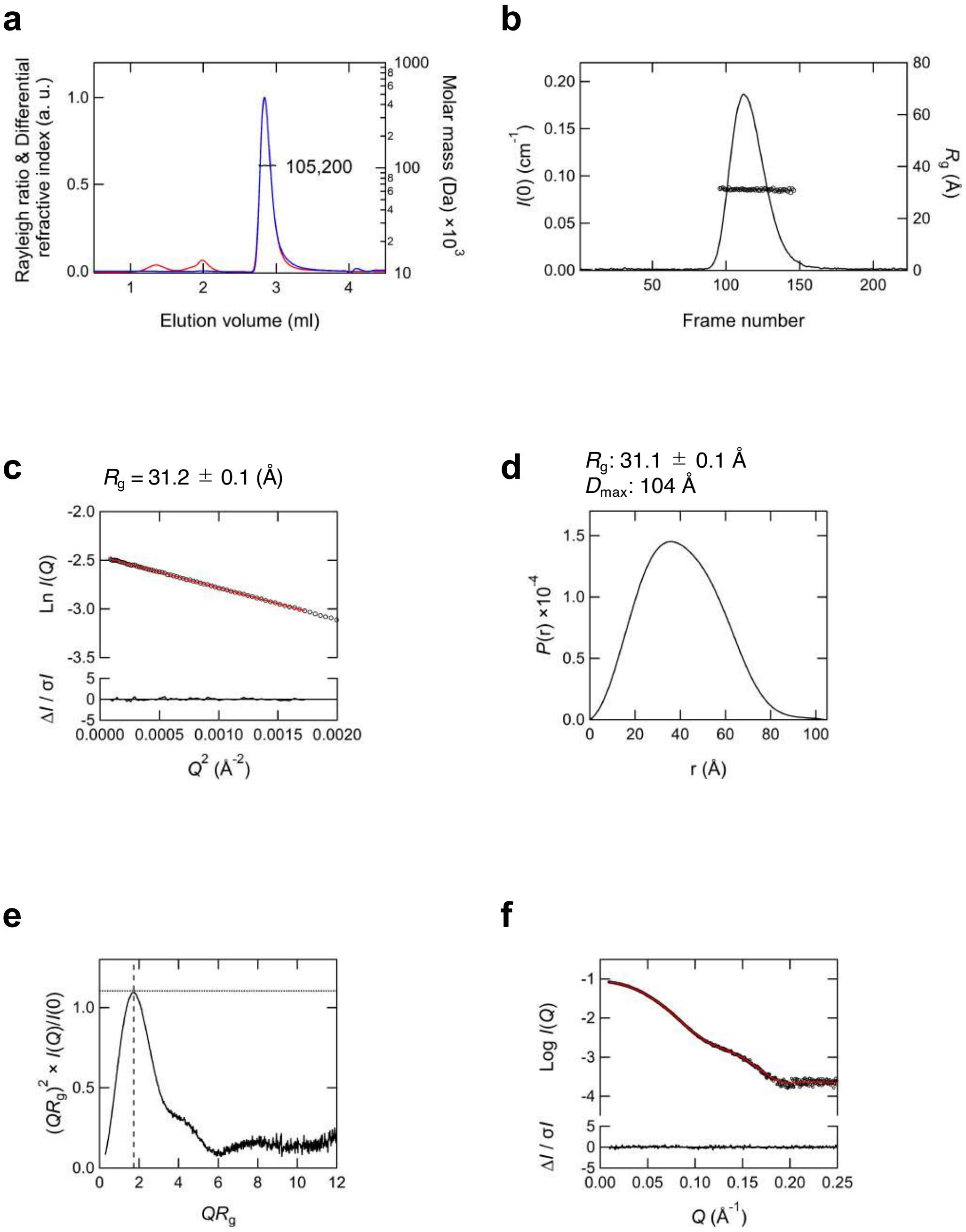


**Supplementary Fig. 16. Results of SEC-MALS/RI and SEC-SAXS measurements for EndoMA1. a,** Red, blue, and black lines show light scattering intensity, differential refractive index, and molar mass, respectively. The light scattering intensity and differential refractive index are normalized with the maximum peak value set at 1.0. **b,** Chromatogram of the forward scattering intensity $I(0)$ for all data (line) overlaid with the distribution of $R_g$ for frame numbers 96 to 146 (open circle). **c,** Guinier plot of the experimental SAXS profile displayed in Fig. 6d (black open circle). The red line represents the result of the linear approximation based on the Guinier analysis. The residuals in the linear approximation are drawn at the bottom of the graph.

**d,** Pair distance distribution function *P*(r). **e,** Normalized Kratky plot calculated using the values of $I(0)$ and $R_g$ obtained by the Guinier analysis. The dashed and the dotted lines show √3 and 3 $e^{-1}$, respectively. **f,** Superposition between the experimental SAXS profile (black) and the theoretical SAXS profile calculated from the bead model in Fig. 6e (red). The residuals of both profiles are drawn at the bottom of the graph.

**a**

**b**

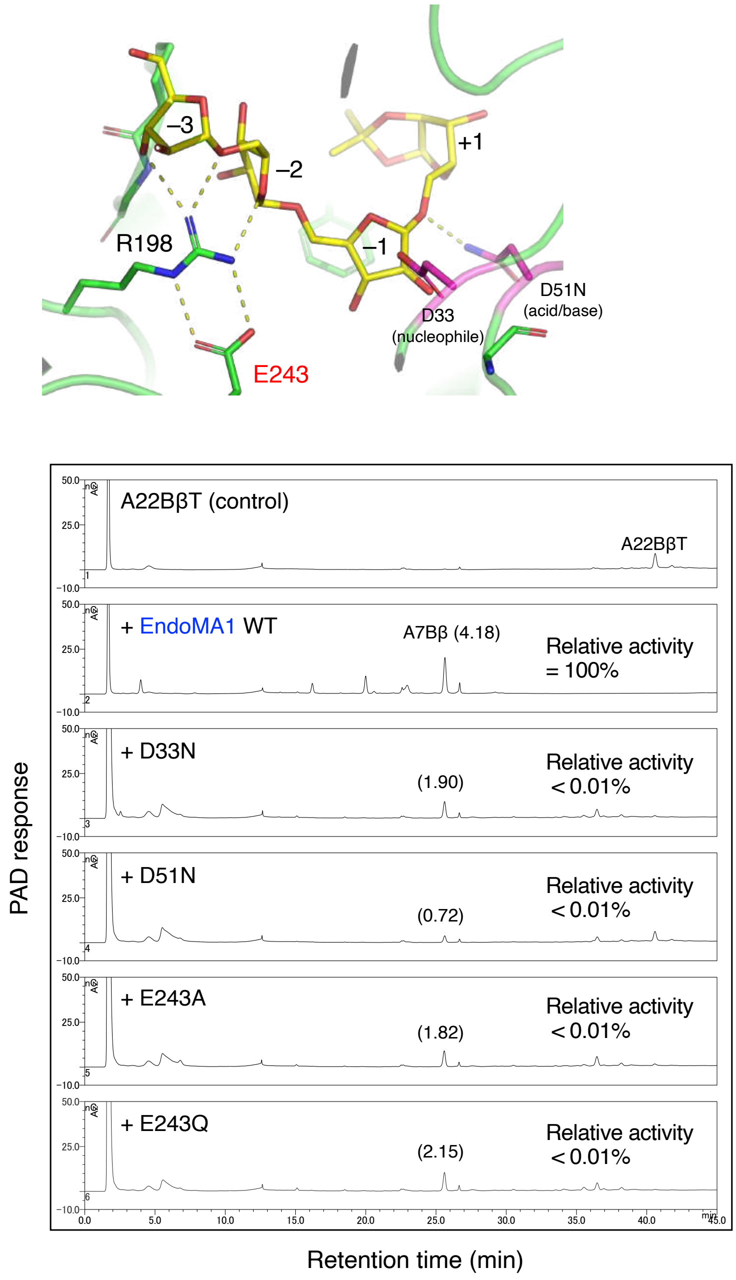


**Supplementary Fig. 17. Environment of Glu243 and EndoMA1 mutant activity. a,** Structure of the active site illustrating the interactions of Glu243. **b,** HPAEC-PAD analysis of the activity of active site mutants using A22BβT as a substrate. A22BβT (0.02 mg/mL) in 50 mM sodium phosphate (pH 6.5) was incubated at 37°C with WT EndoMA1 (4.0 μg/mL) or EndoMA1 mutants (8.0 mg/mL) for 18 h. Relative activities were calculated using the area value of A7Bβ with diluted WT enzyme incubated for 20 min. The numbers in parentheses indicate the peak areas of A7Bβ.

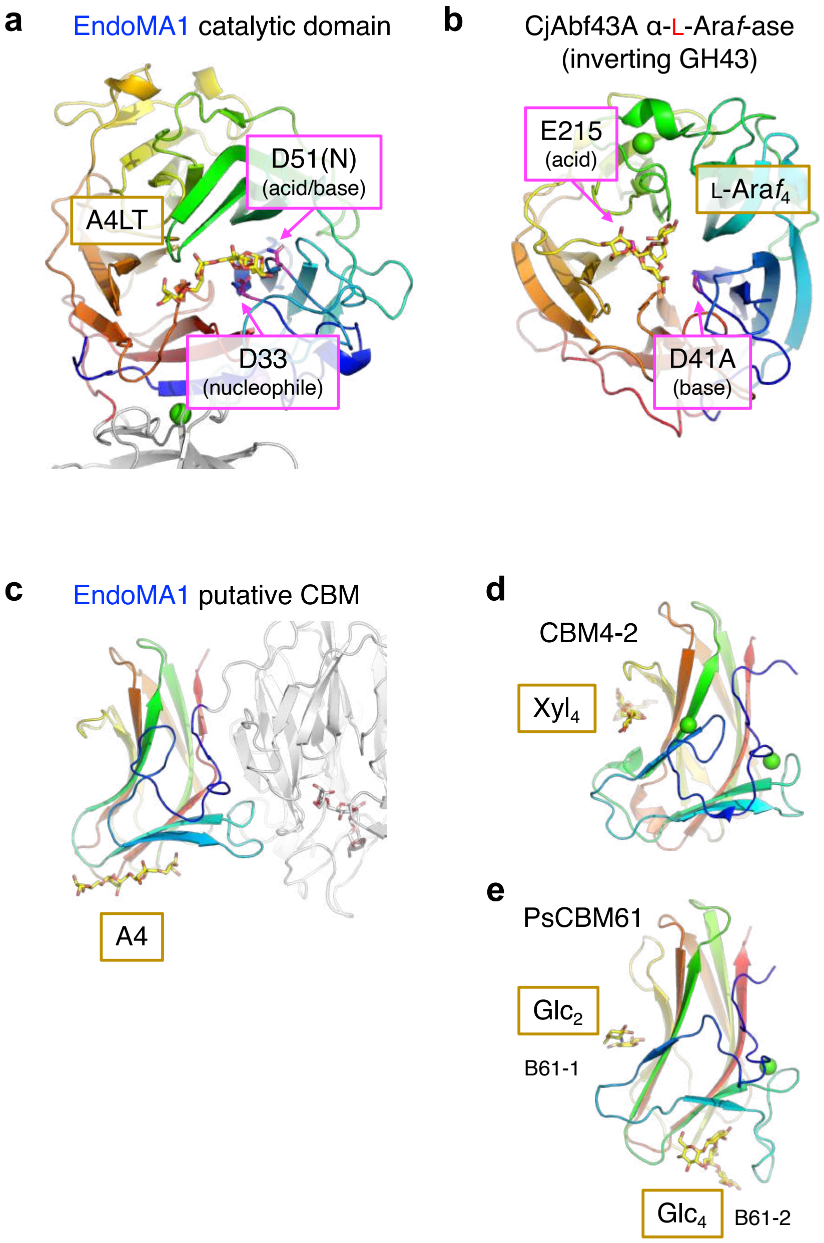


**Supplementary Fig. 18. Comparison of EndoMA1 with structural homologs. a,** The catalytic domain of EndoMA1 complexed with A4LT. **b,** GH43 α-L-Ara*f*-ase CjAbf43A from *Cellvibrio japonicus* (PDB ID: 3QEF) complexed with a tetrasaccharide (L-Ara$f_4$). **c,** Putative CBM domain of EndoMA1 complexed with A4. **d,** CBM4-2 domain in GH10 xylanase Xyn10A from *Rhodothermus marinus* (PDB ID: 2Y6L) complexed with xylotetrasaccharide ($Xyl_4$). **e,** CBM61 in GH31 α-1,6-glucosyltransferase Ps6TG31A from *Paenibacillus* sp. 598K (PDB ID: 5X7Q) complexed with isomaltose ($Glc_2$) and isomaltotetraose ($Glc_4$). A ribbon model with rainbow colors represents these structures.

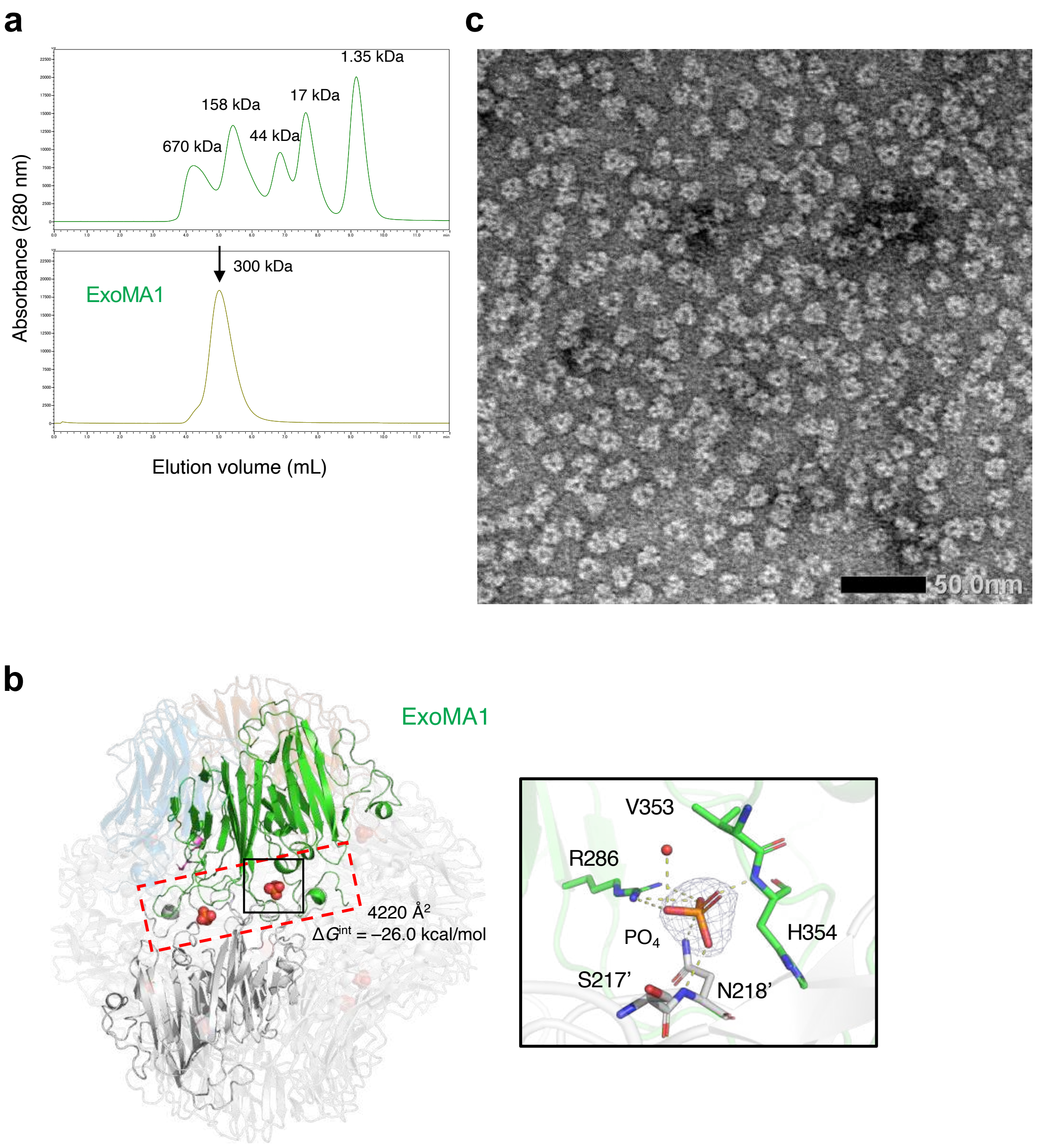


**Supplementary Fig. 19. Size exclusion chromatography, protomer interface, and electron microscopy of ExoMA1. a,** Size exclusion chromatography of molecular markers (upper panel) and ExoMA1 (lower panel). The molecular mass of the molecular markers and ExoMA1 (estimated) are indicated. **b,** Interface between two trimers in the dodecamer. One trimer is colored green, blue, and orange. One molecule of $PO_4$ per protomer is bound at the interface in focus. Details of the interaction between $PO_4$ and the protein are shown in the inset. $\Delta G_{int}$, solvation-free energy gain upon formation of the assembly at the interface indicated by the box with a red dotted line. **c,** Electron micrographs of negatively stained ExoMA1.

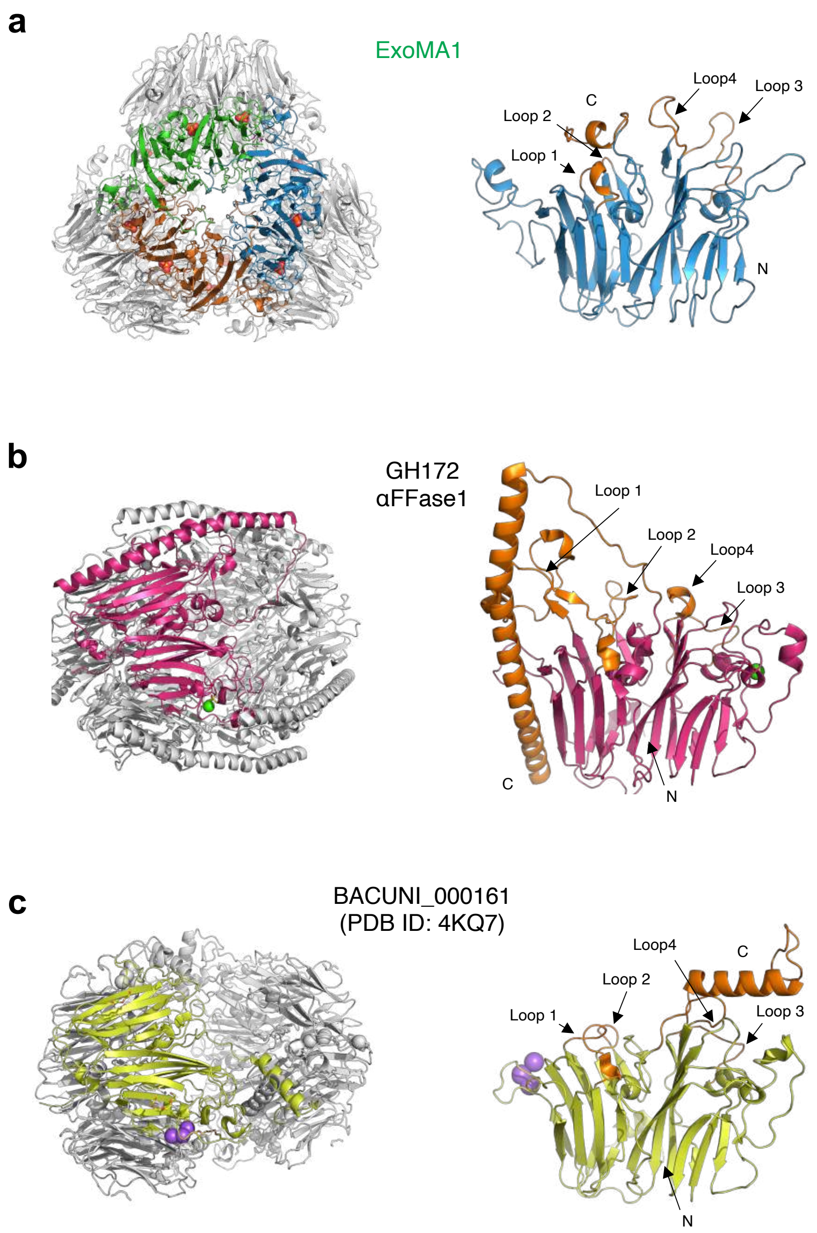


**Supplementary Fig. 20. Structural comparison of ExoMA1 with homolog enzymes.** Overall oligomer structure (left) and monomer structure (right) are shown. Structural elements involved in the oligomer assembly are shown in orange in the monomer structures, and four important loops are indicated by arrows (right). **a,** ExoMA1. The basal trimer of the dodecamer is colored green, blue, and orange (left). **b,** GH172 αFFase1 (PDB ID: 7V1V). One protomer in the hexamer is shown in magenta (left). Calcium ion is shown as a green sphere (right). **c,** DUF2961 family protein (BACUNI_00161) from *Bacteroides uniformis* (PDB ID: 4KQ7). One protomer in the hexamer is shown in yellow (left). Sodium ions are shown as purple spheres (right).

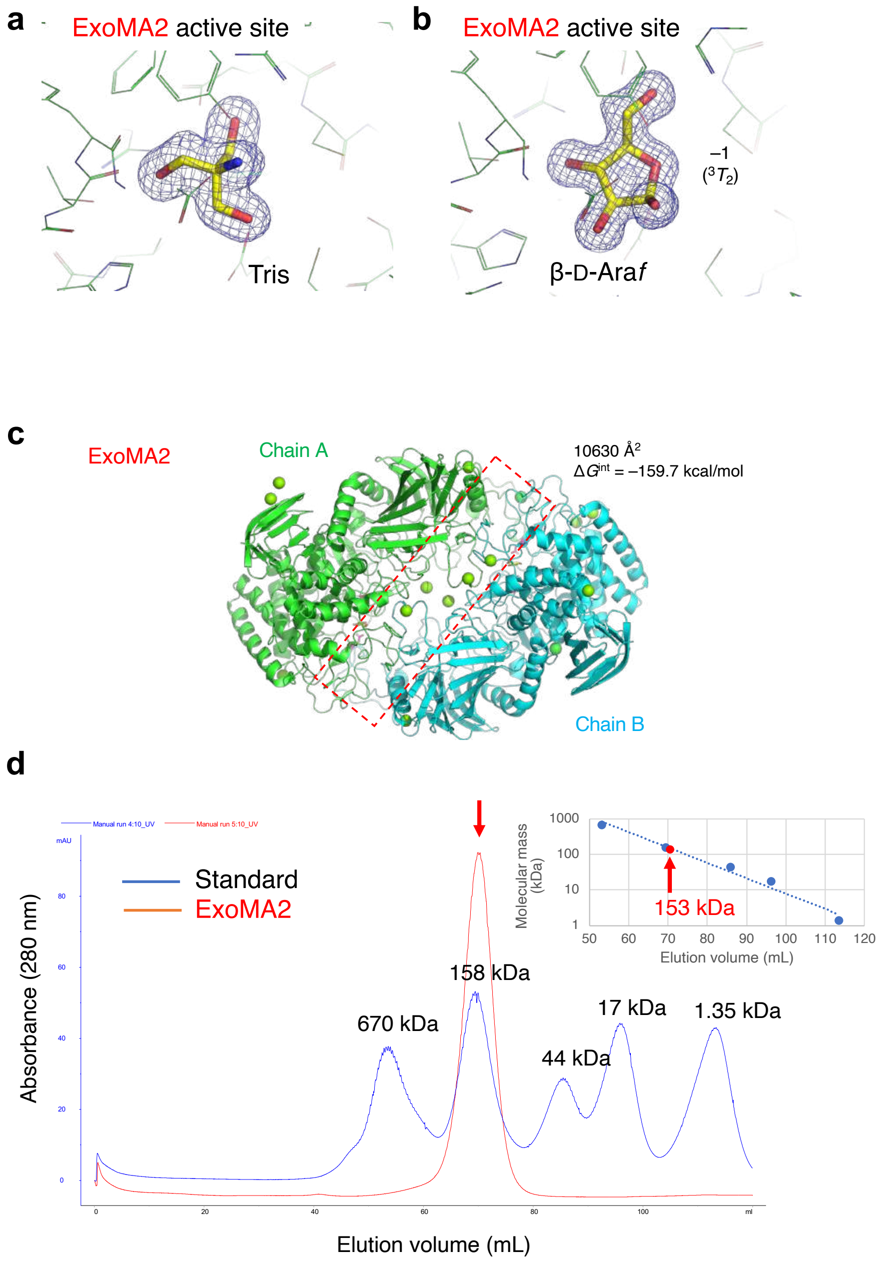


**Supplementary Fig. 21. Electron density maps, quaternary structure, and size exclusion chromatography of ExoMA2. A and b,** Polder maps (3.0σ) of Tris molecule (**a**) and β-D-Ara*f* (**a**) in the active site. The furanose ring conformation of β-D-Ara*f* is shown in parentheses. **C,** Dimer in the asymmetric unit viewed from two orientations. PISA analysis suggested that the assembly in the solution is a dimer. $\Delta G_{\mathrm{int}}$, solvation-free energy gain upon formation of the assembly at the interface indicated by the box with a red dotted line. **d,** Size exclusion chromatography of molecular markers (blue lines) and ExoMA2. Inset, molecular mass estimation of ExoMA2 (red) using molecular markers (blue).

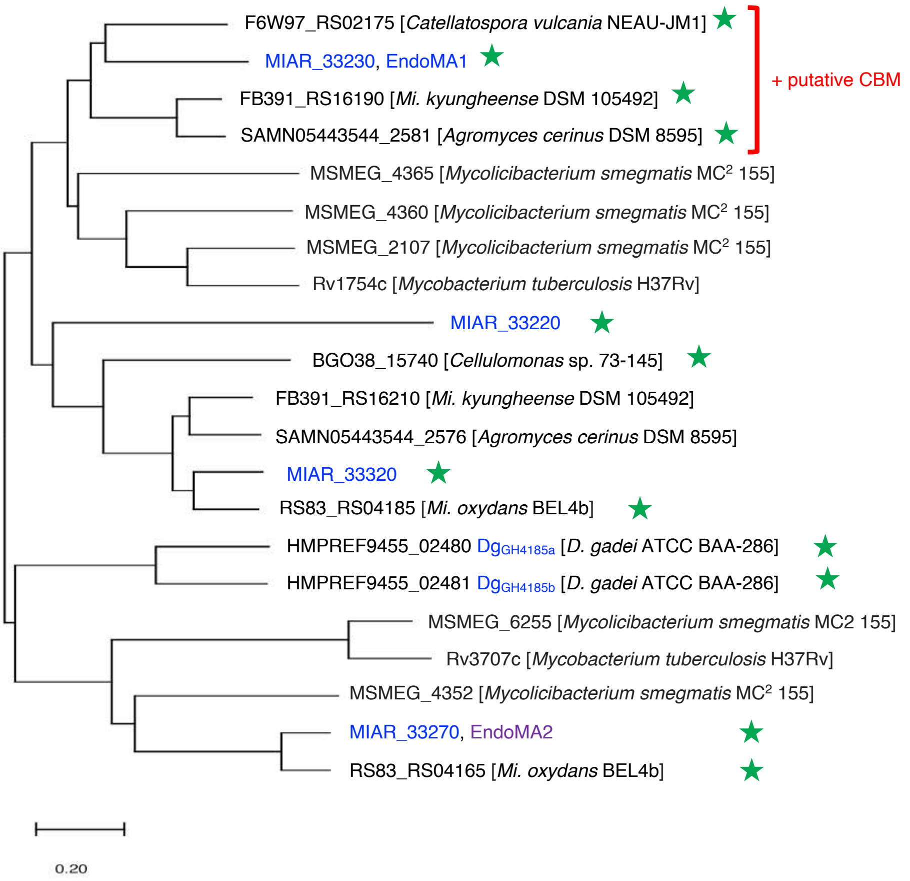


**Supplementary Fig. 22. Phylogenetic tree of EndoMA1, EndoMA2, and DUF4185 (GH183) proteins.** The bar indicates the branch length measured by the number of substitutions per amino acid site. The locus tags are shown alongside bacterial strains or protein names. The top four protein sequences, including EndoMA1, have a putative CBM domain, as analyzed by the InterProScan Search. Green star symbols indicate proteins encoded in the gene clusters in Fig. 9.

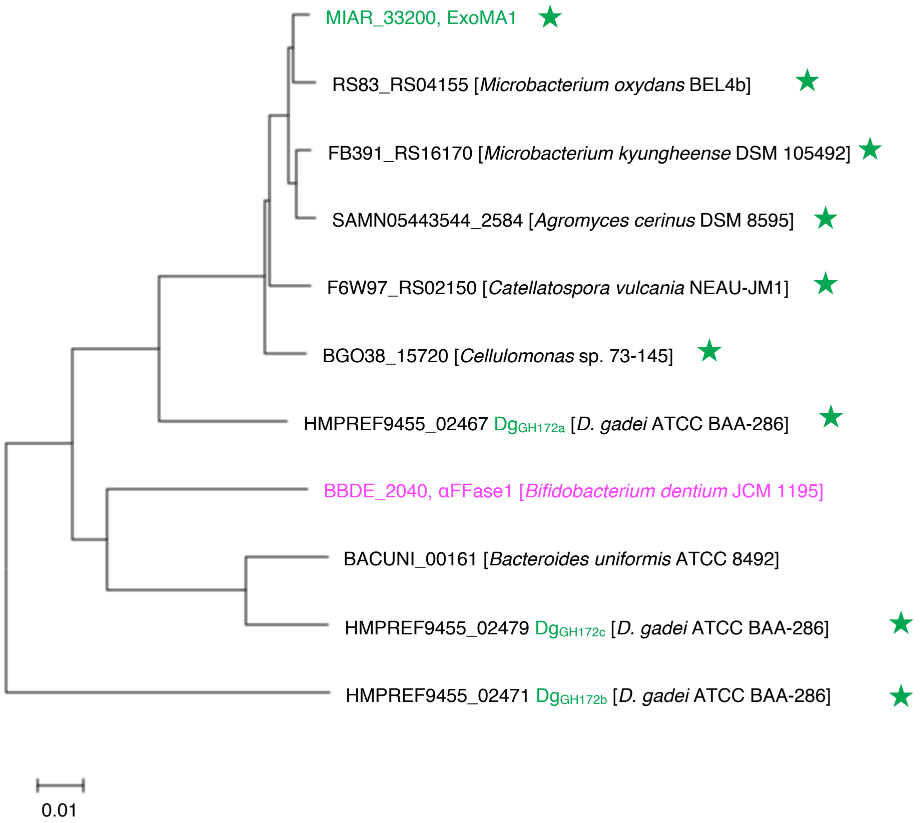


**Supplementary Fig. 23. Phylogenetic tree of ExoMA1 and GH172 proteins.** The bar indicates branch length measures in the number of substitutions per amino acid site. The locus tags are shown alongside bacterial strains or protein names. Proteins encoded in the gene clusters in Fig. 9 are indicated by green star symbols.

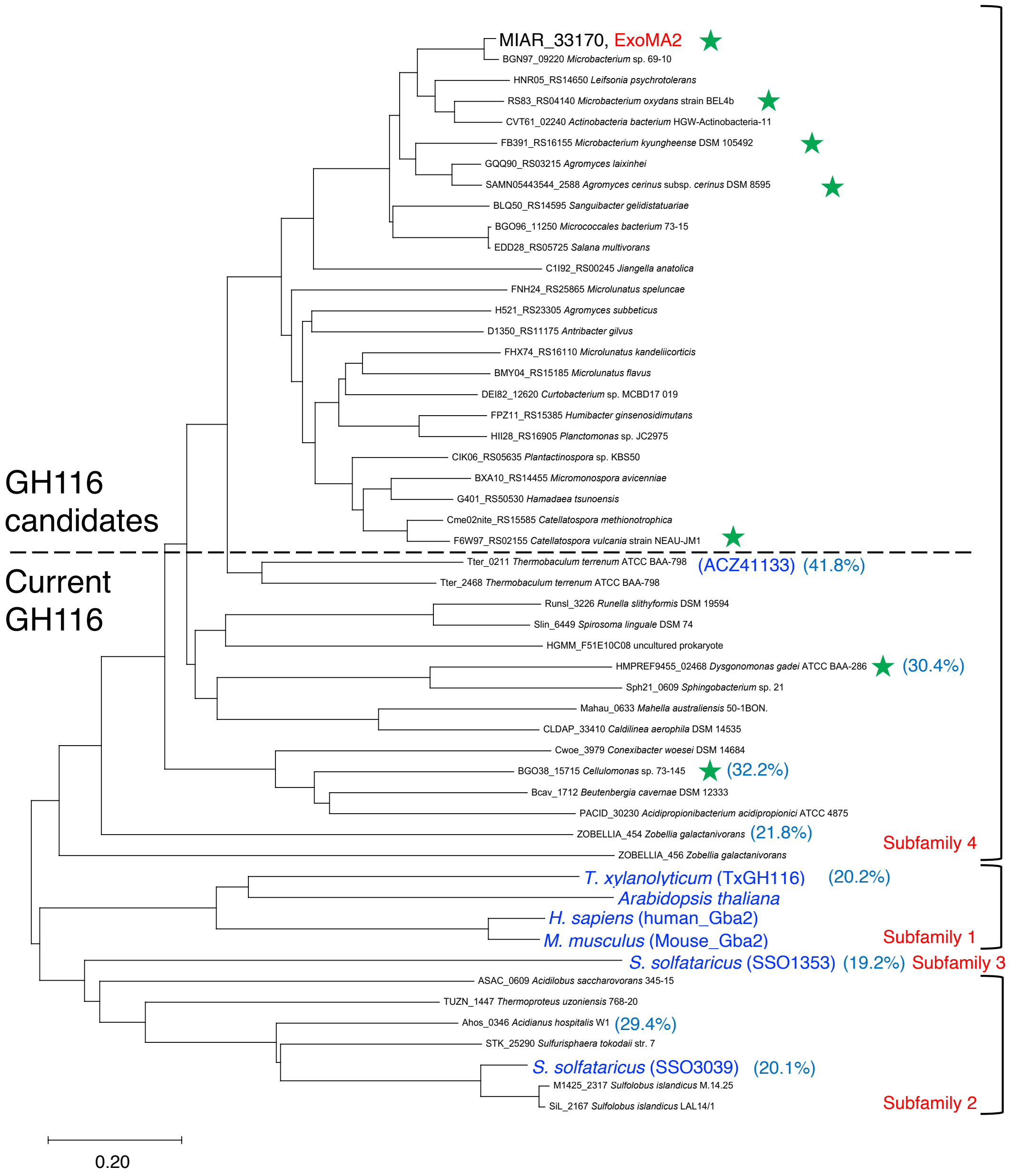


**Supplementary Fig. 24. Phylogenetic tree of ExoMA2 and homologs.** Proteins that are currently included in GH116 are below the dotted line. Amino acid sequence identities to ExoMA2 are shown in parentheses. Characterized GH116 enzymes are labeled with blue characters. The bar indicates branch length measures in the number of substitutions per amino acid site. The locus tags are shown alongside bacterial strains or protein names. Proteins encoded in the gene clusters in Fig. 9 are indicated by green star symbols.

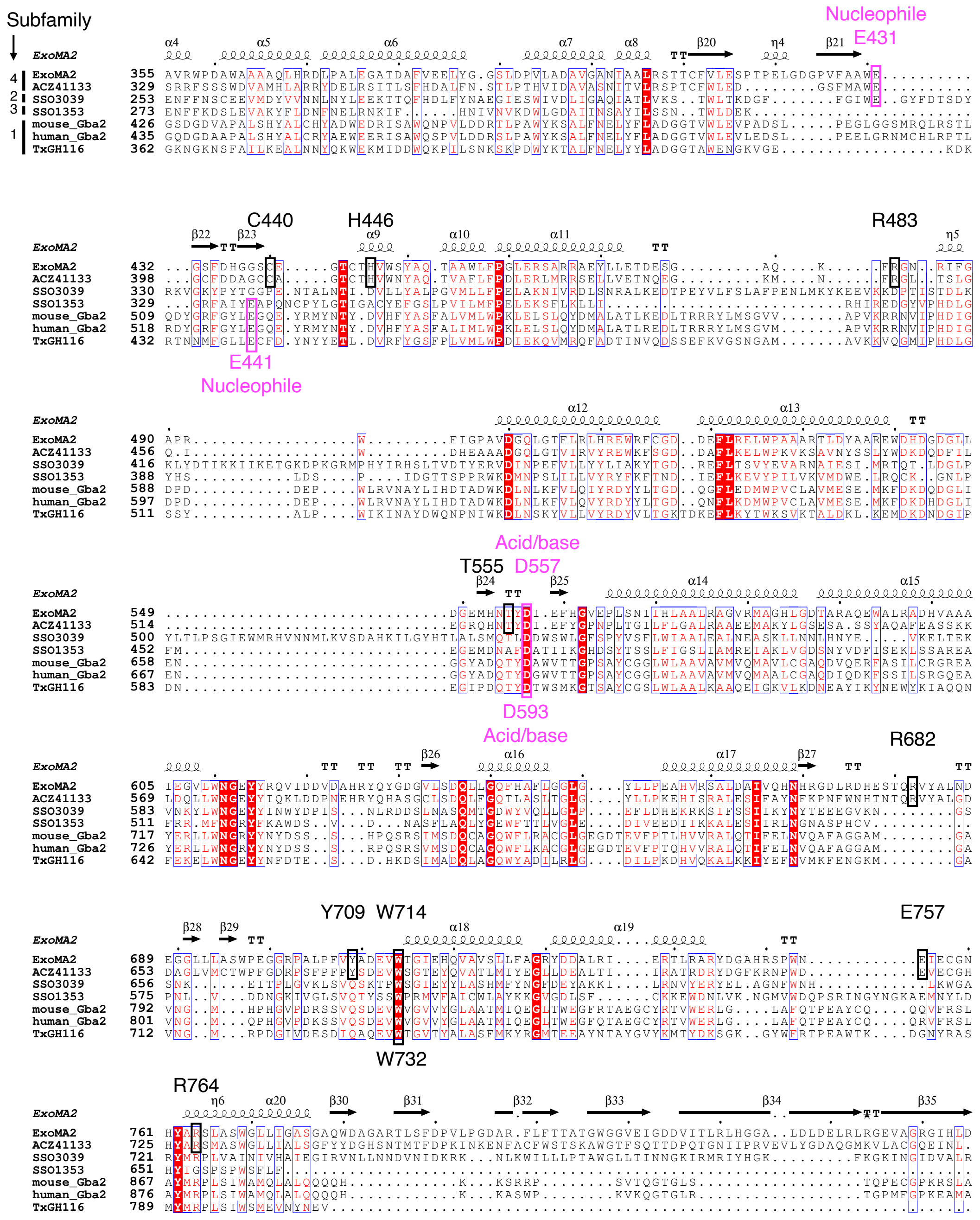

**Supplementary Fig. 25. Amino acid sequence alignment of ExoMA2 and GH116 proteins.** The catalytic residues and residues involved in the substrate recognition of β-D-Ara*f* are boxed with magenta and black lines, respectively. The amino acid residues for ExoMA2 and TxGH116 are shown above and below the box, respectively.

## Supplementary Tables

**Supplementary Table 1. General features of the draft genome of *Mi. arabinogalactanolyticum* JCM 9171**

| | |
|---|---|
| Genome size (bp) | 3,665,624 |
| GC content (%) | 69.8 |
| Number of contigs | 13 |
| Number of coding sequences | 3,458 |
| rRNA genes | 2 |
| tRNA genes | 53 |
| CRISPR | 0 |

The draft genome sequence has been submitted to DDBJ with accession numbers BRZC01000001-BRZC01000013.

**Supplementary Table 2. Previously reported NMR data of Me-α-A4B**

| in $D_2O$, r.t. | | $^{1}H$/ δ ppm | mult., $^{3}J_{H\text{–}H}$ Hz | $^{13}C$/ δ ppm |
|---|---|---|---|---|
| α-D-Ara*f*$^{D}$(1→5) | 1 | 5.09 | d, 1.6 | 108.22 |
| | 2 | 4.07 | dd, 3.3, 1.6 | 82.11 |
| | 3 | 3.86–3.90 | dd, 6.0, 3.3 | 77.45 |
| | 4 | 3.97 | ddd, 6.0, 5.7, 3.2 | 84.80 |
| | 5 | 3.76 | dd, 12.3, 3.2 | 61.99 |
| | | 3.65 | dd, 12.3, 5.7 | |
| α-D-Ara*f*$^{C}$(1→3) | 1 | **5.02** | d, 1.6 | 108.01 |
| | 2 | 4.06 | dd, 3.3, 1.6 | 81.82 |
| | 3 | 3.86–3.90 | dd, 5.8, 3.3 | 77.43 |
| | 4 | 4.04 | ddd, 5.8, 5.7, 3.2 | 84.86 |
| | 5 | 3.76 | dd, 12.3, 3.2 | 62.05 |
| | | 3.66 | dd, 12.3, 5.7 | |
| (→3,5)-α-D-Ara*f*$^{B}$(1→ | 1 | **5.04** | d, 1.0 | 108.33 |
| | 2 | 4.22 | dd, 2.0, 1.0 | 79.99 |
| | 3 | 4.02 | dd, 5.5, 2.0 | 83.53 |
| | 4 | 4.25 | ddd, 5.5, 5.4, 3.0 | 82.56 |
| | 5 | 3.86–3.90 | dd, 11.5, 5.4 | 67.31 |
| | | 3.78 | dd, 11.5 3.0 | |
| →5)-α-D-Ara*f*$^{A}$- | 1 | 4.85 | d, 1.8 | 109.27 |
| | 2 | 3.99 | d, 3.4, 1.8 | 81.53 |
| | 3 | 3.96 | d, 5.9, 3.4 | 77.31 |
| | 4 | 4.16 | ddd, 5.9, 5.4, 3.1 | 82.96 |
| | 5 | 3.88 | dd, 11.5, 5.4 | 67.31 |
| | | 3.72 | dd, 11.5, 3.1 | |
| OMe | | 3.35 | S | 55.9 |

NMR data of methyl α-D-arabinofuranosyl$^{D}$-(1→5)-[α-D-arabinofuranosyl$^{C}$-(1→3)]-α-D-arabinofuranosyl$^{B}$-(1→5)-α-D-arabinofuranoside$^{A}$ (Me-α-A4B in the main text and **SI-15** in **Supplementary Methods**) reported by D'Souza et al.[2] Our synthesis and analysis suggested that chemical shifts for some anomeric protons written in bold should be revised.

**Supplementary Table 3. Crystallographic data statistics of EndoMA1**

| Data set | EndoMA1 Se-Met | EndoMA1 apo | EndoMA1 D51N +A9LT |
|---|---|---|---|
| **Crystallization** | | | |
| Protein solution | 18.2 mg/mL EndoMA1 | 19.2 mg/mL EndoMA1 | 32 mg/mL EndoMA1, 10 mM A9LT |
| Reservoir solution | 0.4 M sodium thiocyanate, 23% PEG 3350 | 0.4 M sodium thiocyanate, 23% PEG 3350 | 0.12 M MES-NaOH (pH 6.0), 5% PEG3000, 20% PEG200 |
| Cryoprotectant | + 30% glycerol | + 30% glycerol | + 10% glycerol |
| **Data collection** [a] | | | |
| Beamline | PF BL-5A | PF BL-5A | PF AR-NE3A |
| Wavelength (Å) | 0.9791 | 1.0000 | 1.0000 |
| Space group | $P2_1$ | $P2_1$ | $P2_12_12_1$ |
| Unit cell (Å, °) | $a$ = 80.959, $b$ = 127.085, $c$ = 103.412, $\beta$ = 109.534 | $a$ = 81.285, $b$ = 128.296, $c$ = 104.019, $\beta$ = 109.378 | $a$ = 114.708, $b$ = 137.731, $c$ = 148.589 |
| Resolution (Å) | 49.20–1.78 (1.81–1.78) | 46.13–1.60 (1.63–1.60) | 46.61–1.80 (1.83–1.80) |
| Total reflections | 2,884,158 (68,698) | 985,559 (47,737) | 1,456,432 (67,939) |
| Unique reflections | 172,300 (4,881) | 261,209 (12,725) | 217,228 (10,677) |
| $R_{merge}$ | 0.195 (1.372) | 0.067 (0.485) | 0.114 (1.235) |
| $R_{pim}$ | 0.062 (0.516) | 0.041 (0.289) | 0.047 (0.528) |
| Mean $I/\sigma(I)$ | 10.4 (1.9) | 9.9 (1.9) | 12.0 (2.0) |
| $CC_{1/2}$ | 0.995 (0.773) | 0.989 (0.844) | 0.996 (0.625) |
| Completeness (%) | 91.6 (52.5) | 99.0 (98.0) | 100.0 (99.9) |
| Multiplicity | 16.7 (14.1) | 3.8 (3.8) | 6.7 (6.4) |
| Anomalous completeness (%) | 91.1 (50.7) | | |
| Anomalous multiplicity | 8.2 (7.0) | | |
| Mol/ASU [b] | 4 | 4 | 4 |
| **Refinement** | | | |
| Resolution (Å) | | 46.18–1.60 | 46.65–1.80 |
| No. of reflections | | 261,169 | 217,104 |
| $R_{work}/R_{free}$ [c] | | 0.177/0.211 | 0.157/0.192 |
| Number of atoms | | | |
| Amino acids | | 14,847 | 14,872 |
| Ions | | 12 | 8 |
| Ligands | | 96 | 416 |
| Waters | | 1,760 | 1,314 |
| B-factors (Å$^2$) | | | |
| Amino acids | | 19.3 | 23.51 |
| Ions | | 14.5 | 22.17 |
| Ligands | | 26.5 | 34.99 |
| Waters | | 28.01 | 34.21 |
| RMSD from ideal values | | | |
| Bond lengths (Å) | | 0.0157 | 0.0213 |
| Bond angles (°) | | 2.01 | 2.02 |
| Ramachandran plot (%) | | | |
| Favored | | 95.28 | 95.49 |
| Allowed | | 4.25 | 3.88 |
| Outlier | | 0.47 | 0.63 |
| PDB code | | 8HHV | 8IC1 |

[a]Values in parentheses represent the highest resolution shell. [b]Number of molecules per asymmetric unit. [c]$R_{free}$ was calculated for a randomly chosen 5% of reflections that were not used for structure refinement, and $R_{work}$ was calculated for the remaining reflections.

**Supplementary Table 4. Dali structural similarity search of the two domains of EndoMA1**

| PDB ID[a] | Z score | RMSD (Å) | LALI[b] | Identity (%) | Protein name | Organism | Reference |
|---|---|---|---|---|---|---|---|
| **N-terminal catalytic domain (2-235)** | | | | | | | |
| 4HBS (A) | 23.8 | 3.3 | 286 | 15 | Putative hydrolase (BACOVA_04882) | *Bacteroides ovatus* ATCC 8483 | – |
| 6N1A (A) | 22.8 | 2.8 | 253 | 11 | *N*-Acetylgalactosamine deacetylase | *Flavonifractor plautii* | 3 |
| 4NOV (A) | 22.3 | 3.1 | 249 | 10 | Xylosidase/arabino-furanosidase Xsa43E (GH43) | *Butyrivibrio proteoclasticus* B316 | 4 |
| 3QEF (B) | 22.0 | 2.9 | 246 | 14 | α-1,2-arabinofuranosidase CjAbf43A (GH43) | *Cellvibrio japonicus* Ueda107 | 5 |
| **C-terminal putative CBM domain (336-480)** | | | | | | | |
| 2Y6L (A) | 16.8 | 2.2 | 139 | 16 | CBM4-2 in GH10 xylanase Xyn10A | *Rhodothermus marinus* | 6 |
| 5X7Q (A) | 16.6 | 2.1 | 132 | 24 | PsCBM61 (1128-1281) in GH31 α-1,6-glucosyltransferase Ps6TG31A | *Paenibacillus* sp. 598K | 7 |
| 2ZXQ (A) | 16.3 | 2.1 | 129 | 18 | Domain 5 (1102-1255) in GH101 endo-α-*N*-acetylgalactosaminidase | *Bifidobacterium longum* | 8 |

[a]Chain ID is shown in parentheses. [b]Number of aligned residues.

**Supplementary Table 5. Crystallographic data statistics of ExoMA1 and ExoMA2**

| Data set | ExoMA1 apo | ExoMA2 +Tris | ExoMA2 +β-D-Ara*f* |
|---|---|---|---|
| **Crystallization** | | | |
| Protein solution | 10 mg/mL ExoMA1 | 7.5 mg/mL ExoMA2 + 20 mM D-Ara | 7.5 mg/mL ExoMA2 + 200 mM D-Ara |
| Reservoir solution | 0.2 M NaCl, 0.1 M phosphate citrate, 20% PEG8000 | 0.18 M $MgCl_2$, 0.1 M Tris-HCl (pH 9.0), 14% PEG8000 | 0.2 M $MgCl_2$, 0.1 M CHES-NaOH (pH 8.9), 14% PEG8000 |
| Cryoprotectant | + 20% trehalose | + 20% MPD | + 20% MPD |
| **Data collection** [a] | | | |
| Beamline | PF AR-NW12A | PF BL-5A | PF AR-NW12A |
| Wavelength (Å) | 1.0000 | 1.0000 | 1.0000 |
| Space group | $P2_12_12_1$ | $P2_1$ | $P2_1$ |
| Unit cell (Å, °) | $a$ = 131.11, $b$ = 207.54, $c$ = 253.88 | $a$ = 66.552, $b$ = 97.511, $c$ = 139.978, $\beta$ = 100.549 | $a$ = 66.763, $b$ = 97.249, $c$ = 139.681, $\beta$ = 101.250 |
| Resolution (Å) | 48.24–2.42 (2.46–2.42) | 48.76–1.75 (1.78–1.75) | 48.62–1.35 (1.37–1.35) |
| Total reflections | 2,008,067 (98,598) | 601,258 (27,247) | 1,287,075 (61,133) |
| Unique reflections | 264,416 (12,946) | 176,777 (8,703) | 382,283 (18,879) |
| $R_{merge}$ | 0.147 (0.616) | 0.052 (0.366) | 0.056 (0.965) |
| $R_{pim}$ | 0.057 (0.239) | 0.043 (0.308) | 0.036 (0.629) |
| Mean $I/\sigma(I)$ | 9.9 (2.8) | 14.8 (2.9) | 9.5 (1.0) |
| $CC_{1/2}$ | 1.00 (0.85) | 0.998 (0.873) | 0.988 (0.609) |
| Completeness (%) | 100 (100) | 100.0 (99.9) | 99.9 (99.7) |
| Multiplicity | 7.6 (7.6) | 3.4 (3.1) | 3.4 (3.2) |
| Mol/ASU [b] | 12 | 2 | 2 |
| **Refinement** | | | |
| Resolution (Å) | 48.92-2.42 | 48.80–1.75 | 45.87–1.35 |
| No. of reflections | 264,283 | 176,748 | 382,249 |
| $R_{work}/R_{free}$ [c] | 0.199/0.229 | 0.138/0.174 | 0.159/0.183 |
| Number of atoms | | | |
| Amino acids | 34,813 | 13,274 | 13,234 |
| Ions | 0 | 16 | 18 |
| Ligands | 60 | 55 | 66 |
| Waters | 1,027 | 1,661 | 1,630 |
| B-factors (Å$^2$) | | | |
| Amino acids | 62.82 | 21.91 | 19.37 |
| Ions | – | 27.11 | 24.43 |
| Ligands | 65.79 | 29.17 | 28.7 |
| Waters | 57.31 | 29.28 | 28.39 |
| RMSD from ideal values | | | |
| Bond lengths (Å) | 0.0088 | 0.0147 | 0.0129 |
| Bond angles (°) | 1.267 | 1.91 | 1.69 |
| Ramachandran plot (%) | | | |
| Favored | 94.01 | 96.13 | 96.07 |
| Allowed | 5.35 | 3.58 | 3.58 |
| Outlier | 0.63 | 0.29 | 0.35 |
| PDB code | 8IC8 | 8IC6 | 8IC7 |

[a]Values in parentheses represent the highest resolution shell. [b]Number of molecules per asymmetric unit. [c]$R_{free}$ was calculated for a randomly chosen 5% of reflections that were not used for structure refinement, and $R_{work}$ was calculated for the remaining reflections.

## Supplementary Methods

### Details of protein production for crystallography

Recombinant EndoMA1 (WT and D51N mutant), ExoMA1, and ExoMA2 proteins were expressed in *E. coli* BL21 CodonPlus RIL (DE3) cultured in lysogeny broth (LB) medium. SeMet-labeled EndoMA1 protein was expressed in *E. coli* B834 (DE3), and the cells were cultured in 23.5 g/L SeMet core medium (FUJIFILM Wako Pure Chemical Corp, Osaka, Japan) supplemented with 25 mg/L Se-Met, 10 g/L glucose, 250 mg/L $MgSO_4 \cdot 7H_2O$, 4.2 mg/L $FeSO_4 \cdot 7H_2O$, and 10 mL/L Kao & Michayluk Vitamin Solution (Sigma-Aldrich Co., St. Louis, MO, USA). After cultivation in 100 mg/L ampicillin at 37°C, 0.1 mM (ExoMA1 and ExoMA2) or 0.3 mM (EndoMA1), isopropyl-β-D-thiogalactopyranoside was added until the absorbance at 600 nm reached 0.3-0.6. The cultures were incubated for 16-20 h at 37°C (unlabeled EndoMA1 and ExoMA1) or 25°C (SeMet-labeled EndoMA1 and unlabeled ExoMA2). Cells were harvested by centrifugation and suspended in 50 mM Tris-HCl (pH 7.5) for EndoMA1 and ExoMA1) or in 50 mM MES-NaOH (pH 6.5) and 350 mM NaCl for ExoMA2. The cells were disrupted by sonication on ice, and the supernatant was filtered using Minisart Syringe filter 0.45 um (Sartorius Stedim Biotech, Göttingen, Germany). The crude extract was purified using immobilized metal affinity, anion exchange, and size-exclusion column chromatography. EndoMA1 and ExoMA1 were purified using columns of cOmplete His-tag Purification Resin (Roche Diagnostics GmbH, Mannheim, Germany), Hiload 16/60 Superdex 200 prep grade, and Mono Q 10/100 GL (Cytiva, Malborough, MA, USA), respectively, whereas ExoMA2 was purified using His-tag, Mono Q, and Superdex columns, respectively. For EndoMA1 and ExoMA1, immobilized metal affinity chromatography was performed in 50 mM Tris-HCl (pH 7.5), 5 mM (wash), or 500 mM (elution) imidazole; size exclusion chromatography was performed in 50 mM Tris-HCl (pH 7.5) and 200 mM NaCl; and anion exchange chromatography was performed in 50 mM Tris-HCl (pH 7.5) and 0-1 M NaCl. For ExoMA2, immobilized metal affinity chromatography was performed in 50 mM MES-NaOH (pH 6.5) and 25 mM (wash) or 500 mM (elution) imidazole; anion exchange chromatography was performed in 25 mM MES-NaOH (pH 6.5) and 0-500 mM NaCl; and size exclusion chromatography was performed in 25 mM MES-NaOH (pH 6.5) and 350 mM NaCl. The purified proteins were concentrated using an Amicon Ultracel-30K centrifugal filter (Millipore, Billerica, MA, USA).

### Software and servers for protein crystallography

Crystallographic datasets were processed using XDS[9] and Aimless software[10]. SeMet phase determination and automated model building of the EndoMA1 crystal structure were performed using PHENIX software[11]. Molecular replacement was performed using MoRDA software[12].

Manual model building was performed using Coot[13]. Crystallographic refinement was performed using Refmac[14] or PHENIX[11]. Polder maps[15] were created using PHENIX software. Molecular graphic images were prepared using PyMOL (Shrödinger LLC, New York, NY, USA). Molecular interface analysis was performed using PISA server[16]. A structural similarity search was performed using the Dali server[17].

**Details of SEC-MALS/RI and SEC-SAXS experiments and analysis**

SEC-MALS/RI was performed using DAWN HELEOS II (Wyatt Technology) and an HPLC system (Alliance 2695, Waters)[18]. The sample concentration required to calculate the absolute molar mass was derived from the differential refractive index using a 2414 online differential refractometer (Waters). A KW403-4F column (Shodex) equilibrated in a buffer containing 20 mM Tris-HCl (pH 7.5) and 200 mM NaCl, was used as the SEC column. The sample was dissolved in the column buffer and concentrated to 2.406 mg/mL for the SEC-SAXS experiments. The flow rate of the HPLC pump was 0.2 mL/min, and 0.03 ml of sample was injected into the column. The measured data were analyzed using the ASTRA 6.1 software (Wyatt Technology).

The SEC-SAXS experiment was carried out at the beamline BL-10C of the Photon Factory (Tsukuba, Japan)[19]. The X-ray wavelength was 1.000 Å, and the camera length was 2081.4 mm. SEC was performed using an HPLC system, Prominence-i (SHIMADZU), with the same KW403-4F column (Shodex) equilibrated with a buffer of 20 mM Tris-HCl, 200 mM NaCl, pH 7.5 as for SEC-MALS. The column temperature was maintained at 293 K. The sample was also identical to that of SEC-MALS (2.406 mg/ml), and a volume of 0.13 ml was injected into the column. The HPLC pump flow rate was set to 0.2 ml min-1 from the start to 2.5 ml and 0.02 mL/min from 2.5 ml to adapt to the peak measurement, referring to the chromatogram measured in SEC-MALS. The SAXS sample cell was composed of stainless steel, and the X-ray irradiated area covered with 0.02 mm-thickness quartz glasses was 3.0 mm horizontal × 1.5 mm vertical × 1.0 mm thickness; it was directly connected to the HPLC system by a tube. 2D SAXS image data were recorded using a PILATUS3 2M detector (DECTRIS). The exposure time and the number of frames measured were 20 s and 247, respectively. All the 2D data were converted to 1D data by azimuthal averaging, and the background data for the buffer region (average data of 20 frames) measured before the sample measurement were subtracted from all the data. The scattering intensities were converted to an absolute intensity scale based on the water scattering intensities. These data reduction processes were performed using SAngler software[20]. The system allows simultaneous UV-visible absorption spectroscopy using a SAXS sample cell and a fiber spectrophotometer (QE65pro, Ocean Insight) positioned at a 45-degree angle to the X-ray to estimate the concentration of the sample during SAXS measurements. Measurements of absorption spectra in the wavelength range from 200 to 450 nm were performed at 10-second

intervals with an integration time of 1 s and repeated 495 times. The SAXS and UV-visible absorption data were analyzed using MOLASS software[21]. Because the ascending and descending sides of the elution peak showed no differences in scattering profile shape, $R_g$ values, etc., and no interparticle interference effects were observed throughout the peak, almost the entire peak from frame numbers 96 to 146 was used to calculate the SAXS profile for the form factor, that is, the SAXS profile extrapolated to zero concentration. The software AUTORG and GNOM from ATSAS were used for the Guinier analysis and the calculation of the pair distance distribution function (PDDF), respectively[22,23]. The DAMMIF module of PRIMUSqt from ATSAS was used to calculate the bead model[24]. CRYSOL calculated the theoretical SAXS profile from the crystal structure of EndoMA1 (PDB entry 8HHV) and fitted it to the experimental profile[25]. The superposition of the crystal structure and the bead model was performed using SUPCOMB software from ATSAS[26]. The details of the experimental conditions and analysis of these SEC-SAXS experiments are summarized in Dataset 1. The SAXS data for the structure modeling were submitted to the small-angle scattering biological data bank (SASBDB) with the deposition ID of SASDQQ8. Fig. 6d and Supplementary Fig. 16 were created using Igor Pro 8 (WaveMetrics). The outer shape of the bead model in Fig. 6e is drawn in Chimera 1.16[27]. The resolution value, indicating the smoothness of the outer shape, was set to 15 Å.

# Synthesis of oligo-D-arabinofuranosides

Akihiro Ishiwata,* Katsunori Tanaka,
Yukishige Ito

*Correspondence to A. Ishiwata. Email: aishiwa@riken.jp

**General procedure**

All reactions sensitive to air and/or moisture were carried out under argon atmosphere with anhydrous solvents. Substrates of glycosylations were dried by azeotropic removal with toluene. Column chromatography was performed on silica gel 60N, 100–210 mesh (Kanto Kagaku Co., Ltd.). Preparative thin layer chromatography (PTLC) was performed on silica gel 60 F254, 0.5 mm (E. Merck). Optical rotations were measured with a JASCO DIP 370 polarimeter. $^{1}$H NMR and $^{13}$C NMR spectra were recorded on a JEOL ECX400 spectrometer. $^{1}$H NMR spectra were referenced to $CHCl_3$ at 7.24, HOD at 4.80, and MeOH at 3.31 ppm. $^{13}$C NMR spectra were referenced to the central peak of $CDCl_3$ at 77.0 ppm, $CD_3OD$ at 49.0 ppm, and native scale except otherwise mentioned. The NMR spectra are supplied in Supplementary Data 2. Assignments were made by standard pfg COSY, pfg HMQC or pfg HMBC, and so on. HRMS determinations were made by the use of a JEOL AccuTOF JMS-T700LCK mass spectrometer with $CF_3CO_2Na$ as the internal standard. All other reagents were purchased from Wako Pure Chemical Industries Ltd., Kanto Chemicals Co. Inc., Tokyo Chemical Industries Co., Ltd., and Sigma-Aldrich Chemical Co. *p*-Nitrophenyl β-D-Ara*f* (*p*NP-β-D-Ara*f*) synthesized according to the previous procedure for the synthesis of the *p*NP-β-L-Ara*f*[28] from D-arabinose instead of L-arabinose, methyl β-D-Ara*f* (Me-β-D-Ara*f*)[28,29] was synthesized according to the reported procedure, and **A22BβT** was synthesized as reported previously[30].

**v**

## Synthesis

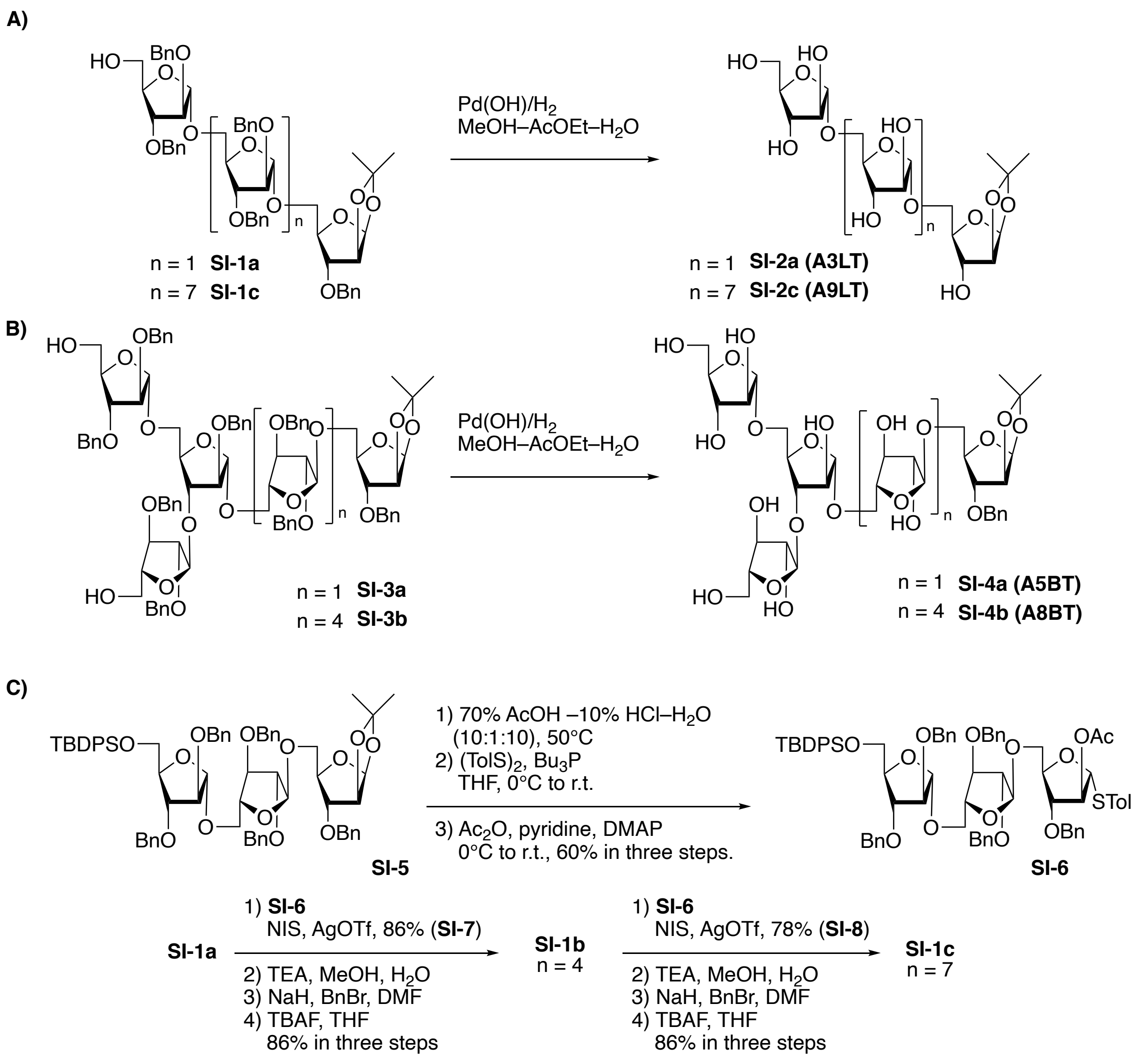


**Supplementary Figure SI-1. Preparation of acetonide derivative of oligo-D-arabinofuranosides, A3LT, A5BT, A8BT and A9LT.** A) Synthesis of A3LT and A9LT from protected starting materials: B) Synthesis of A5BT and A8BT from protected starting materials: C) Fragment couplings toward the protected A9LT. Abbreviations; Bn: benzyl, MeOH: methanol, AcOEt: ethyl acetate, TBDPS: *tert*-butyldiphenylsilyl, AcOH: acetic acid, Tol: tolyl, Bu: *normal*-butyl, Ac: acetyl, DMAP: 1-(*N,N*-dimethylamino)pyridine, NIS: *N*-iodosuccinimide, DMF: *N,N*-dimethylformamide, TBAF: tetra-(*normal*-butyl)-ammonium fluoride, THF: tetrahydrofuran.

### 1) Synthesis of A3LT (SI-2a).

**α-D-Arabinofuranosyl-(1→5)-α-D-arabinofuranosyl-(1→5)-1,2-*O*-isopropylidene-β-D-arabinofuranose (SI-2a).**

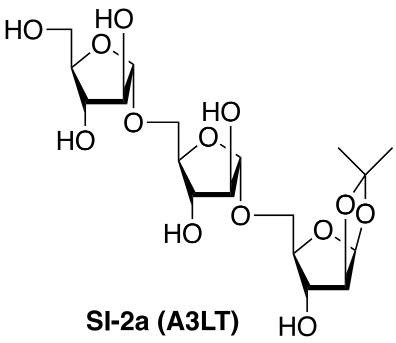

**SI-2a (A3LT)**

Hydrogenolysis of 2,3-di-*O*-benzyl-α-D-arabinofuranosyl-(1→5)-2,3-di-*O*-benzyl-α-D-arabinofuranosyl-(1→5)-3-*O*-benzyl-1,2-*O*-isopropylidene-β-D-arabinofuranose (**SI-1a**)[30] (130 mg, 118 μmol) was carried out in the presence of $Pd(OH)_2$ (130 mg) in EtOAc–MeOH–$H_2O$ (5:5:1, 11 mL) for 16 h at room temperature. After replacement to Ar from $H_2$ atmosphere, the mixture was filtered through a Celite pad, and the filtrate was concentrated *in vacuo* to give the trisaccharide A3LT (**SI-2a**, 47.1 mg, 88%).

α-D-Arabinofuranosyl$^{C}$-(1→5)-α-D-arabinofuranosyl$^{B}$-(1→5)-1,2-*O*-isopropylidene-β-D-arabinofuranose$^{A}$ (A3LT, **SI-2a**): $[\alpha]^{26}_{D}$ 102.79° (*c* 1.00, $CH_3OH$); $^{1}H$ NMR ($CD_3OD$, 400 MHz): δ 1.13 (s, 3 H, $CH_3$), 1.52 (s, 3 H, $CH_3$), 3.58 (dd, *J* = 10.4, 6.4 Hz, 1 H, C5$^{A}$-H), 3.64 (dd, *J* = 12.0, 5.6 Hz, 1 H, C5$^{C}$-H), 3.65 (dd, *J* = 12.0, 4.4 Hz, 1 H, C5$^{B}$-H), 3.75 (dd, *J* = 12.0, 3.2 Hz, 1 H, C5$^{C}$-H), 3.84 (td, *J* = 5.6, 3.2 Hz, 1 H, C3$^{C}$-H), 3.85 (dd, *J* = 12.0, 5.2 Hz, 1 H, C5$^{B}$-H), 3.87 (dd, *J* = 10.4, 7.6 Hz, 1 H, C5$^{A}$-H), 3.90 (td, *J* = 6.4, 3,6 Hz, 1 H, C3$^{B}$-H), 3.97 (td, *J* = 5.6, 3.2 Hz, 1 H, C4$^{C}$-H), 3.99 (d, *J* = 3.6 Hz, 1 H, C2$^{B}$-H), 4.00 (dd, *J* = 3.2, 0.8 Hz, 1 H, C2$^{C}$-H), 4.09 (ddd, *J* = 6.4, 5.2, 3.6 Hz, 1 H, C4$^{B}$-H), 4.11 (ddd, *J* = 7.6, 6.4, 2.0 Hz, 1 H, C4$^{A}$-H), 4.17 (d, *J* = 2.0 Hz, 1 H, C3$^{A}$-H), 4.52 (d, *J* = 4.0 Hz, 1 H, C2$^{A}$-H), 4.89 (s, 1 H, C1$^{B}$-H), 4.94 (d, *J* = 0.8 Hz, 1 H, C1$^{C}$-H), 5.89 (d, *J* = 4.0 Hz, 1 H, C1$^{A}$-H); $^{13}C$ NMR ($CD_3OD$, 100 MHz): δ 26.2 ($CH_3$), 27.2 ($CH_3$), 63.1 (C5$^{C}$), 68.0 (C5$^{B}$), 68.5 (C5$^{A}$), 76.6 (C3$^{A}$), 78.7 (C3$^{C}$), 79.3 (C3$^{B}$), 83.1 (C2$^{C}$), 83.4 (C2$^{B}$), 84.0 (C4$^{B}$), 85.8 (C4$^{C}$), 87.7 (C4$^{A}$), 88.2 (C2$^{A}$), 107.2 (C1$^{A}$), 109.5 (C1$^{B}$) 109.6 (C1$^{C}$), 113.7 (acetonide); MALDI-TOF MS: calcd for $C_{18}H_{30}Na_1O_{13}$ $[M+Na]^+$ 477.16, found 477.23.

**2) Synthesis of A5BT (SI-4a).**

**α-D-Arabinofuranosyl-(1→5)-[α-D-arabinofuranosyl-(1→3)]-α-D-arabinofuranosyl-(1→5)-α-D-arabinofuranosyl-(1→5)-1,2-*O*-isopropylidene-β-D-arabinofuranose (SI-4a).**

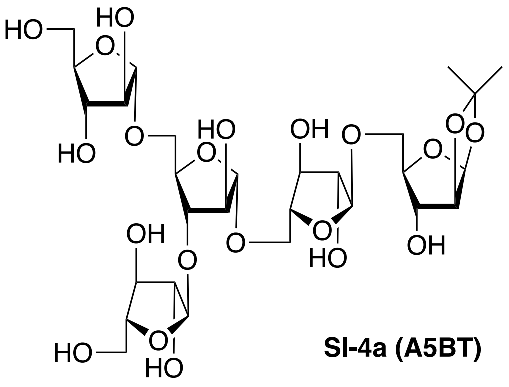

**SI-4a (A5BT)**

Hydrogenolysis of 2,3-di-*O*-benzyl-α-D-arabinofuranosyl-(1→5)-[2,3-di-*O*-benzyl-α-D-arabinofuranosyl-(1→3)]-2-*O*-benzyl-α-D-arabinofuranosyl-(1→5)-2,3-di-*O*-benzyl-α-D-arabinofuranosyl-(1→5)-3-*O*-benzyl-1,2-*O*-isopropylidene-β-D-arabinofuranose (**SI-3a**)[30] (34.4 mg, 23.5 μmol) was carried out in the presence of $Pd(OH)_2$ (34.3 mg) in MeOH–$H_2O$ (5:1, 6.0 mL) for 16 h at room temperature. After replacement to Ar from $H_2$ atmosphere, the mixture was filtered through a Celite pad, and the filtrate was concentrated *in vacuo* to give the title compound A5BT (**SI-4a**, 16.1 mg, 95%).

α-D-Arabinofuranosyl$^{E}$-(1→5)-[α-D-arabinofuranosyl$^{D}$-(1→3)]-α-D-arabinofuranosyl$^{C}$-(1→5)-α-D-arabinofuranosyl$^{B}$-(1→5)-1,2-*O*-isopropylidene-β-D-arabinofuranose$^{A}$ (A5BT, **SI-4a):** $[\alpha]^{22}_{D}$ 178.18 (*c* 0.88, $H_2O$); $^{1}$H NMR ($D_2O$, 400 MHz): δ 1.35 (s, 3 H, $CH_3$), 1.55 (s, 3 H, $CH_3$), 3.64–3.71 (m, 3 H, C5$^{A}$-H, C5$^{D}$-H, C5$^{E}$-H), 3.75 (dd, *J* = 12.0, 3.2 Hz, 1 H, C5$^{B}$-H), 3.78–3.86 (m, 4 H, C5$^{B}$-H, C5$^{C}$-H, C5$^{D}$-H, C5$^{E}$-H), 3.87–3.95 (m, 4 H, C3$^{D}$-H, C3$^{E}$-H, C5$^{A}$-H, C5$^{C}$-H), 3.98–4.03 (m, 2 H, C3$^{B}$-H, C4$^{D}$-H), 4.05–4.11 (m, 5 H, C2$^{B}$-H, C2$^{D}$-H, C2$^{E}$-H, C3$^{C}$-H, C4$^{E}$-H), 4.16 (ddd, *J* = 6.0, 5.6, 3.2 Hz, 1 H, C4$^{B}$-H), 4.23–4.29 (m, 4 H, C2$^{C}$-H, C3$^{A}$-H, C4$^{A}$-H, C4$^{C}$-H), 4.71 (d, *J* = 4.0 Hz, 1 H, C2$^{A}$-H), 5.02 (d, *J* = 1.6 Hz, 1 H, C1$_{B}$-H), 5.05 (d, *J* = 1.2 Hz, 1 H, C1$^{D/E}$-H), 5.08 (s, 1 H, C1$^{C}$-H), 5.12 (d, *J* = 1.6 Hz, 1 H, C1$^{D/E}$-H), 6.03 (d, *J* = 4.0 Hz, 1 H, C1$^{A}$-H); $^{13}$C NMR ($D_2O$, 100 MHz): δ 25.0 ($CH_3$), 25.7 ($CH_3$), 61.07 (C5$^{D/E}$), 61.13 (C5$^{D/E}$), 66.1 (C5$^{B}$), 66.4 (C5$^{C}$), 67.1 (C5$^{A}$), 74.7 (C3$^{A}$), 76.5 (x2) (C3$^{D}$, C3$^{E}$), 76.7 (C3$^{B}$), 79.1 (C2$^{C}$), 80.9 (C3$^{C}$), 81.0 (C2$^{D/E}$), 81.2 (C2$^{D/E}$), 81.6 (C2$^{b}$), 82.0 (C4$^{C}$), 82.3 (C4$^{B}$), 83.9 (C4$^{D/E}$), 84.0 (C4$^{D/E}$), 85.9 (C4$^{A}$), 86.1 (C2$^{A}$), 105.5 (C1$^{A}$), 107.1, 107.33, 107.36, 107.42 (C1 x4), 113.4 (acetonide); MALDI-TOF MS: calcd for $C_{28}H_{46}Na_{1}O_{21}$ $[M+Na]^{+}$ 741.24, found 741.69.

**3) Synthesis of A8BT (SI-4B).**

**α-D-Arabinofuranosyl-(1→5)-[α-D-arabinofuranosyl-(1→3)]-α-D-arabinofuranosyl-(1→5)-α-D-arabinofuranosyl-(1→5)-α-D-arabinofuranosyl-(1→5)-α-D-arabinofuranosyl-(1→5)-α-D-arabinofuranosyl-(1→5)-1,2-*O*-isopropylidene-β-D-arabinofuranose (SI-4B).**

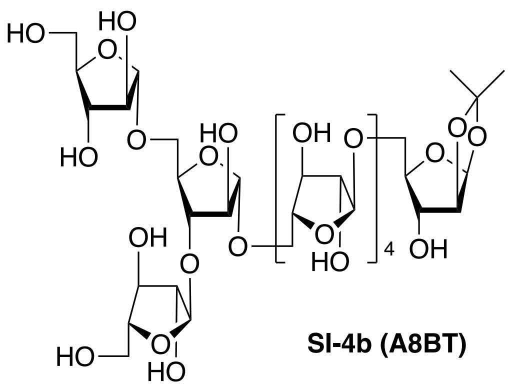


**SI-4b (A8BT)**

Hydrogenolysis of 2,3-di-*O*-benzyl-α-D-arabinofuranosyl-(1→5)-[2,3-di-*O*-benzyl-α-D-arabinofuranosyl-(1→3)]-2-*O*-benzyl-α-D-arabinofuranosyl-(1→5)-2,3-di-*O*-benzyl-α-D-arabinofuranosyl-(1→5)-2,3-di-*O*-benzyl-α-D-arabinofuranosyl-(1→5)-2,3-di-*O*-benzyl-α-D-arabinofuranosyl-(1→5)-2,3-di-*O*-benzyl-α-D-arabinofuranosyl-(1→5)-3-*O*-benzyl-1,2-*O*-isopropylidene-β-D-arabinofuranose (**SI-3b**)[30] (8.9 mg, 3.7 µmol) was carried out in the presence of $Pd(OH)_2$ (8.9 mg) in EtOAc–MeOH–$H_2O$ (5:10:2, 8.5 mL) for 16 h at room temperature. After replacement to Ar from $H_2$ atmosphere, the mixture was filtered through a Celite pad, and the filtrate was concentrated *in vacuo* to give the title compound A8BT (**SI-4b**, 4.1 mg, quant.).

α-D-Arabinofuranosy$^{E}$-(1→5)-[α-D-arabinofuranosyl$^{D}$-(1→3)]-α-D-arabinofuranosyl$^{C}$-(1[→5)-α-D-arabinofuranosyl$^{B}$-(1]$_4$→5)-1,2-*O*-isopropylidene-β-D-arabinofuranose$^{A}$ (A8BT, **SI-4b**): $[\alpha]^{22}_{D}$ 68.00 (*c* 0.10, $H_2O$); $^{1}$H NMR ($D_2O$, 400 MHz): δ 1.35 (s, 3 H, $CH_3$), 1.55 (s, 3 H, $CH_3$), 3.59–3.73 (m, 3 H, C5$^{A}$-H, C5$^{D}$-H, C5$^{E}$-H), 3.74–3.90 (m, 11 H, C5$^{B}$-H x8, C5$^{C}$-H, C5$^{D}$-H, C5$^{E}$-H), 3.90–3.95 (m, 4 H, C3$^{D}$-H, C3$^{E}$-H, C5$^{A}$-H, C5$^{C}$-H), 3.96–4.04 (m, 5 H, C3$^{B}$-H x4, C4$^{E}$-H), 4.05–4.13 (m, 8 H, C2$^{B}$-H x4, C2$^{C}$-H, C3$^{C}$-H, C2$^{E}$-H, C4$^{D}$-H), 4.15–4.21 (m, 4 H, C4$^{B}$-H x4), 4.22–4.31 (m, 4 H, C2$^{D}$-H, C3$^{A}$-H, C4$^{A}$-H, C4$^{C}$-H), 4.71 (d, $J$ = 4.4 Hz, 1 H, C2$^{A}$-H), 5.03 (s, 1 H, C1$^{E}$-H), 5.06 (s, 4 H, C1$^{B}$-H x4), 5.09 (s, 1 H, C1$^{D}$-H), 5.13 (s, 1 H, C1$^{C}$-H), 6.03 (d, $J$ = 4.4 Hz, 1 H, C1$^{A}$-H); $^{13}$C NMR ($D_2O$, 100 MHz): δ 61.08 (C5$^{D/E}$), 61.13 (C5$^{D/E}$), 66.2, 66.4, 66.7, 66.8 (x2) (C5), 67.1 (C5$^{A}$), 74.7 (C3$^{A}$), 76.3, 76.4 (x3), 76.5, 76.7, 76.9 (C3), 79.1 (C2$^{D}$), 80.7, 80.8 (x2), 80.9 (x2), 81.0, 81.2 (C2$^{B}$ x4, C2$^{C}$, C2$^{E}$, C4$^{C}$), 82.17 (C4$^{B}$), 82.19 (C4$^{B}$), 82.3 (C4$^{B}$ x 2), 83.9 (C4$^{E}$), 84.0 (C4$^{D}$), 85.9 (C2$^{A}$), 86.0 (C4$^{A}$), 105.4 (C1$^{A}$), 107.1 (C1$^{C}$), 107.3 (C1$^{D}$), 107.3 (C1$^{B}$ x4, C1$^{E}$), 113.5 (acetonide); MALDI-TOF MS: calcd for $C_{43}H_{70}Na_1O_{33}$ $[M+Na]^+$ 1137.37, found 1137.81.

**4) Synthesis of A9LT (SI-2c):**

The A9LT (**SI-2c**) has been synthesized by fragment couplings (Supplementary Figure SI-1, C).

The hexasaccharide acceptor (**SI-1b**) was obtained by the first fragment coupling between the trisaccharide donor (**SI-6**) and trisaccharide acceptor (**SI-1a**) followed by deprotection in one step. The second fragment coupling of the trisaccharide donor (**SI-6**) with the resultant acceptor (**SI-1b**) afforded nonasaccharide derivative (**SI-1c**) which was then converted to the desired A9LT (**SI-2c**) (Supplementary Figure SI-1, A).

**Tolyl 5-*O*-*t*-butyldiphenylsilyl-2,3-di-*O*-benzyl-α-D-arabinofuranosyl-(1→5)-2,3-di-*O*-benzyl-α-D-arabinofuranosyl-(1→5)-2-*O*-acetyl-3-*O*-benzyl-1-thio-α-D-arabinofuranoside (SI-6).**

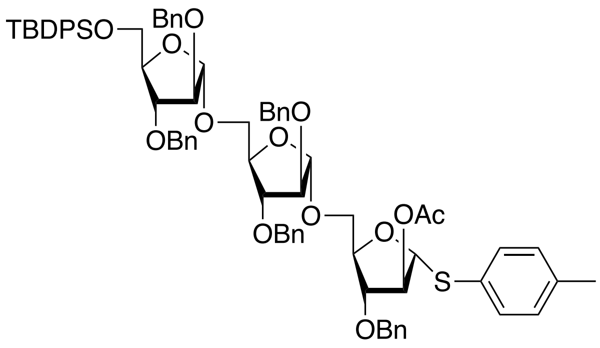


The trisaccharide donor was synthesized from 5-*O*-*t*-butyldiphenylsilyl-2,3-di-*O*-benzyl-α-D-arabinofuranosyl-(1→5)-2,3-di-*O*-benzyl-α-D-arabinofuranosyl-(1→5)-3-*O*-benzyl-1,2-*O*-isopropylidene-β-D-arabinofuranose (**SI-5**) as reported previously[30]. A solution of 5-*O*-*t*-butyldiphenylsilyl-2,3-di-*O*-benzyl-α-D-arabinofuranosyl-(1→5)-2,3-di-*O*-benzyl-α-D-arabinofuranosyl-(1→5)-3-*O*-benzyl-1,2-*O*-isopropylidene-β-D-arabinofuranose (**SI-5**) (567 mg, 486 μmol) in THF (10.0 mL), 10% aqueous HCl (1.0 mL) and 70% aqueous AcOH (10.0 mL) was stirred at 50°C for 2 h. After cooling to room temperature, the reaction was quenched with saturated aqueous $NaHCO_3$, extracted with ethyl acetate, washed with brine, dried over $Na_2SO_4$, concentrated *in vacuo,* and azeotropic removal of $H_2O$ with toluene. The residue was used without further purification. To a solution of the crude hemiacetal and di-*p*-tolyl disulfide (180 mg, 729 μmol) in dry $CH_2Cl_2$ (10.0 mL) was added *n*-$Bu_3P$ (188 μL, 730 μmol) at 0°C under Ar atmosphere. After stirring for 2 h at the same temperature, the mixture was concentrated *in vacuo* (MALDI-TOF MS: calcd for $C_{73}H_{80}Na_1O_{12}S_1Si_1$ $[M+Na]^+$ 1231.5, found 1232.0.). To the mixture in pyridine (5.0 mL) was added $Ac_2O$ (1.0 mL) and DMAP (50.0 mg) at 0°C, and the mixture was stirred for 1 h during which time the temperature was going up to room temperature. After being concentrated *in vacuo*, the residue was purified by flash column chromatography using a gradient solvent system (hexane/ethyl acetate) to give the title compound (**SI-6**) (366 mg, 60% in three

steps) as the mixture of anomers.

Tolyl 5-*O*-*t*-butyldiphenylsilyl-2,3-di-*O*-benzyl-α-D-arabinofuranosyl$^{C}$-(1→5)-2,3-di-*O*-benzyl-α-D-arabinofuranosyl$^{B}$-(1→5)-2-*O*-acetyl-3-*O*-benzyl-1-thio-α-D-arabinofuranoside$^{A}$ (**SI-6**); major isomer: $^{1}$H NMR ($CDCl_3$, 400 MHz): δ 0.96 (s, 9 H, TBDPS), 1.86 (s, 3 H, Ac), 2.22 (s, 3 H, $CH_3$Ph) 3.46–3.64 (m, 2 H, C5$^{A}$-H, C5$^{B}$-H), 3.68–3.83 (m, 4 H, C5$^{A}$-H, C5$^{B}$-H, C5$^{C}$-$H_2$), 3.90–4.10 (m, 6 H, C2$^{B}$-H, C2$^{C}$-H, C3$^{A}$-H, C3$^{C}$-H, C4$^{B}$-H, C4$^{C}$-H), 4.31–4.65 (m, 11 H, Bn-H x10, C4$^{A}$-H), 4.99 (s, 1 H, C1-H), 5.06 (s, 1 H, C1-H), 5.20 (t, $J$ = 1.6 Hz, 1 H, C2$^{A}$-H), 5.39 (s, 1 H, C1$^{A}$-H), 6.96–7.62 (m, 39 H, Ar); $^{13}$C NMR ($CDCl_3$, 100 MHz): δ 19.3 (TBDPS), 20.8 (Ac), 21.1 ($CH_3$Ph), 26.8 (TBDPS), 63.7 (C5$^{C}$), 65.58 (C5$^{A}$), 65.62 (C5$^{B}$), 71.7 (Bn), 71.9 (Bn), 72.0 (Bn), 72.3 (Bn x2), 80.6 (C3$^{C}$), 81.5 (C4$^{A}$), 81.9 (C2$^{A}$), 82.6 (C4$^{C}$), 82.9 (C3$^{A}$), 83.1 (C3$^{B}$), 83.2 (C3$^{C}$), 88.0 (C2$^{C}$), 88.5 (C2$^{B}$), 91.3 (C1$^{A}$), 106.2 (C1$^{B}$), 106.4 (C1$^{C}$), 127.60, 127.63, 127.7, 127.8, 127.9, 128.27, 128.32, 128.37, 128.41, 129.6, 129.7, 132.4, 133.4, 135.6, 135.7, 137.5, 135.6, 138.0 (Ar), 169.8 (Ac).; MALDI-TOF MS: calcd for $C_{75}H_{82}Na_1O_{13}S_1Si_1$ $[M+Na]^+$ 1273.5, found 1272.9; ESI-TOF MS: Calcd for $C_{75}H_{82}Na_1O_{13}S_1Si_1$ $[M+Na]^+$ (*m/z*) 1273.52 found 1273.53; HR ESI-TOF MS: Calcd for $C_{75}H_{82}Na_1O_{13}S_1Si_1$ $[M+Na]^+$ (*m/z*) 1273.5161, found 1273.5143.

**5-*O*-*t*-Butyldiphenylsilyl-2,3-di-*O*-benzyl-α-D-Arabinofuranosyl-(1→5)-2,3-di-*O*-benzyl-α-D-arabinofuranosyl-(1→5)-2-*O*-acetyl-3-*O*-benzyl-α-D-arabinofuranosyl-(1→5)-2,3-di-*O*-benzyl-α-D-arabinofuranosyl-(1→5)-2,3-di-*O*-benzyl-α-D-arabinofuranosyl-(1→5)-3-*O*-benzyl-1,2-*O*-isopropylidene-β-D-arabinofuranose (SI-7).**

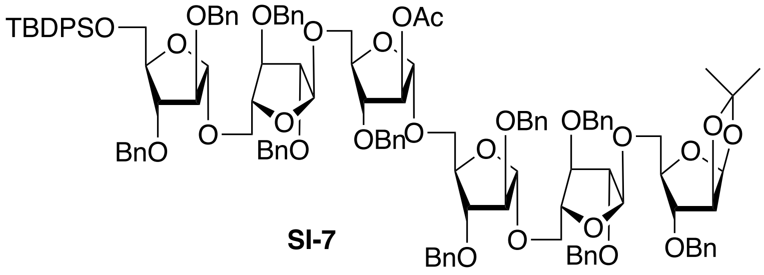


To the mixture of Ara*f*3 acceptor (**SI-1a**)[30] (68.2 mg, 75.4 μmol) and Ara*f*3 donor (**SI-6**) (99.0 mg, 79.1 μmol) in dry $CH_2Cl_2$ (2 mL), MS 4Å (250 mg, freshly dried) was added at room temperature. After cooling to –30°C, NIS (29.6 mg, 128 μmol) and AgOTf (2.0 mg, 7.8 μmol) were added to the mixture and the mixture was stirred for 24 h during which time temperature was going up to 0 °C. Then the reaction was quenched by triethylamine followed by filtration through a Celite pad and washing of the pad with $CHCl_3$. The combined solutions were washed with 20% aqueous $Na_2S_2O_3$, saturated aqueous $NaHCO_3$, and brine, dried over $Na_2SO_4$, and concentrated *in vacuo*. The residue was purified by gel filtration (Bio beads SX-3, toluene/ethyl acetate = 1/1) to give

the title compound Ara$f_6$ (132 mg, 86%).

5-*O*-*t*-Butyldiphenylsilyl-2,3-di-*O*-benzyl-α-D-arabinofuranosyl$^F$-(1→5)-2,3-di-*O*-benzyl-α-D-arabinofuranosyl$^E$-(1→5)-2-*O*-acetyl-3-*O*-benzyl-α-D-arabinofuranosyl$^D$-(1→5)-2,3-di-*O*-benzyl-α-D-arabinofuranosyl$^C$-(1→5)-2,3-di-*O*-benzyl-α-D-arabinofuranosyl$^B$-(1→5)-3-*O*-benzyl-1,2-*O*-isopropylidene-β-D-arabinofuranose$^A$ (**SI-7**): $[\alpha]^{24}_D$ 66.0° (*c* 1.00, $CHCl_3$); $^1$H NMR ($CDCl_3$, 400 MHz): δ 0.96 (s, 9 H, TBDPS), 1.25 (s, 3 H, acetonide), 1.42 (s, 3 H, acetonide), 1.81 (s, 3 H, Ac), 3.46–3.62 (m, 5 H, C5$^A$-H, C5$^B$-H, C5$^C$-H, C5$^D$-H, C5$^E$-H), 3.68–3.83 (m, 8 H, C3$^D$-H, C5$^A$-H, C5$^B$-H, C5$^C$-H, C5$^D$-H, C5$^E$-H, C5$^F$-H x2), 3.91–4.01 (m, 10 H, C2$^B$-H, C2$^C$-H, C2$^E$-H C2$^F$-H, C3$^A$-H, C3$^B$-H, C3$^C$-H, C3$^E$-H, C3$^F$-H, C4$^E$-H), 4.02–4.18 (m, 5 H, C4$^A$-H, C4$^B$-H, C4$^C$-H, C4$^D$-H, C4$^F$-H), 4.28–4.52 (m, 19 H, Bn-H x19), 4.56 (d, *J* = 4.0 Hz, 1 H, C2$^A$-H), 4.62 (d, *J* = 12.4 Hz, 1 H, Bn-H), 4.94 (s, 1 H, C1$^{B/C/E/F}$-H), 4.98 (s, 1 H, C1$^{B/C/E/F}$-H), 5.02 (s, 1 H, C1$^D$-H), 5.04 (s, 1 H, C1$^{B/C/E/F}$-H), 5.06 (s, 1 H, C1$^{B/C/E/F}$-H), 5.09 (s, 1 H, C2$^D$-H), 5.81 (d, *J* = 4.0 Hz, 1 H, C1$^A$-H), 7.14–7.70 (m, 65 H, Ar); $^{13}$C NMR ($CDCl_3$, 100 MHz): δ 19.3 (TBDPS), 20.8 (Ac), 26.4 (Acetonide), 26.8 (TBDPS), 27.2 (Acetonide), 63.6 (C5$^F$), 65.57 (C1$^{B/C/D/E}$), 65,66 (C1$^{B/C/D/E}$), 65.69 (C1$^{B/C/D/E}$), 65.79 (C1$^{B/C/D/E}$), 66.8 (C5$^A$), 71.66 (Bn x2), 71.74 (Bn), 71.8 (Bn), 72.0 (Bn x2), 72.14 (Bn), 72.17 (Bn), 72.21 (Bn), 72.3 (Bn), 80.3 (C4$^F$), 80.53 (C4$^{B/C/E}$), 80.56 (C4$^{B/C/E}$ x2), 81.3 (C2$^D$), 82.0 (C4$^D$), 82.5 (C4$^A$), 82.8 (C3$^{A–F}$), 82.9 (C3$^{A–F}$), 83.0 (C3$^{A–F}$), 83.1 (C3$^{A–F}$), 83.2 (C3$^{A–F}$ x2), 85.3 (C2$^A$), 87.9 (C2$^{B/C/E/F}$), 88.0 (C2$^{B/C/E/F}$), 88.5 (C2$^{B/C/E/F}$ x2), 105.5 (C1$^A$), 106.0 (C1$^D$), 106.20 (C1$^{B/C/E/F}$), 106.3 (C1$^{B/C/E/F}$), 106.4 (C1$^{B/C/E/F}$ x2), 112.8 (Acetonide), 127.56, 127.61, 127.68, 127.71, 127.8, 128.27, 128.30, 128.34, 128.4, 129.56, 129.59, 133.4, 135.58, 135.62, 137.28, 137.49, 137.51, 137.62, 137.67, 137.78, 137.91, 137.99 (Ar), 169.7 (Ac); MALDI-TOF MS: calcd for $C_{121}H_{134}Na_1O_{26}Si_1$ $[M+Na]^+$ 2053.9, found 2053.2; HR ESI-TOF MS: Calcd for $C_{121}H_{134}Na_1O_{26}Si_1$ $[M+Na]^+$ (*m/z*) 2053.88, found 2053.07; HR ESI-TOF MS: Calcd for $C_{121}H_{134}Na_1O_{26}Si_1$ $[M+Na]^+$ (*m/z*) 2053.8830, found 2053.8825.

**2,3-Di-*O*-benzyl-α-D-arabinofuranosyl-(1→5)-2,3-di-*O*-benzyl-α-D-arabinofuranosyl-(1→5)-2,3-di-*O*-benzyl-α-D-arabinofuranosyl-(1→5)-2,3-di-*O*-benzyl-α-D-arabinofuranosyl-(1→5)-2,3-di-*O*-benzyl-α-D-arabinofuranosyl-(1→5)-3-*O*-benzyl-1,2-*O*-isopropylidene-β-D-arabinofuranose (SI-1b).**

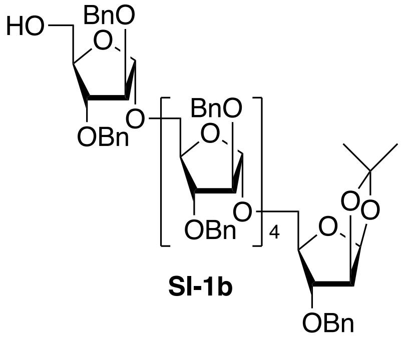

**SI-1b**

To a solution of 5-*O*-*t*-butyldiphenylsilyl-2,3-di-*O*-benzyl-α-D-arabinofuranosyl-(1→5)-2,3-di-*O*-benzyl-α-D-arabinofuranosyl-(1→5)-2-*O*-acetyl-3-*O*-benzyl-α-D-arabinofuranosyl-(1→5)-2,3-di-*O*-benzyl-α-D-arabinofuranosyl-(1→5)-2,3-di-*O*-benzyl-α-D-arabinofuranosyl-(1→5)-3-*O*-benzyl-1,2-*O*-isopropylidene-β-D-arabinofuranose (**SI-7**) (86.0 g, 42.3 μmol) in MeOH (2 mL) and $H_2O$ (0.5 mL) was added $Et_3N$ (2.0 mL) at room temperature. After stirring for 24 h at the same temperature, the mixture was concentrated *in vacuo* to give a crude deacylated compound. After azeotropic removal of residual $H_2O$ with toluene, to a solution of the residue in dry DMF (1.0 mL) was added NaH (2.4 mg, 60.0 μmol) and BnBr (5.5 μL, 46 μmol) at 0°C, and the mixture was stirred for 2 h during which time temperature was going up to room temperature. The reaction was quenched by sat. $NH_4Cl$ aq., extracted with EtOAc washed with brine, dried over $Na_2SO_4$, and evaporated *in vacuo*. The residue was used without further purification. To a solution of the residue in dry THF (1.0 mL), 1 M solution of TBAF in THF (0.5 mL) was added at room temperature. After stirring for 4 h at the same temperature, the mixture was concentrated *in vacuo*. The residue was purified by PTLC (hexane/ethyl acetate =1/1) to give the title compound (**SI-1b**) (76.0 mg, 86% in three steps).

2,3-Di-*O*-benzyl-α-D-arabinofuranosyl$^C$-(1[→5)-2,3-di-*O*-benzyl-α-D-arabinofuranosyl$^B$-(1]$_4$→5)-3-*O*-benzyl-1,2-*O*-isopropylidene-β-D-arabinofuranose$^A$ (**SI-1b**): $[\alpha]^{25}_D$ 81.8° (*c* 1.00, $CHCl_3$); $^1$H NMR ($CDCl_3$, 400 MHz): δ 1.25 (s, 3 H, acetonide), 1.42 (s, 3 H, acetonide), 1.65 (br s, 1 H, OH), 3.46–3.62 (m, 6 H, C5$^A$-H, C5$^B$-H x4, C5$^C$-H), 3.68–3.83 (m, 6 H, C5$^A$-H, C5$^B$-H x4, C5$^C$-H), 3.88 (dd, *J* = 5.6, 4.0 Hz, 1 H, C3$^C$-H), 3.92–4.10 (m, 14 H, C2$^B$-H x4, C2$^C$-H, C3$^A$-H, C3$^B$-H x4, C4$^B$-H x4, C4$^C$-H), 4.14 (ddd, *J* = 6.8, 5.2, 3.6 Hz, 1 H, C4$^A$-H), 4.28–4.52 (m, 22 H, Bn x22), 4.57 (d, *J* = 4.0 Hz, 1 H, C2$^A$-H), 4.94 (s, 1 H, C1$^B$-H), 5.03 (s, 2 H, C1$^{B/C}$-H x2), 5.04 (s, 1 H, C1$^{B/C}$-H), 5.05 (s, 1 H, C1$^{B/C}$-H), 5.81 (d, *J* = 4.0 Hz, 1 H, C1$^A$-H), 7.14–7.25 (m, 55 H, Ar); $^{13}$C NMR ($CDCl_3$, 100 MHz): δ 26.4 (Acetonide), 27.2 (Acetonide), 62,1 (C5$^C$), 65.7 (C5$^B$), 65.9 (C5$^B$ x3), 66.8 (C5$^A$), 71.7 (Bn), 71.8 (Bn), 71.9 (Bn x3), 72.0 (Bn), 72.20 (Bn x2), 72.23 (Bn x2), 72.3 (Bn), 80.34, 80.36, 80.42, 80.5 (C4$^B$ x3, C4$^C$), 82.0 (C4$^A$), 82.7, 82.8, 82.9, 83.12, 83.15 (x3), 83.23 (C3$^A$, C3$^B$ x4, C3$^C$, C4$^B$), 85.3 (C2$^A$), 87.6, 88.20, 88.24, 88.3, 88.5 (C2$^B$ x4,

C2[C]), 105.5 (C1[B]), 106.2 (C1[B/C]), 106.41 (C1[B/C]), 106.45 (C1[B/C] x2), 106.49 (C1[A]), 112.9 (Acetonide), 127,7, 127.8, 127.9, 128.30, 128.34, 137.4, 137.6, 137.89, 137.93 (Ar); MALDI-TOF MS: calcd for $C_{110}H_{120}Na_1O_{25}$ $[M+Na]^+$ 1863.8, found 1862.8; HR ESI-TOF MS: Calcd for $C_{110}H_{120}Na_1O_{25}$ $[M+Na]^+$ (*m/z*) 1863.80, found 1863.83; HR ESI-TOF MS: Calcd for $C_{110}H_{120}Na_1O_{25}$ $[M+Na]^+$ (*m/z*) 1863.8016, found 1863.8063.

**5-*O*-*t*-Butyldiphenylsilyl-2,3-di-*O*-benzyl-α-D-arabinofuranosyl-(1→5)-2,3-di-*O*-benzyl-α-D-arabinofuranosyl-(1→5)-2-*O*-acetyl-3-*O*-benzyl-α-D-arabinofuranosyl-(1→5)-2,3-di-*O*-benzyl-α-D-arabinofuranosyl-(1→5)-2,3-di-*O*-benzyl-α-D-arabinofuranosyl-(1→5)-2,3-di-*O*-benzyl-α-D-arabinofuranosyl-(1→5)-2,3-di-*O*-benzyl-α-D-arabinofuranosyl-(1→5)-2,3-di-*O*-benzyl-α-D-arabinofuranosyl-(1→5)-3-*O*-benzyl-1,2-*O*-isopropylidene-β-D-arabinofuranose Ara*f*$_9$LT (SI-8).**

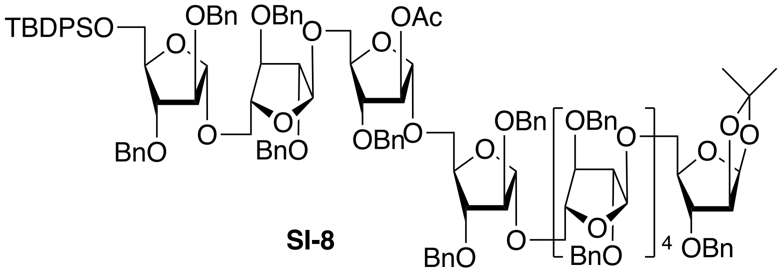


**SI-8**

Ara*f*$_9$ has been synthesized from the Ara*f*$_6$ acceptor (**SI-1b**) with the Ara*f*$_3$ donor (**SI-6**) according to a general fragment coupling procedure for the synthesis of **SI-7** from **SI-1a** with **SI-6**.

To the mixture of Ara*f*$_6$ acceptor (**SI-1b**) (12.0 mg, 6.5 μmol) and Ara*f*$_3$ donor (**SI-6**) (8.1 mg, 6.5 μmol) in dry $CH_2Cl_2$ (2 mL) was added MS 4Å (250 mg, freshly dried) at room temperature. After cooling to –40°C, NIS (2.6 mg, 11.2 μmol) and AgOTf (1.0 mg, 3.9 μmol) were added to the mixture and the mixture was stirred for 24 h during which time temperature was going up to 0°C. Then the reaction was quenched by triethylamine followed by filtration through a Celite pad and washing of the pad with $CHCl_3$. The combined solutions were washed with 20% aqueous $Na_2S_2O_3$, saturated aqueous $NaHCO_3$, and brine, dried over $Na_2SO_4$, and concentrated *in vacuo*. The residue was purified by PTLC (hexane/ethyl acetate = 2/1) to give the title compound Ara*f*$_9$ (**SI-8**) (15.0 mg, 788%).

5-*O*-*t*-Butyldiphenylsilyl-2,3-di-*O*-benzyl-α-D-arabinofuranosyl[I]-(1→5)-2,3-di-*O*-benzyl-α-D-arabinofuranosyl[H]-(1→5)-2-*O*-acetyl-3-*O*-benzyl-α-D-arabinofuranosyl[G]-(1→5)-2,3-di-*O*-benzyl-α-D-arabinofuranosyl[F]-(1→5)-2,3-di-*O*-benzyl-α-D-arabinofuranosyl[E]-(1→5)-2,3-di-*O*-benzyl-α-D-arabinofuranosyl[D]-(1→5)-2,3-di-*O*-benzyl-α-D-arabinofuranosyl[C]-(1→5)-2,3-di-*O*-

benzyl-α-D-arabinofuranosyl$^{B}$-(1→5)-3-*O*-benzyl-1,2-*O*-isopropylidene-β-D-arabinofuranose$^{A}$ (**SI-8**): $[\alpha]^{28}_{D}$ 88.0° (*c* 1.00, $CHCl_3$); $^{1}$H NMR ($CDCl_3$, 400 MHz): δ 0.96 (s, 9 H, TBDPS), 1.25 (s, 3 H, acetonide), 1.42 (s, 3 H, acetonide), 1.80 (s, 3 H, Ac), 3.45–3.60 (m, 8 H, C5$^{A}$-H, C5$^{B}$-H, C5$^{C}$-H, C5$^{D}$-H, C5$^{E}$-H, C5$^{F}$-H, C5$^{G}$-H, C5$^{H}$-H), 3.69–3.85 (m, 11 H, C3$^{G}$-H, C5$^{A}$-H, C5$^{B}$-H, C5$^{C}$-H, C5$^{D}$-H, C5$^{E}$-H, C5$^{F}$-H, C5$^{G}$-H, C5$^{H}$-H, C5$^{I}$-H x2), 3.92–4.00 (m, 16 H, C2$^{B}$-H, C2$^{C}$-H, C2$^{D}$-H, C2$^{E}$-H, C2$^{F}$-H, C2$^{H}$-H, C2$^{I}$-H, C3$^{A}$-H, C3$^{B}$-H, C3$^{C}$-H, C3$^{D}$-H, C3$^{E}$-H, C3$^{F}$-H, C3$^{H}$-H, C3$^{I}$-H, C4$^{G}$-H), 4.02–4.17 (m, 8 H, C4$^{A}$-H, C4$^{B}$-H, C4$^{C}$-H, C4$^{D}$-H, C4$^{E}$-H, C4$^{F}$-H, C4$^{H}$-H, C4$^{I}$-H), 4.28–4.51 (m, 31 H, Bn x31), 4.56 (d, *J* = 4.0 Hz, 1 H, C2$^{A}$-H), 4.62 (d, *J* = 12.4 Hz, 1 H, Bn-H), 4.98 (s, 2 H, C1$^{B/C/D/E/F/H/I}$-H x2), 4.98 (s, 1 H, C1$^{B/C/D/E/F/H/I}$-H), 5.03 (s, 3 H, C1$^{B/C/D/E/F/H/I}$-H x3), 5.04 (s, 1 H, C1$^{G}$-H), 5.05 (s, 1 H, C1$^{B/C/D/E/F/H/I}$-H), 5.08 (d, *J* = 1.2Hz, 1 H, C2$^{G}$-H), 5.81 (d, *J* = 4.0 Hz, 1 H, C1$^{A}$-H), 7.10–7.62 (m, 90 H, Ar); $^{13}$C NMR ($CDCl_3$, 100 MHz): δ 19.3 (TBDPS), 20.8 (Ac), 26.4 (Acetonide), 26.8 (TBDPS), 27.2 (Acetonide), 63.6 (C5$^{I}$), 65.6 (C5$^{B/C/D/E/F/G/H}$), 65.7 (C5$^{B/C/D/E/F/G/H}$), 65.8 (C5$^{B/C/D/E/F/G/H}$ x5), 66.8 (C5$^{A}$), 71.68 (Bn x2), 71.75 (Bn), 71.81 (Bn), 72.0 (Bn x5), 72.1 (Bn), 72.2 (Bn x7), 72.3 (Bn), 80.3 (C4$^{I}$), 80.37 (C4$^{B/C/D/E/F/H}$ x2), 80.41 (C4$^{B/C/D/E/F/H}$ x2), 80.5 (C4$^{B/C/D/E/F/H}$), 80.6 (C4$^{B/C/D/E/F/H}$), 81.3 (C2$^{G}$), 82.0 (C4$^{G}$), 82.5 (C4$^{A}$), 82.8 (C3$^{A–I}$), 82.9, (C3$^{A–I}$), 83.0 (C3$^{A–I}$), 83.1 (C3$^{A–I}$ x5), 83.18 (C3$^{A–I}$), 83.22 (C3$^{A–I}$), 85.3 (C2$^{A}$), 87.97 (C2$^{B/C/D/E/F/H/I}$), 88.01 (C2$^{B/C/D/E/F/H/I}$), 88.2 (C2$^{B/C/D/E/F/H/I}$ x3), 88.5 (C2$^{B/C/D/E/F/H/I}$ x2), 105.5 (C1$^{A}$), 106.0 (C1$^{G}$), 106.2 (C1$^{B/C/D/E/F/H/I}$), 106.3 (C1$^{B/C/D/E/F/H/I}$), 106.4 (C1$^{B/C/D/E/F/H/I}$), 106.5 (C1$^{B/C/D/E/F/H/I}$ x4), 112.9 (acetonide), 127.5, 127.6, 127.69, 127.73, 127.86, 127.91, 128.3, 128.4, 129.59, 129.61, 130.1, 133.4, 135.6, 135.7, 137.30, 137.49, 137.55, 137.59, 137.65, 137.70, 137.89, 137.94, 138.0 (Ar), 169.7 (Ac); ESI-TOF MS: Calcd for $C_{176}H_{192}Na_2O_{37}Si_1$ $[M+Na]^{+}$ (*m/z*) 2990.29, found 2990.29; HR ESI-TOF MS: Calcd for $C_{176}H_{192}Na_2O_{37}Si_1$ $[M+Na_2]^{2+}/2$ (*m/z*) 1485.6354, found 1485.6331; Calcd for $C_{176}H_{192}Na_2O_{37}Si_1$ $[M+Na]^{+}$ (*m/z*) 2990.2915, found 2990.2871.

**2,3-Di-*O*-benzyl-α-D-arabinofuranosyl-(1→5)-2,3-di-*O*-benzyl-α-D-arabinofuranosyl-(1→5)-2,3-di-*O*-benzyl-α-D-arabinofuranosyl-(1→5)-2,3-di-*O*-benzyl-α-D-arabinofuranosyl-(1→5)-2,3-di-*O*-benzyl-α-D-arabinofuranosyl-(1→5)-2,3-di-*O*-benzyl-α-D-arabinofuranosyl-(1→5)-2,3-di-*O*-benzyl-α-D-arabinofuranosyl-(1→5)-2,3-di-*O*-benzyl-α-D-arabinofuranosyl-(1→5)-3-*O*-benzyl-1,2-*O*-isopropylidene-β-D-arabinofuranose (SI-1c).**

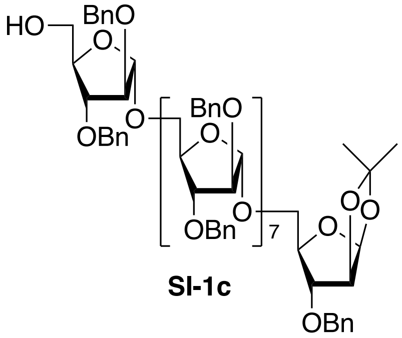

**SI-1c**

Ara*f*$_9$ derivative (**SI-1c**) has been synthesized from Ara*f*$_9$ derivative (**SI-8**) according to a general procedure for the synthesis of **SI-1c** from **SI-7**.

To a solution of 5-*O*-*t*-butyldiphenylsilyl-2,3-di-*O*-benzyl-α-D-arabinofuranosyl-(1→5)-2,3-di-*O*-benzyl-α-D-arabinofuranosyl-(1→5)-2-*O*-acetyl-3-*O*-benzyl-α-D-arabinofuranosyl-(1→5)-2,3-di-*O*-benzyl-α-D-arabinofuranosyl-(1→5)-2,3-di-*O*-benzyl-α-D-arabinofuranosyl-(1→5)-2,3-di-*O*-benzyl-α-D-arabinofuranosyl-(1→5)-2,3-di-*O*-benzyl-α-D-arabinofuranosyl-(1→5)-2,3-di-*O*-benzyl-α-D-arabinofuranosyl-(1→5)-3-*O*-benzyl-1,2-*O*-isopropylidene-β-D-arabinofuranose (**SI-8**) (8.6 g, 2.9 μmol) in MeOH (2 mL) and $H_2O$ (0.5 mL) was added $Et_3N$ (2.0 mL) at room temperature. After stirring for 24 h at the same temperature, the mixture was concentrated *in vacuo* to give a crude deacylated compound. After azeotropic removal of residual $H_2O$ with toluene, to a solution of the residue in dry DMF (1.0 mL) was added NaH (1.0 mg, 25 μmol) and BnBr (3.8 μL, 32.0 μmol) at 0°C, and the mixture was stirred for 2 h during which time temperature was going up to room temperature. The reaction was quenched by sat. $NH_4Cl$ aq., extracted with EtOAc washed with brine, dried over $Na_2SO_4$, and evaporated *in vacuo*. The residue was used without further purification. To a solution of the residue in dry THF (1.0 mL), 1 M solution of TBAF in THF (0.5 mL) was added at room temperature. After stirring for 4 h at the same temperature, the mixture was concentrated *in vacuo*. The residue was purified by PTLC (hexane/ethyl acetate =1/1) to give the title compound (**SI-1c**) (7.1 mg, 88% in three steps).
2,3-Di-*O*-benzyl-α-D-arabinofuranosyl$^{I}$-(1→5)-2,3-di-*O*-benzyl-α-D-arabinofuranosyl$^{H}$-(1→5)-2,3-di-*O*-benzyl-α-D-arabinofuranosyl$^{G}$-(1→5)-2,3-di-*O*-benzyl-α-D-arabinofuranosyl$^{F}$-(1→5)-2,3-di-*O*-benzyl-α-D-arabinofuranosyl$^{E}$-(1→5)-2,3-di-*O*-benzyl-α-D-arabinofuranosyl$^{D}$-(1→5)-2,3-di-*O*-benzyl-α-D-arabinofuranosyl$^{C}$-(1→5)-2,3-di-*O*-benzyl-α-D-arabinofuranosyl$^{B}$-(1→5)-3-*O*-benzyl-1,2-*O*-isopropylidene-β-D-arabinofuranose$^{A}$ (**SI-1c**): $[\alpha]^{26}_{D}$ 147.99° (*c* 1.00, $CHCl_3$); $^{1}$H NMR ($CDCl_3$, 400 MHz): δ 1.32 (s, 3 H, acetonide), 1.49 (s, 3 H, acetonide), 1.80 (br s, 1 H, OH), 3.52–3.70 (m, 9 H, C5$^{A–I}$-H x 9), 3.75–3.90 (m, 9 H, C5$^{A–I}$-H x 9), 3.95 (dd, *J* = 6.4, 3.2 Hz, 1 H, C3$^{I}$-H), 4.00–4.25 (m, 17 H, C2$^{B–I}$-H x8, C3$^{A–H}$-H x8, C4$^{I}$-H), 4.02–4.17 (m, 8 H, C4$^{A–H}$-H

x8), 4.35–4.60 (m, 34 H, Bn x34), 4.64 (d, $J$ = 4.0 Hz, 1 H, C2$^{A}$-H), 5.01 (s, 1 H, C1$^{I}$-H), 5.09 (s, 5 H, C1$^{B–H}$-H x5), 5.11 (s, 1 H, C1$^{B–H}$-H), 5.12 (s, 1 H, C1$^{B–H}$-H), 5.88 (d, $J$ = 4.0 Hz, 1 H, C1$^{A}$-H), 7.15–7.50 (m, 90 H, Ar); $^{13}$C NMR ($CDCl_3$, 100 MHz): δ 26.3 (Acetonide), 27.0 (Acetonide), 61.8 (C5$^{I}$), 65.6 (C5$^{B–H}$ x7), 66.6 (C5$^{A}$), 71.4 (Bn), 71.6 (Bn x2), 71.7 (Bn x4), 71.8 (Bn x2), 71.9 (Bn x2), 72.0 (Bn x4), 72.1 (Bn x2), 80.2 (C4$^{B–H}$ x7), 80.4 (C4$^{I}$), 81.9 (C3$^{I}$), 82.5 (C4$^{A}$), 82.65 (C3$^{A}$), 82.9 (C3$^{B–H}$ x5), 83.0 (C3$^{B–H}$ x2), 85.0 (C2$^{A}$), 87.5 (C2$^{I}$), 88.1 (C2$^{B–H}$ x6), 88.3 (C2$^{B–H}$ x1), 105.53 (C1$^{A}$), 106.0 (C1$^{I}$), 106.3 (C1$^{B–I}$ x7), 112.6 (Acetonide), 127.0, 127.2, 127.4, 127.5, 127.6, 127.7, 128.1, 128.2, 128.6, 137.1, 137.2, 137.3, 137.4, 137.6, 137.8 (Ar); MALDI-TOF MS: calcd for $C_{167}H_{180}Na_1O_{37}$ $[M+Na]^+$ 2800.2, found 2799.6; ESI-TOF MS: Calcd for $C_{167}H_{180}Na_2O_{37}$ $[M+Na_2]^{2+}/2$ (*m/z*) 1411.60, found 1411.90; ESI-TOF MS: Calcd for $C_{167}H_{180}Na_1O_{37}$ $[M+Na]^+$ (*m/z*) 2800.21, found 2800.79; HR ESI-TOF MS: Calcd for $C_{167}H_{180}Na_2O_{37}$ $[M+Na_2]^{2+}/2$ (*m/z*) 1411.5999, found 1411.5990; HR ESI-TOF MS: Calcd for $C_{167}H_{180}Na_1O_{37}$ $[M+Na]^+$ (*m/z*) 2800.2101, found 2800.2130.

**α-D-Arabinofuranosyl-(1→5)-α-D-arabinofuranosyl-(1→5)-α-D-arabinofuranosyl-(1→5)-α-D-arabinofuranosyl-(1→5)-α-D-arabinofuranosyl-(1→5)-α-D-arabinofuranosyl-(1→5)-α-D-arabinofuranosyl-(1→5)-α-D-arabinofuranosyl-(1→5)-1,2-*O*-isopropylidene-β-D-arabinofuranose A9LT (SI-2c).**

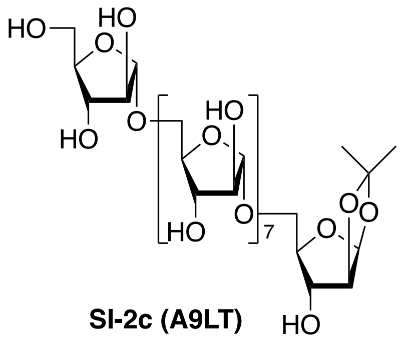


**SI-2c (A9LT)**

Hydrogenolysis of protected Ara*f*$_9$ (**SI-1c**) (14.2 mg, 5.1 μmol) was carried out in the presence of $Pd(OH)_2$ (14.2 mg) in EtOAc–MeOH–$H_2O$ (5:10:2, 8.5 mL) for 16 h at room temperature. After replacement to Ar from $H_2$ atmosphere, the mixture was filtered through a Celite pad, and the filtrate was concentrated *in vacuo* to give the title compound A9LT (**SI-2c**, 5.6 mg, 88%).

α-D-Arabinofuranosyl$^{E}$-(1→5)-α-D-arabinofuranosyl$^{D}$-(1[→5)-α-D-arabinofuranosyl$^{C}$-(1]$_5$→5)-α-D-arabinofuranosyl$^{B}$-(1→5)-1,2-*O*-isopropylidene-β-D-arabinofuranose$^{A}$ (A9LT, **SI-2c**): $[α]^{22}_{D}$ 340.00 (*c* 0.46, $H_2O$); $^{1}$H NMR ($D_2O$, 400 MHz): δ 1.34 (s, 3 H, $CH_3$), 1.55 (s, 3 H, $CH_3$), 3.63–3.71 (m, 2 H, C5$^{A}$-H, C5$^{E}$-H), 3.72–3.80 (m, 8 H, C5$^{B}$-H, C5$^{C}$-H x5, C5$^{D}$-H, C5$^{E}$-H), 3.81–3.94

(m, 10 H, $C2^{B/D}$-H, $C3^{E}$-H, $C5^{A}$-H, $C5^{B}$-H, $C5^{C}$-H x5, $C5^{D}$-H), 3.95–4.00 (m, 6 H, $C2^{B/D}$-H, $C3^{C}$-H x5), 4.04–4.12 (m, 9 H, $C2^{C}$-H x5, $C2^{E}$-H, $C3^{B}$-H, $C3^{D}$-H, $C4^{E}$-H), 4.14–4.21 (m, 7 H, $C4^{B}$-H, $C4^{C}$-H x5, $C4^{D}$-H), 4.22–4.27 (m, 2 H, $C3^{A}$-H, $C4^{A}$-H), 4.71 (d, $J$ = 4.4 Hz, 1 H, $C2^{A}$-H), 5.03 (d, $J$ = 1.6 Hz, 1 H, $C1^{E}$-H), 5.05 (s, 2 H, $C1^{B}$-H, $C1^{D}$-H), 5.06 (s, 5 H, $C1^{C}$-H x5), 6.03 (d, $J$ = 4.0 Hz, 1 H, $C1^{A}$-H); $^{13}$C NMR ($D_2O$, 100 MHz): δ 25.0 ($CH_3$), 25.7 ($CH_3$), 61.1 ($C5^{E}$), 66.7 ($C5^{B}$, $C5^{D}$), 66.8 ($C5^{C}$ x 5), 67.1 ($C5^{A}$), 74.7($C3^{A}$), 76.50 ($C3^{B/D}$), 76.54 ($C3^{B/D}$), 76.7 ($C3^{C}$ x5), 76.9 ($C3^{E}$), 80.8 (x6) ($C2^{E}$, $C2^{C}$ x5,), 80.9 ($C2^{B/D}$), 81.0 ($C2^{B/D}$), 81.7 ($C4^{B/D}$), 82.2 ($C4^{B/D}$), 82.3 ($C4^{C}$x5), 83.9 ($C4^{E}$), 85.9 ($C2^{A}$), 86.0 ($C4^{A}$), 105.5 ($C1^{A}$), 107.4 (x3), 107.5 (x5) ($C1^{B}$, $C1^{C}$ x5, $CI^{D}$, $C1^{E}$), 113.4 (acetonide); MALDI-TOF MS: calcd for $C_{48}H_{78}Na_1O_{37}$ $[M+Na]^+$ 1269.41, found 1269.99.

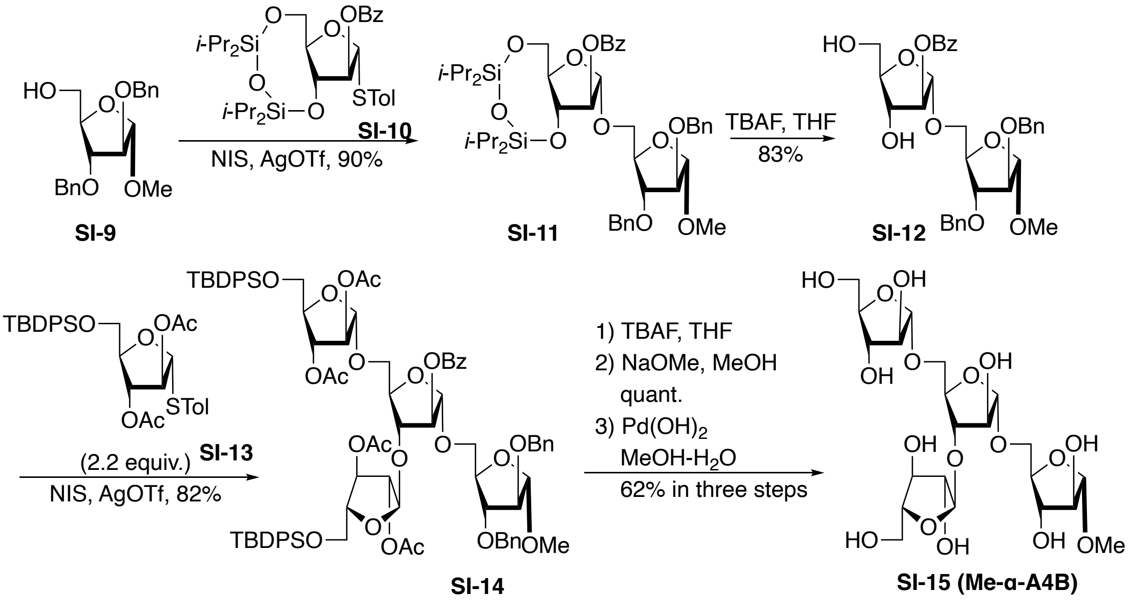


**Supplementary Figure SI-2.** Preparation of α-methyl glycoside of A4B (Me-α-A4B, **SI-15**) as the authentic for the enzymatic degradation of A5BT (**SI-4b**). Abbreviations; Bn: benzyl, Me: methyl, Tol: tolyl, *i*-Pr: isopropyl, Bz: benzoyl, NIS: *N*-iodosuccinimide, Tf: trifluoromethanesulfonyl, TBAF: tetra-(*normal*-butyl)ammonium fluoride, THF: tetrahydrofuran. TBDPS: *tert*-butyldiphenylsilyl, Bu: *normal*-butyl, Ac: acetyl.

**Methyl α-D-arabinofuranosyl-(1→5)-[α-D-arabinofuranosyl-(1→3)]-α-D-arabinofuranosyl-(1→5)-α-D-arabinofuranoside[31] Me-α-A4B (SI-15).**

To the mixture of methyl 2,3-di-*O*-benzyl-α-D-arabinofuranoside (**SI-9**)[31] (537 mg, 1.56 mmol) and tolyl 2-*O*-benzoyl-3.5-*O*-(1',1',3',3'-tetraisopropyldisiloxanilidene)-α-D-arabinofuranoside (**SI-10**)[2] (987 mg, 1.64 mmol) in dry $CH_2Cl_2$ (5 mL) was added MS 4Å (2 g, freshly dried) at

room temperature. After cooling to 0°C, NIS (608 mg, 2.70 mmol) and AgOTf (40.9 mg, 159 μmol) were added to the mixture and the mixture was stirred for 2 h at the same temperature. Then the reaction was quenched by triethylamine followed by filtration through a Celite pad and washing of the pad with ethyl acetate. The combined diluted solutions in ethyl acetate were washed with 20% aqueous $Na_2S_2O_3$, saturated aqueous $NaHCO_3$, and brine, dried over $Na_2SO_4$, and concentrated *in vacuo*. The residue was purified by $SiO_2$ column chromatography using gradient solvent system (hexane/ethyl acetate = 50 to 25 to 10 to 5) to give the methyl 2-*O*-benzoyl-3.5-*O*-(1',1',3',3'-tetraisopropyldisiloxanilidene)-α-D-arabinofuranosyl$^B$-(1→5)-di-*O*-benzyl-α-D-arabinofuranoside$^A$ **(SI-11)** (1.12 g, 90%):$^1$H NMR ($CDCl_3$, 400 MHz): δ 0.88–1.14 (m, 21 H, TIPDS), 3.35 (s, 3 H, Me), 3.59 (dd, *J* = 11.2, 5.6 Hz, 1 H, C5-H$^A$), 3.87 (dd, *J* = 11.2, 5.2 Hz, 1 H, C5-H$^A$), 3.90–4.08 (m, 5 H, C2-H$^A$, C3-H$^A$, C4-H$^B$ C5-H$_2$$^B$), 4.21 (dt, *J* = 5.6, 5.2 Hz, 1 H, C4-H$^A$), 4.44 (d, *J* = 12.0 Hz, 1 H, Bn), 4.47 (dd, *J* = 8.0, 5.2 Hz, 1 H, C3-H$^B$), 4.48 (d, *J* = 12.0 Hz, 1 H, Bn), 4.57 (s, 2 H, Bn), 4.90 (s, 1 H, C1-H$^A$), 5.05 (d, *J* = 1.2 Hz, 1 H, C1-H$^B$), 5.49 (d, *J* = 5.2, 1.2 Hz, 1 H, C2-H$^B$) 7.22–8.04 (m, 15 H, Ar); $^{13}$C NMR ($CDCl_3$, 100 MHz): δ 12.5 (TIPDS), 12.8 (TIPDS), 13.1 (TIPDS), 13.4 (TIPDS), 16.86 (TIPDS), 16.90 (TIPDS), 17.0 (TIPDS), 17.31 (TIPDS), 17.39 (TIPDS), 17.44 (TIPDS), 54.9 (OMe), 61.6 (C5$^B$), 67.5 (C5$^A$), 71.8 (Bn), 72.1 (Bn), 76.1 (C3$^B$), 80.8 (C4$^A$), 81.1 (C4$^B$), 83.6 (C2 $^A$/3$^A$), 84.1 (C2$^B$), 88.0 (C2 $^A$/3$^A$), 105.5 (C1$^B$), 107.2 (C1$^A$), 127.67 (Ar), 127.73 (Ar), 127.8 (Ar), 127.9 (Ar), 128.4 (Ar), 129.7 (Ar), 133.2 (Ar), 137.6 (Ar), 137.9 (Ar), 165.4 (Bz); MALDI-TOF MS: calcd for $C_{44}H_{62}Na_1O_{11}Si_2$ $[M+Na]^+$ 845.37, found 845.58. To the mixture of Ara*f*$_2$ derivative (**SI-11**) (1.12 g, 1.36 mmol) in dry THF (5 mL) was added TBAF in THF (1 M, 3.39 mL, 3.39 mmol) at 0°C and the mixture was stirred for 2 h at the same temperature. After quenching with AcOH (2 mL), the mixture was concentrated *in vacuo* and the residue was purified by $SiO_2$ column chromatography using gradient solvent system (toluene/acetone = 50 to 25 to 10 to 5 to 2 to 1) to give the methyl 2-*O*-benzoyl-α-D-arabinofuranosyl$^B$-(1→5)-di-*O*-benzyl-α-D-arabinofuranoside$^A$ **(SI-12)** (651 mg, 83%):$^1$H NMR ($CDCl_3$, 400 MHz): δ 3.36 (s, 3 H, Me), 3.70 (dd, *J* = 10.8, 3.6 Hz, 1 H, C5-H$^A$), 3.71 (dd, *J* = 12.4, 4.4 Hz, 1 H C5-H$^B$), 3.83 (dd, *J* = 12.4, 3.6 Hz, 1 H, C5-H$^B$), 3.86 (dd, *J* = 10.8, 5.2 Hz, 1 H, C5-H$^A$), 3.89 (dd, *J* = 6.0, 2.4 Hz, 1 H, C3-H$^A$), 3.97 (d, *J* = 2.4 Hz, 1 H, C2-H$^A$), 4.03 (ddd, *J* = 6.0, 5.2, 3.2 Hz, 1 H, C4-H$^B$), 4.12 (dd, *J* = 6.0, 2.4 Hz, 1 H, C3-H$^B$), 4.19 (ddd, *J* = 6.0, 4.8, 3.6 Hz, 1 H, C4-H$^A$), 4.44 (d, *J* = 12.0 Hz, 1 H, Bn), 4.46 (d, *J* = 12.4 Hz, 1 H, Bn), 4.54 (d, *J* = 12.0 Hz, 1 H, Bn), 4.57 (d, *J* = 12.4 Hz, 1 H, Bn), 4.93 (s, 1 H, C1-H$^A$), 5.13 (d, *J* = 2.4 Hz, 1 H, C2-H$^B$), 5.26 (s, 1 H, C1-H$^B$), 7.25–8.00 (m, 15 H, Ar); $^{13}$C NMR ($CDCl_3$, 100 MHz): δ 54.9 (OMe), 62.0 (C5$^B$), 66.2 (C5$^A$), 71.9 (Bn), 72.1 (Bn), 76.2 (C3$^B$), 80.7 (C4$^A$), 83.3 (C3$^A$), 84.9 (C4$^B$), 85.0 (C2$^B$), 87.4 (C2$^A$), 105.0 (C1$^B$), 107.2 (C1$^A$), 127.83 (Ar), 127.86 (Ar), 127.95 (Ar), 128.01 (Ar), 128.41 (Ar), 128.44 (Ar), 128.48 (Ar), 129.0 (Ar), 129.7 (Ar), 133.5 (Ar), 137.2 (Ar), 137.5 (Ar), 166.3 (Bz); MALDI-TOF MS: calcd for $C_{32}H_{36}Na_1O_{10}$

$[M+Na]^+$ 603.22, found 603.63. To the mixture of Ara*f*$_2$ diol acceptor (**SI-12**) (149 mg, 257 μmol) and tolyl 2,3-di-*O*-acetyl-5-*O*-*t*-butyldiphenylsilyl-1-deoxy-1-thio-α-D-arabinofuranoside[32] (**SI-13**) (327 mg, 566 μmol) in dry $CH_2Cl_2$ (5 mL) was added MS 4Å (2 g, freshly dried) at room temperature. After cooling to 0°C, NIS (201 mg, 892 μmol) and AgOTf (13.4 mg, 52.2 μmol) were added to the mixture and the mixture was stirred for 4 h at the same temperature. Then the reaction was quenched with triethylamine followed by filtration through a Celite pad and washing of the pad with ethyl acetate. The combined diluted solutions in ethyl acetate were washed with 20% aqueous $Na_2S_2O_3$, saturated aqueous $NaHCO_3$, and brine, dried over $Na_2SO_4$, and concentrated *in vacuo*. The residue was purified by $SiO_2$ column chromatography using gradient solvent system (toluene/acetone = 50 to 25 to 10 to 5 to 2 to 1) to give the 2,3-di-*O*-acetyl-5-*O*-*t*-butyldiphenylsilyl-α-D-arabinofuranosyl$^D$-(1→5)-[2,3-di-*O*-acetyl-5-*O*-*t*-butyldiphenylsilyl-α-D-arabinofuranosyl$^C$-(1→3)]-2-*O*-benzoyl-α-D-arabinofuranosyl$^B$-(1→5)-di-*O*-benzyl-α-D-arabinofuranoside$^A$ (**SI-14**) (132 mg, 86%): $^1$H NMR ($CDCl_3$, 400 MHz): δ 0.99 (s, 9 H, *t*-Bu), 1.01 (s, 9 H, *t*-Bu), 1.77 (s, 3 H, Ac), 1.94 (s, 9 H, Ac x3), 3.35 (s, 3 H, Me), 3.64 (dd, *J* = 11.2, 4.8 Hz, 1 H, C5-H$^A$), 3.69 (dd, *J* = 11.6, 2.4 Hz, 1 H, C5-H$^B$), 3.72–3.82 (m, 4 H, C5-$H_2{}^C$, C5-$H_2{}^D$), 3.86 (dd, *J* = 11.6, 4.8 Hz, 1 H, C5-H$^B$), 3.86 (dd, *J* = 11.2, 4.8 Hz, 1 H, C5-H$^A$), 3.96 (dd, *J* = 3.2, 0.8 Hz, 1 H, C2-H$^A$), 3.99 (dd, *J* = 6.0, 3.2 Hz, 1 H, C3-H$^A$), 4.01–4.06 (m, 2 H, C4-H$^C$, C4-H$^D$), 4.19 (ddd, *J* = 6.0, 5.2, 4.4 Hz, 1 H, C4-H$^A$), 4.24 (ddd, *J* = 6.4, 4.0, 2.4 Hz, 1 H, C4-H$^B$), 4.29 (d, *J* = 6.4 Hz, 1 H, C3-H$^B$), 4.44 (d, *J* = 12.0 Hz, 1 H, Bn), 4.50 (d, *J* = 12.0 Hz, 1 H, Bn), 4.58 (s, 2 H, Bn), 4.90 (s, 1 H, C1-H$^A$), 5.08 (d, *J* = 1.6 Hz, 1 H, C2-H$^D$), 5.10 (s, 1 H, C1-H$^D$), 5.15 (d, *J* = 1.6 Hz, 1 H, C2-H$^C$), 5.21 (d, *J* = 6.0 Hz, 1 H, C3-H$^D$), 5.21–5.24 (m, 1 H, C3-H$^C$), 5.23 (s, 1 H, C1-H$^B$), 5.38 (s, 2 H, C1-H$^C$, C2-H$^B$), 7.25–8.05 (m, 25 H, Ar); $^{13}$C NMR ($CDCl_3$, 100 MHz): δ 19.22 (TBDPS), 19.27 (TBDPS), 20.47 (TBDPS), 20.66 (TBDPS), 26.65 (TBDPS), 55.0 (OMe), 62.79 (C5$^{C/D}$), 62.84 (C5$^{C/D}$), 65.4 (C5$^B$), 66.3 (C5$^A$), 71.8 (Bn), 72.2 (Bn), 76.8 (C3$^C$,C3$^D$), 80.2 (C3$^B$), 80.5 (C4$^A$), 81.6 (C4$^B$), 81.7 (C2$^D$), 81.9 (C2$^C$), 82.3 (C2$^B$), 83.2 (C4$^C$,C4$^D$), 83.3 (C3$^A$), 87.7 (C2$^A$), 105.0 (C1$^C$), 105.6 (C1$^D$), 105.9 (C1$^B$), 107.2 (C1$^A$), 127.61 (Ar), 127.63 (Ar), 127.73 (Ar), 127.77 (Ar), 127.88 (Ar), 127.92 (Ar), 128.4 (Ar), 128.5 (Ar), 129.4 (Ar), 129.6 (Ar), 129.7 (Ar), 129.8 (Ar), 133.0 (Ar), 133.12 (Ar), 133,14 (Ar), 133.2 (Ar), 133.4 (Ar), 135.58 (Ar), 135.60 (Ar), 137.5 (Ar), 137.9 (Ar), 165.3 (Bz), 169.6 (Ac x2), 170.0 (Ac), 170.1 (Ac); HMBC: irradiated 5.23 (C1-H$^B$) → enhanced 66.3 (C5$^A$); irr. 5.10 (C1-H$^D$) → enhanced 65.4 (C5$^B$); irr. 4.90 (C1-H$^A$) → enhanced 55.0 (OMe); irr. 4.29 (C3-H$^B$) → enhanced 105.0 (C1$^C$). MALDI-TOF MS: calcd for $C_{82}H_{96}Na_1O_{22}Si_2$ $[M+Na]^+$ 1511.58 found 1511.75. To the solution of **SI-14** (105 mg, 70.5 μmol) in dry THF (2 mL) was added TBAF in THF (1 M, 353 μL, 353 μmol) at room temperature and the mixture was stirred for 12 h at the same temperature. Then the mixture was concentrated *in vacuo*. The residue was used without further purification. To a solution of the residue in MeOH (5 mL), 72% solution NaOMe in MeOH (20 μL) was added at

room temperature and the mixture was stirred for 2 h at the same temperature. After addition of Amberlyst® 15(H) resin followed by filtration through Celite pad, the filtrate was then evaporated *in vacuo* and the residue was purified by PTLC ($SiO_2$, $CHCl_3$/MeOH = 10/1) to afford the methyl α-D-arabinofuranosyl$^D$-(1→5)-[α-D-arabinofuranosyl$^C$-(1→3)]-α-D-arabinofuranosyl$^B$-(1→5)-di-*O*-benzyl-α-D-arabinofuranoside$^A$ (54.0 mg, quant.): $^1$H NMR ($CD_3OD$, 400 MHz): δ 3.35 (s, 3 H, Me), 3.61 (dd, *J* = 12.0, 4.0 Hz, 1 H, C5-H$^A$), 3.63 (dd, *J* = 12.0, 4.0 Hz, 1 H, C5-H$^{C/D}$), 3.64 (dd, *J* = 12.0, 3.6 Hz, 1 H, C5-H$^{C/D}$),3.80 (dd, *J* = 12.0, 3.2 Hz, 1 H, C5-H$^B$), 3.75 (dd, *J* = 12.0, 4.0 Hz, 1 H, C5-H$^{C/D}$), 3.76 (dd, *J* = 12.0, 3.6 Hz, 1 H, C5-H$^{C/D}$), 3.80 (dd, *J* = 12.0, 5.2 Hz, 1 H, C5-H$^A$), 3.82–3.86 (m, 2 H, C3-H$^C$, C3-H$^D$), 3.92 (dd, *J* = 12.0, 4.8 Hz, 1 H, C5-H$^B$), 3.92–3.96 (m, 2 H, C2-H$^A$, C4-H$^{C/D}$), 3.98 (dd, *J* = 6.4, 3.2 Hz, 1 H, C3-H$^A$), 3.92–3.96 (m, 1 H, C4-H$^{C/D}$), 4.09 (dd, *J* = 3.6, 0.8 Hz, 1 H, C2-H$^D$), 4.09 (dd, *J* = 4.0, 2.0 Hz, 1 H, C2-H$^C$), 4.06–4.11 (m, 1 H, C4-H$^A$), 4.09 (d, *J* = 2.8 Hz, 1 H, C3-H$^B$), 4.12–4.16 (m, 1 H, C4-H$^B$), 4.16 (dd, *J* = 2.4, 0.8 Hz, 1 H, C2-H$^B$), 4.48 (d, *J* = 12.4 Hz, 1 H, Bn), 4.54 (s, 2 H, Bn), 4.57 (d, *J* = 12.4 Hz, 1 H, Bn), 4.89 (s, 1 H, C1-H$^A$), 4.90 (d, *J* = 0.8 Hz, 1 H, C1-H$^B$), 4.96 (d, *J* = 0.8 Hz, 1 H, C1-H$^D$), 5.07 (d, *J* = 2.0 Hz, 1 H, C1-H$^C$), 7.26–7.36 (m, 10 H, Ar); $^{13}$C NMR ($CD_3OD$, 100 MHz): δ 55.1 (OMe), 62.9 (C5$^{C/D}$), 63.1 (C5$^{C/D}$), 67.47 (C5$^B$), 67.51 (C5$^A$), 72.9 (Bn), 73.3 (Bn), 78.6 (C3$^{C/D}$), 78.8 (C3$^{C/D}$), 81.8 (C2$^B$,C3$^B$), 82.8 (C2$^{C/D}$), 83.0 (C2$^{C/D}$), 83.6 (C4$^B$), 84.1 (C4$^A$), 84.6 (C3$^A$), 85.4 (C4$^{C/D}$), 85.8 (C4$^{C/D}$), 89.1 (C2$^A$), 108.5 (C1$^A$), 109.0 (C1$^C$), 109.4 (C1$^D$), 109.6 (C1$^B$), 128.8 (Ar), 129.0 (Ar), 129.1 (Ar), 129.3 (Ar), 129.4 (Ar), 129.5 (Ar), 138.9 (Ar), 139.3 (Ar); HMBC: irradiated 4.90 (C1-H$^B$) → enhanced 67.51 (C5$^A$); irr. 4.96 (C1-H$^D$) → enhanced 67.47 (C5$^B$); irr. 4.89 (C1-H$^A$) → enhanced 55.1 (OMe); irr. 4.09 (C3-H$^B$) → enhanced 109.0 (C1$^C$); MALDI-TOF MS: calcd for $C_{35}H_{48}Na_1O_{17}$ $[M+Na]^+$ 763.74, found 763.42. Hydrogenolysis of the resultant dibenzyl ether (30.0 mg, 39.3 μmol) was carried out in the presence of $Pd(OH)_2$ (30.0 mg) in MeOH–$H_2O$ (1:1, 4.0 mL) for 18 h at room temperature. After replacement to Ar from $H_2$ atmosphere, the mixture was filtered through a Celite pad and filtrate was concentrated *in vacuo* and the residue was purified by PTLC ($SiO_2$, $CHCl_3$/MeOH = 4/1) to afford the title compound Me-α-A4B (**SI-15**, 14.2 mg, 62%).

Methyl α-D-arabinofuranosyl$^D$-(1→5)-[α-D-arabinofuranosyl$^C$-(1→3)]-α-D-arabinofuranosyl$^B$-(1→5)-α-D-arabinofuranoside$^A$ (Me-α-A4B, **SI-15**)[2]: $^1$H NMR ($D_2O$, 400 MHz): δ 3.42 (s, 3 H, Me), 3.708 (dd, *J* = 12.8, 6.0 Hz, 1 H, C5-H$^C$), 3.712 (dd, *J* = 12.4, 5.6 Hz, 1 H, C5-H$^D$), 3.78 (dd, *J* = 12.0, 3.2 Hz, 1 H, C5-H$^A$), 3.80–3.85 (m, 2 H, C5-H$^C$, C5-H$^D$), 3.84 (dd, *J* = 12.0, 3.2 Hz, 1 H, C5-H$^B$), 3.88 (dd, *J* = 12.0, 5.2 Hz, 1 H, C5-H$^A$), 3.94 (dd, *J* = 6.4, 3.2 Hz, 1 H, C3-H$^C$), 3.95 (dd, *J* = 12.0, 5.6 Hz, 1 H, C5-H$^B$), 3.96 (dd, *J* = 5.6, 2.8 Hz, 1 H, C3-H$^D$), 4.00–4.06 (m, 1 H, C4-H$^C$), 4.03 (dd, *J* = 6.0, 3.2 Hz, 1 H, C3-H$^A$), 4.05 (dd, *J* = 3.2, 1.6 Hz, 1 H, C2-H$^A$), 4.07–4.12 (m, 1 H, C4-H$^D$), 4.10 (dd, *J* = 6.0, 3.6 Hz, 1 H, C3-H$^B$), 4.12–4.13 (m, 1 H, C2-H$^D$), 4.13–4.14 (m, 1 H, C2-H$^C$), 4.16 (ddd, *J* = 6.0, 5.2, 3.2 Hz, 1 H, C4-H$^A$), 4.29 (brs, 1 H, C2-H$^B$), 4.31 (dd, *J*

= 6.0, 5.6, 3.2 Hz, 1 H, C4-$H^B$), 4.93 (d, $J$ = 1.6 Hz, 1 H, C1-$H^A$), 5.09 (d, $J$ = 1.6 Hz, 1 H, C1-$H^D$), 5.11 (s, 1 H, C1-$H^B$), 5.15 (d, $J$ = 0.8 Hz, 1 H, C1-$H^C$); $^{13}C$ NMR ($D_2O$, 100 MHz): δ 55.6 (OMe), 61.7 ($C5^D$), 61.8 ($C5^C$), 67.0 ($C5^B$, $C5^A$), 77.0 ($C3^A$), 77.2 ($C3^C$, $C3^D$), 79.7 ($C2^B$), 81.3 ($C2^A$), 81.6 ($C2^C$), 81.9 ($C2^D$), 82.3 ($C4^B$), 82.7 ($C4^A$), 83.0 ($C3^B$), 84.5 ($C4^C$), 84.6 ($C4^D$), 107.8 ($C1^D$), 108.0 ($C1^C$), 108.1 ($C1^B$), 109.0 ($C1^A$); HMBC: irradiated 5.11 (C1-$H^B$) → enhanced 67.0 ($C5^A$); irr. 5.09 (C1-$H^D$) → enhanced 67.0 ($C5^B$); irr. 4.93 (C1-$H^A$) → enhanced 55.6 (OMe); irr. 4.10 (C3-$H^B$) → enhanced 108.1 ($C1^C$); irr. 3.42 (Me) → enhanced 109.0 ($C1^C$) (Supplementary Table SI-1); MALDI-TOF MS: calcd for $C_{21}H_{36}Na_1O_{17}$ $[M+Na]^+$ 583.19, found 583.18.

**Supplementary Table SI-1.** Methyl α-D-arabinofuranosyl$^{\mathrm{D}}$-(1→5)-[α-D-arabinofuranosyl$^{\mathrm{C}}$-(1→3)]-α-D-arabinofuranosyl$^{\mathrm{B}}$-(1→5)-α-D-arabinofuranoside$^{\mathrm{A}}$ (Me-α-A4B, **SI-15**)

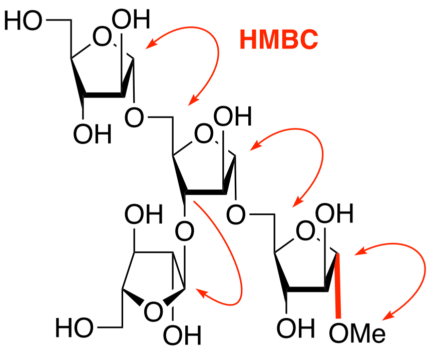


| in $D_2O$, r.t. | | $^{1}$H/ δ ppm | mult., $^{3}J_{\mathrm{H-H}}$ Hz | $^{13}$C/ δ ppm | $^{1}J_{\mathrm{C1-H}}$ | HMBC |
|---|---|---|---|---|---|---|
| α-D-Ara*f*$^{\mathrm{D}}$(1→5) | 1 | 5.09 | d, 1.6 | 107.8 | 180.7 | → 67.0 |
| | 2 | 4.13 | m | 81.9 | | |
| | 3 | 3.96 | dd, 5.6, 2.8 | 77.2 | | |
| | 4 | 4.10 | m | 84.5 | | |
| | 5 | 3.83 | dd, 12.4, 3.2 | 61.7 | | |
| | | 3.712 | dd, 12.4, 5.6 | | | |
| α-D-Ara*f*$^{\mathrm{C}}$(1→3) | 1 | 5.15 | d, 0.8 | 108.1 | 177.8 | |
| | 2 | 4.14 | m | 81.6 | | |
| | 3 | 3.94 | dd, 6.4, 3.2 | 77.2 | | |
| | 4 | 4.03 | m | 84.6 | | |
| | 5 | 3.83 | dd, 12.8, 3.2 | 61.8 | | |
| | | 3.708 | dd, 12.8, 6.0 | | | |
| (→3,5)- | 1 | 5.11 | s | 108.1 | 179.0 | → 67.0 |
| α-D-Ara*f*$^{\mathrm{B}}$(1→ | 2 | 4.29 | brs | 79.7 | | |
| | 3 | 4.10 | dd, 6.0, 3.6 | 83.0 | | →108.1 |
| | 4 | 4.31 | ddd, 6.0, 5.6, 3.2 | 82.3 | | |
| | 5 | 3.95 | dd, 12.0, 5.6 | 67.0 | | |
| | | 3.84 | dd, 12.0, 3.2 | | | |
| →5)- α-D-Ara*f*$^{\mathrm{A}}$- | 1 | 4.93 | d, 1.6 | 109.0 | 180.7 | → 55.6 |
| | 2 | 4.05 | d, 3.2, 1.6 | 81.3 | | |
| | 3 | 4.03 | d, 6.0, 3.2 | 77.0 | | |
| | 4 | 4.16 | ddd, 6.0, 5.2, 3.2 | 82.7 | | |
| | 5 | 3.88 | dd, 12.0, 5.2 | 67.0 | | |
| | | 3.78 | dd, 12.0, 3.2 | | | |
| OMe | | 3.42 | s | 55.6 | | →109.0 |

For the standard of $^{1}$H, the HOD peak was used at δ 4.80 ppm, and for $^{13}$C NMR, data were processed using a native scale.

Enzymatic cleavage of a branched pentasaccharide acetonide A5BT (**SI-4b**) in the presence of MeOH afforded the corresponding branched methyl tetrasaccharide Me-α-A4B (**SI-15**).
Methyl α-D-arabinofuranosyl$^{D}$-(1→5)-[α-D-arabinofuranosyl$^{C}$-(1→3)]-α-D-arabinofuranosyl$^{B}$-(1→5)-α-D-arabinofuranoside$^{A}$ Me-α-A4B (**SI-15**): $^{1}$H NMR ($D_2O$, 400 MHz): δ 3.42 (s, 3 H, Me), 3.706 (dd, *J* = 12.0, 5.2 Hz, 1 H, C5-H$^{C}$), 3.707 (dd, *J* = 12.4, 6.0 Hz, 1 H, C5-H$^{D}$), 3.78 (dd, *J* = 12.0 3.2 Hz, 1 H, C5-H$^{A}$), 3.80–3.85 (m, 3 H, C5-H$^{C}$, C5-H$^{D}$, C5-H$^{B}$), 3.88 (dd, *J* = 12.0, 6.0 Hz, 1 H, C5-H$^{A}$), 3.92–3.96 (m, 3 H, C3-H$^{C}$, C5-H$^{B}$, C3-H$^{D}$), 4.00–4.06 (m, 2 H, C4-H$^{C}$, C3-H$^{A}$), 4.04–4.06 (m, 1 H, C2-H$^{A}$), 4.07–4.12 (m, 1 H, C4-H$^{D}$), 4.11 (dd, *J* = 6.0, 3.2 Hz, 1 H, C3-H$^{B}$), 4.12–4.14 (m, 2 H, C2-H$^{D}$, C2-H$^{C}$), 4.16 (ddd, *J* = 6.0, 3.2, 1,6 Hz, 1 H, C4-H$^{A}$), 4.29 (dd, *J* = 2.4, 0.8 Hz, 1 H, C2-H$^{B}$), 4.31 (dd, *J* = 6.0, 5.6, 3.2 Hz, 1 H, C4-H$^{B}$), 4.92 (s 1 H, C1-H$^{A}$), 5.08 (s, 1 H, C1-H$^{D}$), 5.11 (s, 1 H, C1-H$^{B}$), 5.15 (s, 1 H, C1-H$^{C}$).

The reason why we resynthesized the known compound Me-α-A4B (**SI-15**)[2] is that the degraded product with MeOH does not match the reported $^{1}$H NMR peaks at some anomeric positions and we wanted to make sure. We revised them by completing the synthesis of the authentic Me-α-A4B (**SI-15**) and could confirm that the degraded product is the α-methyl glycoside of tetrasaccharide A4B.

## Supplementary References


1. Kashima, T. *et al.* Identification of a difructose dianhydride I synthase/hydrolase from oral bacterium establishes a novel glycoside hydrolase family. *Journal of Biological Chemistry* **297**, 101324 (2021).
2. D'Souza, F. W., Ayers, J. D., McCarren, P. R. & Lowary, T. L. Arabinofuranosyl oligosaccharides from mycobacteria: Synthesis and effect of glycosylation on ring conformation and hydroxymethyl group rotamer populations. *J Am Chem Soc* **122**, 1251–1260 (2000).
3. Rahfeld, P. *et al.* An enzymatic pathway in the human gut microbiome that converts A to universal O type blood. *Nature Microbiology 2019 4:9* **4**, 1475–1485 (2019).
4. Till, M. *et al.* Structural analysis of the GH43 enzyme Xsa43E from *Butyrivibrio proteoclasticus*. *Acta Crystallogr Section F Struct Biol Commun* **70**, 1193–1198 (2014).
5. Cartmell, A. *et al.* The structure and function of an arabinan-specific α-1,2-arabinofuranosidase identified from screening the activities of bacterial GH43 glycoside hydrolases. *Journal of Biological Chemistry* **286**, 15483–15495 (2011).
6. von Schantz, L. *et al.* Structural basis for carbohydrate-binding specificity—A comparative assessment of two engineered carbohydrate-binding modules. *Glycobiology* **22**, 948–961 (2012).
7. Fujimoto, Z. *et al.* Carbohydrate-binding architecture of the multi-modular α-1,6-glucosyltransferase from *Paenibacillus* sp. 598K, which produces α-1,6-glucosyl-α-glucosaccharides from starch. *Biochemical Journal* **474**, 2763–2778 (2017).
8. Suzuki, R. *et al.* Crystallographic and mutational analyses of substrate recognition of *endo*-alpha-*N*-acetylgalactosaminidase from *Bifidobacterium longum*. *J Biochem* **146**, 389–98 (2009).
9. Kabsch, W. *XDS*. *Acta Crystallogr D Biol Crystallogr* **66**, 125–132 (2010).
10. Evans, P. R. & Murshudov, G. N. How good are my data and what is the resolution? *Acta Crystallogr D Biol Crystallogr* **69**, 1204–1214 (2013).
11. Adams, P. D. *et al.* PHENIX: A comprehensive Python-based system for macromolecular structure solution. *Acta Crystallogr D Biol Crystallogr* **66**, 213–221 (2010).
12. Vagin, A. & Lebedev, A. MoRDa , an automatic molecular replacement pipeline. *Acta Crystallogr A Found Adv* **71**, s19–s19 (2015).
13. Emsley, P., Lohkamp, B., Scott, W. G. & Cowtan, K. Features and development of Coot. *Acta Crystallogr D Biol Crystallogr* **66**, 486–501 (2010).
14. Murshudov, G. N. *et al.* REFMAC5 for the refinement of macromolecular crystal structures. *Acta Crystallogr D Biol Crystallogr* **67**, 355–367 (2011).

15. Liebschner, D. *et al.* Polder maps: Improving OMIT maps by excluding bulk solvent. *Acta Crystallogr D Struct Biol* **73**, 148–157 (2017).
16. Krissinel, E. & Henrick, K. Inference of macromolecular assemblies from crystalline state. *J Mol Biol* **372**, 774–797 (2007).
17. Holm, L. & Laakso, L. M. Dali server update. *Nucleic Acids Res* **44**, W351–W355 (2016).
18. Bernadó, P., Shimizu, N., Zaccai, G., Kamikubo, H. & Sugiyama, M. Solution scattering approaches to dynamical ordering in biomolecular systems. *Biochimica et Biophysica Acta (BBA) - General Subjects* **1862**, 253–274 (2018).
19. Shimizu, N. *et al.* BL-10C, the small-angle x-ray scattering beamline at the photon factory. *AIP Conf Proc* **2054**, 060041 (2019).
20. Shimizu, N. *et al.* Software development for analysis of small-angle x-ray scattering data. *AIP Conf Proc* **1741**, 050017 (2016).
21. Yonezawa, K., Takahashi, M., Yatabe, K., Nagatani, Y. & Shimizu, N. MOLASS: Software for automatic processing of matrix data obtained from small-angle X-ray scattering and UV–visible spectroscopy combined with size-exclusion chromatography. *Biophys Physicobiol* **20**, e200001 (2023).
22. Petoukhov, M. V., Konarev, P. V., Kikhney, A. G. & Svergun, D. I. ATSAS 2.1 – towards automated and web-supported small-angle scattering data analysis. *J Appl Crystallogr* **40**, s223–s228 (2007).
23. Svergun, D. I. Determination of the regularization parameter in indirect-transform methods using perceptual criteria. *J Appl Cystallogr* **25**, 495–503 (1992).
24. Manalastas-Cantos, K. *et al.* ATSAS 3.0: expanded functionality and new tools for small-angle scattering data analysis. *J Appl Crystallogr* **54**, 343–355 (2021).
25. Franke, D. *et al.* ATSAS 2.8: a comprehensive data analysis suite for small-angle scattering from macromolecular solutions. *J Appl Crystallogr* **50**, 1212–1225 (2017).
26. Kozin, M. B. & Svergun, D. I. Automated matching of high- and low-resolution structural models. *J Appl Crystallogr* **34**, 33–41 (2001).
27. Pettersen, E. F. F. *et al.* UCSF Chimera - A visualization system for exploratory research and analysis. *J Comput Chem* **25**, 1605–1612 (2004).
28. Kaeothip, S. *et al.* Preparation of *p*-nitrophenyl β-L-arabinofuranoside as a substrate of β-L-arabinofuranosidase. *Carbohydr Res* **382**, 95–100 (2013).
29. Désiré, J. & Prandi, J. Synthesis of methyl β-d-arabinofuranoside 5-[1d (and 1)-myo-inositol 1-phosphate], the capping motif of the lipoarabinomannan of *Mycobacterium smegmatis*. *Carbohydr Res* **317**, 110–118 (1999).
30. Ishiwata, A. & Ito, Y. Synthesis of docosasaccharide arabinan motif of mycobacterial cell wall. *J Am Chem Soc* **133**, 2275–2291 (2011).

31. Yin, H., D'Souza, F. W. & Lowary, T. L. Arabinofuranosides from mycobacteria: Synthesis of a highly branched hexasaccharide and related fragments containing β-arabinofuranosyl residues. *Journal of Organic Chemistry* **67**, 892–903 (2002).
32. Ishiwata, A., Akao, H. & Ito, Y. Stereoselective synthesis of a fragment of mycobacterial arabinan. *Org Lett* **8**, 5525–5528 (2006).
