# Supplementary material for: Identification and characterization of endo-α-, exo-α-, and exo-β-d-arabinofuranosidases degrading lipoarabinomannan and arabinogalactan of mycobacteria
Source: Nat Commun. 2023 Sep 19;14:5803. doi: 10.1038/s41467-023-41431-2 (PMC10509167; doi:10.1038/s41467-023-41431-2)
Supplement: Supplementary file 2 — Reporting Summary [file 41467_2023_41431_MOESM2_ESM.pdf]

Corresponding author(s): Shinya Fushinobu
Last updated by author(s): Aug 6, 2023

Reporting Summary

Nature Portfolio wishes to improve the reproducibility of the work that we publish. This form provides structure for consistency and transparency in reporting. For further information on Nature Portfolio policies, see our Editorial Policies and the Editorial Policy Checklist.

Statistics

For all statistical analyses, confirm that the following items are present in the figure legend, table legend, main text, or Methods section.

n/a	Confirmed	
☐	☒	The exact sample size (*n*) for each experimental group/condition, given as a discrete number and unit of measurement
☐	☒	A statement on whether measurements were taken from distinct samples or whether the same sample was measured repeatedly
☒	☐	The statistical test(s) used AND whether they are one- or two-sided<br>*Only common tests should be described solely by name; describe more complex techniques in the Methods section.*
☒	☐	A description of all covariates tested
☒	☐	A description of any assumptions or corrections, such as tests of normality and adjustment for multiple comparisons
☐	☒	A full description of the statistical parameters including central tendency (e.g. means) or other basic estimates (e.g. regression coefficient) AND variation (e.g. standard deviation) or associated estimates of uncertainty (e.g. confidence intervals)
☒	☐	For null hypothesis testing, the test statistic (e.g. *F*, *t*, *r*) with confidence intervals, effect sizes, degrees of freedom and *P* value noted<br>*Give P values as exact values whenever suitable.*
☒	☐	For Bayesian analysis, information on the choice of priors and Markov chain Monte Carlo settings
☒	☐	For hierarchical and complex designs, identification of the appropriate level for tests and full reporting of outcomes
☒	☐	Estimates of effect sizes (e.g. Cohen's *d*, Pearson's *r*), indicating how they were calculated

Our web collection on statistics for biologists contains articles on many of the points above.

Software and code

Policy information about availability of computer code

Data collection	No custom software or code was used.
Data analysis	No custom software or code was used.

For manuscripts utilizing custom algorithms or software that are central to the research but not yet described in published literature, software must be made available to editors and reviewers. We strongly encourage code deposition in a community repository (e.g. GitHub). See the Nature Portfolio guidelines for submitting code & software for further information.

Data

Policy information about availability of data

All manuscripts must include a data availability statement. This statement should provide the following information, where applicable:

- Accession codes, unique identifiers, or web links for publicly available datasets
- A description of any restrictions on data availability
- For clinical datasets or third party data, please ensure that the statement adheres to our policy

Atomic coordinates and structural factors of EndoMA1 apo, EndoMA1 + A9LT, ExoMA1, ExoMA2 + Tris, and ExoMA2 + β-D-Araf have been deposited in the PDB under accession numbers 8HHV [http://doi.org/10.2210/pdb8HHV/pdb], 8IC1 [http://doi.org/10.2210/pdb8IC1/pdb], 8IC8 [http://doi.org/10.2210/pdb8IC8/pdb], 8IC6 [http://doi.org/10.2210/pdb8IC6/pdb], and 8IC7 [http://doi.org/10.2210/pdb8IC7/pdb], respectively. The draft genome of Mi. arabinogalactanolyticum JCM 9171 has been deposited to DDBJ under accession number SAMD00521460 (BioProject accession number, PRJDB14210; raw sequence reads in the DRA accession

number, DRA0147510). Sequence Read Archive is publicly available: DRR401167 [https://www.ncbi.nlm.nih.gov/sra/DRR401167]. The 13 contig sequences are publicly available on GenBank at NCBI with IDs BRZC01000001-BRZC01000013. Source data are provided with this paper.

Research involving human participants, their data, or biological material

Policy information about studies with human participants or human data. See also policy information about sex, gender (identity/presentation), and sexual orientation and race, ethnicity and racism.

Reporting on sex and gender	No human research participants.
Reporting on race, ethnicity, or other socially relevant groupings	No human research participants.
Population characteristics	No human research participants.
Recruitment	No human research participants.
Ethics oversight	No human research participants.

Note that full information on the approval of the study protocol must also be provided in the manuscript.

Field-specific reporting

Please select the one below that is the best fit for your research. If you are not sure, read the appropriate sections before making your selection.

☒ Life sciences ☐ Behavioural & social sciences ☐ Ecological, evolutionary & environmental sciences

For a reference copy of the document with all sections, see nature.com/documents/nr-reporting-summary-flat.pdf

Life sciences study design

All studies must disclose on these points even when the disclosure is negative.

Sample size	All data points in the kinetic analysis are shown in the plots. The sample size was chosen so that the kinetic data could be correctly fitted to the expected equations, such as the Michaelis equation, and sufficiently small fitting errors were calculated. All statistics in the crystallographic and SAXS analyses are shown in the tables.
Data exclusions	No data was excluded.
Replication	The activity measurements were always performed multiple times and the results are shown after examining the conditions under which similar data can be obtained repeatedly. Most crystallization data are under conditions that yielded similar crystals, and are shown for which the best resolution was obtained after multiple measurements. Although EndoMA1-A9LT was crystallized only once, crystallographic statistical analysis confirmed the validity of the data from the single crystal.
Randomization	No grouping was performed in the experiments.
Blinding	Blinding was not relevant in biochemical experiments such as enzyme purification, activity measurements, NMR measurements, and structural analyses such as crystallography, SAXS, and electron microscopy, because sample information does not influence the results for these experiments.

Reporting for specific materials, systems and methods

We require information from authors about some types of materials, experimental systems and methods used in many studies. Here, indicate whether each material, system or method listed is relevant to your study. If you are not sure if a list item applies to your research, read the appropriate section before selecting a response.

Materials & experimental systems

n/a	Involved in the study
☒	☐ Antibodies
☒	☐ Eukaryotic cell lines
☒	☐ Palaeontology and archaeology
☒	☐ Animals and other organisms
☒	☐ Clinical data
☒	☐ Dual use research of concern
☒	☐ Plants

Methods

n/a	Involved in the study
☒	☐ ChIP-seq
☒	☐ Flow cytometry
☒	☐ MRI-based neuroimaging
