# Supplementary material for: Identification and characterization of endo-α-, exo-α-, and exo-β-d-arabinofuranosidases degrading lipoarabinomannan and arabinogalactan of mycobacteria
Source: Nat Commun. 2023 Sep 19;14:5803. doi: 10.1038/s41467-023-41431-2 (PMC10509167; doi:10.1038/s41467-023-41431-2)
Supplement: Supplementary file 4 — Description of Additional Supplementary files [file 41467_2023_41431_MOESM4_ESM.docx]

**Description of Additional Supplementary Files**

File Name: Supplementary Data 1

Description: SAXS results for EndoMA1

File Name: Supplementary Data 2

Description: NMR spectra of oligosaccharides as additional information for synthesis of oligo-D-arabinofuranosides

File Name: Supplementary Data 3

Description: Sequences of primers used for gene cloning, mutagenesis, and quantitative real-time PCR
