# Supplementary material for: Identification and characterization of endo-α-, exo-α-, and exo-β-d-arabinofuranosidases degrading lipoarabinomannan and arabinogalactan of mycobacteria
Source: Nat Commun. 2023 Sep 19;14:5803. doi: 10.1038/s41467-023-41431-2 (PMC10509167; doi:10.1038/s41467-023-41431-2)

Supplementary Data 2
Additional information for synthesis of oligo-D-arabinofuranosides

NMR spectra of oligosaccharides

^{1}H NMR spectrum of **Ara*f*$_3$LT (SI-2a)** in CD_3OD

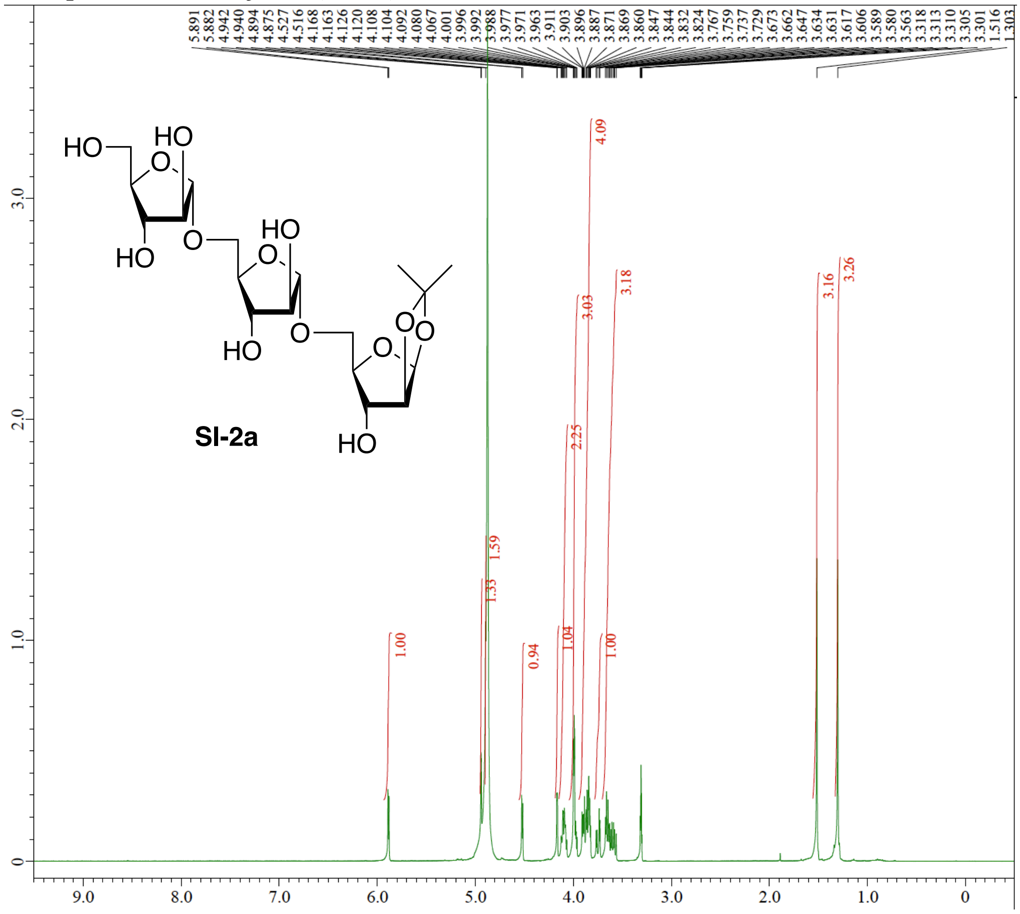

^{1}H NMR spectrum of **Araf3LT (SI-2a)** in CD_3OD

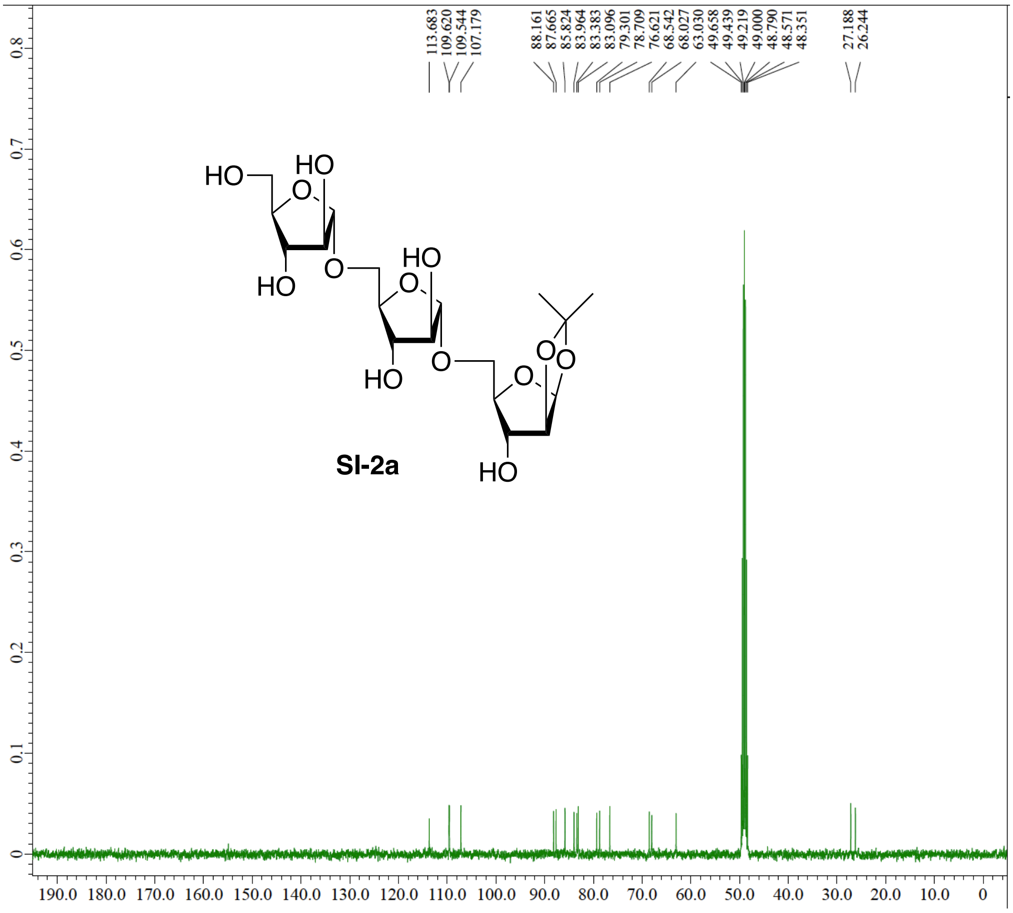

^{1}H NMR spectrum of **Ara*f*$_5$BT (SI-4a)** in D_2O

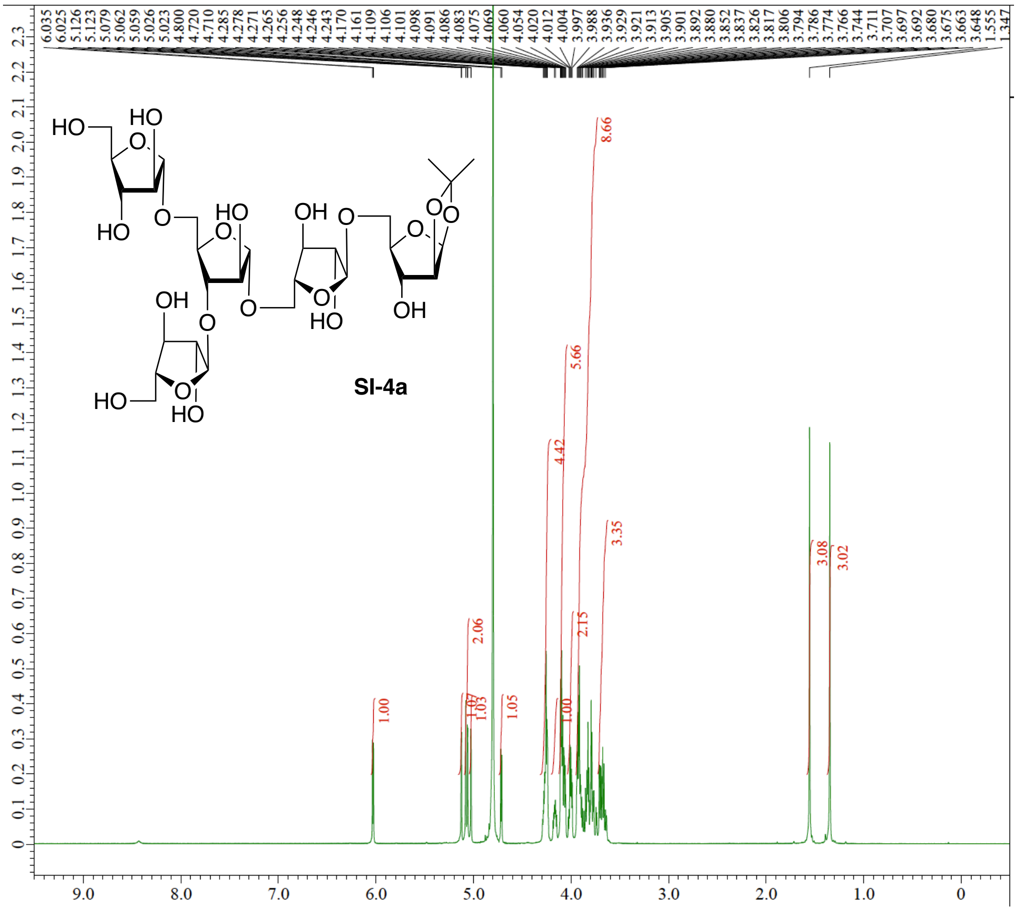

^{13}C NMR spectrum of **Ara*f*$_5$BT** (**SI-4a**) in D_2O (X indicated the impurity.)

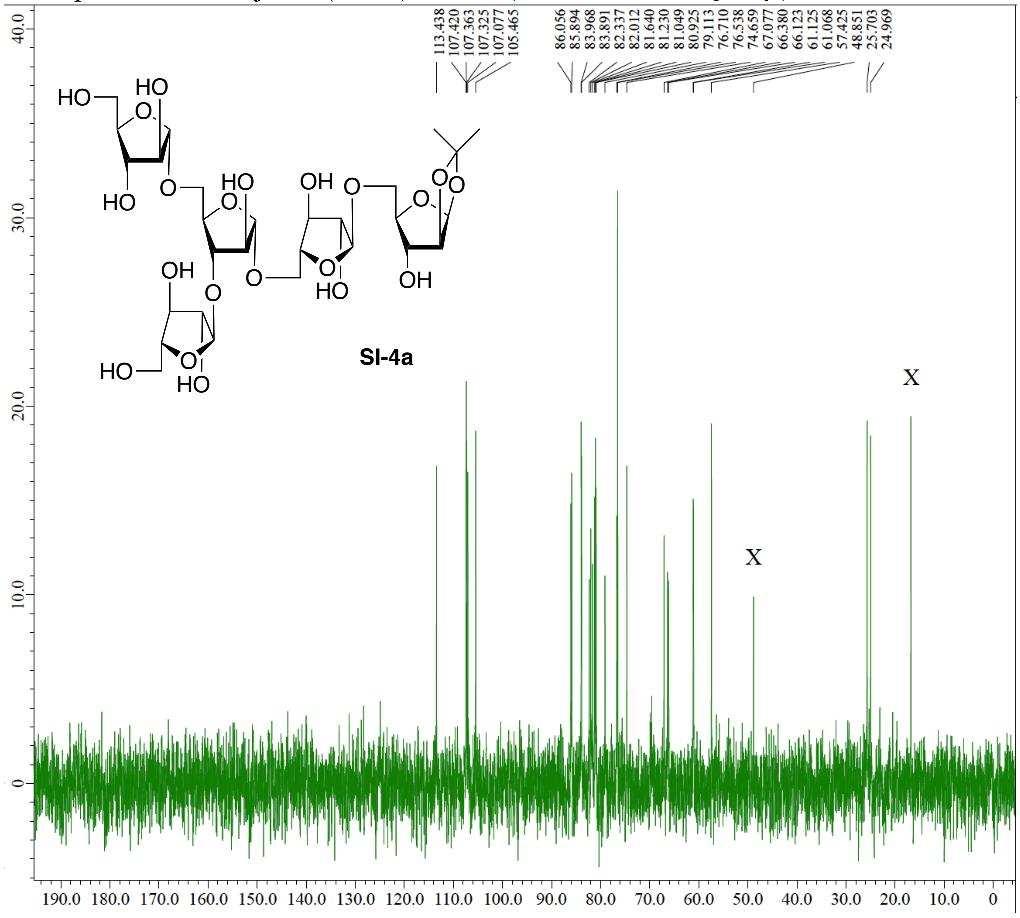

^{1}H NMR spectrum of **Ara*f*$_8$BT** (**SI-4b**) in D_2O (X indicated the impurity.)

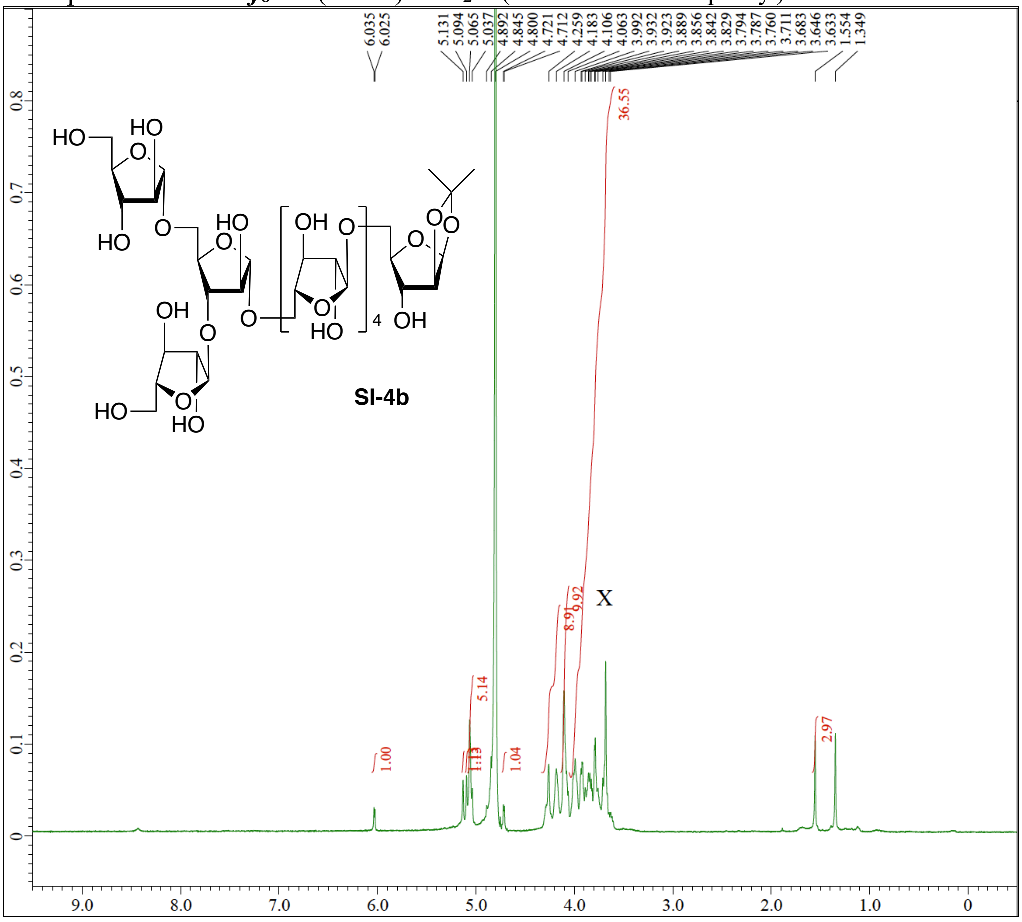

^{13}C NMR spectrum of **Ara*f*$_8$BT** (**SI-4b**) in D_2O (X indicated the impurity.)

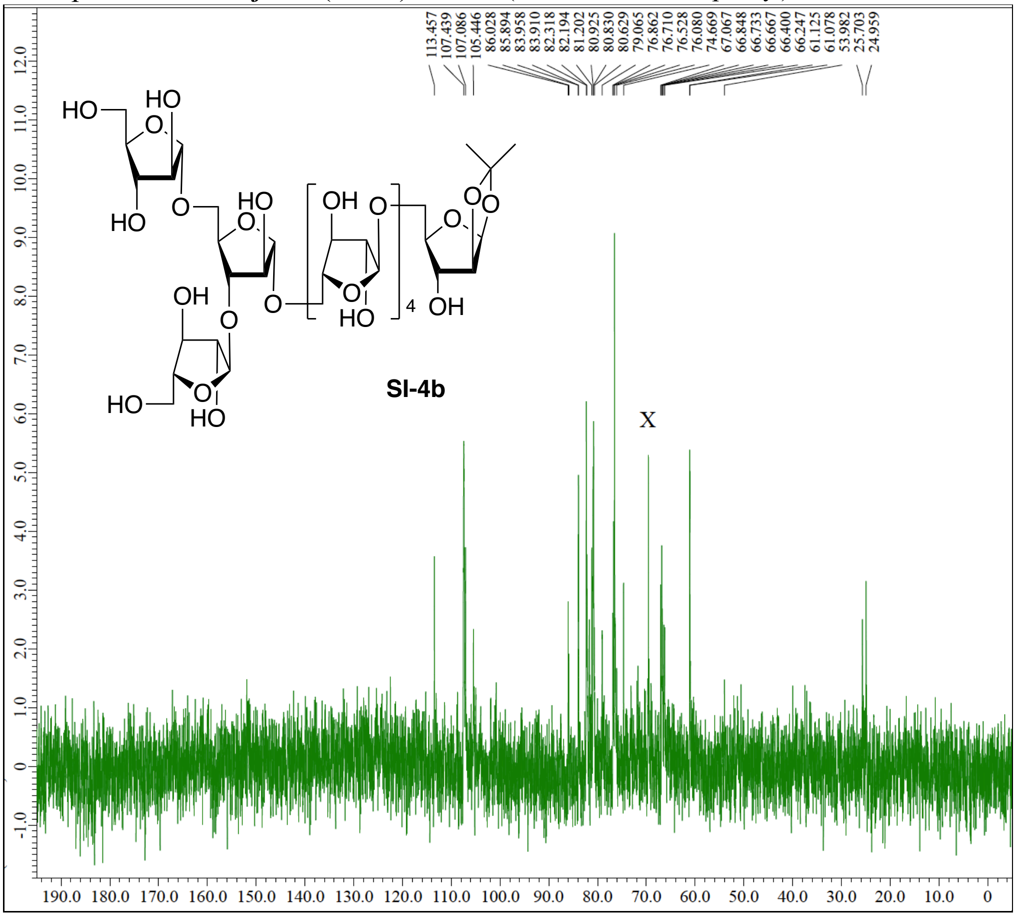

^{1}H NMR spectrum of **SI-6** in $CDCl_3$

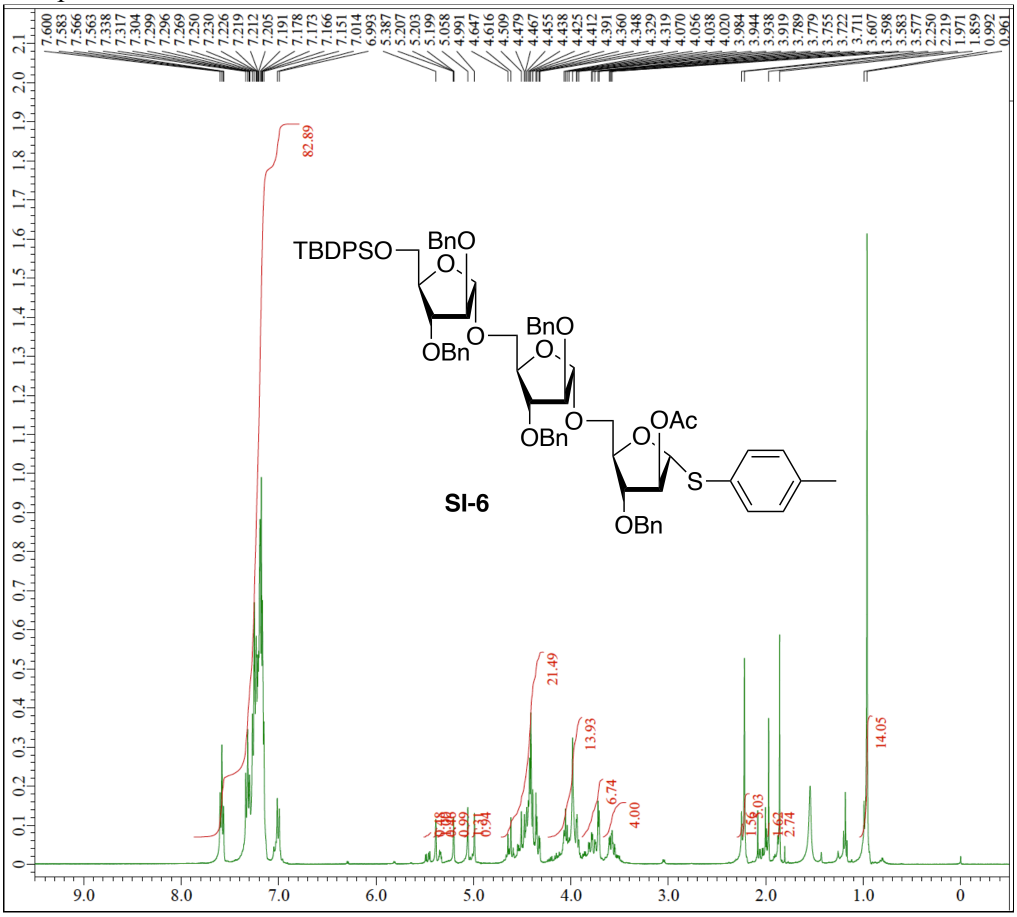

^{13}C NMR spectrum of **SI-6** in $CDCl_3$

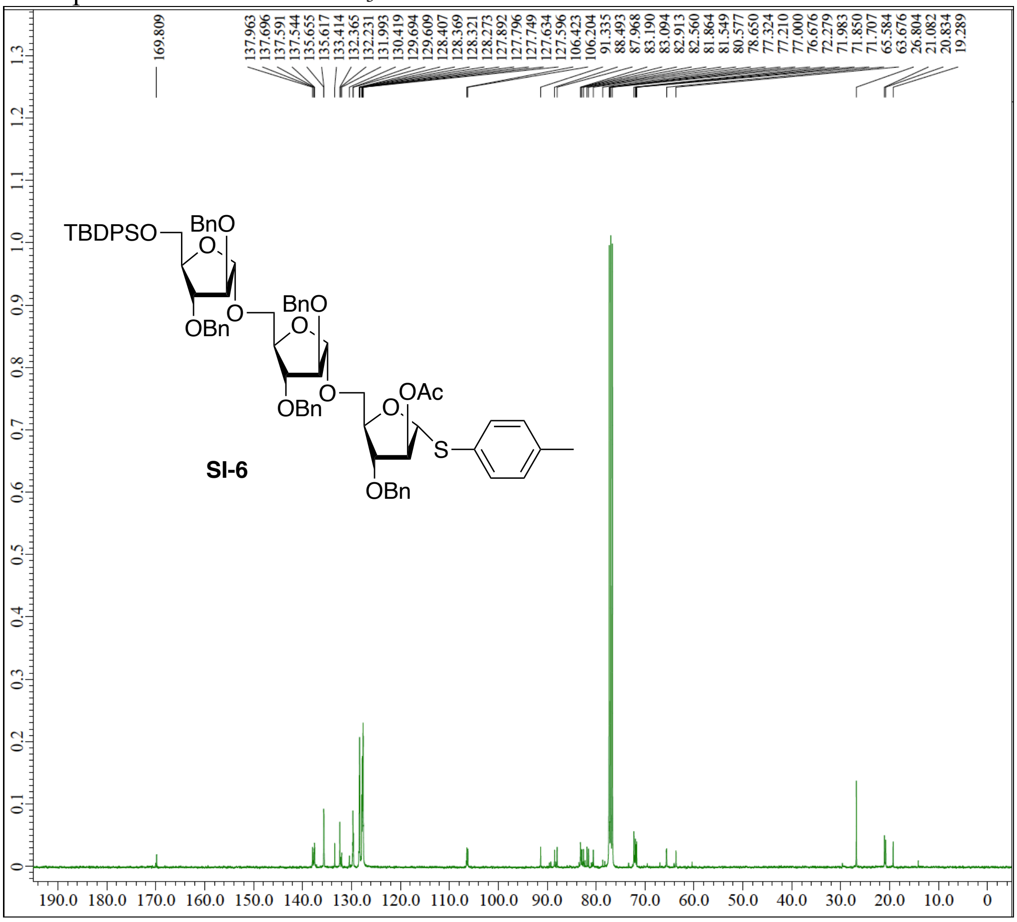

^{1}H NMR spectrum of **SI-7** in $CDCl_3$

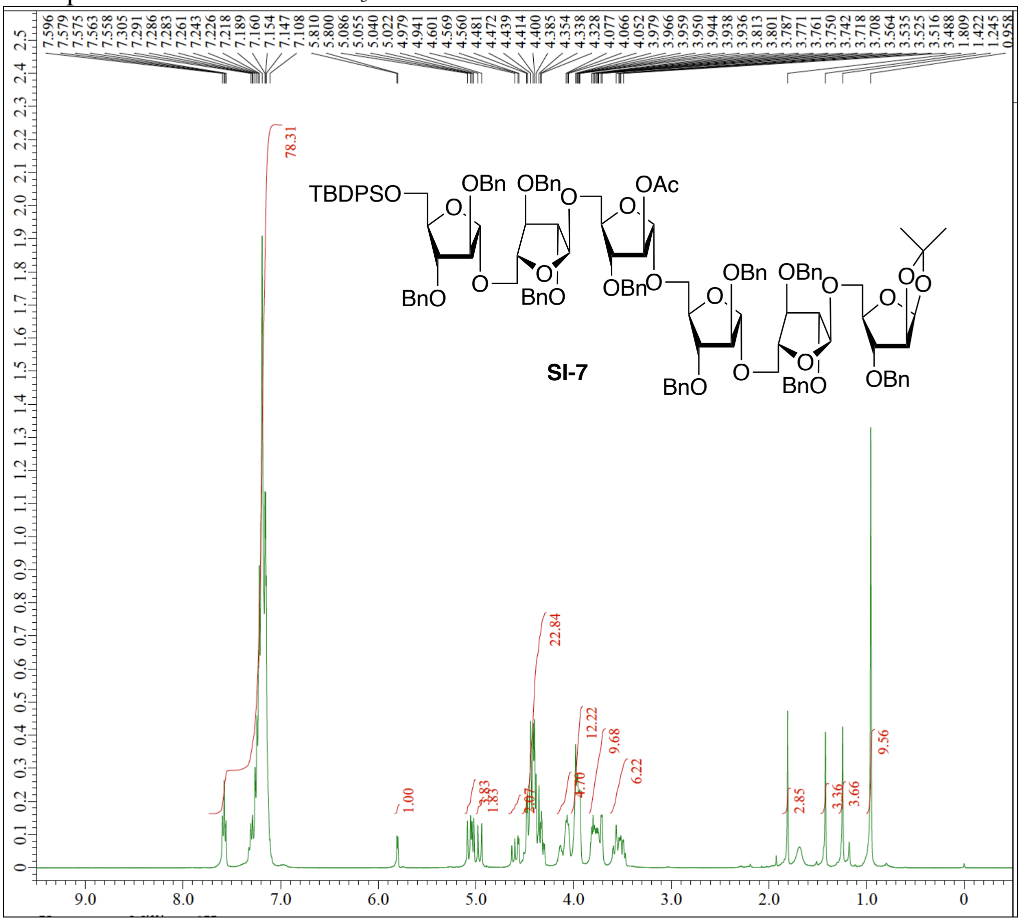

^{13}C NMR spectrum of **SI-7** in $CDCl_3$

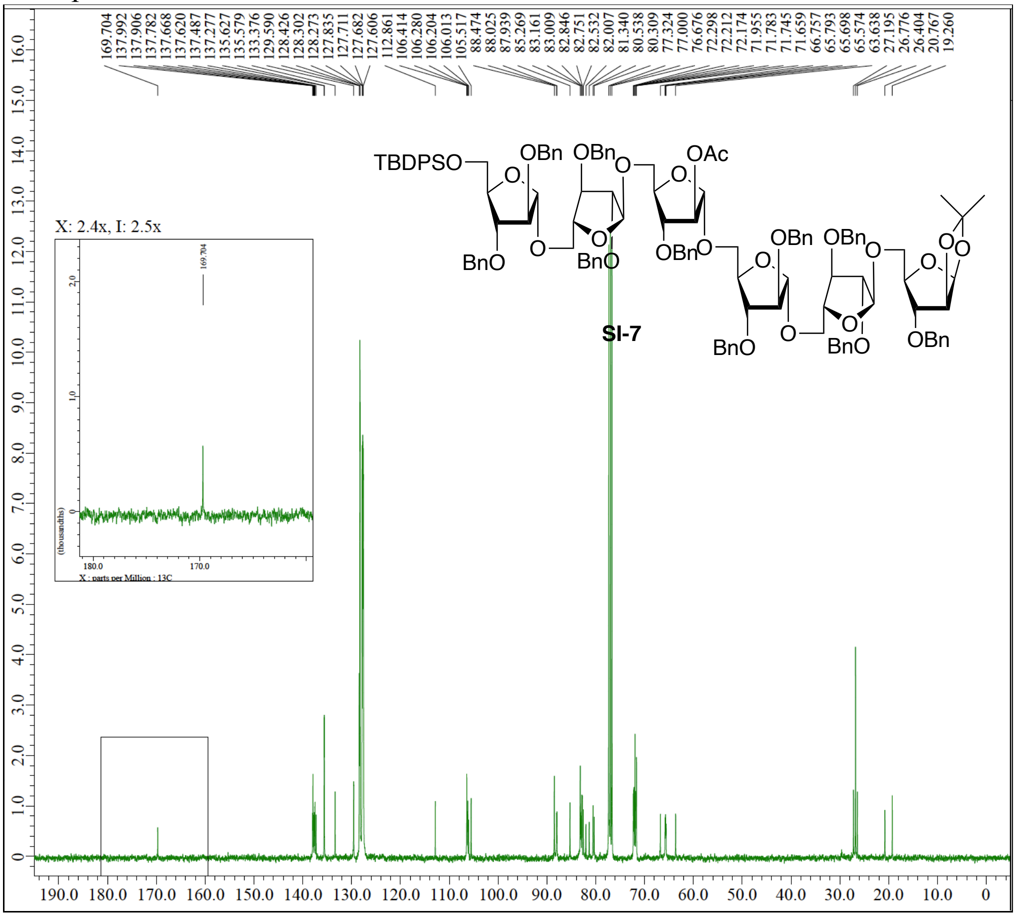

^{1}H NMR spectrum of **SI-1b** in $CDCl_3$

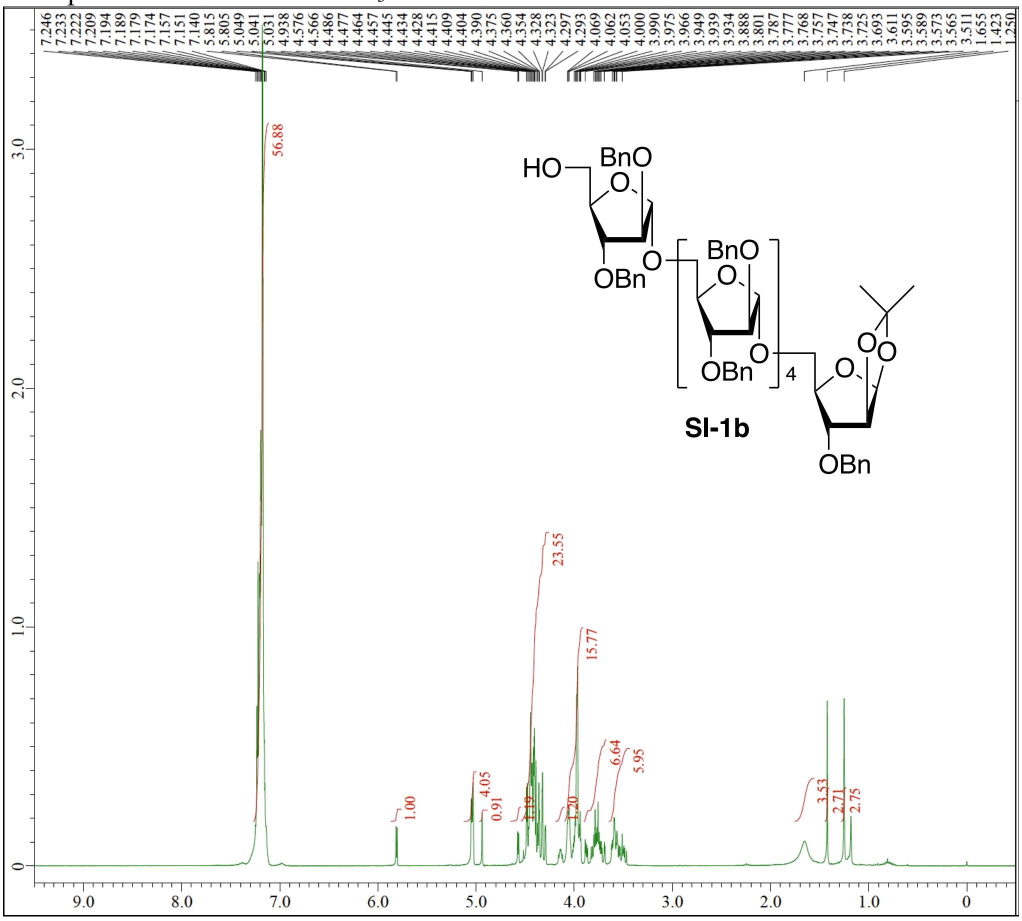

^{13}C NMR spectrum of **SI-1b** in $CDCl_3$

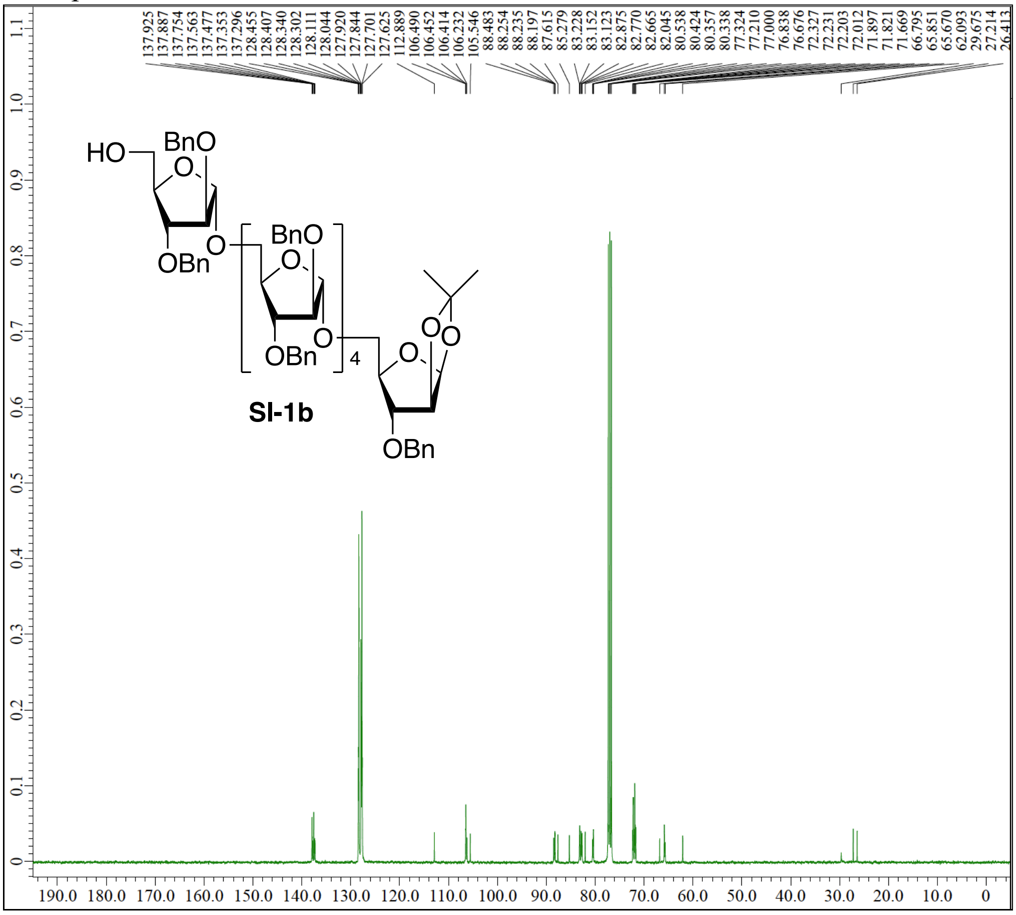

^{1}H NMR spectrum of **SI-8** in $CDCl_3$

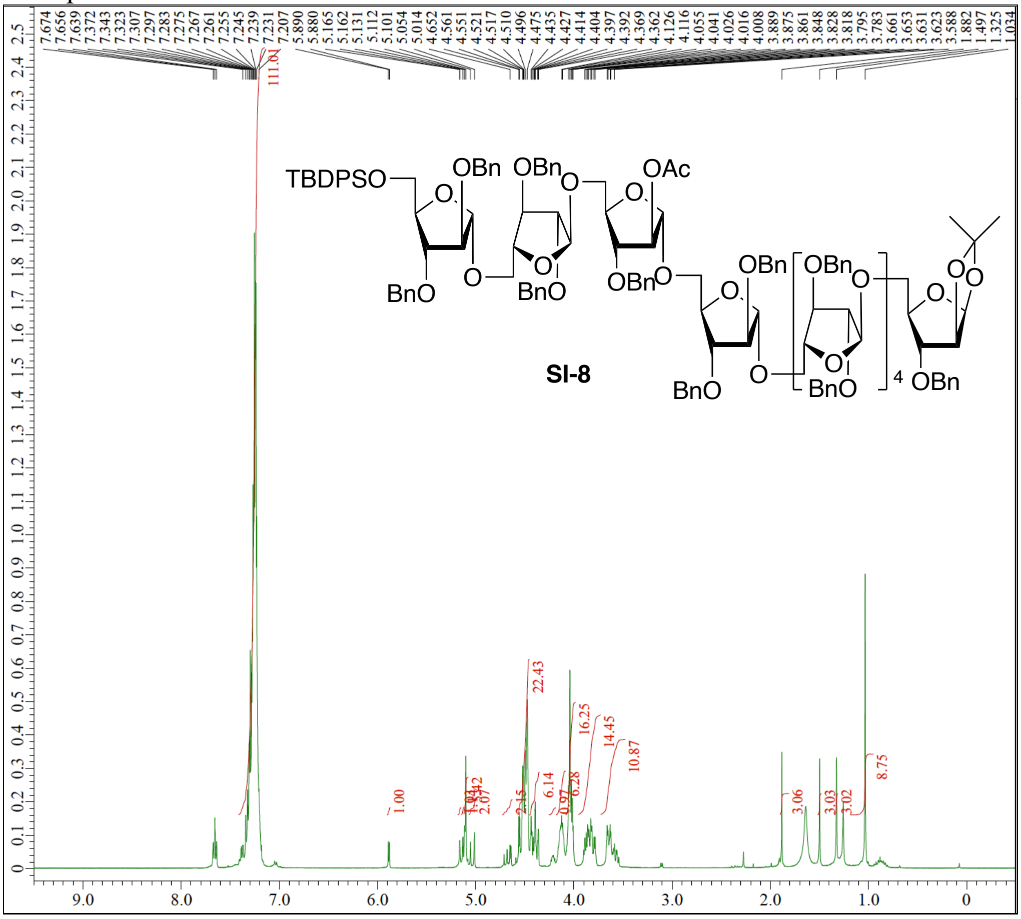

^{13}C NMR spectrum of **SI-8** in $CDCl_3$

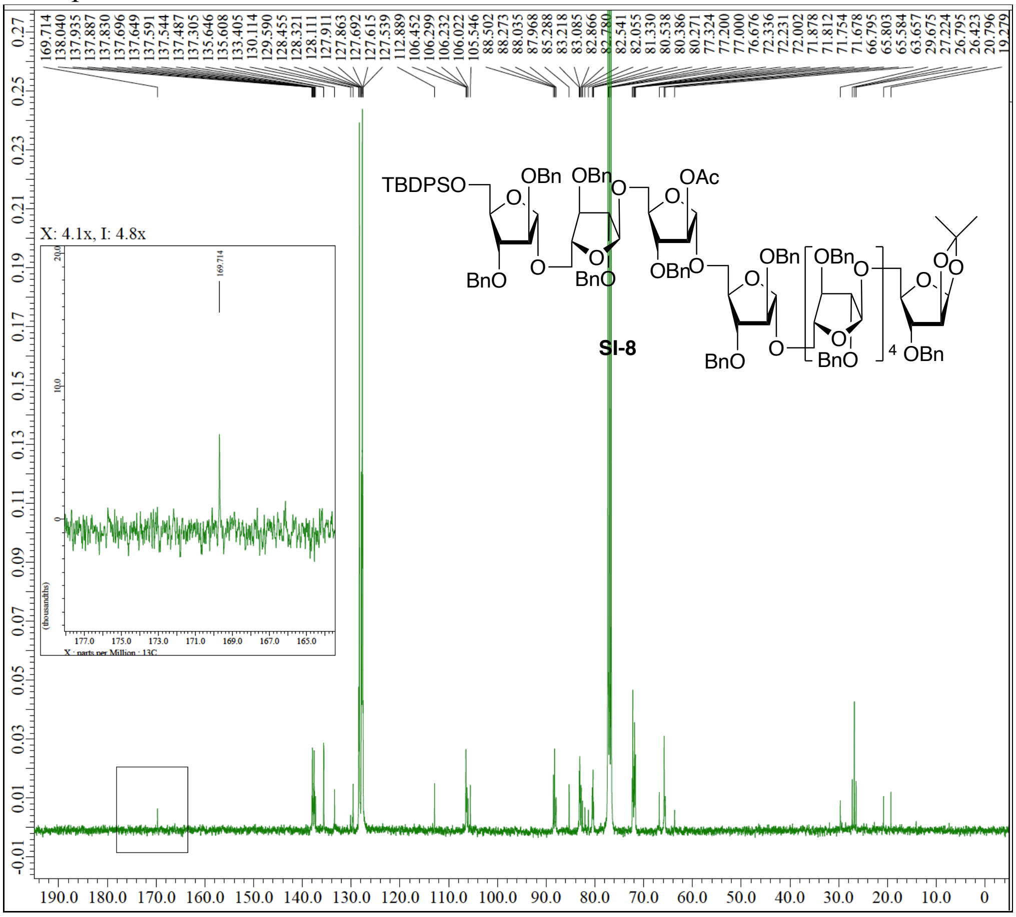

^{1}H NMR spectrum of **SI-1c** in $CDCl_3$

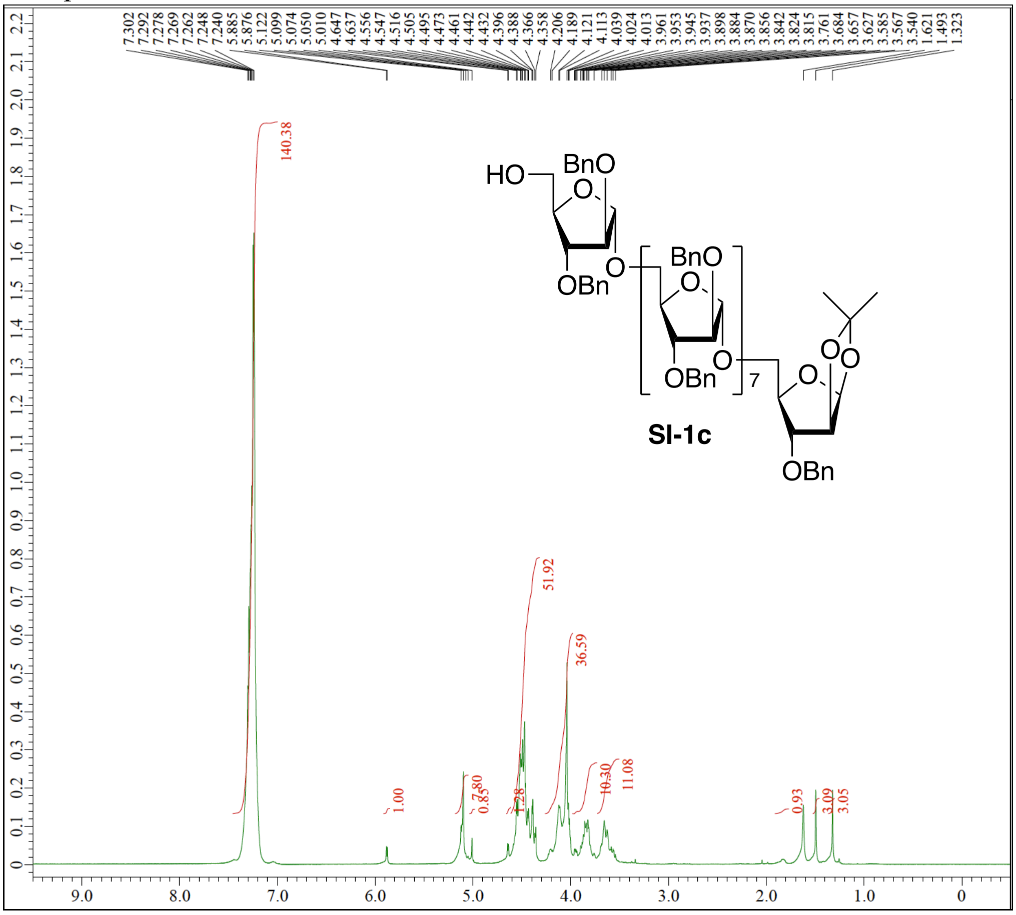

^{13}C NMR spectrum of **SI-1c** in $CDCl_3$

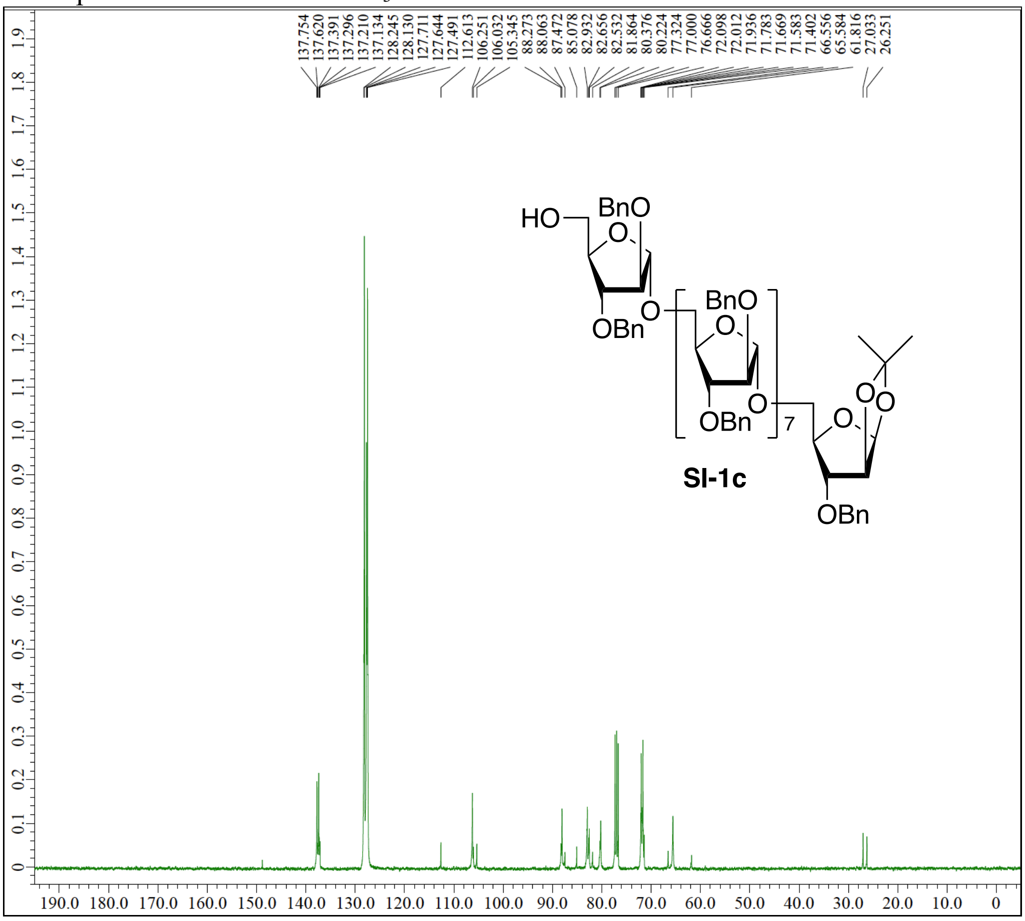

^{1}H NMR spectrum of **Ara*f*$_9$LT (SI-2c)** in D_2O

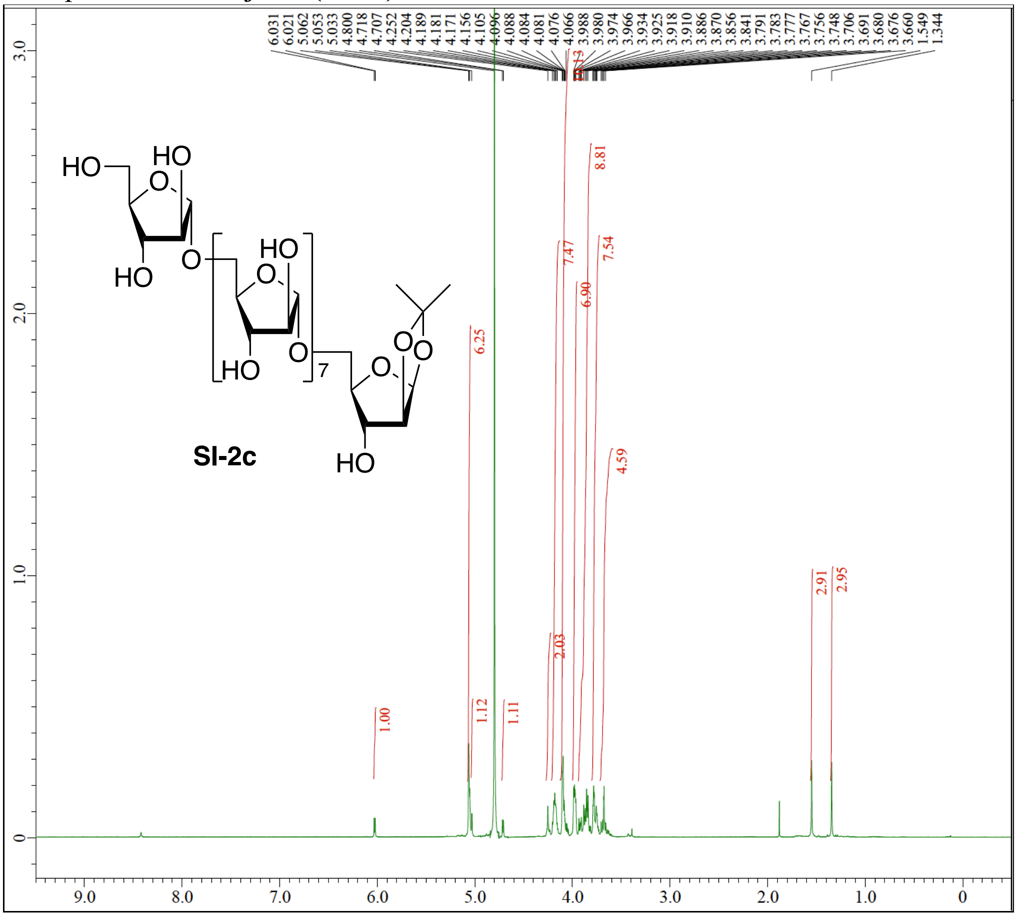

^{13}C NMR spectrum of **Ara*f*$_9$LT (SI-2c)** in D_2O

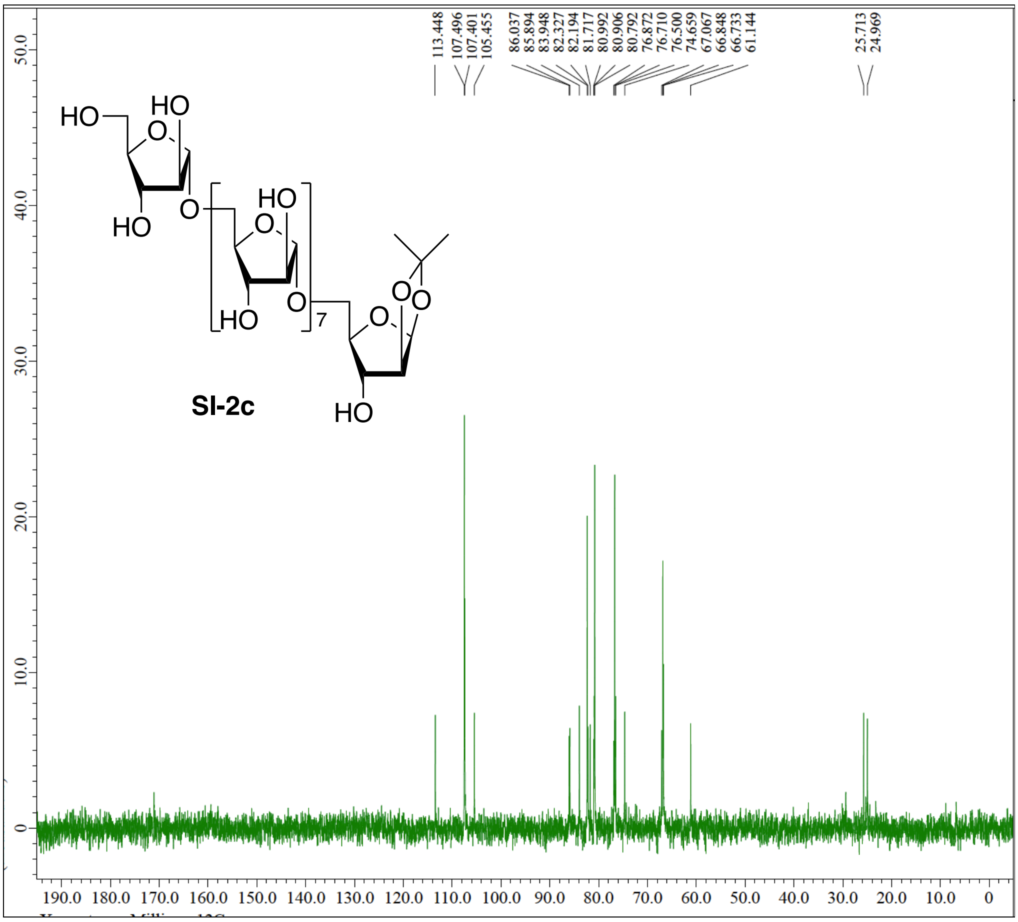

^{1}H NMR spectrum of **SI-11** in $CDCl_3$

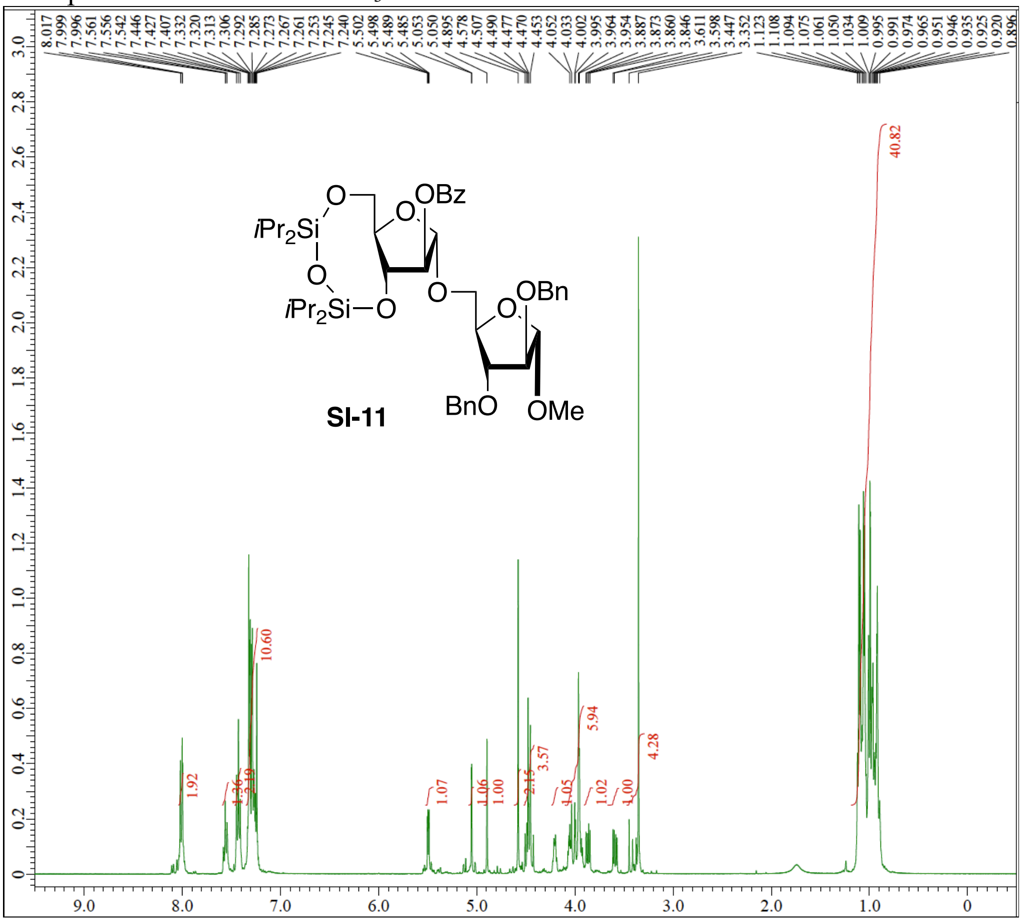

^{13}C NMR spectrum of **SI-11** in $CDCl_3$

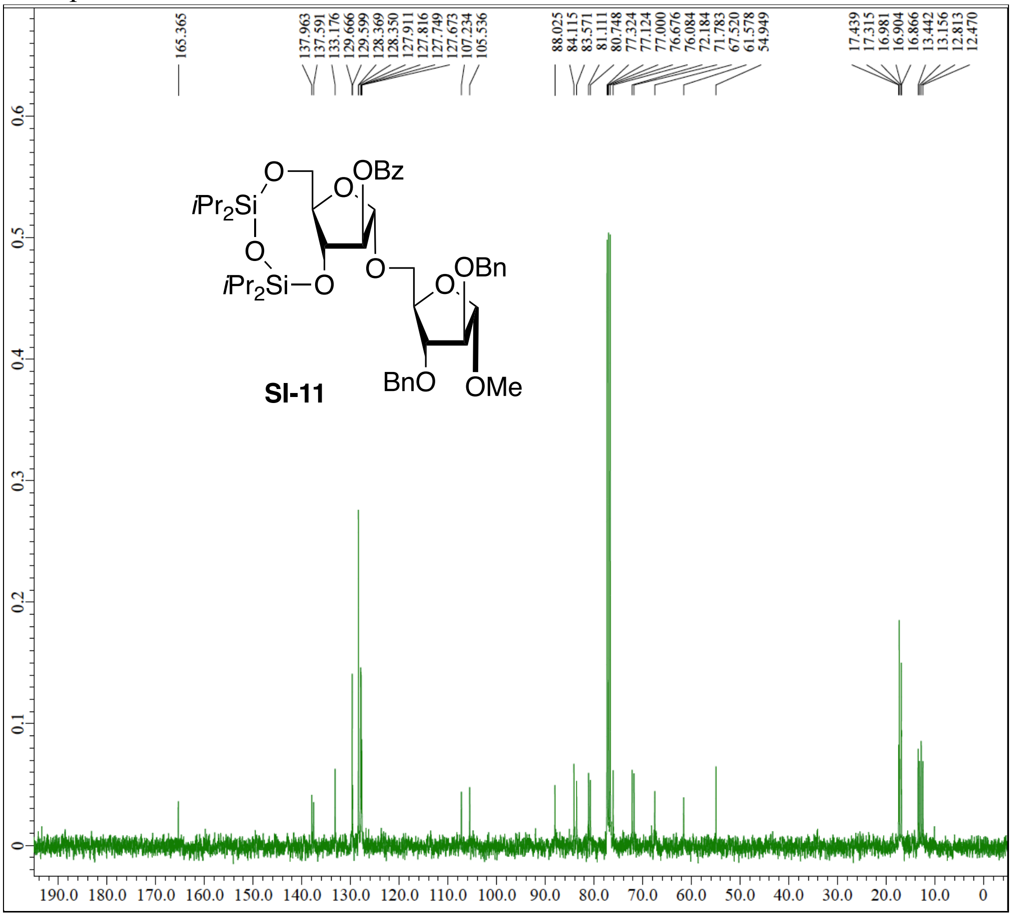

^{1}H NMR spectrum of **SI-12** in $CDCl_3$

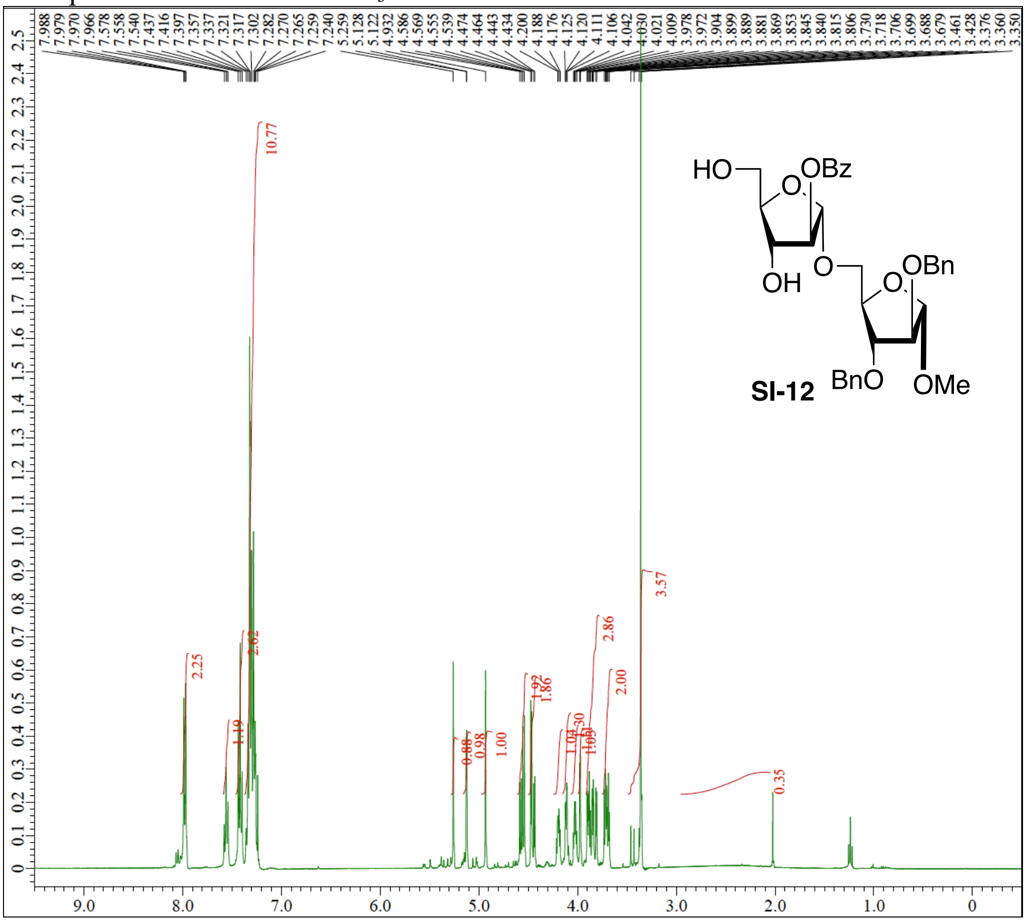

^{13}C NMR spectrum of **SI-12** in $CDCl_3$

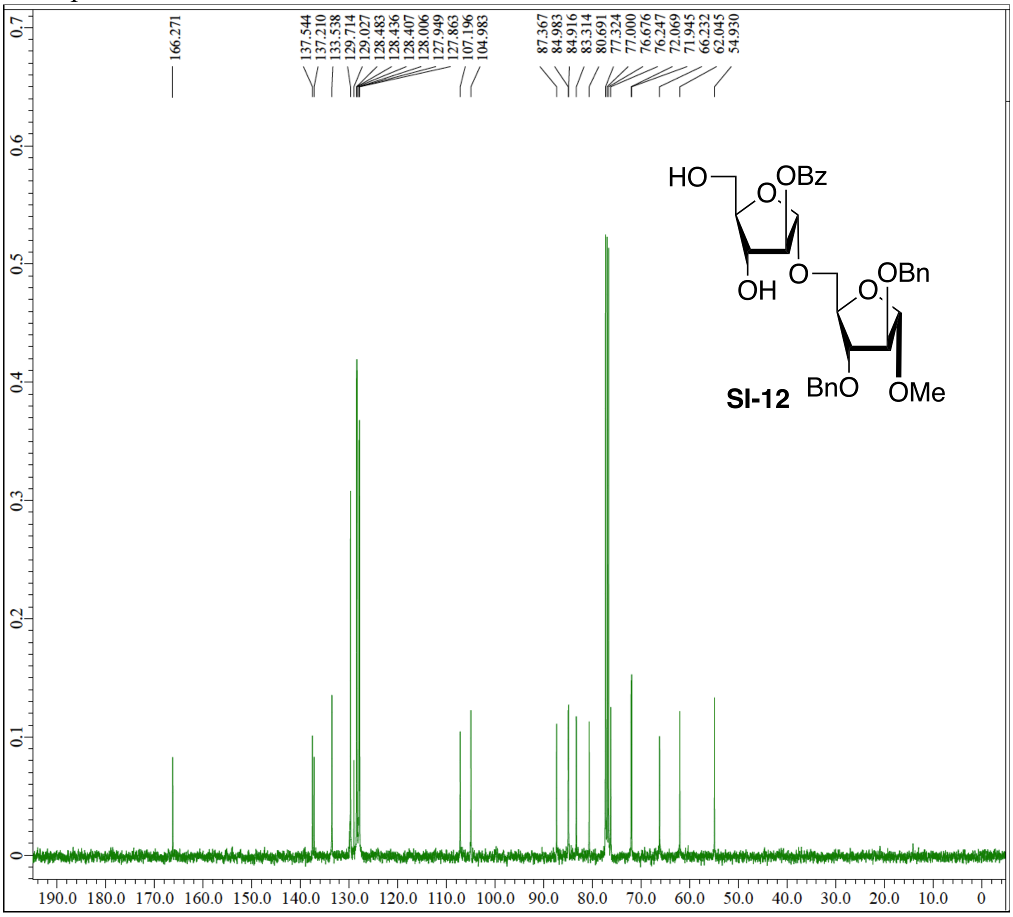

^{1}H NMR spectrum of **SI-14** in $CDCl_3$

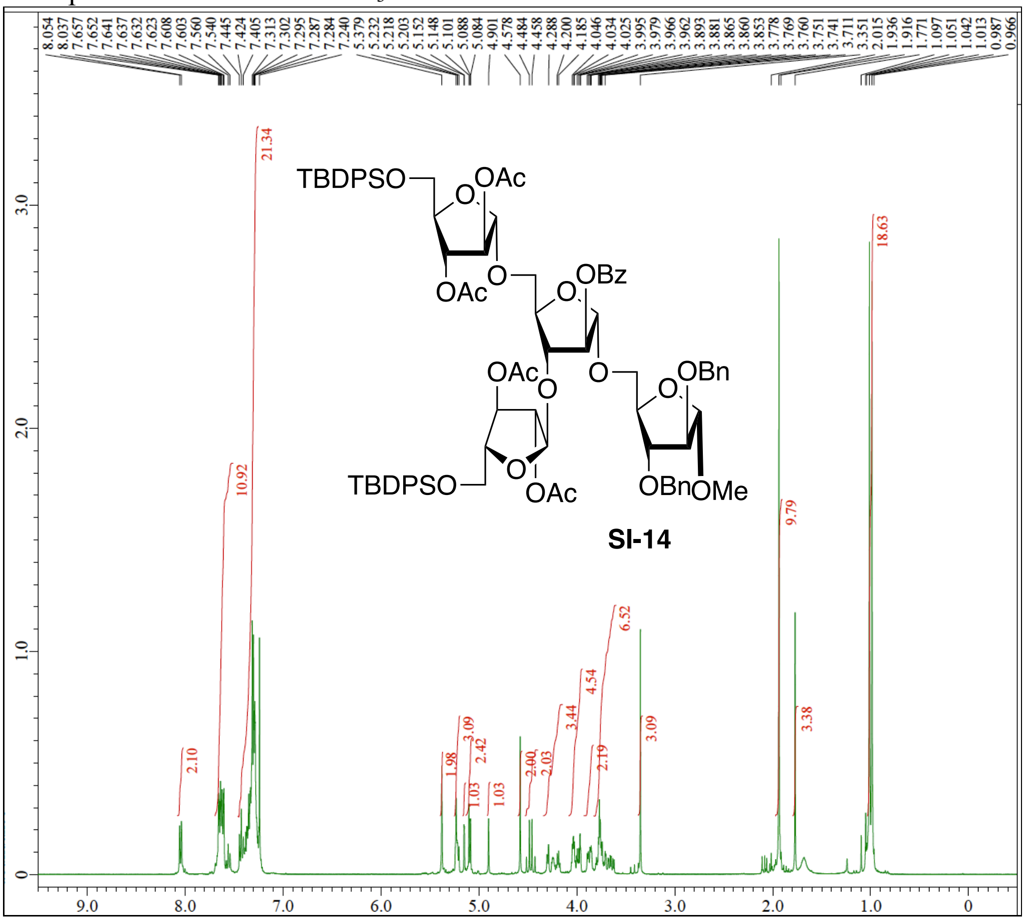

^{13}C NMR spectrum of **SI-14** in $CDCl_3$

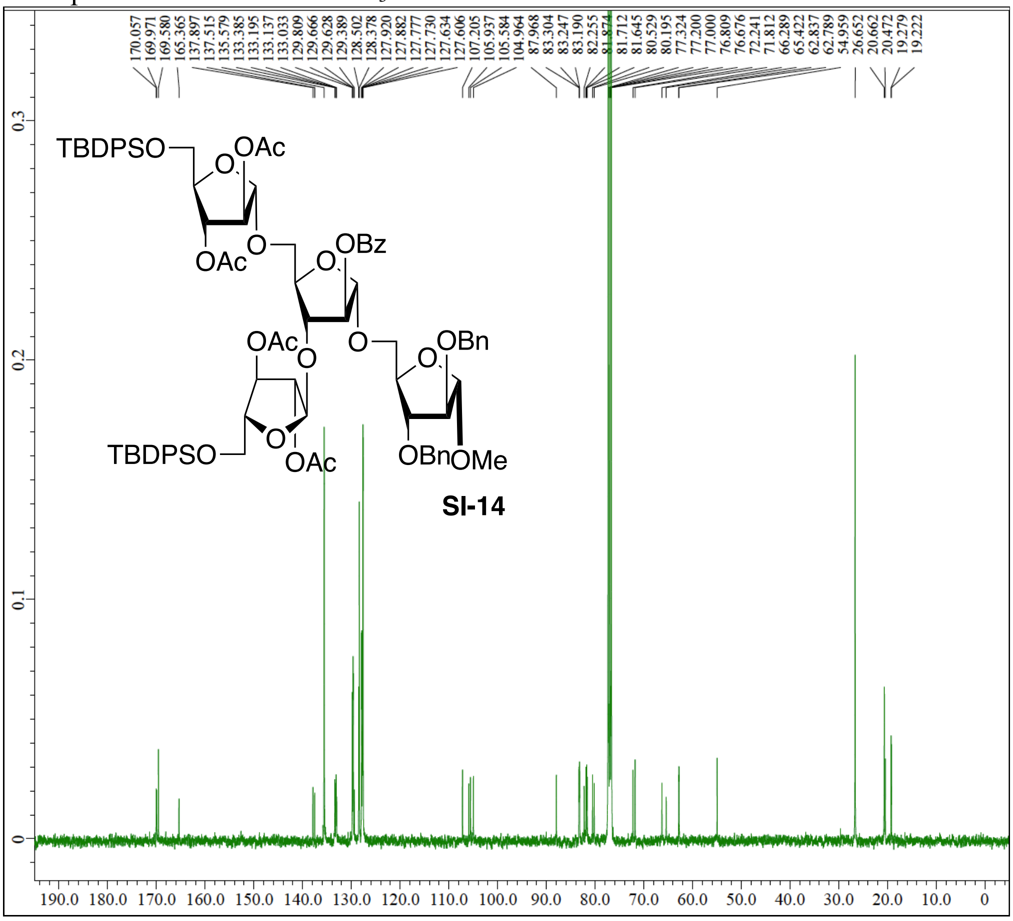

^{1}H NMR spectrum of **SI-15** in D_2O (synthetic)

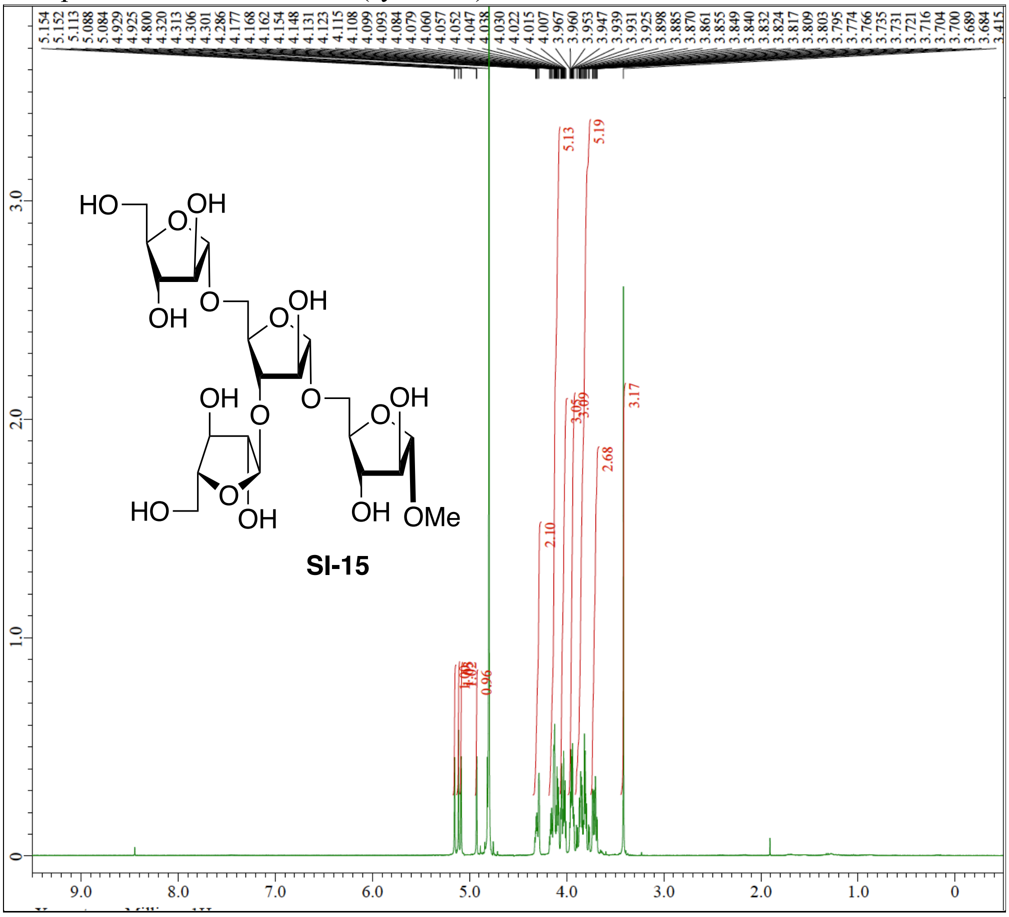

^{13}C NMR spectrum of **SI-15** in D_2O (synthetic)

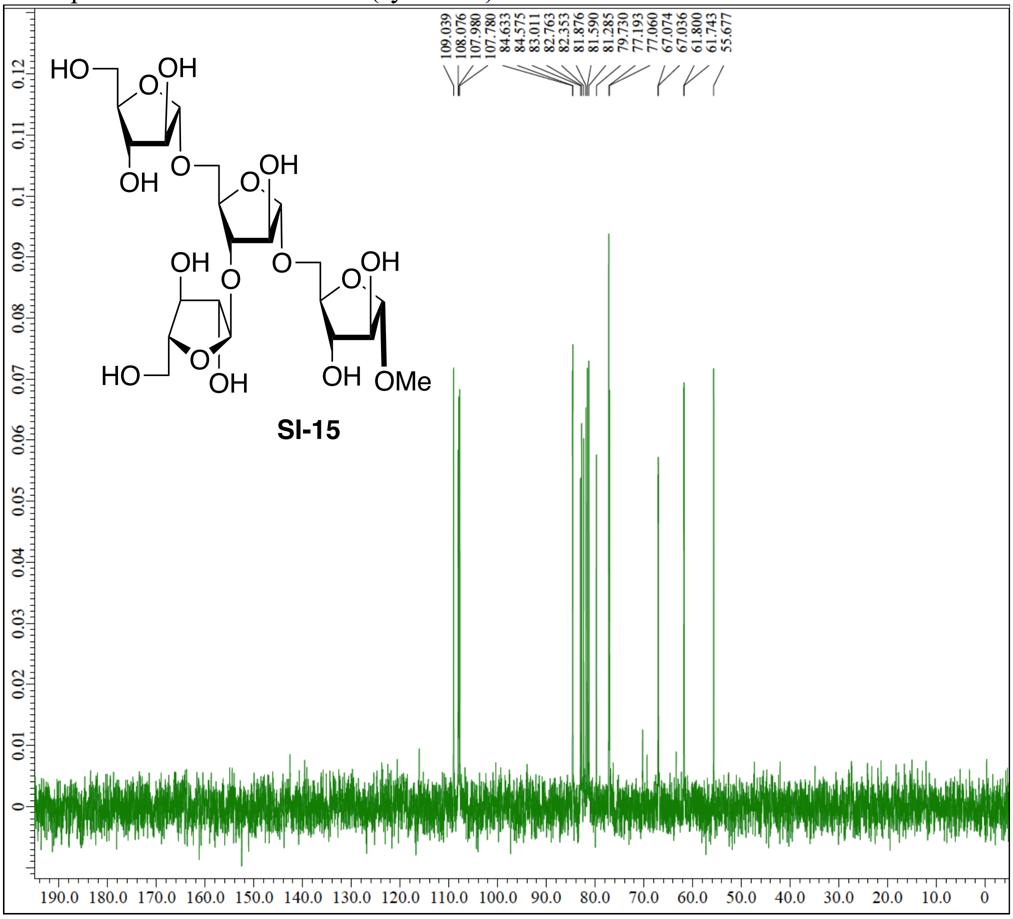

^{1}H-^{1}H COSY spectrum of synthetic **SI-15** in D_2O (synthetic)

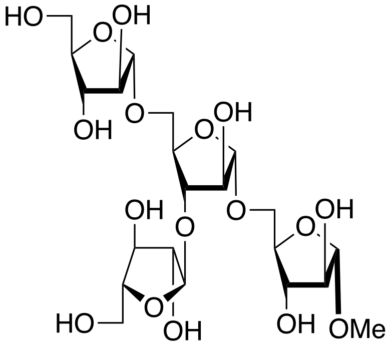

SI-15

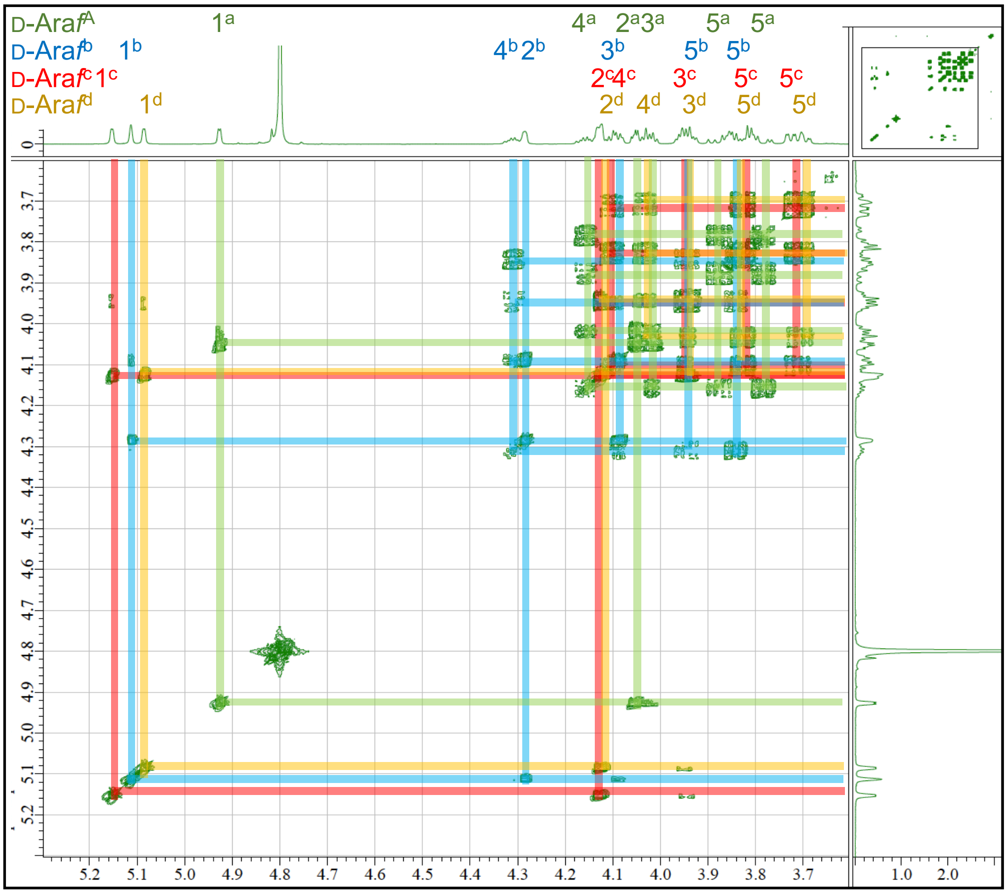

HMQC spectrum of **SI-15** in D_2O (synthetic)

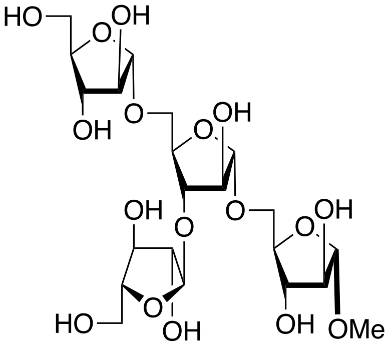

SI-15

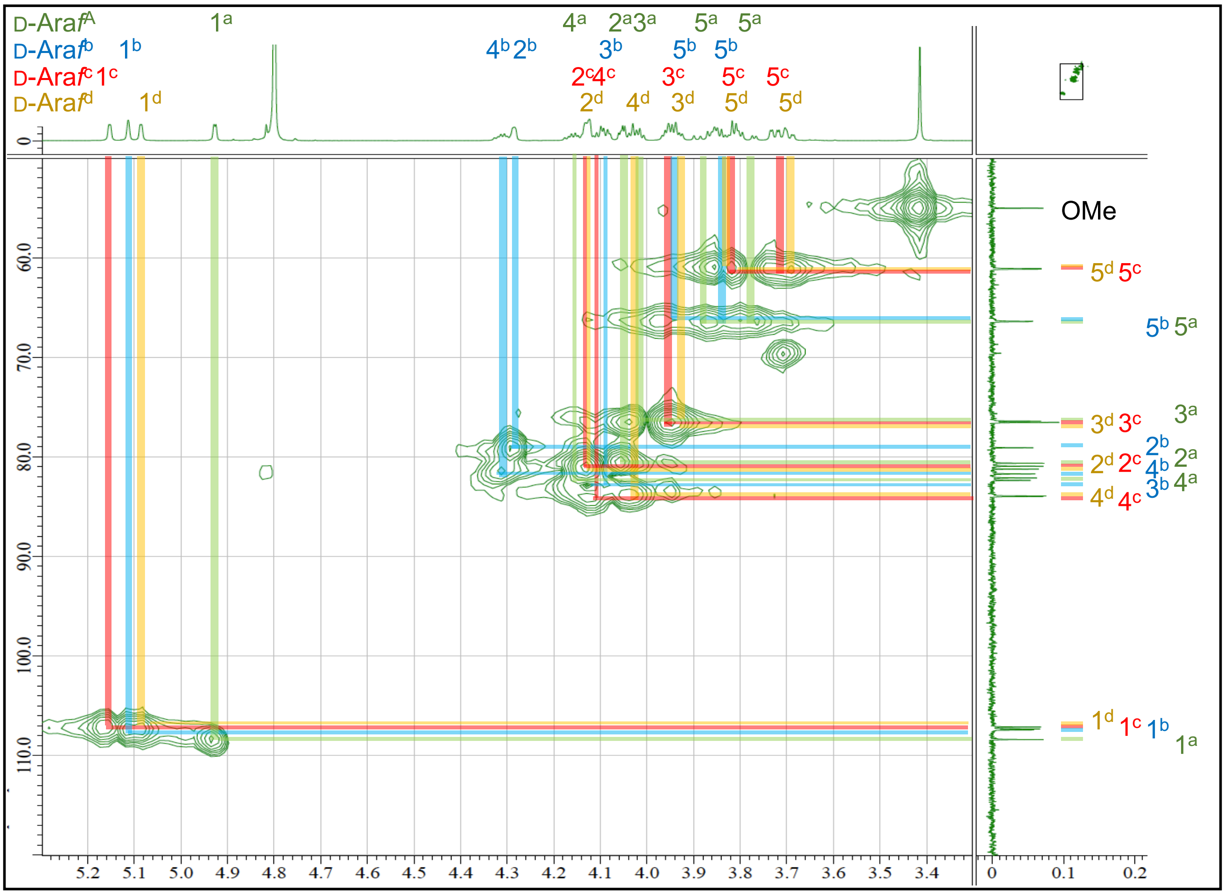

HMBC spectrum of **SI-15** in D_2O (synthetic)

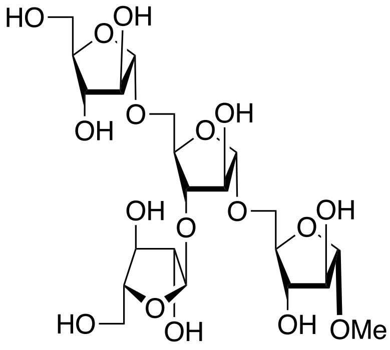

SI-15

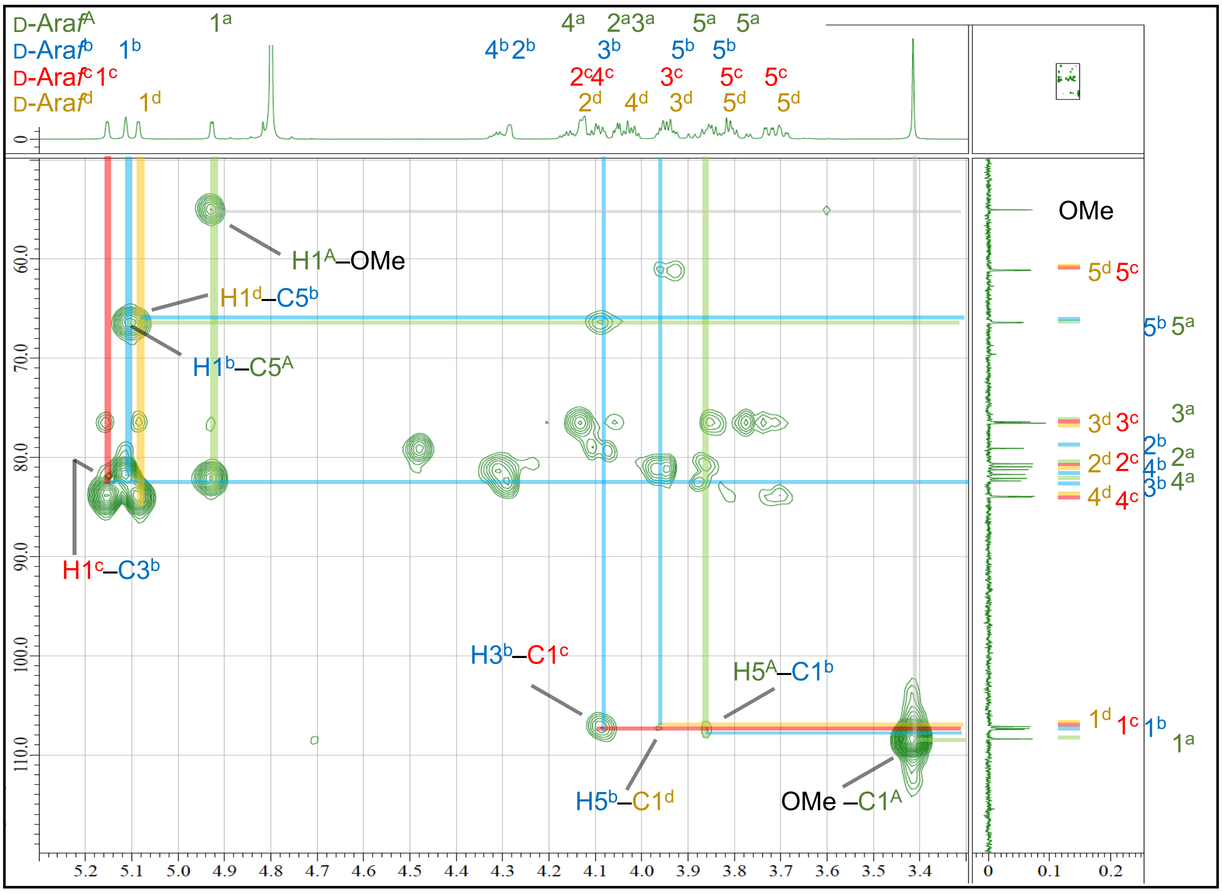

^{1}H NMR spectrum of **SI-15** in D_2O obtained by enzymatic cleavage of **SI-4b** in the presence of MeOH

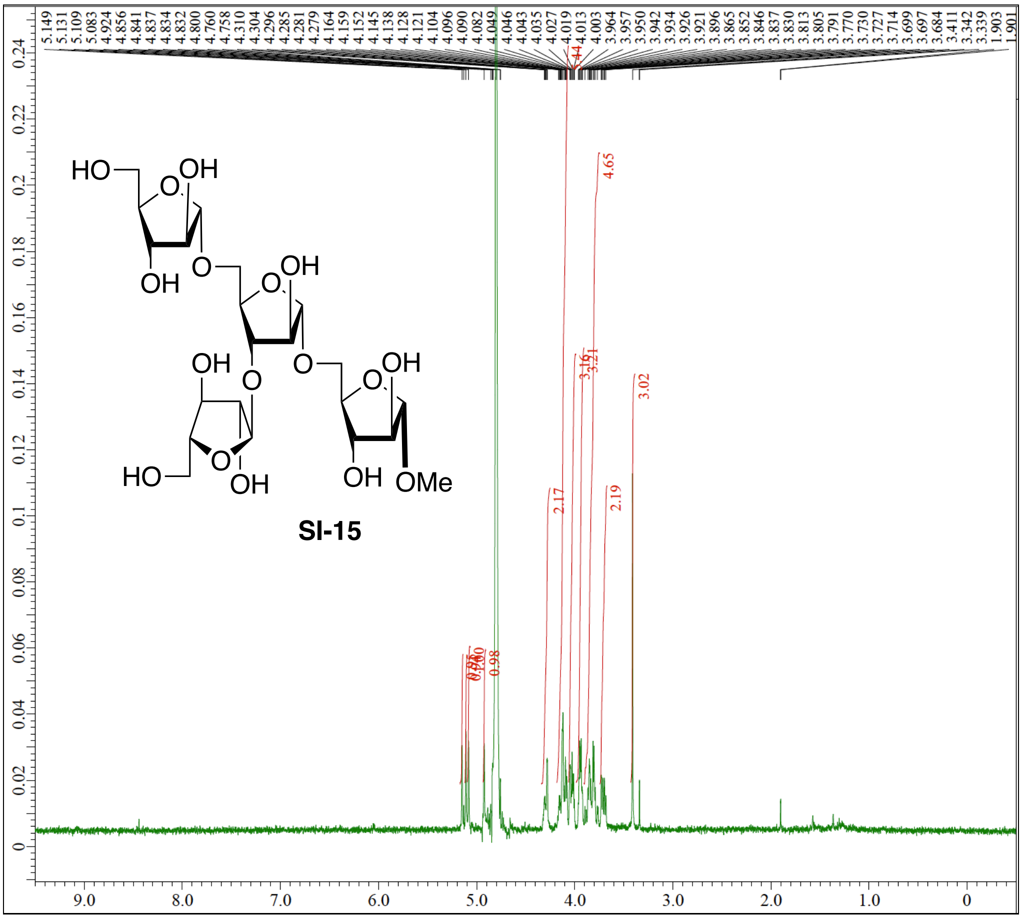

^{1}H-^{1}H COSY spectrum of **SI-15** in D_2O obtained by enzymatic cleavage of **SI-4b** in the presence of MeOH

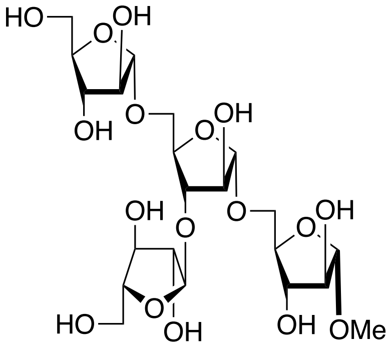

SI-15

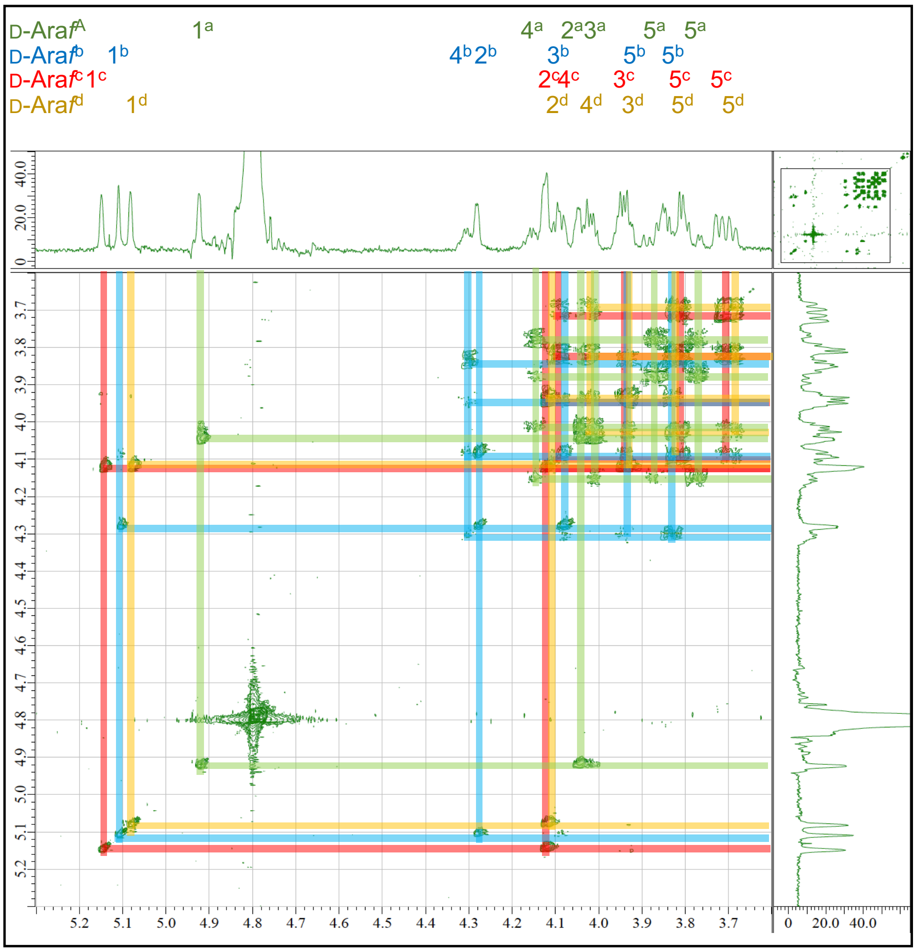

Supplement: Supplementary file 6 — Dataset 2 [file 41467_2023_41431_MOESM6_ESM.pdf]
