# Supplementary material for: Identification and characterization of endo-α-, exo-α-, and exo-β-d-arabinofuranosidases degrading lipoarabinomannan and arabinogalactan of mycobacteria
Source: Nat Commun. 2023 Sep 19;14:5803. doi: 10.1038/s41467-023-41431-2 (PMC10509167; doi:10.1038/s41467-023-41431-2)
Supplement: Supplementary file 8 — Source Data [file 41467_2023_41431_MOESM8_ESM.zip › Source Data/SupFig6_d2.pdf]

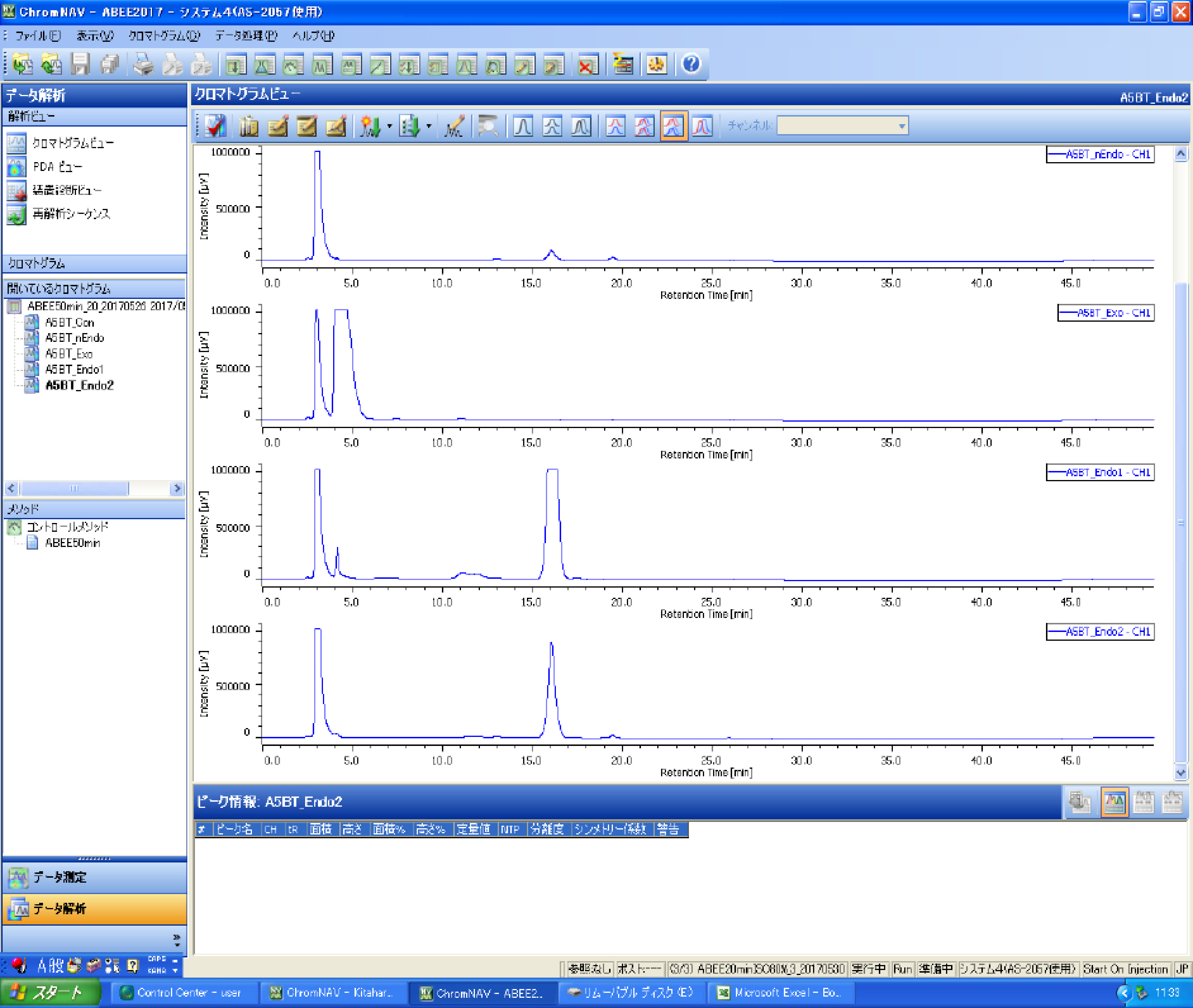
ChromNAV - ABEE2017 - システム4(AS-2057使用)
ファイル(F) 表示(V) クロマトグラム(C) データ処理(P) ヘルプ(H)
データ解析
解析ビュー
クロマトグラムビュー
PDA ビュー
装置診断ビュー
再解析シーケンス
クロマトグラム
開いているクロマトグラム
A5BT_Con
A5BT_nEndo
A5BT_Exo
A5BT_Endo1
A5BT_Endo2
メソッド
コントロールメソッド
ABEE50min
データ測定
データ解析
クロマトグラムビュー
A5BT_Endo2
チャンネル:
A5BT_nEndo - CH1
A5BT_Exo - CH1
A5BT_Endo1 - CH1
A5BT_Endo2 - CH1
Intensity [μV]
1000000
500000
0
Retention Time [min]
0.0
5.0
10.0
15.0
20.0
25.0
30.0
35.0
40.0
45.0
ピーク情報: A5BT_Endo2
ピーク名 CH tR 面積 高さ 面積% 高さ% 定量値 NTP 分離度 シンメトリー係数 警告
参照なし ポスト- 実行中 Run 準備中 システム4(AS-2057使用) Start On Injection JP
スタート
Control Center - user
ChromNAV - Kitahar..
ChromNAV - ABEE2..
リムーバブル ディスク (E:)
Microsoft Excel - Bo..
11:33
