# Supplementary material for: Identification and characterization of endo-α-, exo-α-, and exo-β-d-arabinofuranosidases degrading lipoarabinomannan and arabinogalactan of mycobacteria
Source: Nat Commun. 2023 Sep 19;14:5803. doi: 10.1038/s41467-023-41431-2 (PMC10509167; doi:10.1038/s41467-023-41431-2)
Supplement: Supplementary file 8 — Source Data [file 41467_2023_41431_MOESM8_ESM.zip › Source Data/SupFig6_c2.pdf]

ChromNAV - ABEE2017 - システム4(AS-2057使用)

ファイル(F) 表示(V) クロマトグラム(C) データ処理(P) ヘルプ(H)

データ解析

解析ビュー

- クロマトグラムビュー
- PDA ビュー
- 装置診断ビュー
- 再解析シーケンス

クロマトグラム

開いているクロマトグラム

- ABEE50min_20_20170526 2017/0
  - ABBT_Con
  - ABBT_nEndo
  - ABBT_Exo
  - ABBT_Endo1
  - **ABBT_Endo2**

メソッド

- コントロールメソッド
  - ABEE50min

データ測定

データ解析

クロマトグラムビュー

ABBT_Endo2

チャンネル:

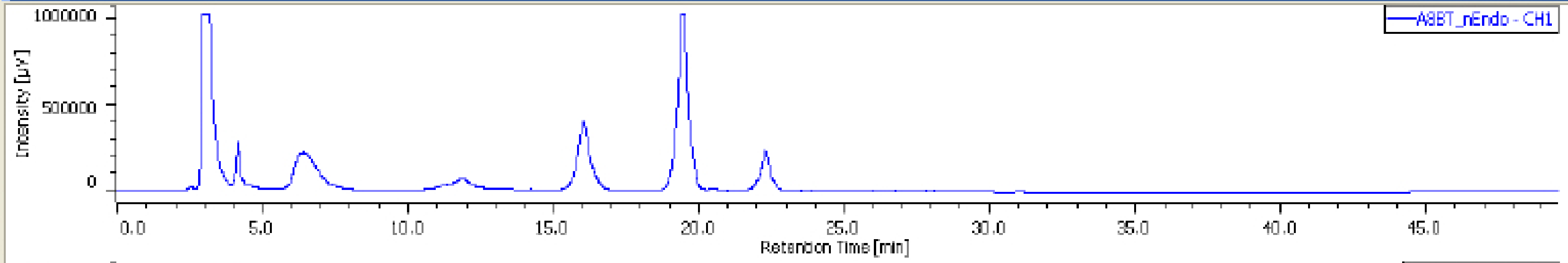

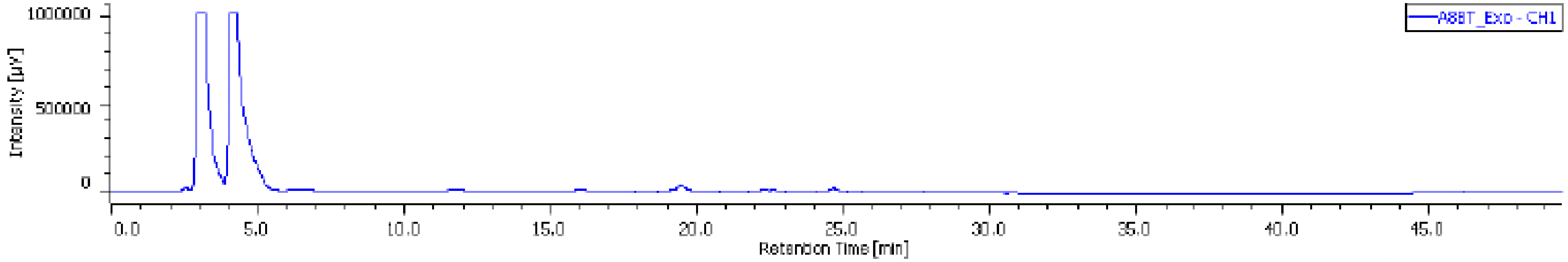

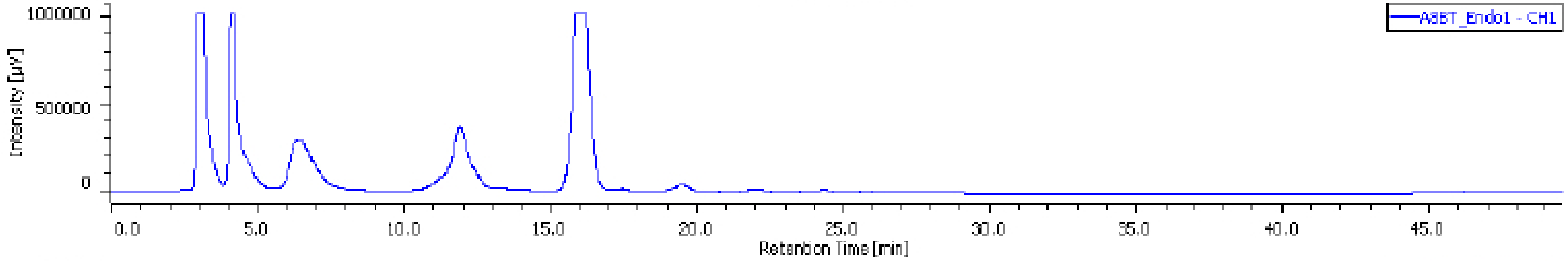

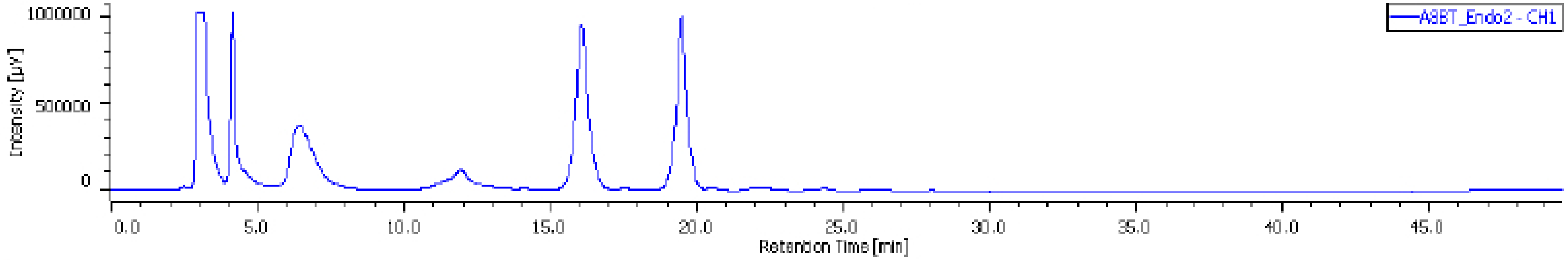

ピーク情報: ABBT_Endo2

#	ピーク名	CH	tR	面積	高さ	面積%	高さ%	定量値	NTP	分離度	シンメトリー係数	警告

A 般

参照なし | ポスト--- | (3/3) ABEE20min|SO80%_3_20170530 | 実行中 | Run | 準備中 | システム4(AS-2057使用) | Start On Injection | JP

スタート | Control Center - user | ChromNAV - Kitahar... | ChromNAV - ABEE2... | リムーバブル ディスク (E:) | Microsoft Excel - Bo... | 11:34
