# Supplementary figures and images for: Identification and characterization of endo-α-, exo-α-, and exo-β-d-arabinofuranosidases degrading lipoarabinomannan and arabinogalactan of mycobacteria
Source: Nat Commun. 2023 Sep 19;14:5803. doi: 10.1038/s41467-023-41431-2 (PMC10509167; doi:10.1038/s41467-023-41431-2)

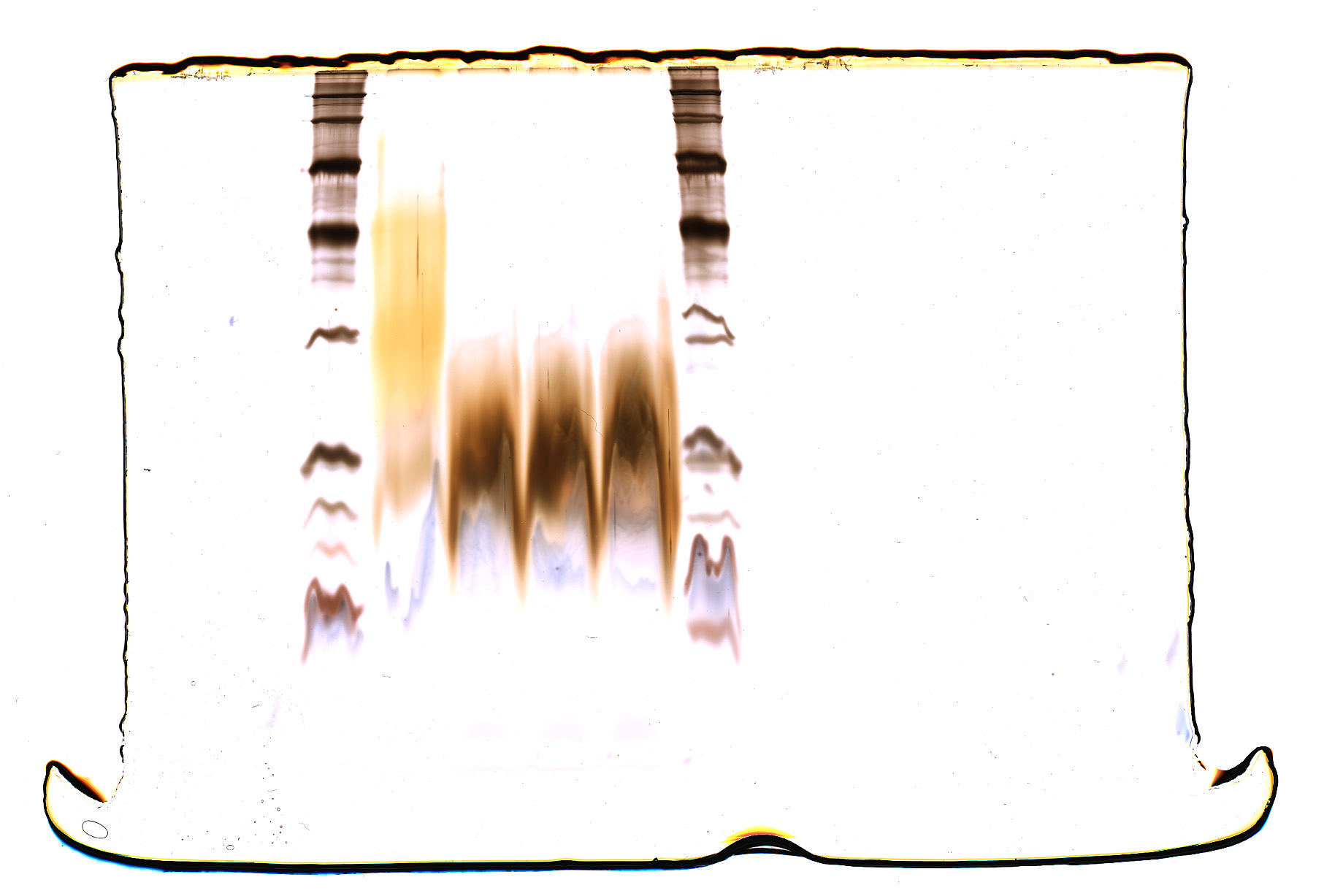

Supplement: Supplementary file 8 — Source Data [file 41467_2023_41431_MOESM8_ESM.zip › Source Data/SupFig1a.jpg]

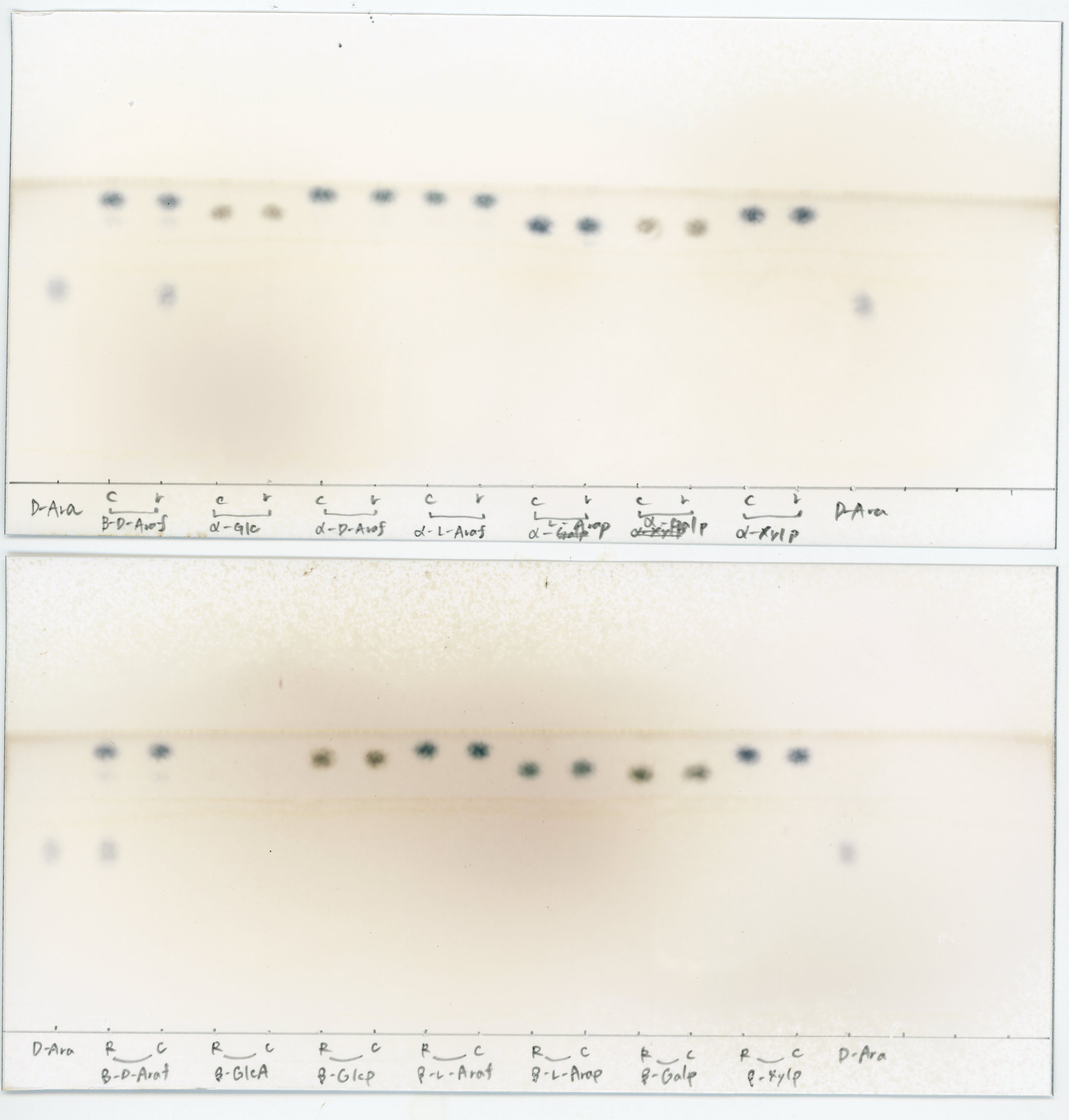

Supplement: Supplementary file 8 — Source Data [file 41467_2023_41431_MOESM8_ESM.zip › Source Data/SupFig11a2_TLC.jpg]

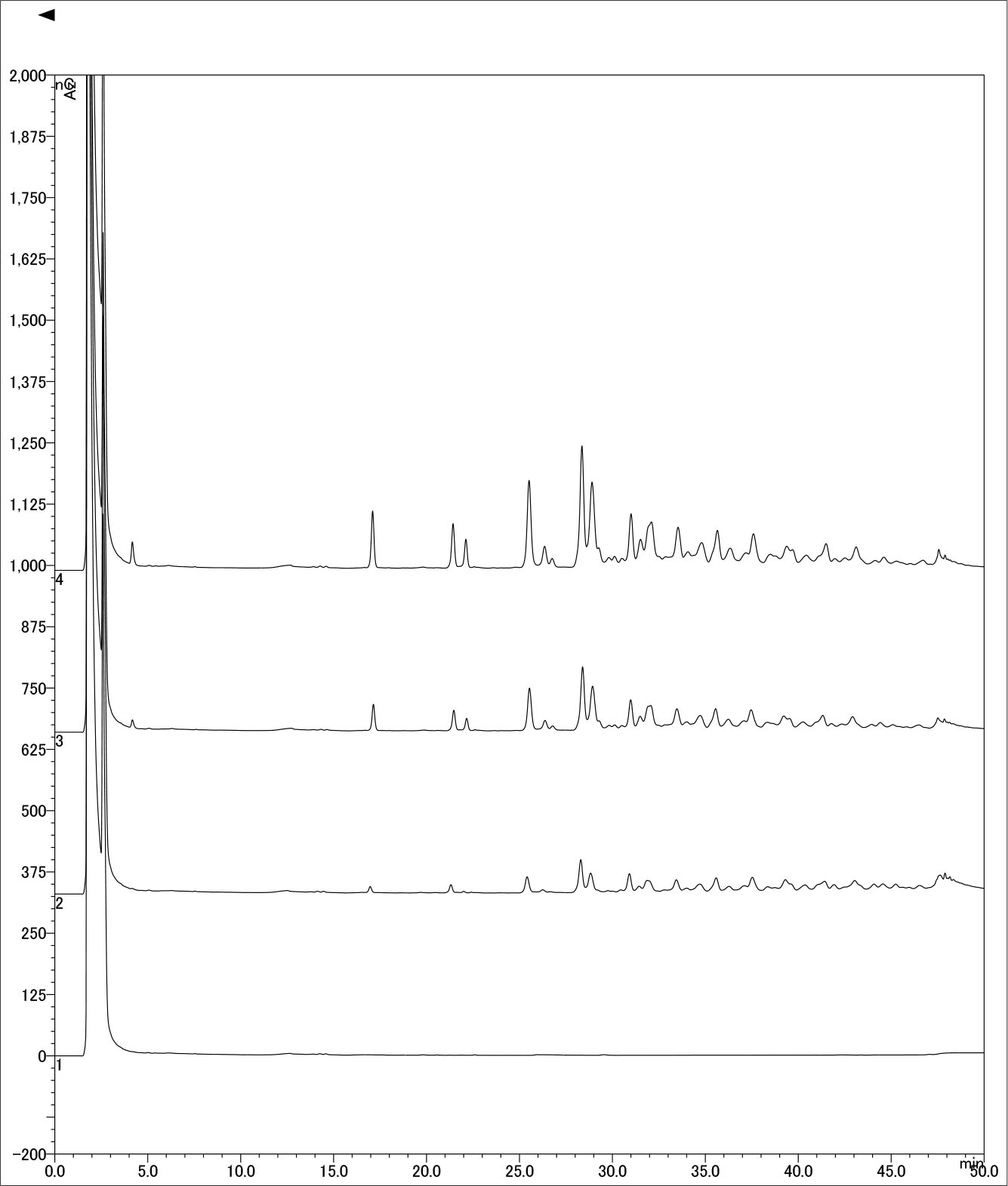

Supplement: Supplementary file 8 — Source Data [file 41467_2023_41431_MOESM8_ESM.zip › Source Data/SupFig1b.jpg]

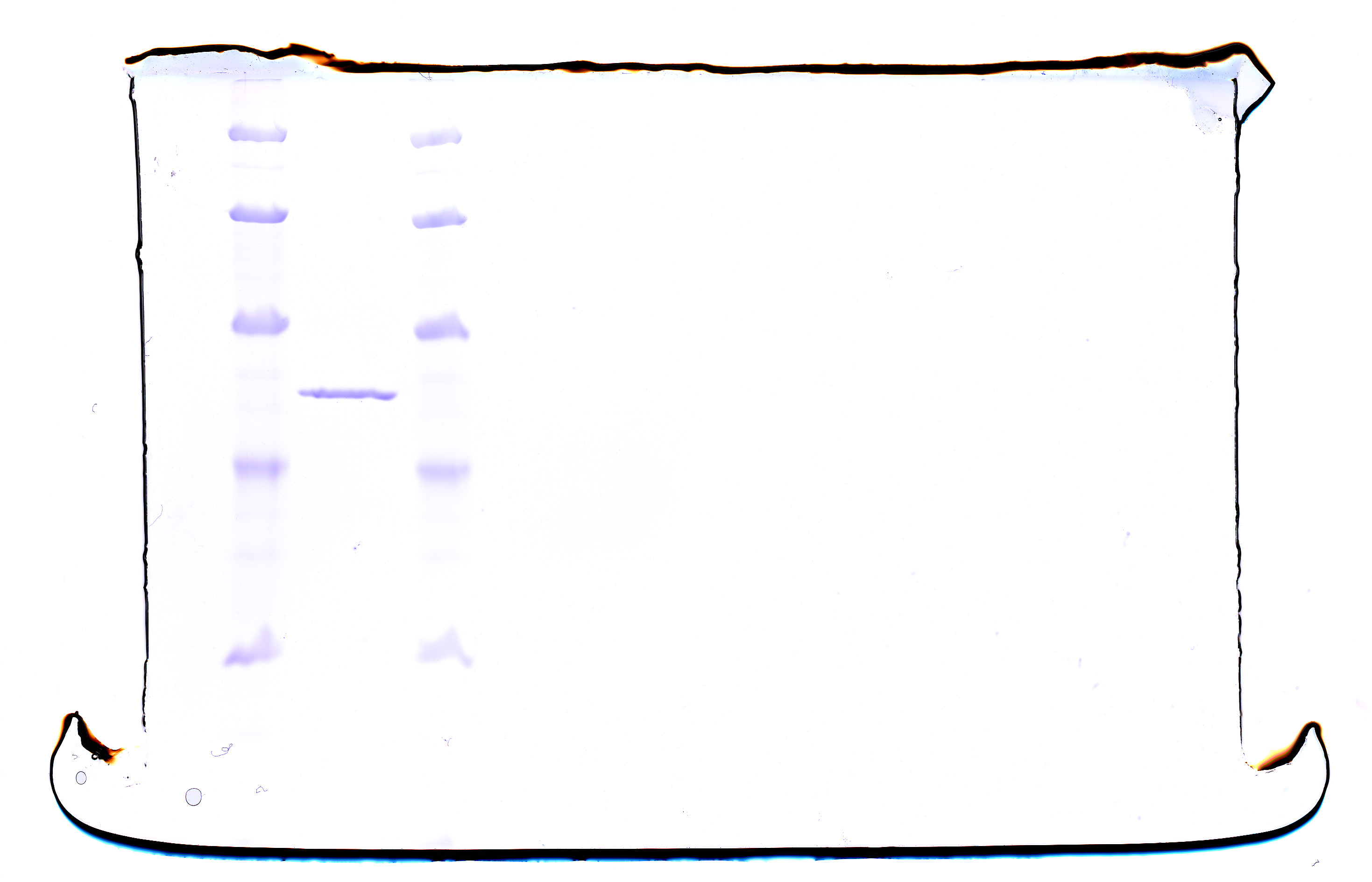

Supplement: Supplementary file 8 — Source Data [file 41467_2023_41431_MOESM8_ESM.zip › Source Data/SupFig5_a_SDS.jpg]

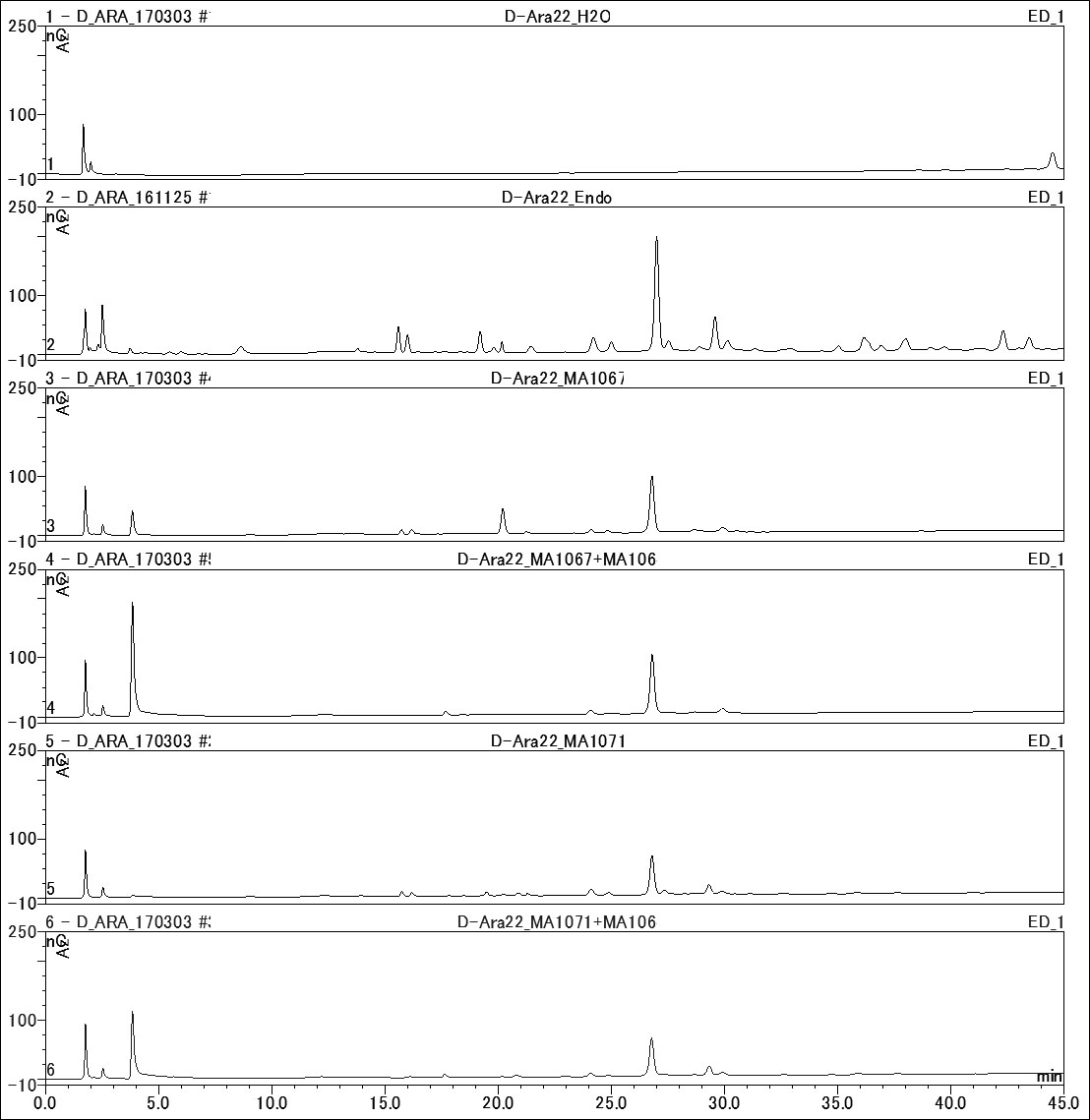

Supplement: Supplementary file 8 — Source Data [file 41467_2023_41431_MOESM8_ESM.zip › Source Data/SupFig6_a1.jpg]

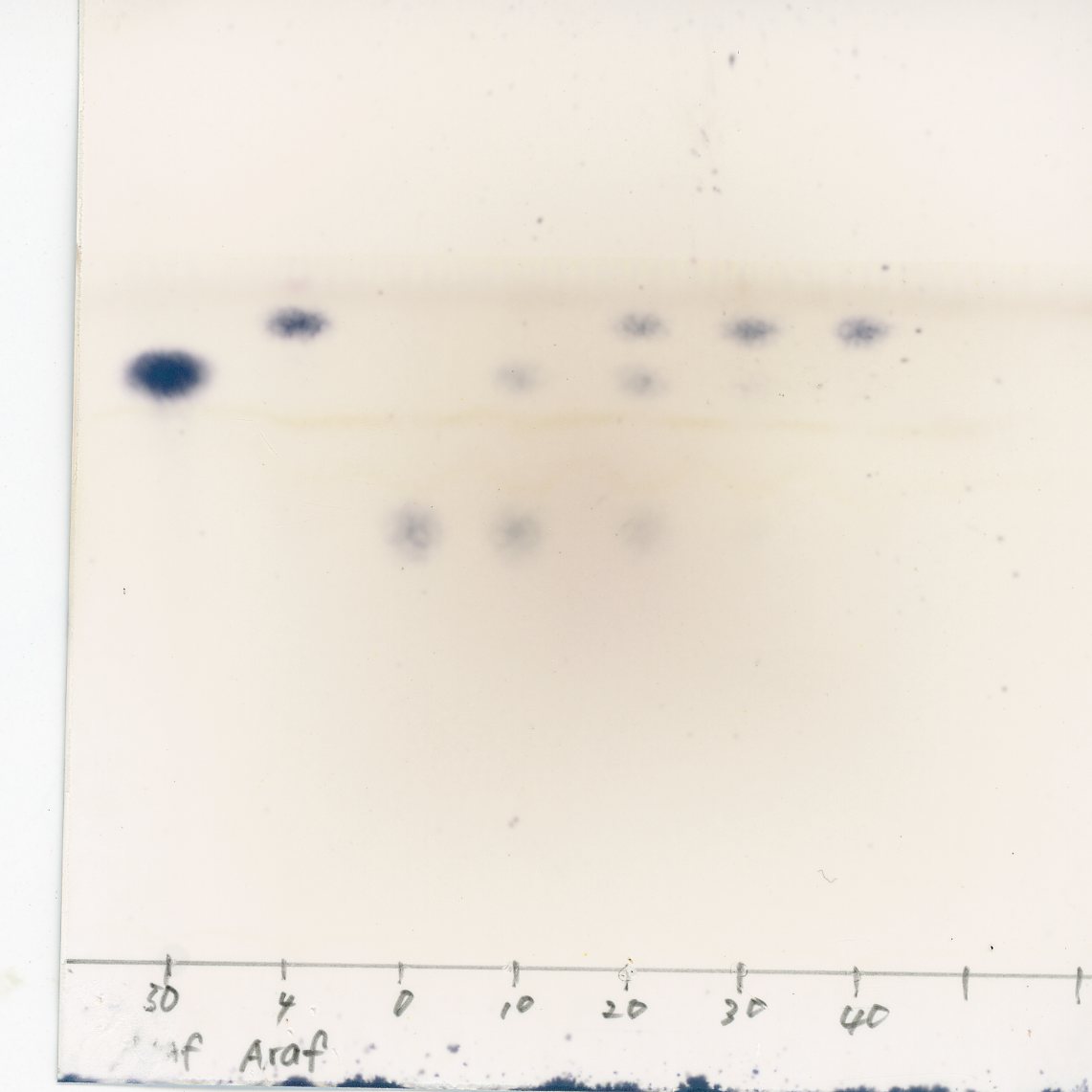

Supplement: Supplementary file 8 — Source Data [file 41467_2023_41431_MOESM8_ESM.zip › Source Data/Fig5e_TLC.jpg]

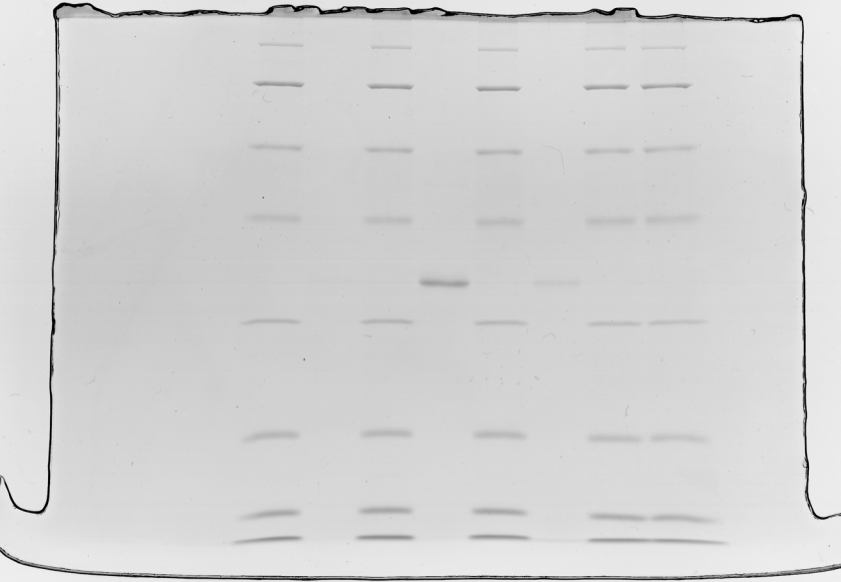

Supplement: Supplementary file 8 — Source Data [file 41467_2023_41431_MOESM8_ESM.zip › Source Data/Fig1_b_SDS.pdf]

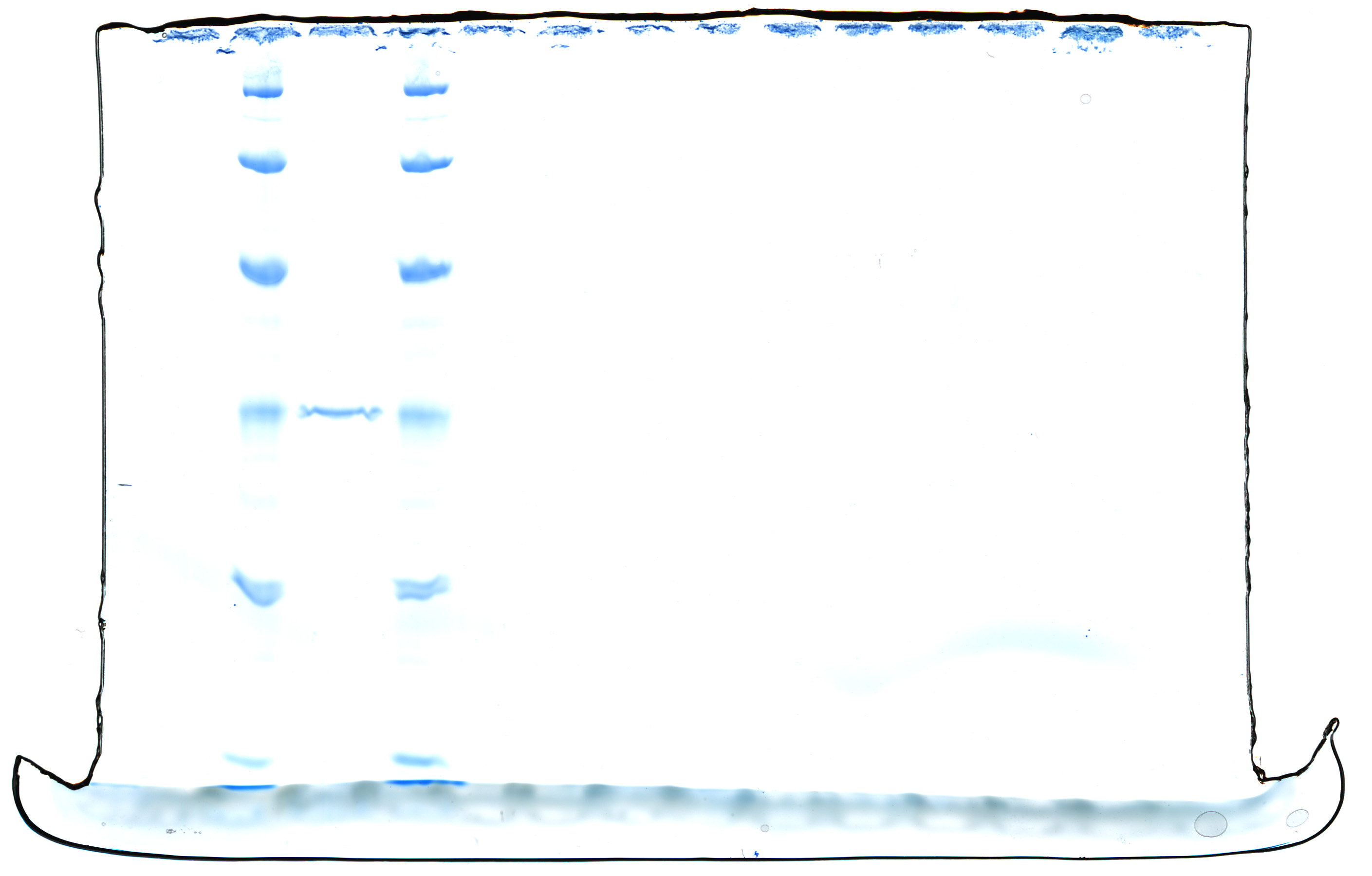

Supplement: Supplementary file 8 — Source Data [file 41467_2023_41431_MOESM8_ESM.zip › Source Data/SupFig9a_SDS.jpg]

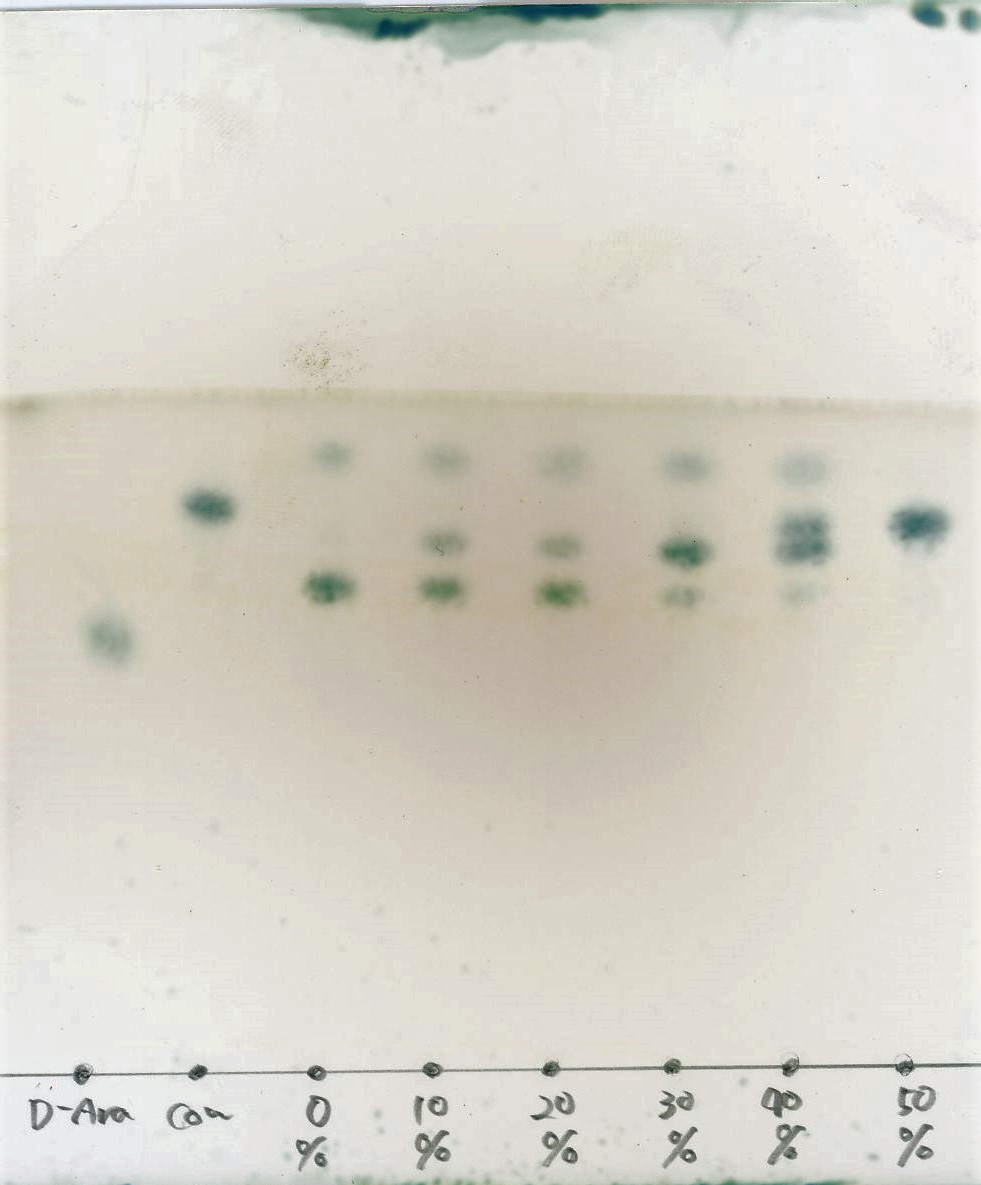

Supplement: Supplementary file 8 — Source Data [file 41467_2023_41431_MOESM8_ESM.zip › Source Data/Fig5_a.jpg]

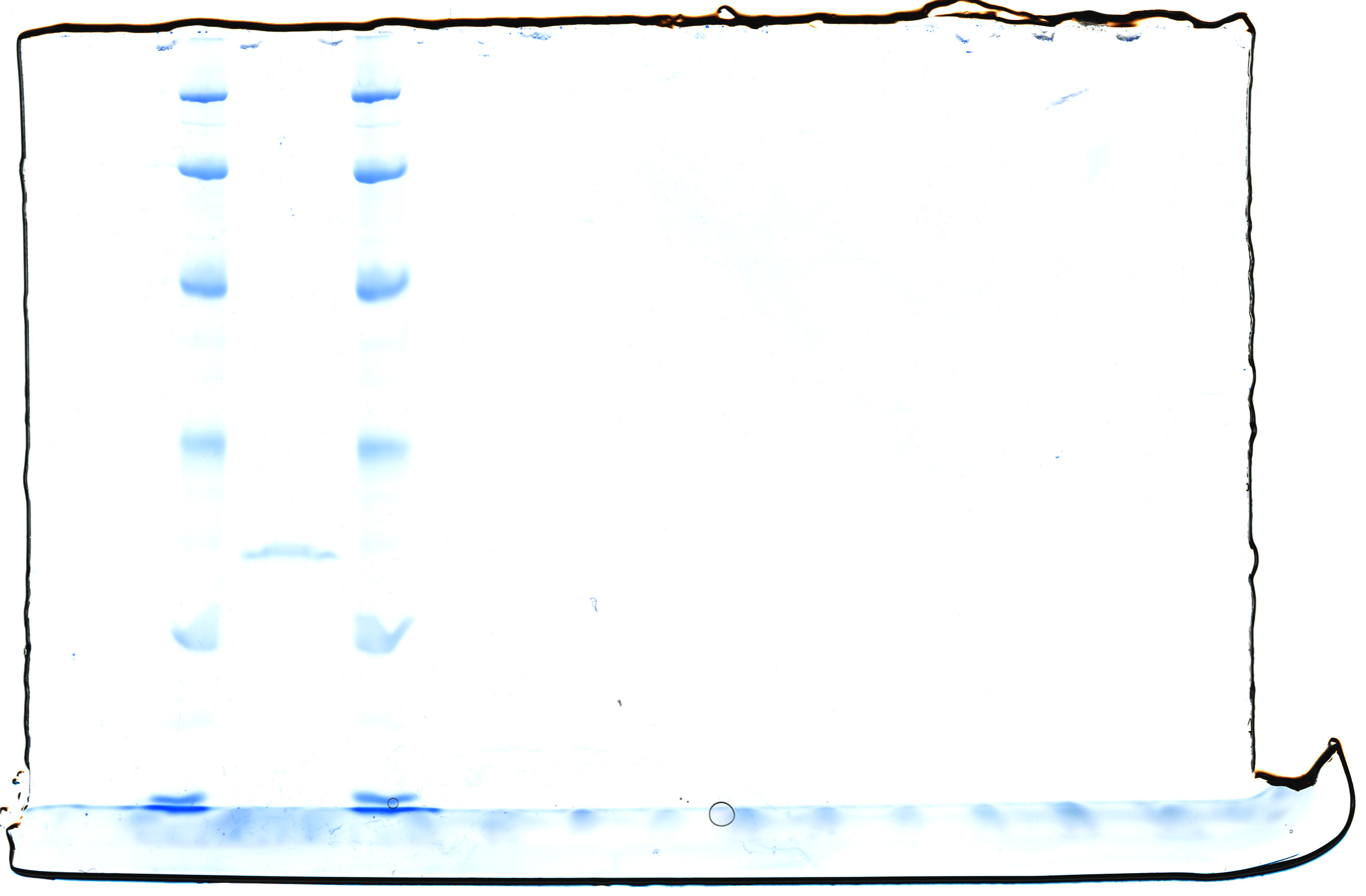

Supplement: Supplementary file 8 — Source Data [file 41467_2023_41431_MOESM8_ESM.zip › Source Data/SupFig5_b_SDS.jpg]

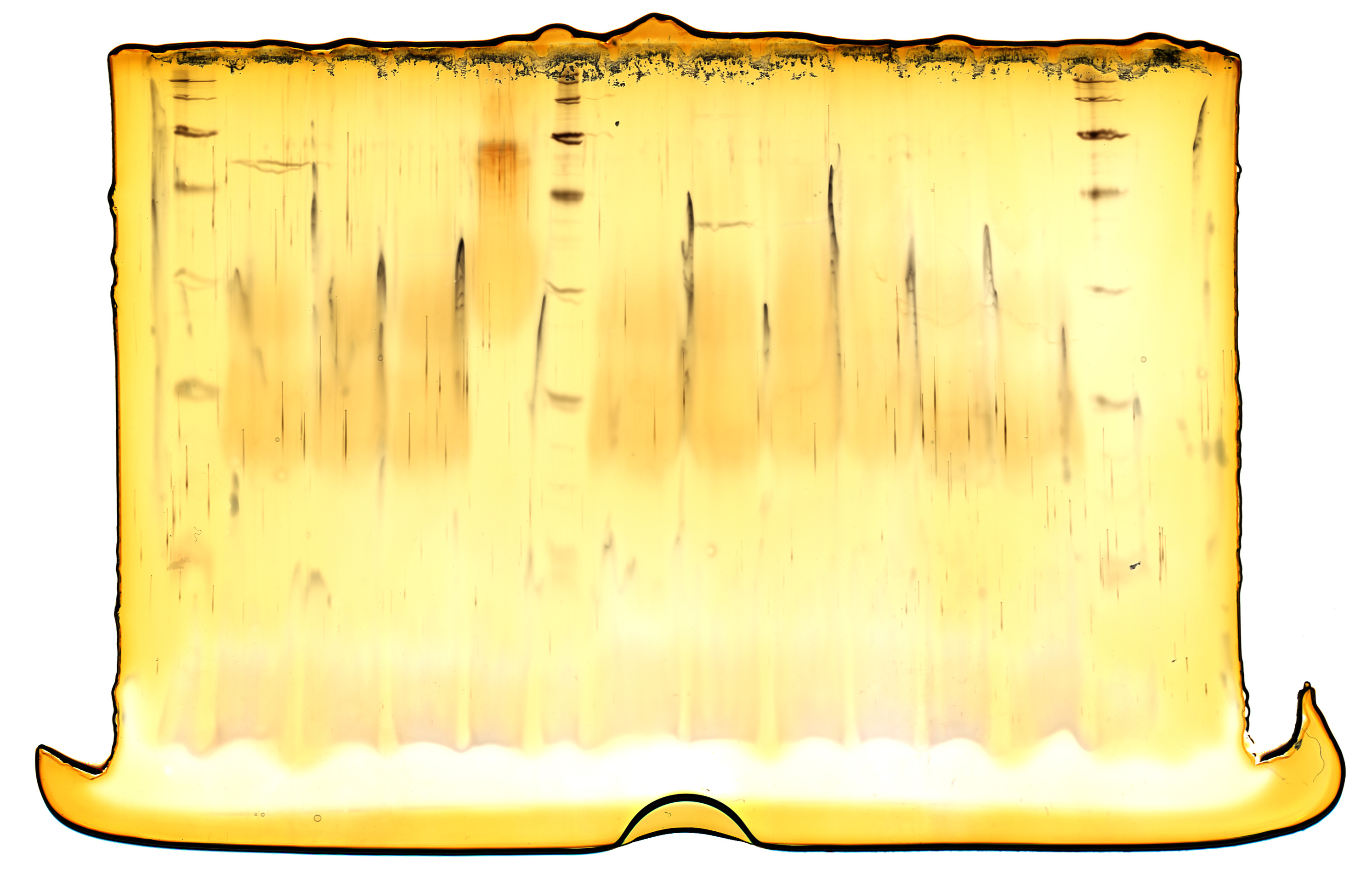

Supplement: Supplementary file 8 — Source Data [file 41467_2023_41431_MOESM8_ESM.zip › Source Data/Fig1_c_LAM.jpg]

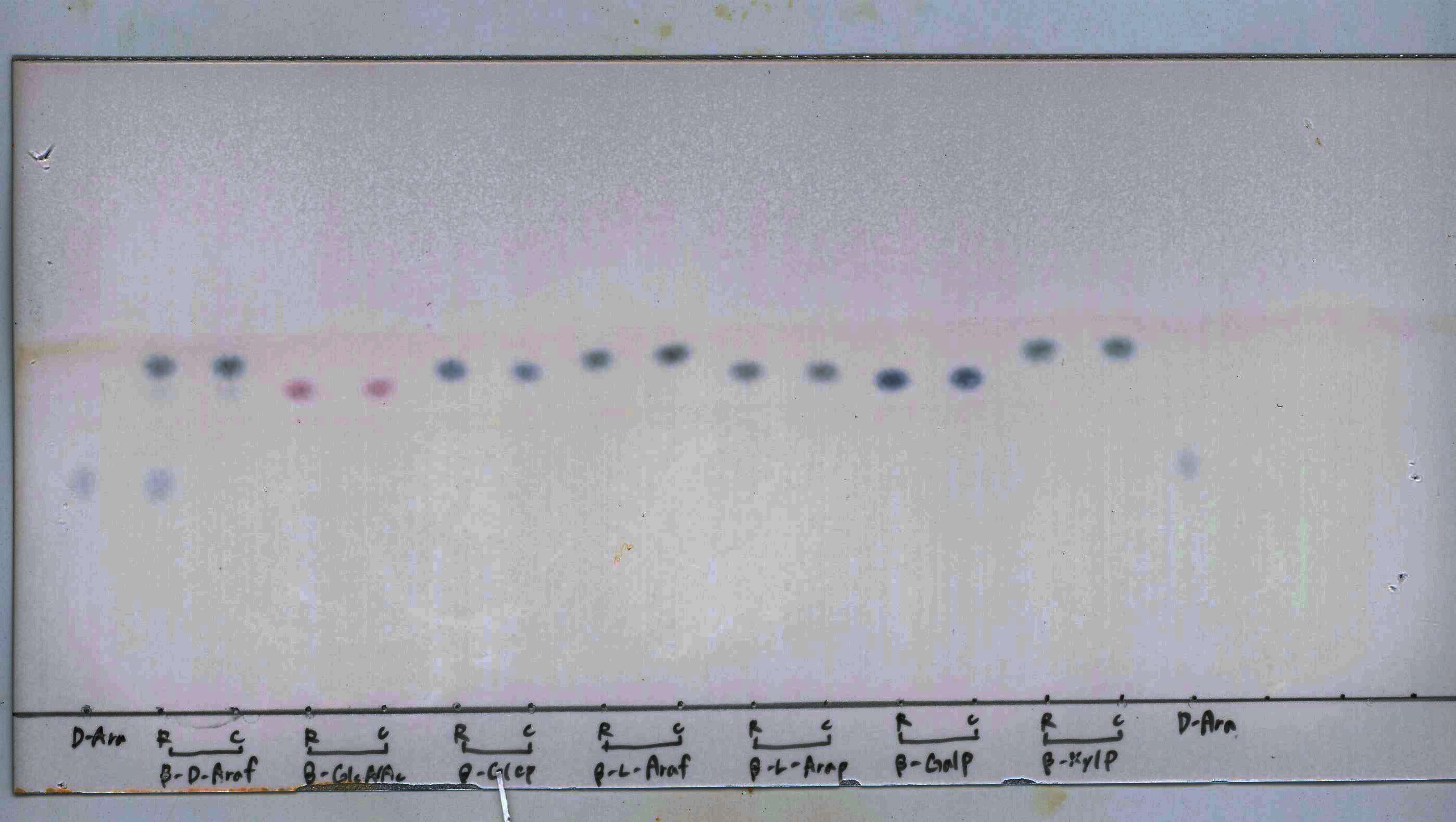

Supplement: Supplementary file 8 — Source Data [file 41467_2023_41431_MOESM8_ESM.zip › Source Data/SupFig11a1_TLC.jpg]

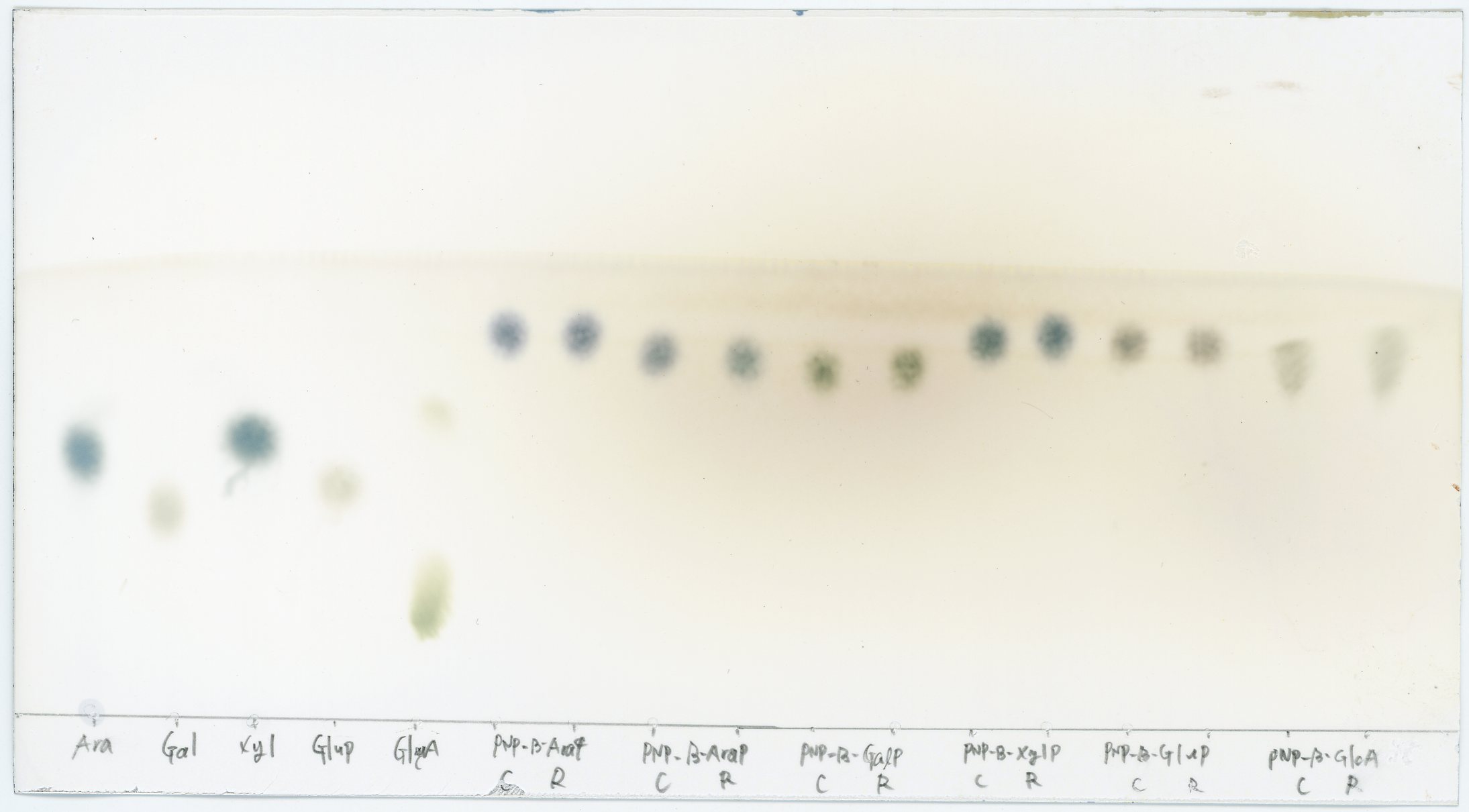

Supplement: Supplementary file 8 — Source Data [file 41467_2023_41431_MOESM8_ESM.zip › Source Data/SupFig10a2.jpg]

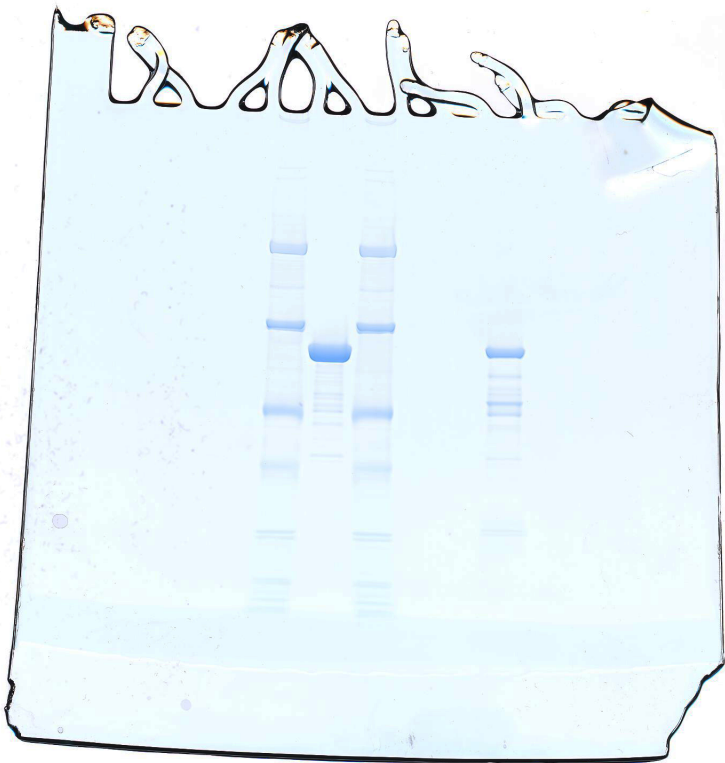

Supplement: Supplementary file 8 — Source Data [file 41467_2023_41431_MOESM8_ESM.zip › Source Data/SupFig9b_sds.pdf]

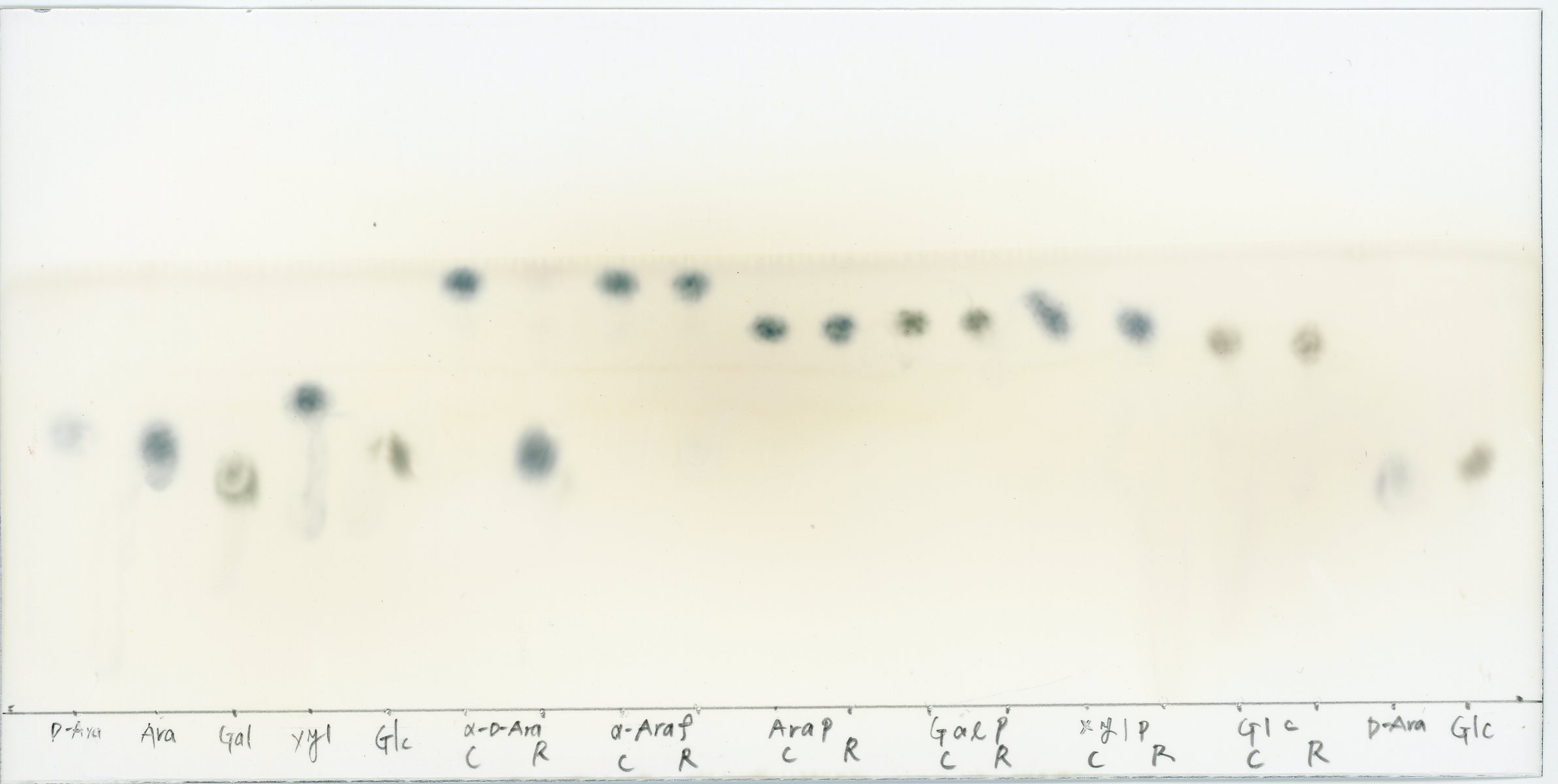

Supplement: Supplementary file 8 — Source Data [file 41467_2023_41431_MOESM8_ESM.zip › Source Data/SupFig10a1.jpg]

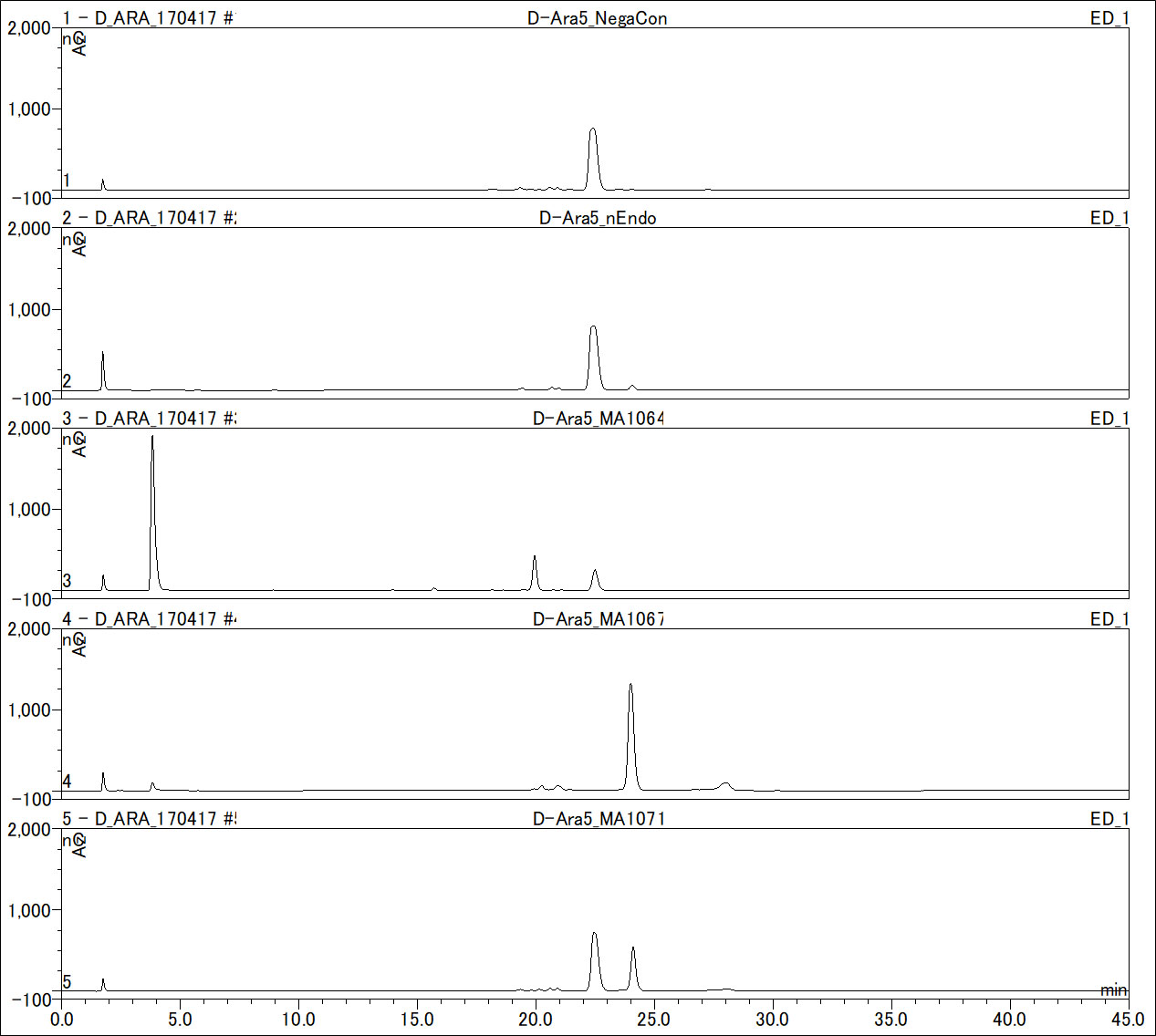

Supplement: Supplementary file 8 — Source Data [file 41467_2023_41431_MOESM8_ESM.zip › Source Data/SupFig6_d1.jpg]

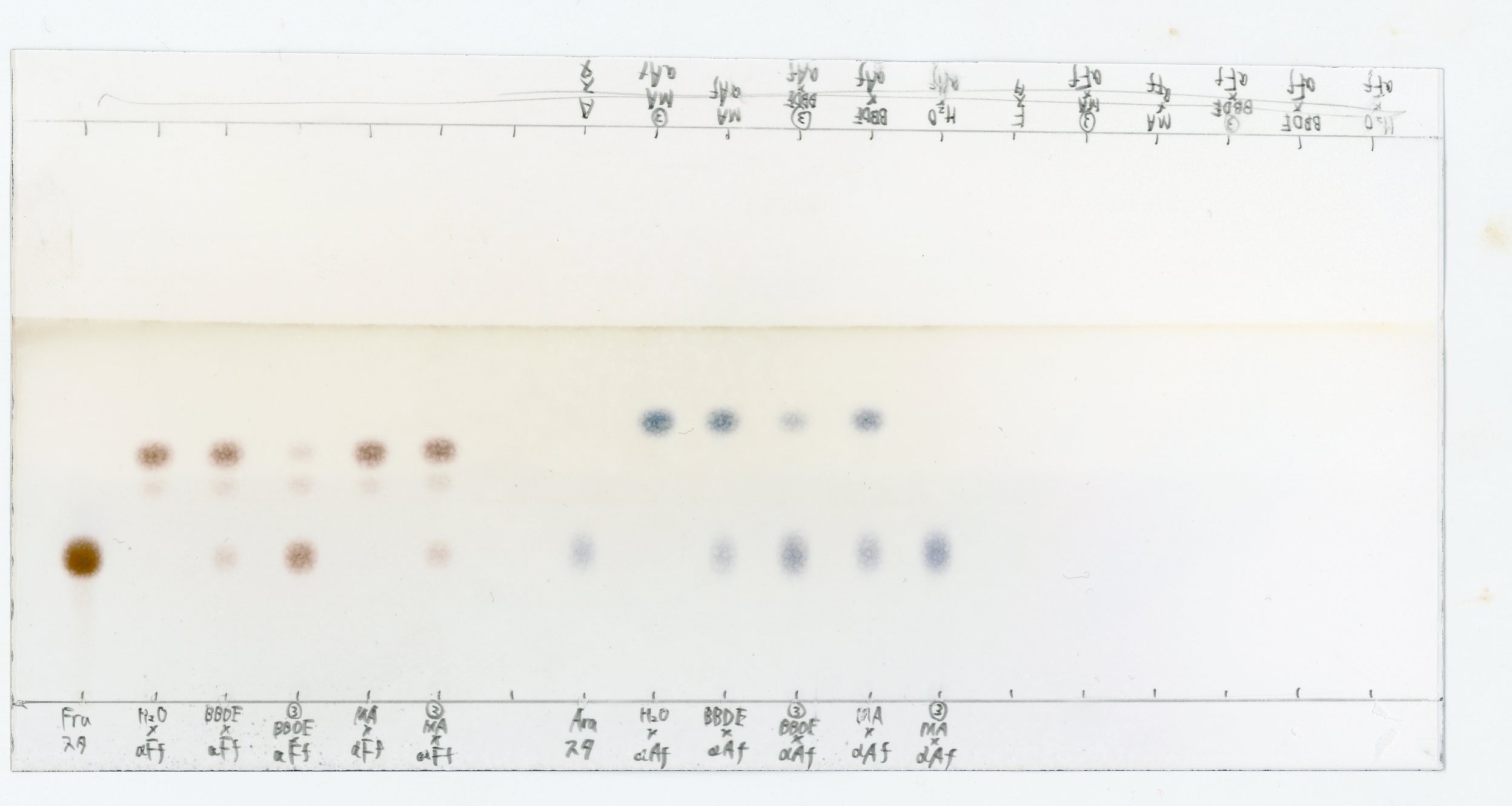

Supplement: Supplementary file 8 — Source Data [file 41467_2023_41431_MOESM8_ESM.zip › Source Data/Fig5c_TLC.jpg]

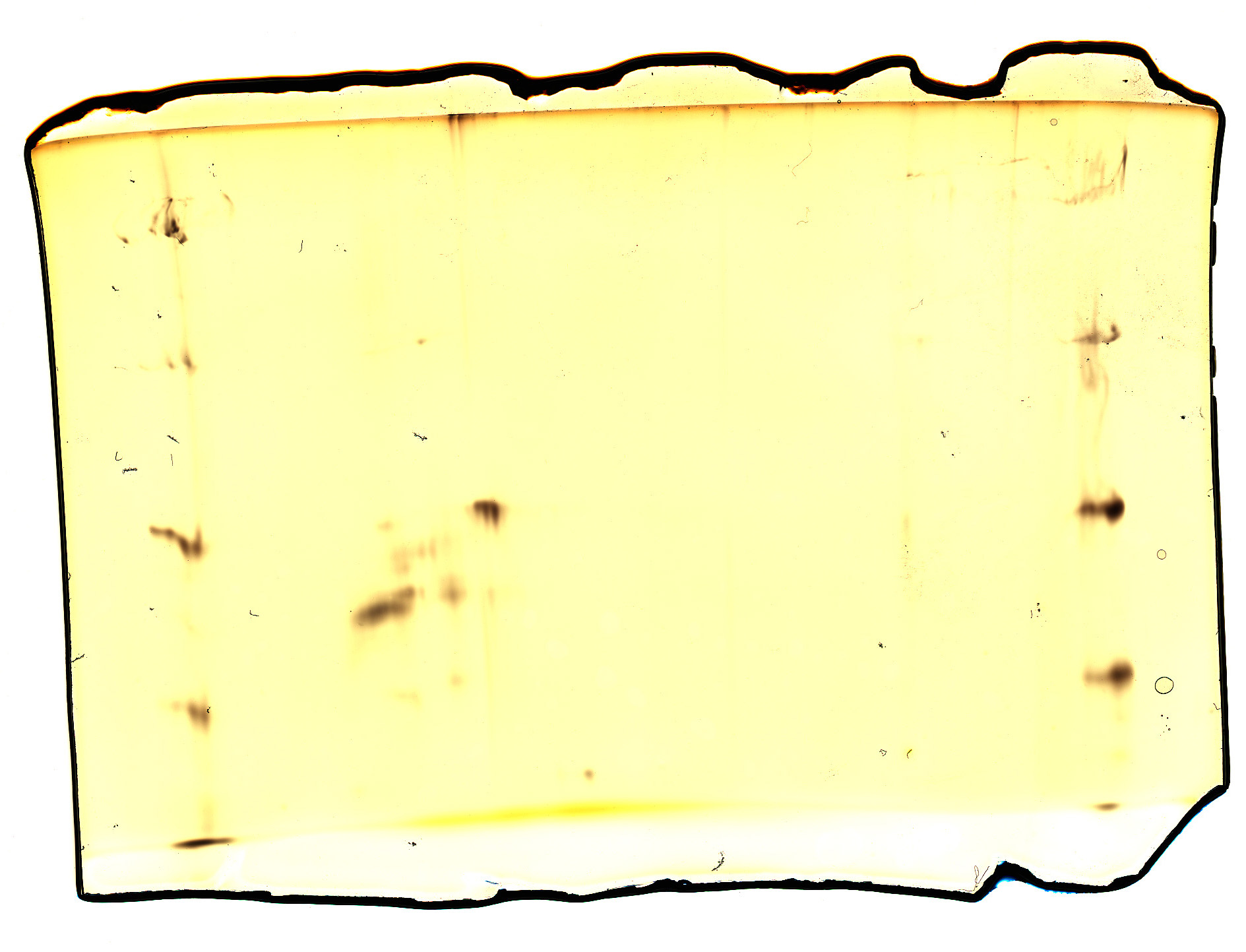

Supplement: Supplementary file 8 — Source Data [file 41467_2023_41431_MOESM8_ESM.zip › Source Data/SupFig2a_2D.jpg]

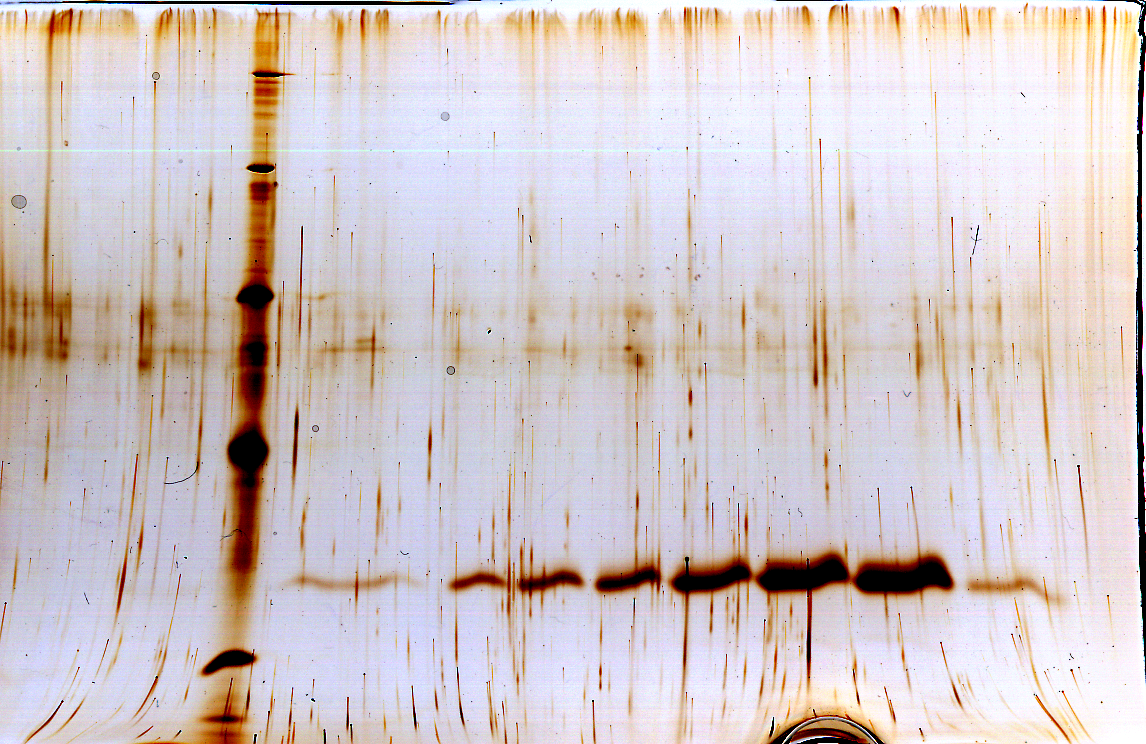

Supplement: Supplementary file 8 — Source Data [file 41467_2023_41431_MOESM8_ESM.zip › Source Data/Fig2C_SDS.bmp]

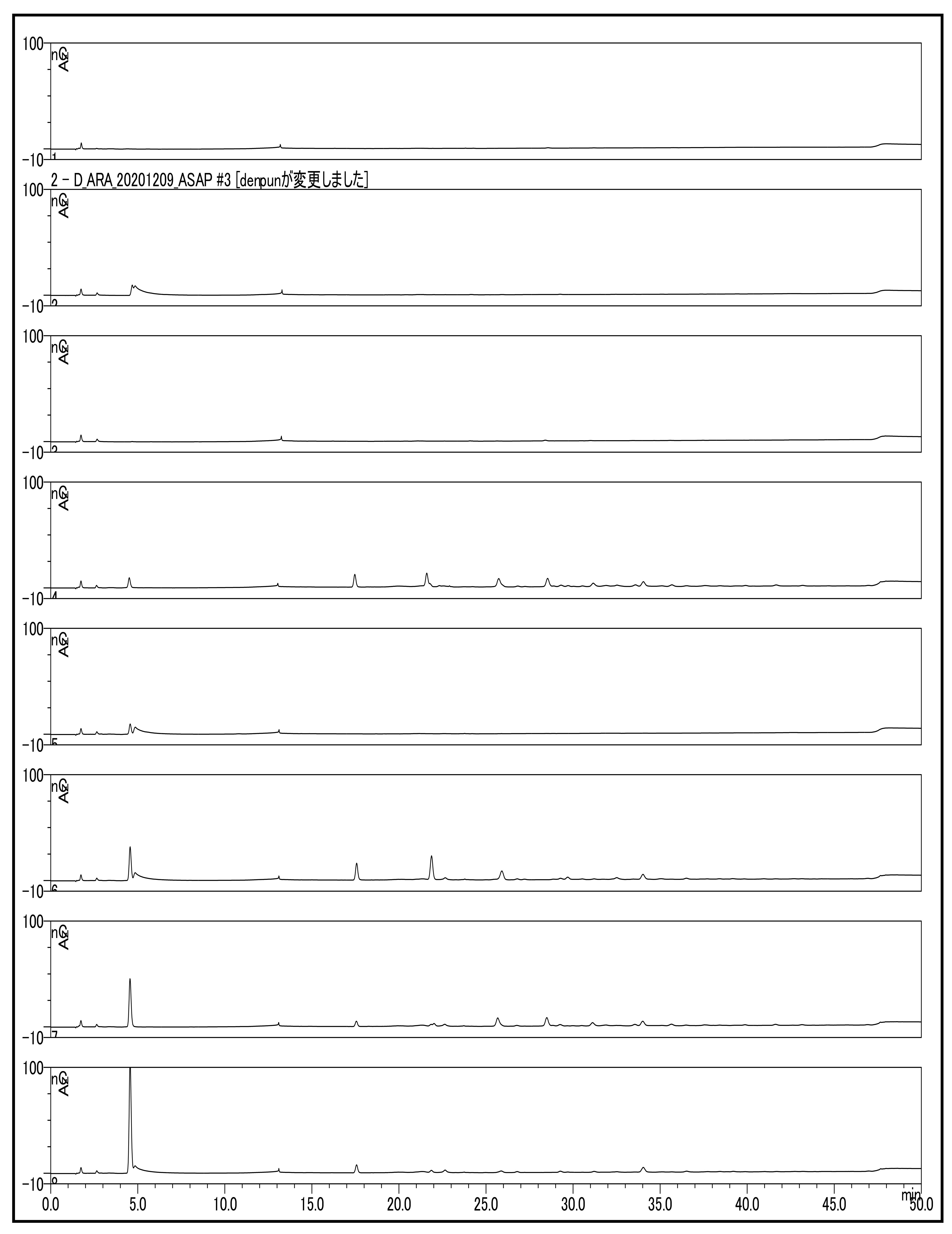

Supplement: Supplementary file 8 — Source Data [file 41467_2023_41431_MOESM8_ESM.zip › Source Data/SupFig13a.jpg]

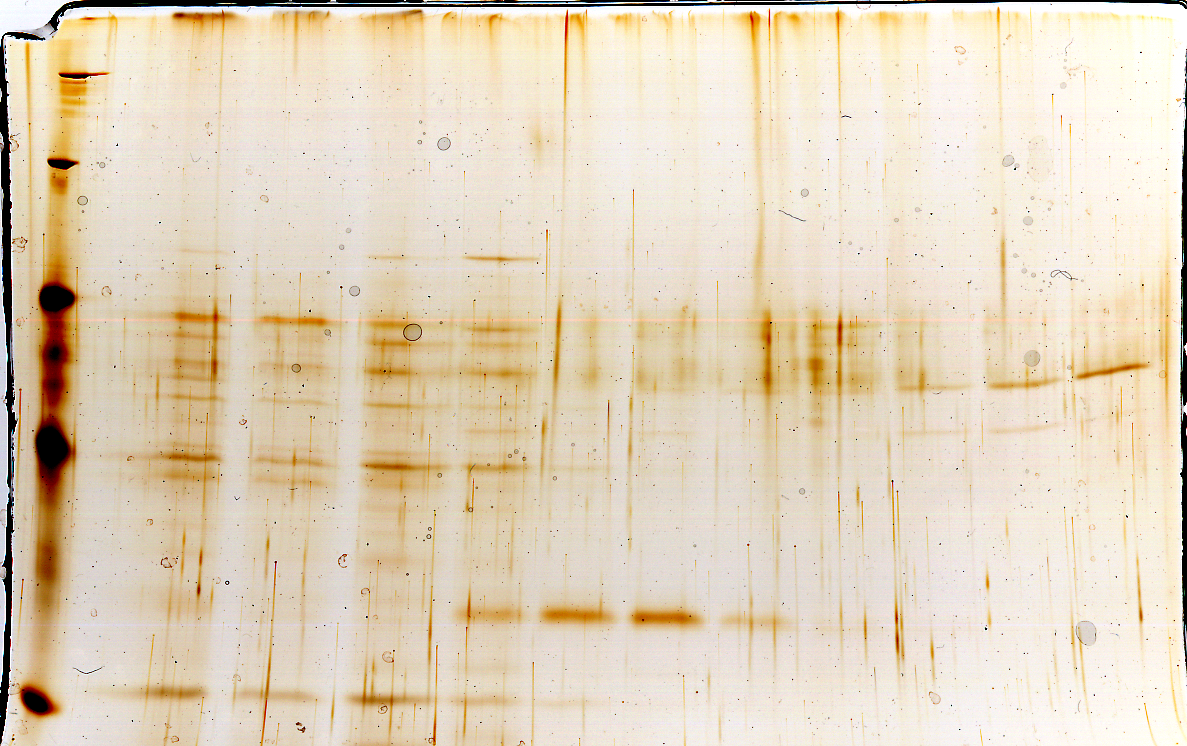

Supplement: Supplementary file 8 — Source Data [file 41467_2023_41431_MOESM8_ESM.zip › Source Data/Fig2b_SDS.bmp]

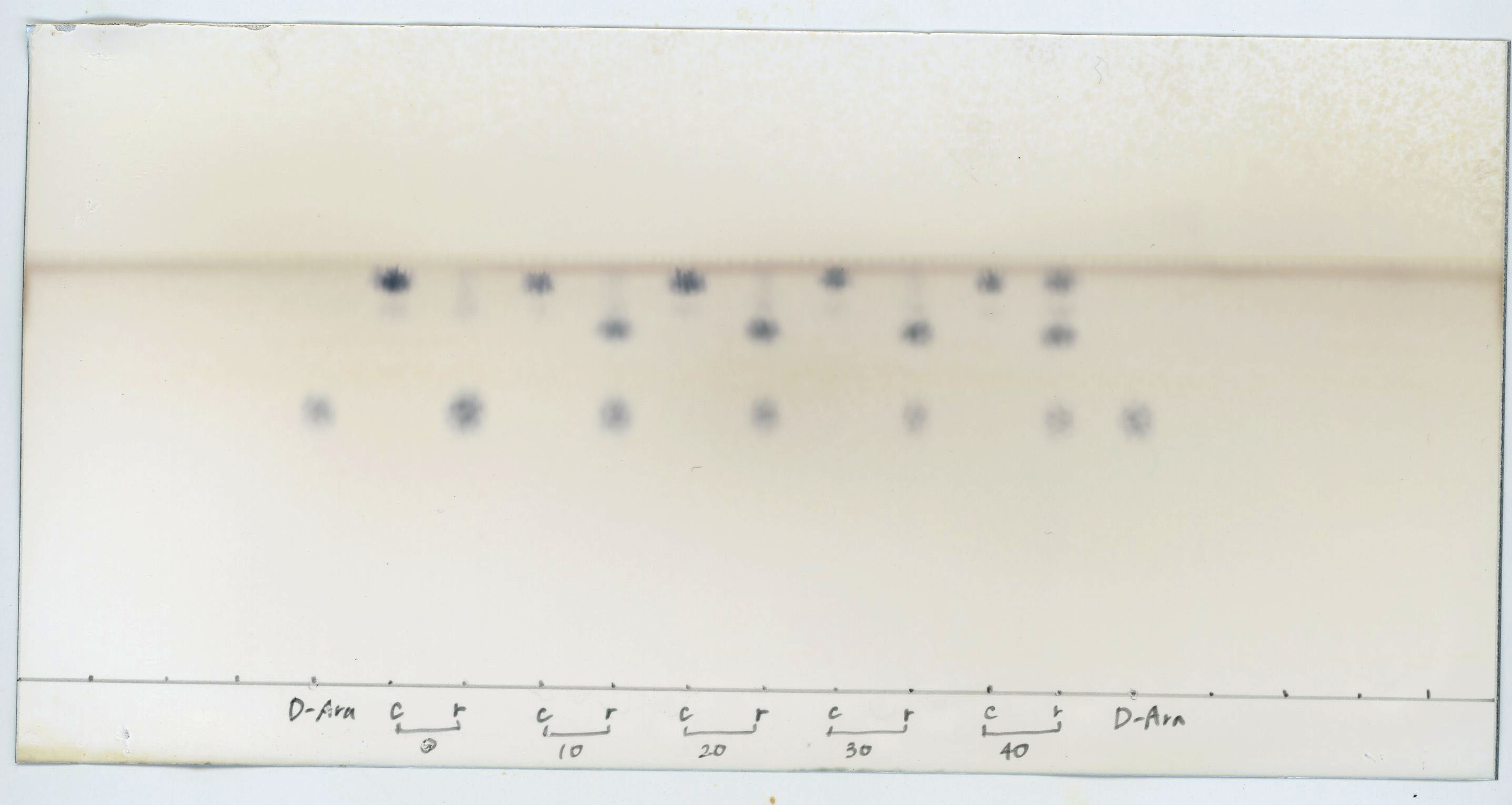

Supplement: Supplementary file 8 — Source Data [file 41467_2023_41431_MOESM8_ESM.zip › Source Data/Fig5f.jpg]

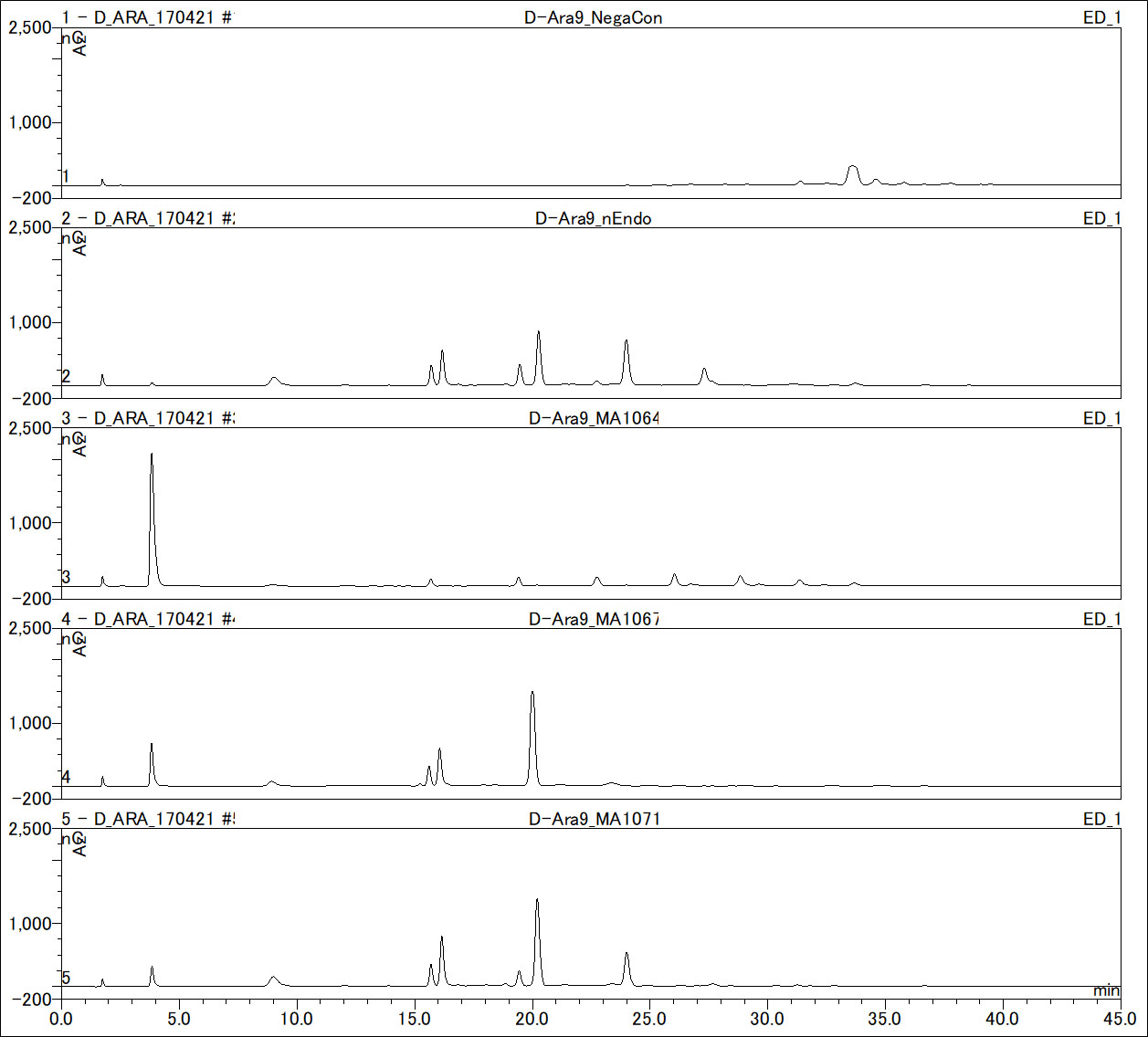

Supplement: Supplementary file 8 — Source Data [file 41467_2023_41431_MOESM8_ESM.zip › Source Data/SupFig6_b1.jpg]

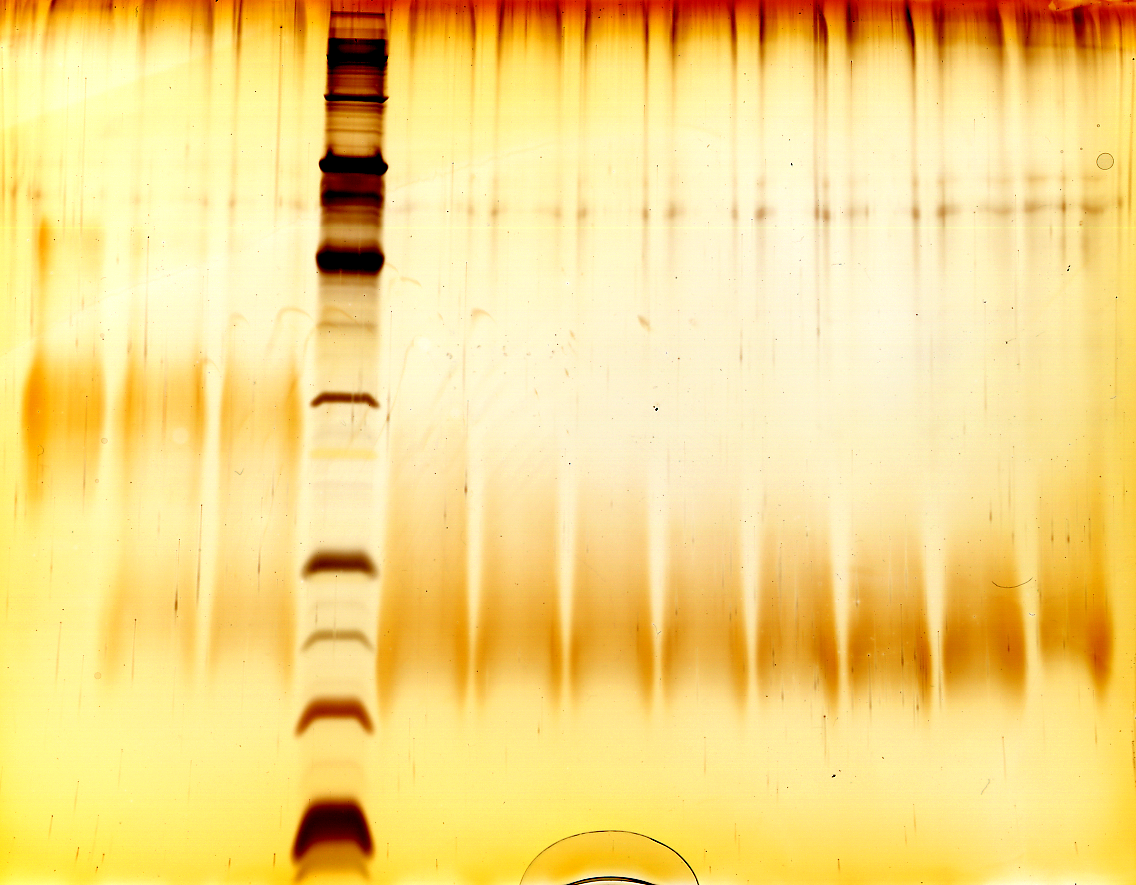

Supplement: Supplementary file 8 — Source Data [file 41467_2023_41431_MOESM8_ESM.zip › Source Data/Fig2C_LAM2.bmp]

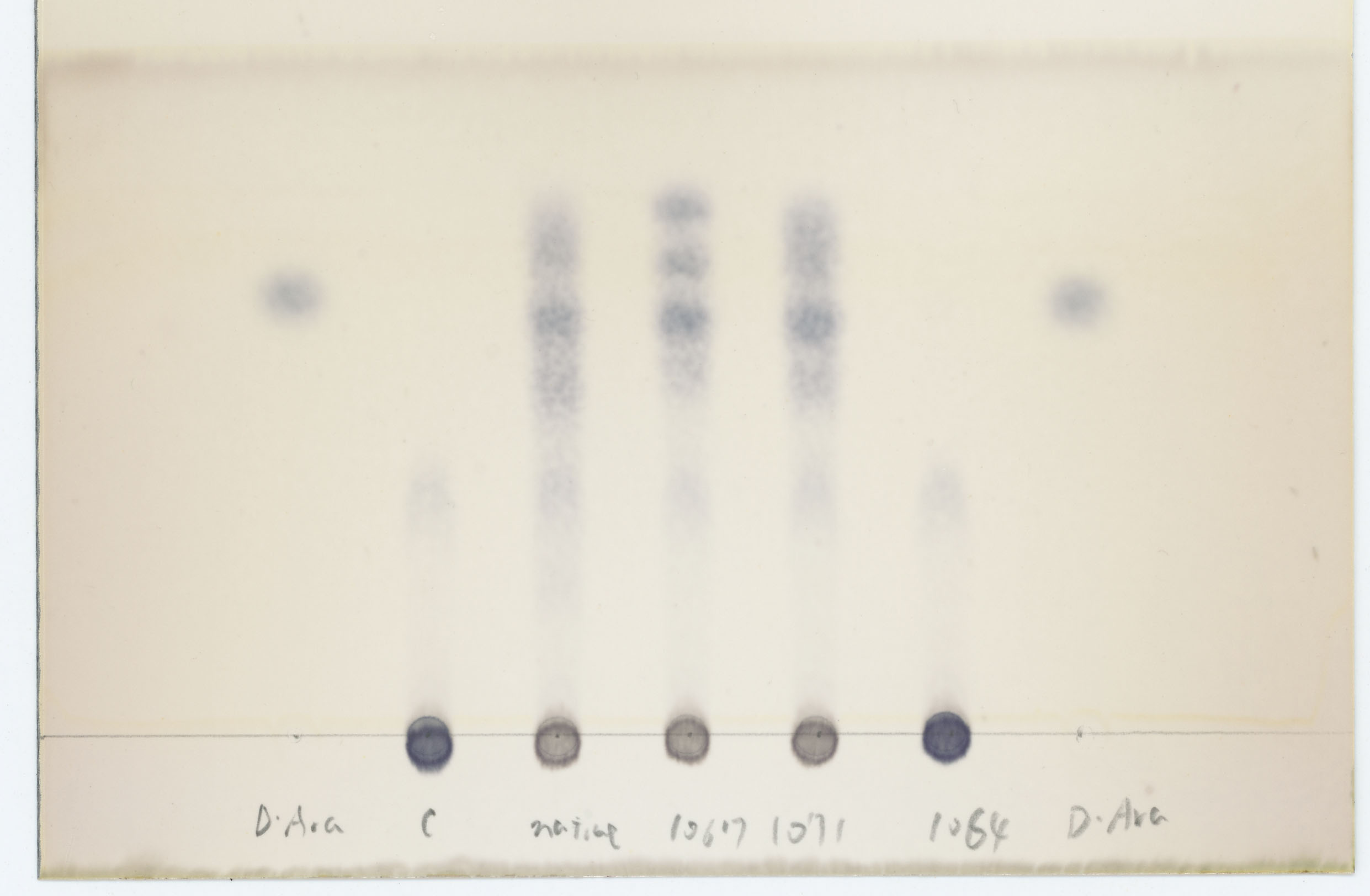

Supplement: Supplementary file 8 — Source Data [file 41467_2023_41431_MOESM8_ESM.zip › Source Data/Fig3b_TLC.jpg]

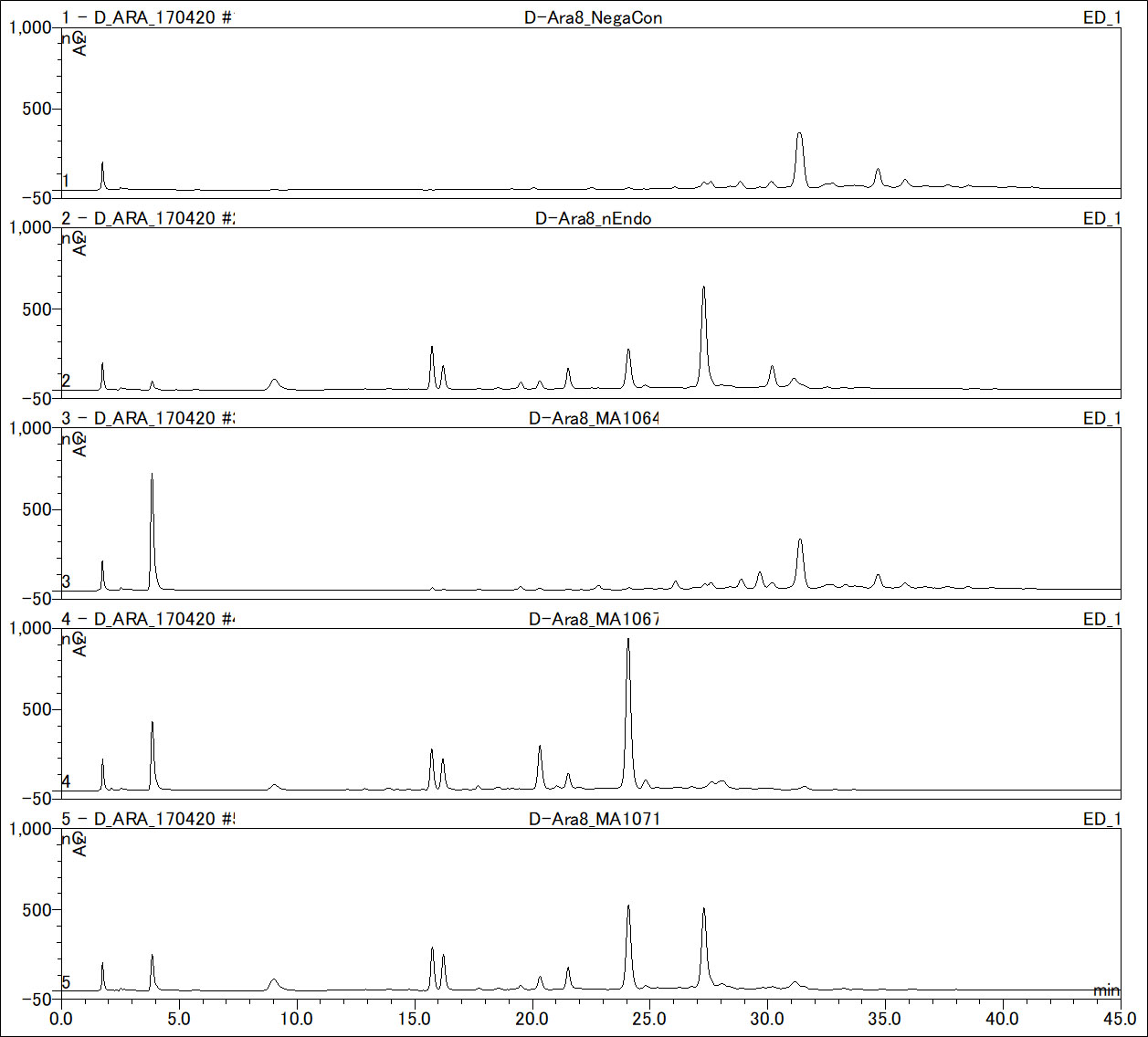

Supplement: Supplementary file 8 — Source Data [file 41467_2023_41431_MOESM8_ESM.zip › Source Data/SupFig6_c1.jpg]

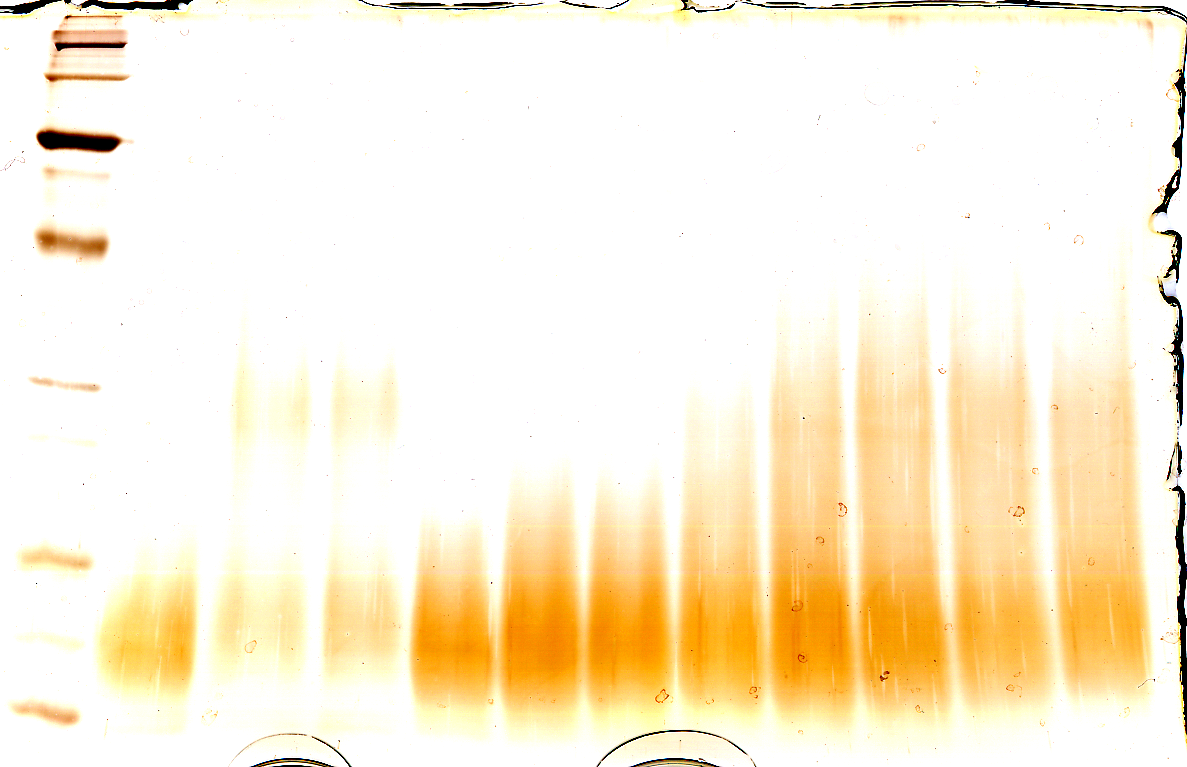

Supplement: Supplementary file 8 — Source Data [file 41467_2023_41431_MOESM8_ESM.zip › Source Data/Fig2b_LAM.bmp]
